# Single cell expression analysis of primate-specific retroviruses-derived HPAT lincRNAs in viable human blastocysts identifies embryonic cells co-expressing genetic markers of multiple lineages

**DOI:** 10.1016/j.heliyon.2018.e00667

**Published:** 2018-06-28

**Authors:** Gennadi Glinsky, Jens Durruthy-Durruthy, Mark Wossidlo, Edward J. Grow, Jason L. Weirather, Kin Fai Au, Joanna Wysocka, Vittorio Sebastiano

**Affiliations:** a Institute of Engineering in Medicine, University of California, San Diego, 9500 Gilman Dr. MC 0435, La Jolla, CA 92093-0435, USA; b Department of Obstetrics and Gynecology, Institute for Stem Cell Biology & Regenerative Medicine, Stanford University, Stanford, CA 94305, USA; c Department of Cell- and Developmental Biology, Center of Anatomy and Cell Biology, Schwarzspanierstrasse 17, 1090 Vienna, Austria; d Department of Chemical and Systems Biology, Stanford University, Stanford, California, USA; e Department of Internal Medicine, University of Iowa, Iowa City, IA, USA; f Department of Biostatistics, University of Iowa, Iowa City, IA, USA

**Keywords:** Developmental biology

## Abstract

Chromosome instability and aneuploidies occur very frequently in human embryos, impairing proper embryogenesis and leading to cell cycle arrest, loss of cell viability, and developmental failures in 50–80% of cleavage-stage embryos. This high frequency of cellular extinction events represents a significant experimental obstacle challenging analyses of individual cells isolated from human preimplantation embryos. We carried out single cell expression profiling of 241 individual cells recovered from 32 human embryos during the early and late stages of viable human blastocyst (VHB) differentiation. Classification of embryonic cells was performed solely based on expression patterns of human pluripotency-associated transcripts (*HPAT*), which represent a family of primate-specific transposable element-derived lincRNAs highly expressed in human embryonic stem cells and regulating nuclear reprogramming and pluripotency induction. We then validated our findings by analyzing transcriptomes of 1,708 individual cells recovered from more than 100 human embryos and 259 mouse cells from more than 40 mouse embryos at different stages of preimplantation embryogenesis. *HPAT*'s expression-guided spatiotemporal reconstruction of human embryonic development inferred from single-cell expression analysis of VHB differentiation enabled identification of telomerase-positive embryonic cells co-expressing key pluripotency regulatory genes and genetic markers of three major lineages. Follow-up validation analyses confirmed the emergence in human embryos prior to lineage segregation of telomerase-positive cells co-expressing genetic markers of multiple lineages. Observations reported in this contribution support the hypothesis of a developmental pathway of creation embryonic lineages and extraembryonic tissues from telomerase-positive pre-lineage cells manifesting multi-lineage precursor phenotype.

## 1 Introduction

Precise spatio-temporal activation of primate-specific transposable genetic elements (TGE), and particularly of regulatory sequences derived from human endogenous retroviruses (HERVs), have been associated with the induction and maintenance of human pluripotency, functional identity and integrity of naïve-state embryonic stem cells, and anti-viral resistance of the early-stage human embryos [1, 2, 3, 4, 5, 6, 7, 8, 9, 10, 11, 12, 13, 14, 15, 16, 17]. These studies identified long intergenic non-coding RNAs (lincRNAs) derived from HERVs as the reliable genetic markers and critically important regulators of the pluripotent state in human cells [8, 14, 15, 17]. Active expression of lincRNAs encoded by specific HERVs is required for pluripotency maintenance in hESC [8, 17] and plays essential functional roles in naïve-like hESC [15, 16], development of human preimplantation embryos [16, 17], and pluripotency induction during nuclear reprogramming [9, 10, 15, 17]. Individual members of one functionally and structurally-related family of TGE-derived lincRNAs, which are highly expressed in hESC and termed human pluripotency-associated transcripts (*HPATs*), have been shown to affect the regulation of nuclear reprogramming and pluripotency induction during preimplantation embryogenesis [17]. Despite this significant progress facilitated by single-cell analyses of hESCs and human embryos, our understanding of how the earliest lineage commitments are regulated and individual cell fate decisions are executed remains limited. In particular, the expression dynamics and biological roles of TGE-derived lincRNAs in these processes are not fully understood.

Several methodological hurdles and fundamental problems complicate the analysis of human preimplantation embryogenesis. Chromosome instability is common in the early-stage human embryonic development and aneuploidies are observed in 50–80% of cleavage-stage human embryos leading to cell cycle arrest, impaired viability of embryonic cells' and developmental failures [18, 19, 20, 21, 22]. Therefore, without the application of reliable prospective controls of the viability of human embryonic cells, the likelihood of studying a significant fraction of dying human embryonic cells is high and should approach the 50–80% probability.

A systematic application of non-invasive assays that accurately predict the likelihood of viable blastocyst development during evaluation of the mature oocytes and/or zygotes should help to address this limitation. It has been shown that the analysis of zygote viscoelastic properties as well as cytokinesis timing in the early stages of human embryo development can predict viable blastocyst formation with the high degree of accuracy [22, 23]. The reported method predicts human blastocyst's viability with more than 90% precision, 95% specificity and 75% sensitivity [22, 23].

Another potentially significant limitation of single cell studies of human embryos is the use of the timeline after fertilization as end-point for isolating individual human embryonic cells. This approach would likely generate developmentally heterogeneous mixes of single cells isolated from human embryos that are not fully synchronized and will have varying proportions of dying versus viable cells at any given developmental time point.

To address these limitations, we implemented an experimental approach ensuring the high likelihood of studying truly viable human embryonic cells and employing the strict morphological criteria for segregation of viable blastocyst cells into the early and late blastocyst developmental stages defined by classic embryological criteria [24]. Using this approach, single-cell gene expression profiling was successfully carried-out in 241 individual cells recovered from early and late human blastocysts to delineate the spatiotemporal dynamics of protein-coding gene expression changes during human blastocyst differentiation [24]. It has been demonstrated that expression profiling of the relatively small number of selected protein-coding genes in this experimental system faithfully distinguished all three lineages that form the human blastocyst [24]. These experiments facilitated development of a three-dimensional *in silico* model of the inner cell mass and trophectoderm, in which individual cells were mapped into distinct expression domains corresponding to the lineage differentiation in human blastocysts [24]. This *in situ* model was then used to design the first web-based online tool to study early cell fate decisions in the human blastocyst, which is available online at http://web.stanford.edu/∼sunilpai/HumanBlastocystViewer.html (Firefox/Chrome compatible).

The main objective of this study was the single cell expression profiling analysis of recently discovered family of primate-specific retrotransposon-derived lincRNAs in human embryos and to compare and contrast our findings with the results of the single cell analyses of human preimplantation embryogenesis which were focused primarily on expression analyses of protein-coding genes. To achieve these goals, we employed the experimental system of viable human blastocyst (VHB) differentiation. We performed the targeted expression analysis of endogenous primate-specific retroviruses-derived lincRNAs in 241 individual cells recovered from early and late differentiation stages of VHB. In contrast to expression profiling analyses of VHB focused predominantly on protein-coding genes [24], the segregation of individual human blastocyst cells into distinct populations solely based on expression patterns of three primate-specific retrovirus HERVH-derived lincRNAs enables identification of telomerase-positive cells co-expressing genetic markers of multiple embryonic lineages. We confirmed our experimental findings and mechanistic models by *in silico* gene expression profiling analyses of four independent validation data sets comprising of 1,708 individual embryonic cells recovered from more than 100 human embryos at different stages of preimplantation embryogenesis [12, 13, 25, 26]. Based on these observations, we propose a hypothesis that telomerase-positive cells co-expressing genetic markers of multiple embryonic lineages may function as the multi-lineage precursor cells during human preimplantation embryogenesis. Our analyses suggest that emergence of the embryonic cells' population manifesting the Multi-Lineage Markers Expression phenotype (the MLME cells) at the E4 through E7 stages of human preimplantation embryogenesis may represent the alternative developmental pathway to the creation of the three major embryonic lineages as well as extraembryonic tissues.

## 2 Results

### 2.1 Single-cell expression profiling of *HPAT* lincRNAs' expression-defined populations during human blastocyst differentiation

Detailed analyses of expression patterns of *HPAT* lincRNAs in 241 individual human blastocyst cells revealed that essentially all cells express varying combinations of three lincRNAs, namely *HPAT21; HPAT2;* and *HPAT15,* and there is only one cell manifesting *HPAT21/HPAT2/HPAT15*-null phenotype (Supplemental Note 1; Supplemental Fig. S1B). It was of interest to identify and analyze gene expression signatures (GES) of the *HPAT* expression-defined populations of human blastocyst cells. All individual human blastocyst cells were segregated into sub-groups based on common expression patterns of three lincRNAs (*HPAT21; HPAT2;* and *HPAT15*) as well as other previously reported genetic markers of pluripotency and distinct embryonic lineages (Table 1; Supplemental Figs. S1 and S2). The results of the analyses of these *HPATs'* expression-defined populations of human embryonic cells are reported in this contribution.

**Table 1:** Classification of human blastocyst cells based on the expression patterns of 3 primate-specific retrotransposon-derived HPAT lincRNAs (HPAT21; HPAT2; and HPAT15).

Expression patterns of three HPAT lincRNAs	Expression phenotype	HPAT3 lincRNA expression	HPAT5 lincRNA expression	Expression of naïve pluripotency inducers & markers	TERT-positive cells in late blastocysts, %	Phenotype designation
*HPAT21*^*(+)*^*HPAT2*^*(−)*^*HPAT15*^*(−)*^	Single positive	DOWN	DOWN	DOWN	0.0%	Single-positive *HPAT21* cells (*spHPAT21*)
*HPAT21*^*(+)*^*HPAT2*^*(−)*^*HPAT15*^*(+)*^	Double positive	DOWN	DOWN	DOWN	3.5%	Double-positive *HPAT15* cells (*dpHPAT15*)
*HPAT21*^*(+)*^*HPAT2*^*(+)*^*HPAT15*^*(−)*^	Double positive	UP	UP	UP	29.0%	*HPAT2* positive cells (*HPAT2pos*)
*HPAT21*^*(+)*^*HPAT2*^*(+)*^*HPAT15*^*(+)*^	Triple positive	UP	UP	UP	56.0%	*HPAT2* positive cells (*HPAT2pos*)

A total of 241 individual human embryonic cells were recovered from viable human blastocysts and subjected to the single-cell gene expression profiling. Essentially all cells (240 of 241) were found to express three HPAT lincRNAs in various combinations. Human embryonic cells were then segregated into distinct sub-groups based on the expression profiles of 3 HPAT lincRNAs and analyzed for common and distinct expression patterns of genetic markers of distinct embryonic lineages.

Assignment of cells to distinct subgroups based on expression of three *HPAT lincRNAs* collectively captured 240 of 241 human embryonic cells isolated from VHB (Table 1; Supplemental Fig. S1A). The numbers of individual human embryonic cells comprising the corresponding *HPAT*'s expression-defined sub-populations of early (Supplemental Fig. S2F) and late (Supplemental Fig. S2G) blastocysts were quantified and each individual cell within these sub-populations was analyzed. The overall structure of the *HPAT*'s expression-defined sub-populations remains stable and appears very similar during the transition from the early to late blastocysts (Supplemental Figs. S1; S2E–S2G). Notably, expression patterns of seven TGE-derived lincRNAs appear significantly different in these populations of human embryonic cells (Supplemental Fig. S1C).

Based on the results of these analyses, we concluded that the expression profiling of HPAT lincRNAs in VHB reliably distinguish three main populations of human embryonic cells: *HPAT21* ^*(+)*^ *HPAT2* ^*(+)*^ *HPAT15* ^*(+)*^ *& HPAT21* ^*(+)*^ *HPAT2* ^*(+)*^ *HPAT15* ^*(−)*^ cells (*HPAT2pos* cells)*; HPAT21* ^*(+)*^ *HPAT2* ^*(−)*^ *HPAT15* ^*(−)*^ cells (*spHPAT21* cells); and *HPAT21* ^*(+)*^ *HPAT2* ^*(−)*^ *HPAT15* ^*(+)*^ cells (*dpHPAT15* cells) (Table 1; Supplemental Fig. S1).

### 2.2 Analysis of embryonic cells comprising the *HPAT2pos* population of viable human blastocysts

Single-cell expression profiling experiments provide the unique opportunity to characterize the distinct populations of cells based on the relative prevalence within a population of individual cells expressing the particular sets of genetic markers. We thought to utilize this approach, rather than comparisons of mean expression values [17, 24, 25], reasoning that applications of these single cell analysis-enabled metrics would be more informative in distinguishing different populations. This approach enables the formal statistical evaluation of differences in the relative prevalence of individual cells expressing the particular sets of genetic markers within distinct *HPAT* expression-defined populations of human blastocysts. The *HPAT2pos* populations manifest similar gene expression signatures that remain relatively stable during the blastocyst differentiation (Supplemental Figs. S1E & S1F), indicating that these cells do not experience dramatic large-scale changes in gene expression during the transition from the early to late blastocyst stages. Most visible common changes observed in the *HPAT2pos* cells during VHB differentiation distinguishing these cells from other embryonic cells were increase in the numbers of telomerase-positive cells and *HPAT3*/*HPAT5* lincRNA-positive cells (*TERT* ^*(+)*^, *HPAT3* ^*(+)*^, and *HPAT5* ^*(+)*^ cells; marked by the arrows and stars in the Supplemental Figs. S1–S1F). Significantly, the expression of the pluripotency regulatory genes (also defined as the genetic markers of the epiblast-like populations in human blastocysts) appears markedly enriched in the *HPAT2pos* cells compared with the sp*HPAT21* and *dpHPAT15* cells (Supplemental Fig. S1D).

We observed that structures of the *HPAT15* ^*(−)*^ sub-populations among the *HPAT2pos* cells (Table 1) manifest changes of cellular compositions during the transition from early to late blastocyst stages (Supplemental Fig. S1F). These changes appear to reflect the diversification of cellular compositions within early- and late-stage sub-populations, which is characterized by the relative losses of cells expressing the *FN1; CXCR4; EpCAM; BRG1; MAPK1; WNT1; HPAT4* lincRNA transcripts and the relative gains of cells expressing the *HPAT5; HPAT3; EED; THAP11; TET1; LEFTY2; SALL4; MAPK3; NANOG; LATS2; DPPA3; TERT; FGFR2; ID2; CDKN2A* transcripts (Supplemental Fig. S1F). Notably, similar although apparently less profound changes were observed within the early- and late-stage sub-populations of triple-positive embryonic cells assigned to the *HPAT2pos* population (Table 1; Supplemental Fig. S1E), consistent with the hypothesis that collectively the *HPAT2pos* population may comprise of functionally closely-related embryonic cells. Overall, we observe that the functional identities of genes comprising the GES of the *HPAT2pos* population appear highly consistent with the putative biological role of these cells as the ground-state pluripotency precursor cells in human embryos defined as the epiblast-like cells (Supplemental Figs. S1D–S1F).

### 2.3 Analysis of expression profiles of genetic markers of distinct lineages created during human blastocyst differentiation

In contrast to the *HPAT2pos* cells, we observed clear evidence that other *HPAT*'s expression-defined populations appear to display the gene expression changes which were previously identified as genetic elements of distinct differentiation programs during the human blastocyst development (Supplemental Fig. S2). The validity of the proposed lineage assignments to the *HPAT's* expression-defined populations in human blastocyst were assessed and confirmed using single-cell expression profiling data of genes specifically expressed in the trophectoderm (*TROP2, CDX2, TEAD4*); expression profiling of known markers of the pluripotent epiblast (*LIN28A* and *TDGF1*) and known markers of primitive endoderm (*GRB2* and *GATA4*) [27, 28, 29]. These analyses demonstrate that the *HPAT2pos* cells appear enriched for expression of genetic markers of the pluripotent epiblasts; the *dpHPAT15* population resembles the primitive endoderm-like cells, while the *spHPAT21* population resembles cells of the trophectoderm-like lineage.

To further strengthen these conclusions, additional sets of the human blastocyst lineage-specific genetic markers identified in the recent study were analyzed [24]. It has been documented that primitive endoderm (PE) cells express high levels of the *FGFR2* and low levels of the *GATA4* at the early-stage blastocyst differentiation [24]. During the transition from the early to late blastocyst stages, the *FGFR2* expression in the PE cells is markedly diminished, while expression of *GATA4* is concomitantly significantly increased [24]. These observations are highly consistent with previous reports demonstrating that FGFR2 is expressed by PE progenitor cells and activation of the FGF4/FGFR2 signaling axis is an essential factor for PE differentiation in the mouse [27, 29, 30]. FGF4 is released by epiblast cells and binds to FGFR2-expressing PE progenitor cells, resulting in repression of *NANOG* and activation of *GRB2* and *MAPK* genes in PE progenitor cells, which ultimately leads to the induction of the mature PE specific marker GATA4 [31, 32]. Remarkably, the *dpHPAT15* ^*(+)*^ cells, in striking contrast to other *HPAT* expression-defined populations recovered from early and late blastocysts, recapitulated exactly the PE-like patterns of *FGFR2* and *GATA4* expression changes during human blastocyst differentiation (Supplemental Figs. S2A–S2C). These data suggest that the *dpHPAT15* cells resemble the PE-like population of human blastocysts. This conclusion is in a good agreement with the recent observation that *HPAT15* lincRNA appeared to be predominantly expressed in mature PE cells [24].

Expression analysis of markers of trophectoderm (TE) cells demonstrates that the *spHPAT21* cells recovered from the early-stage blastocysts contain only 27% of *CDX2/TROP2/TEAD4*-positive cells (Supplemental Fig. S2B). In contrast, the *CDX2/TROP2/TEAD4*-positive cells became a dominant sub-population (67%) among the *spHPAT21* cells recovered from late-stage blastocysts. This marked enrichment for cells expressing TE-like genetic markers during blastocyst differentiation appears specific to the *spHPAT21* population since it has not been observed in other *HPAT* expression-defined populations recovered from early- and late-stage human blastocysts (Supplemental Figs. S2B & S2C). Collectively, these data indicate that the *spHPAT21* cells resemble the TE-like lineage of human blastocysts.

Based on these considerations we conclude that *HPAT's* expression-defined stratification of embryonic cells isolated from VHB identifies three distinct populations resembling three major embryonic lineages, which were previously identified based on the expression analyses of protein-coding genes conclusively established as the definitive markers of corresponding embryonic lineages. According to our analysis, the *HPAT2pos* cells are enriched for expression of genetic markers of the pluripotent epiblasts (EPI); the *dpHPAT15* cells resemble the PE-like population of human blastocysts, while the *spHPAT21* cells resemble the TE-like lineage of human blastocyst.

### 2.4 Expression patterns of human naïve pluripotency regulatory genes in HPAT expression-defined populations of embryonic cells during human blastocyst differentiation

**Fig. 1:**
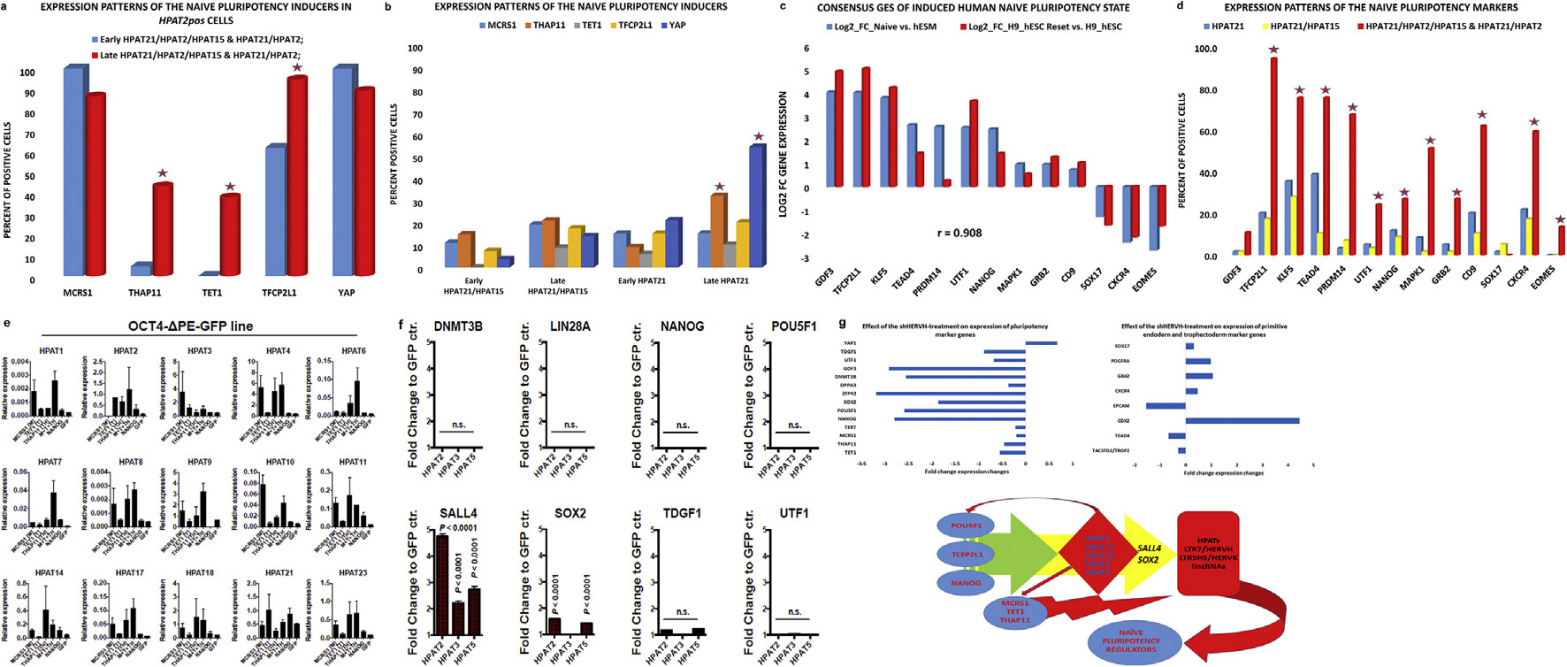
Single-cell analysis of expression patterns of the genetic markers and inducers of the human naïve pluripotency state in HPAT lincRNA expression-defined populations during human blastocyst differentiation. Expression changes of designated genetic inducers (A; B) and markers (C; D) of the human naïve pluripotency state (see text for details) were evaluated in individual cells comprising the corresponding HPAT expression-defined populations that were recovered from the early-stage and late-stage human blastocysts. The statistical significance of the observed expression changes between populations or within a population during blastocyst differentiation were estimated based on comparisons of the numbers of positive and negative cells using two-tailed Fisher's exact test. Stars designate populations harboring the significantly different numbers of cells expressing defined genetic markers (p < 0.05). P values reported in the Supplemental Table S2. Figure (E) reports the results of HPAT lincRNA expression profiling experiments performed on the OCT4-ΔPE-GFP cell line after the induction of the naïve pluripotency state by overexpression of the *MCRS1, TET1* and *THAP11* genes [24]. Figure (F) shows the results of expression profiling analysis of pluripotency regulatory genes in human cells engineered to overexpress the HPAT lincRNAs. Note significant increase of the *SALL4* and *SOX2* expression. Figure (G) illustrates a model of principal molecular events contributing to regulation of the naïve pluripotency induction *in vivo* during transition from *TERT*^*(+)*^ MLME cells to pluripotent epiblast and hESC in human embryos. The first wave of increased expression of 5 most abundant in human blastocysts primate-specific HPAT lincRNAs (highlighted by the green arrow) is triggered by the master pluripotency transcription factors (*POU5F1/OCT4; TCFP2L1/LBP9; NANOG*). The positive feed-back regulatory loop mediated by the activity of HPAT lincRNAs (red arrows) increases expression of master pluripotency transcription factors and naïve pluripotency inducers (*MCRS1; TET1; THAP11*), which triggers the second wave of increased expression of a multitude of transposable elements (TE) – derived lincRNAs (HPATs; LTR7/HERVH; LTR5HS/HERVK; and SVA families). Increased activities of TE-derived lincRNAs make a critical contribution to the embryonic cells' chromatin remodeling by enabling transitions to thermodynamically-stable triple-stranded state of double helix and facilitating targeted delivery of POU5F1/OCT4 & Mediator proteins to thousands of genomic loci. A second wave of the positive feed-back regulatory loop mediated by the activities of TE-derived lincRNAs increases expression of key naïve pluripotency regulators. Top two panels in the Figure (G) show the graphical summary of the effects of shRNA-mediated targeted knockdown of LTR7/HERVH lincRNAs in hESC (15) inducing statistically significant changes in expression of genes encoding naïve pluripotency regulators (top left figure) and genetic markers of TE and PE lineages (top right figure). Only statistically significant gene expression changes are reported (P < 0.05). Experimental evidence supporting the model are reported and discussed in the text.

Several recent studies reported identification of genes that play essential role in induction, reset, and/or maintenance of the naïve pluripotent state in human cells [24, 33, 34, 35]. Forced expression of these genes induce or reset the naïve pluripotent state in human cells whereas their targeted silencing markedly affect the naïve pluripotency phenotypes. We observed that blastocyst cells expressing the genes encoding all these naïve pluripotency inducers are markedly enriched within the *HPAT2pos* population (Fig. 1A and B), particularly among the cells recovered from late-stage blastocysts.

To further test these observations, we identified the consensus GES of the induced naïve pluripotent state based on microarray analyses reported in recent studies, which utilized unique and markedly different protocols of naïve pluripotency induction, reset, and maintenance [33, 34]. This consensus GES of the human induced naïve pluripotent state comprises 13 genes, expression of which was consistently significantly altered in naïve pluripotent cells compared with the primed pluripotent cell cultures resulting in highly correlated profiles of their gene expression changes (Fig. 1C). We thought to utilize this consensus GES to further test the hypothesis that *HPAT2pos* populations of human blastocyst cells may contain putative immediate precursors of naïve pluripotent cells. Significantly, we observed that human embryonic cells comrpising the *HPAT2pos* population are markedly enriched for cells expressing 11 of 13 (85%) of the naïve pluripotency-associated genes compared with *dpHPAT15* and *spHPAT21* cells recovered from human blastocysts (Fig. 1D).

Previous analyses demonstrate that cells expressing genetic markers of the pluripotent epiblasts are significantly enriched within the *HPAT2pos* population (Supplemental Fig. S1). Strikingly, analysis of expression patterns of TE and PE lineage-specific markers revealed that cells expressing multiple genetic markers of both TE and PE lineages appear significantly more prevalent among the *HPAT2pos* population (Supplemental Fig. S2D). These results demonstrate that *HPAT2pos* cells express genetic markers of all three major lineages created during blastocyst differentiation (Supplemental Fig. S2D). Collectively, our observations strongly argue that during human blastocyst differentiation, the unique population of *HPAT2pos* human embryonic cells is created. Individual human embryonic cells comprising the *HPAT2pos* population co-express genetic markers of all three major embryonic lineages. Therefore, the *HPAT2pos* population, in contrast to other cells recovered from human VHB, could be defined as human embryonic cells manifesting the multi-lineage markers' expression phenotype. Characterization of the *HPAT2pos* population as the multi-lineage markers expressing (MLME) cells suggest that these cells may represent putative multi-lineage precursor cells, subsequent specialization and differentiation of which results in the emergence of the trophectoderm, primitive endoderm and pluripotent epiblast lineages.

### 2.5 Activation of the *MTTH/HPAT* lincRNA regulatory axis during human embryogenesis

Previous reports demonstrated that expression of primate-specific retrovirus HERVH-derived lincRNAs [8, 15] and individual *HPAT* lincRNAs [17] in human pluripotent cells is regulated by a combination of transcription factors defined as a core (master) pluripotency regulators. We recently showed that expression of the three protein-coding genes *MCRS1, TET1, THAP11 (MTTH)*, and master pluripotency regulator *NANOG,* was concomitantly highly upregulated in the early epiblast-precursor cells recovered from human blastocysts and demonstrated that forced expression of these three genes (*MTTH*) induces the naïve pluripotency-like phenotype in human cells [24]. Notably, cells expressing all three *MTTH* genes implicated in the naïve pluripotency induction in human cells are significantly enriched among the *HPAT2pos* population recovered from late-stage human blastocysts (Fig. 1A; Supplemental Figs. S1E; S1F).

Based on these observations, we thought to utilize the *MTTH* model of induced human naïve pluripotent cells to determine whether the expression of *HPAT* lincRNAs is affected in human cells that acquired the naïve pluripotency phenotype. Strikingly, we observed (Fig. 1E) that the expression of a majority of *HPAT* lincRNAs was significantly increased in *MTTH*-induced human naïve pluripotent cells which were engineered in accordance with the previously reported protocol [24]. Of note, expression of multiple *HPAT* lincRNAs appears significantly up-regulated in *MCRS1*-overexpressing cells (Fig. 1E), suggesting that the *MCRS1* gene product may regulate the expression of *HPAT* lincRNAs perhaps by relieving the DAXX protein-mediated transcriptional repression via direct protein-protein interaction and sequestering DAXX to the nucleolus [36]. These findings are consistent with the hypothesis that the *MTTH*-induced naïve pluripotent state is associated with marked up-regulation of the *HPAT* lincRNAs expression, which is also increased in distinct populations of cells recovered from human blastocysts.

Active expression of *HPATs* and other HERVH-derived lincRNAs appears necessary for a sustained activity of key pluripotency-regulatory genes since significant reduction of their expression levels were observed in hESC after small hairpin RNA (shRNA) interference-mediated targeting of HERVH-derived transcripts (Fig. 1G; top panel). Notably, siRNA-mediated and/or CRISPR-targeted interference with the expression of specific *MTTH*-regulated HPAT lincRNAs (*HPAT2; HPAT3;* and *HPAT5*) alters the pluripotent cell fate in human preimplantation embryogenesis and during nuclear reprogramming [17]. Therefore, the results of gene silencing experiments indicate that following activation of *HPAT* lincRNA's expression by the core pluripotency transcription factors, the positive feed-back regulatory loop may operate to reinforce the maintenance of the pluripotent state and support a sustained expression of key pluripotency regulators (Fig. 1G).

To further test this hypothesis, we transiently overexpressed *HPAT* lincRNAs in differentiated human cells and measured the expression levels of several key pluripotency regulatory genes (Fig. 1F). We observed consistently increased expression of the *SALL4* and *SOX2* mRNAs in human cells engineered to overexpress the *HPAT2* and *HPAT5* lincRNAs (Fig. 1F). Our experiments indicate that increased expression of *HPAT* lincRNAs may contribute to the sustained activity of both SALL4/NANOG and SOX2/POU5F1 enhancers' networks designed to maintain the stemness regulatory circuitry in human embryonic cells, in part, by increasing the expression levels of the *SALL4* and *SOX2* genes (Fig. 1F and G). This model is further supported by the experimental analyses of the protein-protein interaction networks in ESC indicating that Sall4 and Nanog proteins form a stemness regulatory circuit similar to that of Oct4 and Sox2 proteins to maintain the phenotypic identity of embryonic stem cells (see Discussion).

Collectively, the results of our experiments suggest that *MTTH/HPAT* lincRNAs regulatory axis is established in an *HPAT2pos* population of embryonic cells recovered from VHB (Fig. 1G). To our knowledge, this model for the first time mechanistically links regulatory actions of three protein-coding genes (*MCRS1, TET1, and THAP11*) and primate-specific retrotransposon-derived HPAT lincRNAs during human preimplantation embryogenesis in defined populations of human embryonic cells. Consistent with the documented role of primate-specific retroviruses-derived lincRNAs in pluripotency maintenance in hESC [8, 17], their essential functional roles in induction and maintenance of naïve-like state of hESC [15, 16], in development of human preimplantation embryos [16, 17], and in pluripotency induction during nuclear reprogramming [9, 10, 15, 17], our analysis suggests that specialization and differentiation of the *HPAT2pos* population of human embryonic cells may be associated with the induction of the naïve pluripotency state in human embryos (Fig. 1G).

### 2.6 Single-cell gene expression profiling of *HPAT* lincRNAs in human preimplantation embryos

**Fig. 2:**
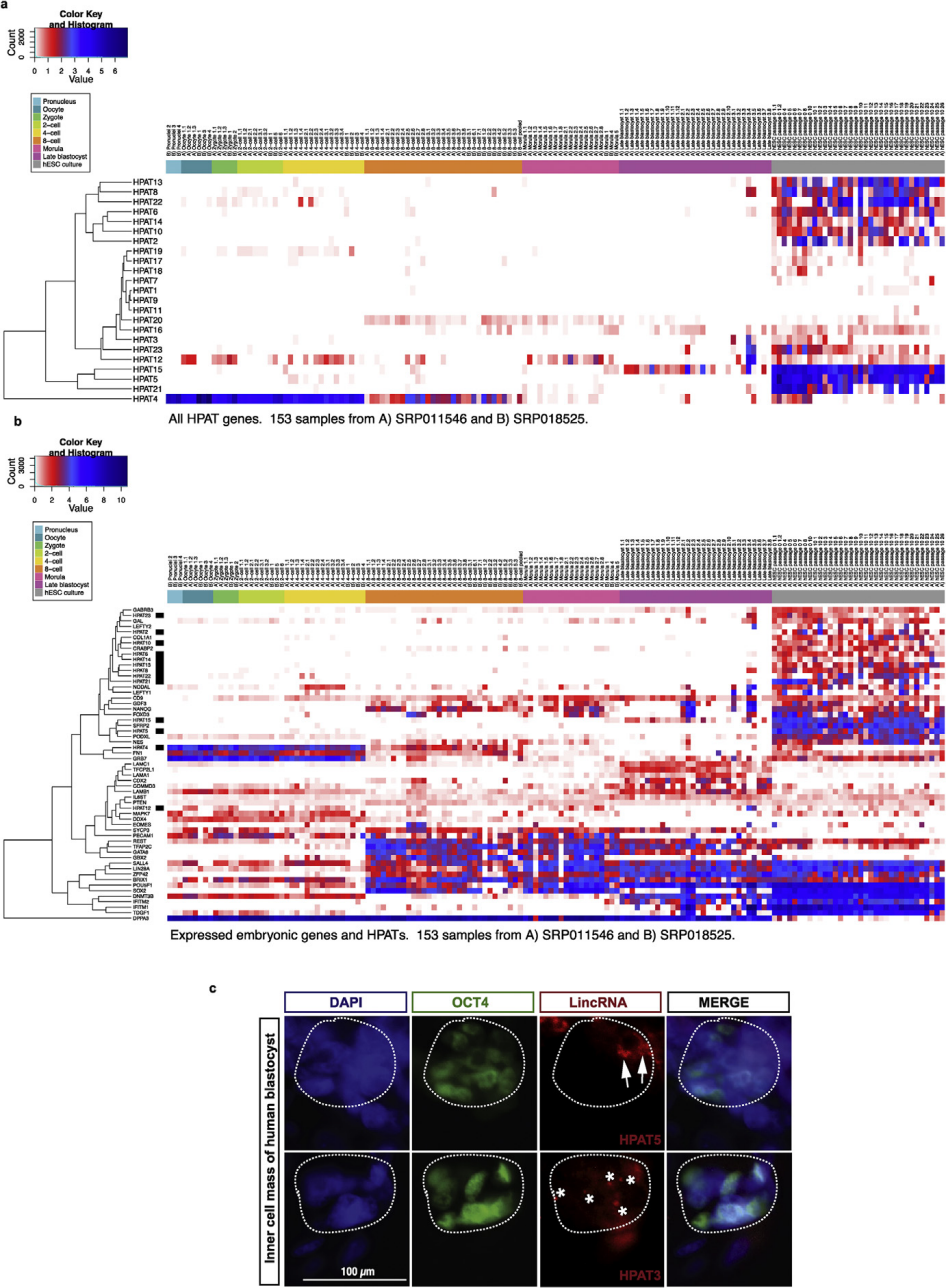
Molecular and gene expression profiling analyses of HPAT lincRNAs during human preimplantation embryo development. A–B, Hierarchical clustering analysis represented in a heat map of HPAT lincRNA expression patterns inferred from a single-cell expression profiling of human embryos from the pronuclei to the late blastocyst stages and hESC stage. Expression of HPAT linRNAs alone (A) or combined with selected embryonic-specific genes (B; C) is shown. Details of methodologies and main experimental outcomes of molecular analyses of *HPAT2*; *HPAT3*; and *HPAT5* lincRNAs were reported elsewhere [17]. Selected results of specific molecular analyses, including RNA FISH images, are presented here to illustrate the common features of the human embryonic cells designated the multi-lineage markers expressing (MLME) cells, which were identified based on the single cell expression analysis of most abundant in human blastocysts primate-specific HPAT lincRNAs. Materials and Methods section provides additional details of the relevant experimental protocols. C, Magnified view of the ICM in a human blastocyst revealing specific staining patterns of *HPAT3* and *HPAT5* lincRNAs and documenting that expression of both HPAT lincRNAs overlap expression of the OCT4 protein. Immunohistochemistry and RNA FISH for OCT4 gene (green) and lincRNAs (red), respectively, in human blastocysts. Sections are counterstained with DAPI (blue). ICMs are circled by dotted white lines. Notably, lincRNA expression signal was specific to the human ICM and was not detectable in the mouse blastocysts (n = 3; data not shown). Images are representative several independent analyses (n = 9; human blastocysts for *HPAT3*, n = 11; human blastocysts for *HPAT5*; n = 2 independent replicate experiments). Scale bar, 100 μm. Stars depict *HPAT3* signal. Arrows depict *HPAT5* signal. Methodology and results of the HPAT RNA FISH experiments were previously reported elsewhere (17).

We next profiled gene expression of all 23 *HPAT* lincRNAs on previously published single-cell RNA-seq datasets (Yan et al. [13] and Xue et al [12]) of human preimplantation embryogenesis (Supplemental Table S1). Individual human embryonic cells were collected during early embryo development, analyzed for expression of 23 HPAT lincRNAs and 89 coding genes and performed hierarchical cluster analysis (Fig. 2A). Moreover, we included a list of embryonic specific genes in the RNA-seq analysis (Fig. 2B; see Supplementary Table 4 and Material and Methods for details). We found that based on their *in vivo* (human preimplantation embryos) and *in vitro* (hESC) gene expression profiles, *HPATs* can be separated into three groups: i) relatively low *in vivo* expression and high *in vitro* expression in hESCs (e.g. *HPAT2*); ii) virtually no *in vivo* expression and very little expression in hESCs (e.g. *HPAT7*); iii) fair to moderately high expressions in late stages during embryo development (morula and late blastocyst) and high expression levels in hESCs (e.g. *HPAT3*). Notably, *HPAT4* revealed an expression pattern that was distinct for each developmental stage during in vivo development and clustered closely with *FN1* and *GRB7* (Fig. 2). While it is highly expressed early on (pronucleus to 4-cell stage), it drops during the 8-cell stage to eventually decline further during later stages (morula and late blastocyst). Collectively and in agreement with recent reports, the results of our analyses are highly consistent with the model predicting numerous diverse functions for primate-specific retroviruses-derived lincRNAs, suggesting that individual HPAT lincRNAs may exert different functions during human preimplantation development, *in vivo* pluripotency induction and maintenance.

It was important to confirm our main observations using independent experimental approaches such as single cell molecular imaging analyses. *HPAT* lincRNAs, like many other HERV-derived non-coding RNA molecules, display a high degree of sequence similarity due to the repetitive nature and common evolutionary origins of their sequences. These features make it difficult to design highly specific probes suitable for the RNA FISH. We were able to overcome these limitations by developing and validating the RNA FISH probes specifically detecting *HPAT3* and *HPAT5* lincRNAs in human embryonic cells [17]. Single cell expression profiling analyses of HPAT lincRNAs demonstrated that both *HPAT3* and *HPAT5* lincRNAs are significantly overexpressed in HPAT2pos population of human blastocyst cells (Table 1; Figs. S1C; S1E; S1F). Therefore, we reasoned that the validated FISH RNA probes for *HPAT3* and *HPAT5* lincRNAs would be useful to validate our findings using independent molecular techniques. To this end, we carried out the immunohistochemistry and RNA FISH for OCT4 protein (green) and HPAT lincRNAs (red), respectively, in human blastocysts (Ref. [17]; Fig. 2C). These experiments revealed specific staining patterns of *HPAT3* and *HPAT5* lincRNAs and demonstrated that expression of both HPAT lincRNAs overlaps expression of OCT4 protein in the ICM isolated from human blastocysts (Ref. [17]; Fig. 2C). Consistent with results of our single cell expression profiling experiments, embryonic cells expressing the *HPAT3* lincRNAs and *HPAT5* lincRNAs are clearly detectable by RNA FISH analysis of the ICM recovered from human embryos (Fig. 2C). Significantly, three HPAT lincRNAs significantly overexpressed in the *HPAT2pos* population of human blastocysts (*HPAT2*; *HPAT3*; and *HPAT5*) were previously implicated in regulation of nuclear reprogramming and pluripotency induction [17]. Collectively, these data are highly consistent with the proposed role of HPAT lincRNAs in human preimplantation embryogenesis.

### 2.7 Single-cell expression profiling of regulatory regions associated with genomic loci encoding *HPAT* lincRNAs during preimplantation development of human embryos

To further validate our observations, we thought to carry-out single-cell expression profiling analyses during human preimplantation development of putative regulatory regions associated with HERVH-derived lincRNAs, including expression of two HERVH-derived lincRNAs (*HPAT2* and *HPAT3*), which appears markedly enriched in *HPAT2pos* blastocyst cells (see above). We reasoned that if the proposed model is valid, the high expression levels of TGE-derived regulatory loci associated with these lincRNAs should be observed in late blastocysts, epiblast cells, and naïve hESCs. To this end, we ascertained the expression profiles of LTR7 and HERVH-loci of *HPAT* lincRNAs in 153 individual human embryonic cells of the Yan et al. [13] and Xue et al [12] validation data sets (Supplemental Table S1). In striking agreement with the model, expression profiles of HERV-derived putative regulatory loci of all five most abundant in human blastocysts *HPAT* lincRNAs (*HPAT21; HPAT15; HPAT5; HPAT2;* and *HPAT3*) in human blastocysts manifest similar patterns of the apparent activation in epiblast cells and reach the maximum expression levels in the naïve hESCs (designated in the Supplemental Figs. S2.2. & S3 as hESCp0 cells). Similar expression patterns of these HERV-derived regulatory loci of *HPAT* lincRNAs during human preimplantation embryo development were clearly discernable during the analyses of both cells' population-based profiles plotted as mean expression values (Supplemental Figs. S3A–S3E) and expression profiles of 153 individual human embryonic cells isolated at various stages of preimplantation embryogenesis (Supplemental Fig. S3F–S3I).

We noted that individual human embryonic cells manifesting low expression of HERV-derived regulatory loci associated with *HPAT* lincRNA are detectable throughout the preimplantation embryo development, including the late blastocyst stage. Intriguingly, some HERV-derived regulatory loci associated with *HPAT* lincRNA manifest clearly distinct expression patterns in naïve hESCs (designated as hESCp0 in the Supplemental Fig. S3) compared with cultured hESCs (designated as hESCp10 in the Supplemental Figs. S2.2. & S3). Individual human embryonic cells with the highest expression of the *HPAT3* lincRNA-associated HERV-derived loci appear at the late blastocyst stage; they represent all naïve hESCp0 cells, and their prevalence is markedly diminished in population of cultured hESCp10 cells. Cells expressing high levels of *HPAT2* lincRNA-associated HERV-derived loci are apparent in hESC populations: they comprise approximately half of the naïve hESCp0 population and continue to persist as a major sub-population in hESCp10 cultures (Supplemental Figs. S3 & S2.2). Taken together, these results provide additional arguments supporting the validity of the proposed model and indicating that the *HPAT2pos* cells manifesting the MLME phenotype may serve as putative precursor cells of naïve-state hESCs.

### 2.8 Promoter analysis of *HPAT* lincRNAs expressed in human blastocysts

We reasoned that similar to other HERVH-derived transcripts previously analyzed in hESC, the expression of the *HPAT* lincRNAs may be regulated by NANOG and POU5F1 transcription factors bound to LTR7 promoter sequences [1, 3, 5, 8, 15]. Specifically, a previous study reported the LTR7 enrichment for OCT4 and NANOG binding sites [15]. To identify high-confidence NANOG-binding sites with putative HPAT transcription regulatory functions, we carried out ChIP-Seq analysis of NANOG occupancy and integrated our ChIP-Seq data with previously published catalogues of NANOG and POU5F1-binding sites in hESC [5], including primate-specific and human-specific sequences bound by NANOG and POU5F1 proteins [3]. Interestingly, our analysis revealed that genomic loci of seven HPAT lincRNAs analyzed in this study (*HPAT*-1; -2; -3; -4; -5; -15; -21) harbor multiple primate-specific NANOG- and POU5F1-binding sites (data not shown).

**Fig. 3:**
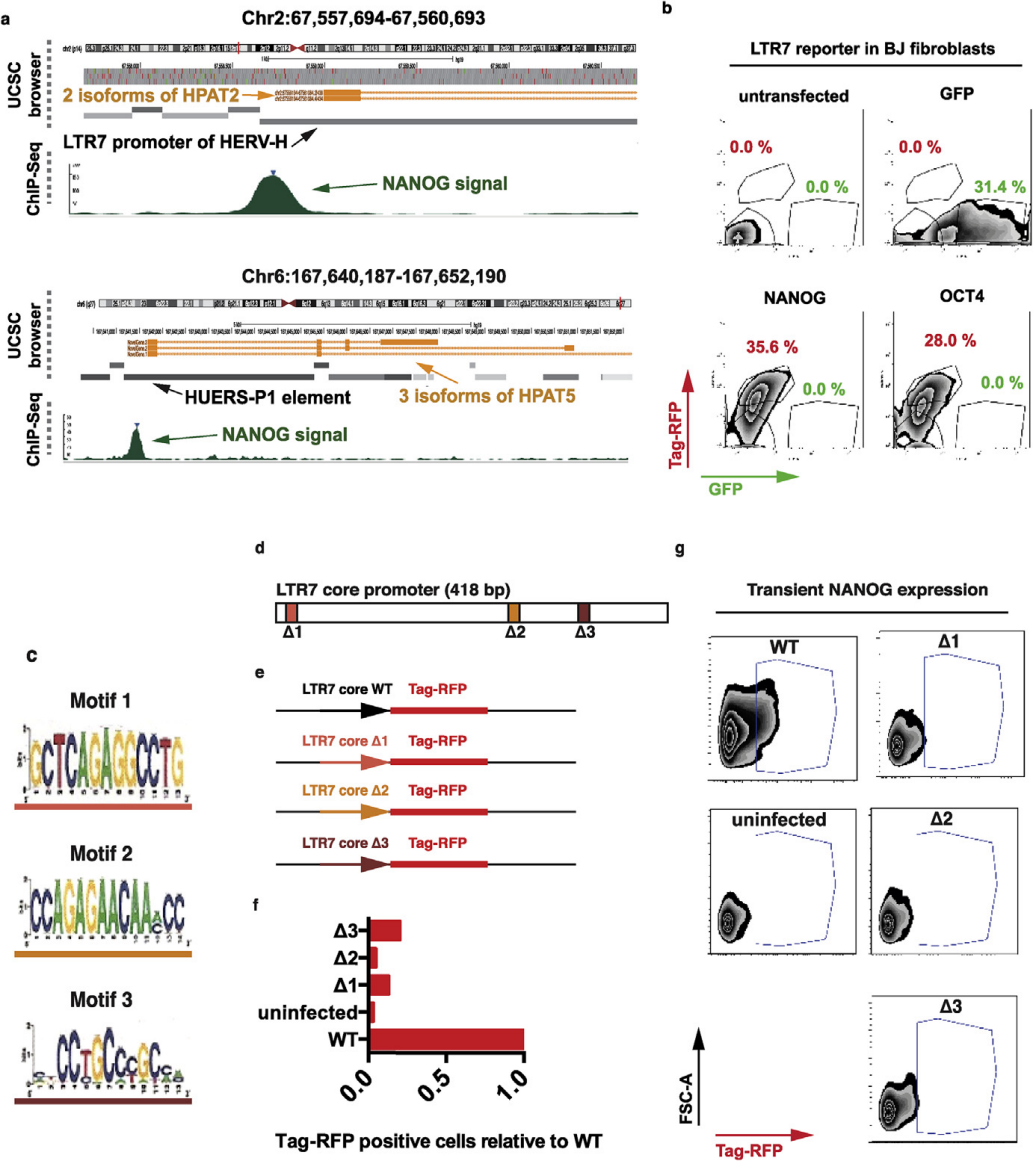
Functional promoter analysis of HPAT transcripts. A, Two snapshots of UCSC browser (with genome location) aligned with NANOG binding region from ChIP-Seq analysis. NANOG signal peaks (green) at putative LTR7-lincRNA promoter of HPAT2 (2 isoforms displayed) and at HUERS-P1 element of HPAT5 (3 isoforms). B, FACS analysis of LTR7 reporter BJ fibroblast line. Reporter line was transiently transfected with mRNA encoding GFP, NANOG or OCT4 following FACS analysis 24 h post-transfection. Tag-RFP and GFP positive cells were gated. C, Predicted motifs of NANOG binding sites in LTR7-promoter regions that are also found on LTR7-reporter cassette. D–E, Deletion mapping of reporter cassettes by construction of lentiviral promoter reporters that drive Tag-RFP. F–G, FACS analysis of LTR7-Mutant reporters in BJ fibroblasts. Reporter lines were transiently transfected with NANOG following FACS analysis 24 h post-transfection. Details of methodologies and main experimental outcomes of molecular analyses of *HPAT2*; *HPAT3*; and *HPAT5* lincRNAs were reported elsewhere [17] and selected specific results, including Chip-Seq data, are described here to illustrate the common molecular features of the most abundant in human blastocyst HPAT licnRNAs expression of which has been utilized to identify the MLME cells in human embryos.

By comparing NANOG DNA occupancy sequences from ChIP-seq experiments, we predicted three putative binding motifs within the core LTR7 reporter cassette and compared them against databases of known motifs (TomTom). However, we failed to identify similar motifs bound by known transcription factors, suggesting that the predicted binding motifs are noncanonical NANOG motifs (Fig. 3). Next, we cloned the sequence of core promoter of LTR7 reporter upstream of Tag-RFP, generated lentiviral reporter constructs and used them to infect BJ fibroblasts. Reporter line was transiently transfected with mRNA encoding GFP, NANOG or OCT4 following FACS analysis 24 h post-transfection. Tag-RFP and GFP positive cells were gated to document the performance of the LTR7 core promoter reporter construct (Fig. 3). To identify the regulatory regions of the LTR7 core promoter, we then deleted the predicted motifs in our LTR7 reporter cassette and generated lentiviral reporter constructs (Fig. 3) that were used to infect BJ fibroblasts. With transient NANOG overexpression, all three deletions significantly impaired Tag-RFP reporter expression compared with the wild-type construct, with Motif 2 revealing the lowest reporter activity (similar to uninfected negative control) (Fig. 3). Thus, ChIP-Seq analysis followed by deletion mapping of the LTR7 core promoter element identified three binding motifs that appear crucial for transcriptional activation triggered by NANOG occupancy of *HPAT* lincRNA promoters. These observations strongly argue that expression of primate-specific HPAT lincRNAs in human blastocysts is regulated by the NANOG binding to the LTR7 promoter sequences, which is highly consistent with their proposed role in human embryonic development and reflected in the mechanistic model shown in Fig. 1G.

### 2.9 Identification and characterization of *TERT*^(+)^ MLME cells recovered from viable human blastocysts

The importance of telomerase reverse transcriptase (*TERT*) expression for embryogenesis and reproductive potential was clearly established [37]. Furthermore, it was reasonable to expect that *TERT* ^*(+)*^ population may display prominently during the transition from early to late blastocyst stages because all examined human fetal tissues of eight-week of gestation express telomerase [38, 39]. We reasoned that the MLME cells containing the putative multi-lineage precursor cell population may be found among populations of human blastocyst cells expressing the *TERT* gene. Importantly, it has been shown that telomerase activity and maintenance of telomere stability conferred increased resistance to programmed cell death in human cells [40]. *HPAT* expression-guided reconstruction of human blastocyst differentiation inferred from a single-cell expression profiling experiments indicates that the population of *TERT* ^(+)^ cells appears to markedly expand within the *HPAT2pos* population during the late blastocyst stage (Table 1): 56% of *HPAT21* ^*(+)*^ *HPAT2* ^*(+)*^ *HPAT15* ^*(+)*^ triple-positive cells express *TERT* gene in the late blastocyst cell population compared to just ∼1% in the early blastocysts (Table 1; Supplemental Figs. S2G & S4). Similarly, 29% of *HPAT21* ^*(+)*^ *HPAT2* ^*(+)*^ *HPAT15* ^*(−)*^ cells and 3.5% of *HPAT21* ^*(+)*^ *HPAT2* ^*(−)*^ *HPAT15* ^*(+)*^ cells express the *TERT* gene among the late blastocyst cells (Table 1; Supplemental Fig. S2G). In striking contrast, all ninety-two *spHPAT21* cells comprising ∼38% of the early and late blastocyst cell populations remain *TERT*-negative (Table 1; Supplemental Figs. S2F & S2G). These results indicate that *HPAT2pos* cells are enriched for the *TERT*-expressing cells and suggest that *TERT*^(+)^ *HPAT2pos* cells may contain a sub-population of the MLME cells. Consistent with this hypothesis, we observed that *TERT*^(+)^ *HPAT2pos* cells are markedly enriched for individual cells co-expressing genetic markers of the pluripotent state and all three embryonic lineages in striking contrast with the *spHPAT21* cells and *dpHPAT15* cells.

In our experiments, the *HPAT2pos* populations contain a vast majority (16 of 18 cells; 89%) of *TERT* ^*(+)*^ cells identified in human blastocysts. Significantly, the functional identities of genes comprising the gene expression signature common for *TERT* ^(+)^ cells within the *HPAT2pos* populations appear highly consistent with the potential biological role of these cells in establishing and maintaining a multi-lineage precursor phenotype in human blastocysts (Supplemental Fig. S4B–S4D). One of the notable features of the cellular identity of the *TERT* ^(+)^ population of human blastocyst cells is a strikingly distinct pattern of representations of cells expressing specific human endogenous retroviruses (HERV)-derived HPAT lincRNAs: a majority (56–100%) of *TERT* ^(+)^ cells express the *HPAT21; HPAT2; HPAT5; HPAT15*; and *HPAT3* lincRNAs, whereas there were no *TERT* ^(+)^ cells expressing *HPAT4; HPAT6; HPAT7; HPAT9; HPAT14; HPAT17; HPAT20;* and *LINC-ROR* lincRNAs (Supplemental Figs. S4B–S4D). These observations are highly consistent with the hypothesis that individual HERV-derived lincRNAs may play different biologically-significant roles during the embryonic development [17] and support the concept that *HPAT2pos* populations of human blastocysts contain the *TERT*^(+)^ MLME cells.

### 2.10 Analysis of telomerase expression dynamics in human preimplantation embryos

We carried out a series of follow-up analyses to identify human embryonic cells resembling the putative MLME cells among individual cells and distinct lineages which were identified and characterized in a recently reported independent data set of single cell expression profiles of 1,529 embryonic cells recovered from 88 human embryos at different stages of preimplantation embryonic development [25]. To this end, we evaluated *TERT* mRNA expression patterns in each of the 1,529 individual human embryonic cells that were recovered at different stages of preimplantation embryogenesis and assigned to distinct lineages based on their gene expression signatures (Supplemental Figs. S5 and S6). These analyses demonstrate that *TERT* ^(+)^ cells could be observed among all lineages and are readily detectable as early as at E3-E4 stages followed by the expansion during the E5 stage coincidently with blastocyst formation (Supplemental Figs. S5 and S6). We found that E5 pre-lineage cells manifest significantly increased levels of *TERT* mRNA expression compared to EPI, PE, and TE lineages (Supplemental Fig. S6). Interestingly, the second peak of *TERT* activity was observed at the E6 in all three major lineages created during human preimplantation embryonic development. We observed that the E5.early cell population has the highest level of *TERT* mRNA expression and contains the largest percentage of *TERT* ^(+)^ cells compared to other stages of human preimplantation embryonic development (Supplemental Fig. S6). Therefore, based on the results of analyses of *TERT* ^(+)^ cells in human preimplantation embryos we built a quantitative model of *TERT* ^(+)^ cells' dynamics in an individual human embryo demonstrating that:

i) *TERT* ^(+)^ cells pervade all lineages and are readily detectable throughout the E3-E7 stages of embryonic development (Supplemental Figs. S5 and S6);
ii) E5 pre-lineage population expresses the highest level of *TERT* mRNA compared to E5.EPI, E5.PE, and E5.TE lineages;
iii) E5.early cells appear most enriched for *TERT*^(+)^ cells and express the highest level of telomerase mRNA (Supplemental Fig. S6).

**Fig. 4:**
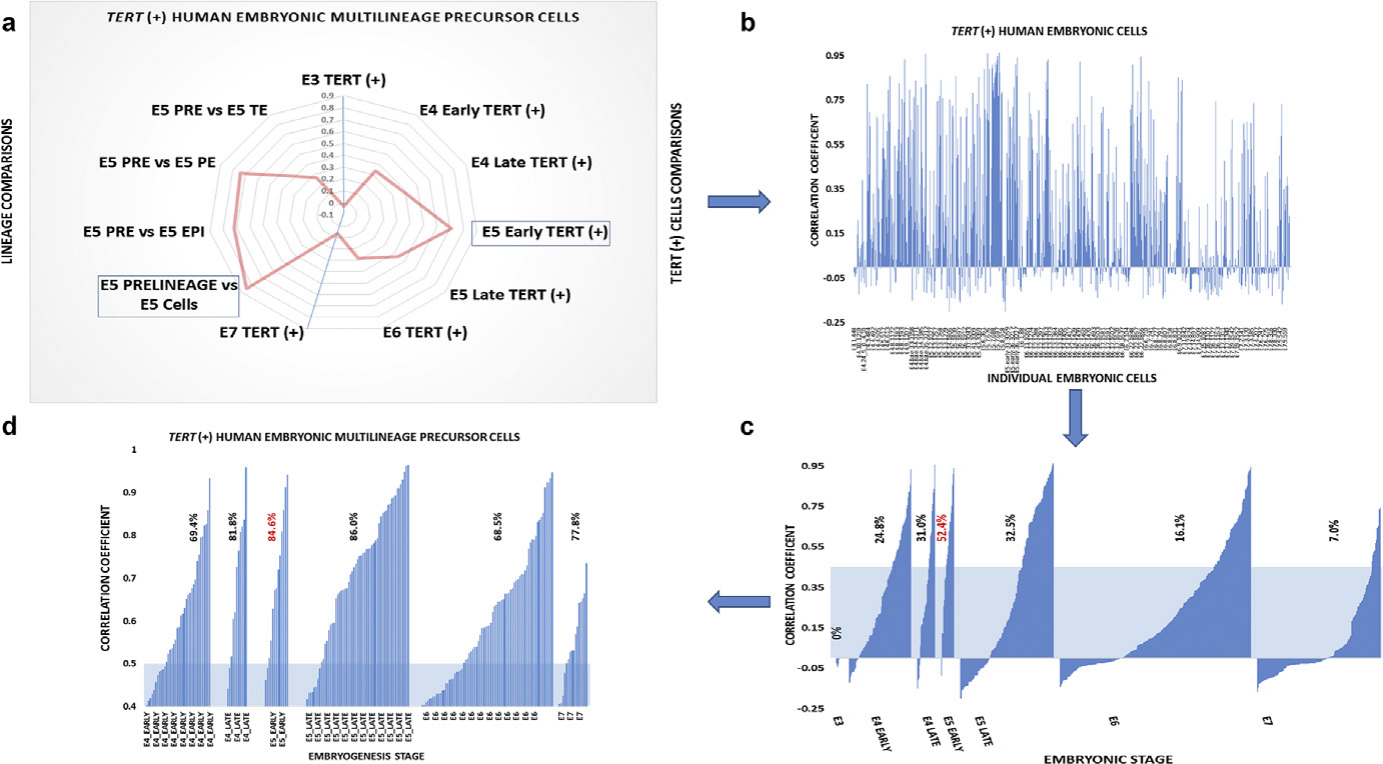
Identification of *TERT*^(+)^ cells resembling the MLME cells' population harboring putative immortal multi-lineage precursor cells (iMPCs) in independent validation sets of 1,529 individual human embryonic cells recovered from 88 human embryos at distinct stages of preimplantation human embryonic development. Forty-six gene expression signature of the MLME cells (putative iMPC population) identified in the single-cell gene expression profiling experiments of 241 human blastocyst cells recovered from 32 human embryos (discovery data set; Supplemental Figs. S1–S5; Supplemental Note 1) was utilized to identify the MLME-resembling cells among the 1,529 individual embryonic cells recovered from 88 human embryos at distinct stages of preimplantation embryonic development (validation data set 1; Supplemental Table S1 and ref. 34). (A) A graphical summary of the population-based correlation score analyses performed on distinct lineages (lineage comparisons scores on the left) and on telomerase-positive cells of distinct embryonic stages [TERT (+) cells comparisons on the right]. Note that E5.Pre-lineage cells (left) and E5.Early *TERT*^*(+)*^ cells (right) manifest the most significant enrichment for the MLME-like cells in the validation data set 1. Left box highlights correlation patterns of the 46-gene MLME signature defined by gene expression ratios of *TERT*^*(+)*^ cells versus EPI cells (discovery data set) and E5.Pre-lineage cells versus E5.EPI cells (validation data set 1). High positive values of the correlation score indicate the resemblance to the MLME/iMPC gene expression profile. Similarly, correlation scores were calculated for the 46-gene MLME signature defined by the gene expression ratios of *TERT*^*(+)*^ cells versus EPI/PE/TE cells (discovery data set) and E5.Pre-lineage cells versus E5.EPI/PE/TE cells (validation data set 1); for the 46-gene MLME signature defined by the gene expression ratios of *TERT*^(+)^ cells versus EPI/PE/TE cells (discovery data set) and E5.Early *TERT*^*(+)*^ cells versus E5 cells (validation data set 1); and for the forty-six gene MLME signature defined by the gene expression ratios of *TERT*^*(+)*^ cells versus EPI/PE/TE cells (discovery data set) and E5.Early *TERT*^*(+)*^ cells versus E5 cells (validation data set 1). Finally, correlation score analysis of the 46-gene MLME signature defined by the gene expression ratios of the *TERT*^*(+)*^ cells versus EPI/PE/TE cells (discovery data set) and E3. *TERT*^*(+)*^ cells versus E3 cells (validation data set 1). Note that in this instance no significant correlation was observed. (B) Correlation score patterns of the 46-gene MLME signature defined by the gene expression ratios of the *TERT*^*(+)*^ cells versus EPI cells (discovery data set), which were assessed in 819 individual *TERT*^*(+)*^ cells identified in the validation data set 1. (C) Correlation score patterns of the 46-gene iMPC signature defined by the gene expression ratios of the *TERT*^*(+)*^ cells versus EPI cells (discovery data set) which were assessed in 819 individual *TERT*^*(+)*^ cells identified in the validation data set 1. Cells were sorted in the ascending order of correlation scores within corresponding developmental stages and all 819 cells were placed in the ascending order of embryonic development stages [from E3 on the left to E7 on the right]. The shaded area highlights a cut-off value of correlation scores chosen for identification of the MLME-resembling cells. Percent's of cells having the correlation score values above the threshold are indicated for corresponding embryonic stages. (D) Panel D shows the zoom-in view of the single-cell analysis of a subset of cells of the validation data set 1, which were segregated at a cut-off value of correlation score 0.4. Percentage values are the percent of individual cells within a population with the correlation score >0.5. Using this approach, a total of 158 *TERT*^*(+)*^ human embryonic cells manifesting correlation scores >0.5 were identified in the validation set 1.

Petropoulos et al. [25] reported that cells during human embryonic development acquire an intermediate state of co-expression of lineage-specific genes, followed by a concurrent establishment of the trophectoderm (TE), epiblast (EPI), and primitive endoderm (PE) lineages coincidently with blastocyst formation. They concluded that segregation of all three lineages in human embryos occurs simultaneously and coincides with blastocyst formation at E5. During the early E5 stage, embryonic cells that had activated TE genes also maintain the expression of EPI genes, consistent with the hypothesis of an intermediate stage of co-expression of genetic markers of multiple lineages [25]. We reasoned that cells within the cell population co-expressing genetic markers of distinct lineages and designated the E5 pre-lineage may be phenotypically similar to identified herein MLME cells. Consistent with this line of reasoning, we observed that the E5 pre-lineage population harbors a population of *TERT* ^*(+)*^ cells resembling the MLME cells (Fig. 4A).

To detect cells resembling the MLME cells among the 1,529 individual embryonic cells reported in the Petropolous et al. study [25], we carried out correlation analyses of expression profiles of 46 genes distinguishing MLME cells from other lineages (Fig. 4; Supplemental Fig. S4; Supplemental Table S3). In these experiments, the expression profiles of 46 genes comprising candidate cell identity genes of MLME cells were identified in individual cells and different lineages in the Petropolous et al. data set [25] and compared to the MLME expression profile identified in this study. Cells were defined as MLME-resembling if their correlation coefficient of gene expression profiles exceeds 0.5. These analyses revealed the consistent presence of MLME-resembling cells among human embryonic cells characterized in the Petropolous et al. data set (Fig. 4). Importantly, the MLME-resembling cells are represented most prominently among E5 pre-lineage cells and *TERT* ^(+)^ E5.early cells of the Petropolous et al. study (Fig. 4). In striking contrast, the population of *TERT* ^(+)^ E3 cells does not appear to contain the MLME-like cells (Fig. 4). This conclusion remains valid when observations were made employing the population-based approach by comparing either the lineage-defined or embryonic stage-defined populations (Fig. 4).

### 2.11 Single cell analysis of human preimplantation embryos revealed a timeline of emergence and sustained presence of MLME cells and identified common gene expression signatures in independently-defined populations of *TERT*^*(+)*^ MLME cells

We thought to extend the population-based observations identifying the MLME-resembling cells by performing the analyses of all telomerase-positive individual human embryonic cells of the Petropolous et al study. To this end, we selected all 819 *TERT* ^(+)^ cells that are reported in the Petropolous et al. data set and compared their individual gene expression profiles to the 46-gene MLME expression signature (Fig. 4B–D). Consistently with the conclusions made based on the analyses of the embryonic lineage-defined and embryonic stage-defined populations (Fig. 4A), there were no MLME-like individual cells detectable at the E3 stage (Fig. 4C). We observed that individual MLME-resembling cells emerged among human embryonic cells at the E4.early stage and persisted throughout E4-E7 stages of human embryonic development (Fig. 4C and D). The largest percentage of the MLME-resembling cells was observed among the *TERT* ^(+)^ E5.early cells (Fig. 4C and D). Using this strategy, we identified *158 TERT* ^*(+)*^ human embryonic cells in the Petropolous et al. data set, which recapitulate the 46-gene expression profile of the MLME cells (Fig. 4C & D). Furthermore, our analyses identify the E5 pre-lineage cells and the E5.early *TERT* ^*(+)*^ cells as populations of human embryonic cells that appears most enriched for the MLME-like cells. Therefore, it seems reasonable to conclude that these analyses demonstrate a consistent presence of the MLME-resembling cells in human preimplantation embryos.

**Fig. 5:**
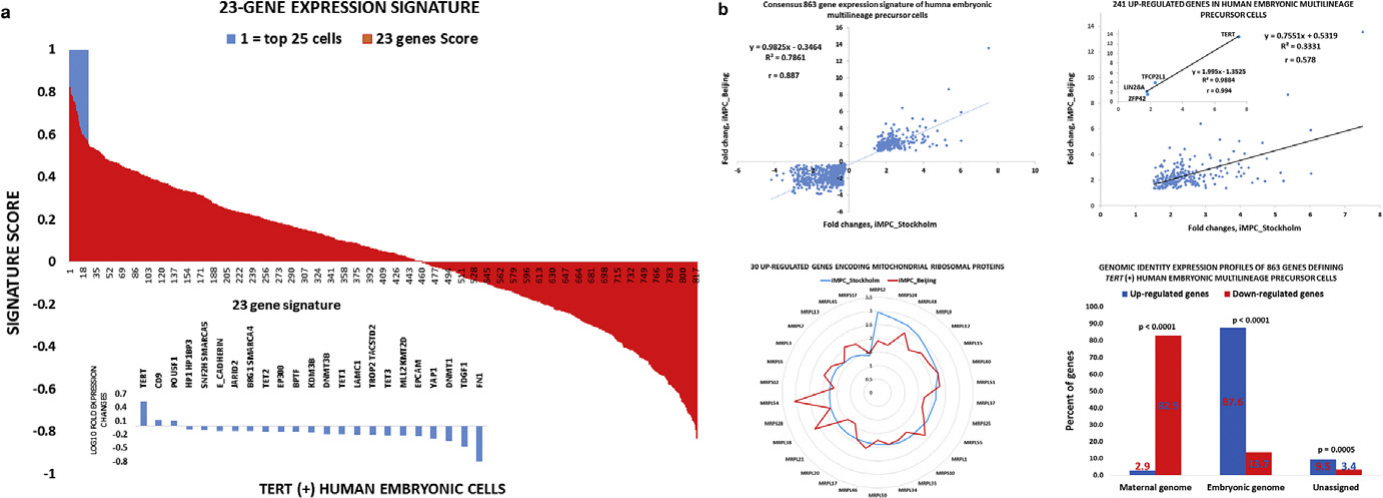
Characterization of the MLME cells identified in the independent validation data sets of human preimplantation embryogenesis. (A) Expression profiles of the twenty-three gene signature in 819 individual *TERT*^*(+)*^ cells identified in the validation data set 1. The 23-gene signature comprises a sub-set of genes of the 46-gene signatures that manifest significantly different expression levels in 158 *TERT*^*(+)*^ cells (r > 0.5) compared to 661 *TERT*^*(+)*^ cells (r < 0.5) in the validation data set 1. Using the gene expression profile of the 23-gene signature, correlation scores were calculated for 819 *TERT*^*(+)*^ cells of the validation data set 1 (red colored bars). Blue bars designate top-scoring twenty-five individual *TERT*^*(+)*^ cells manifesting correlation scores r > 0.55. The MLME phenotype of these cells was validated using the independent sets of human embryonic lineage-specific genetic markers. (B) Consensus 863-gene expression signature of the *TERT*^*(+)*^ human embryonic multi-lineage precursor cells manifesting the MLME phenotype. A total of 863 genes (241 up-regulated and 622 down-regulated genes) manifesting highly concordant gene expression profiles (r = 0.887; top left figure) in the *TERT*^*(+)*^ human embryonic multi-lineage precursor cells were identified using two independent validation data sets (Petropolous et al. validation data set 1 and Yan et al. validation data set 2). Concordant expression profiles of 241 up-regulated genes of the consensus gene expression signature of iMPC are shown in the top right figure. The inset highlights concordant expression patterns of four key pluripotency regulatory genes (*TERT; TCFP2L1; LIN28A;* and *ZFP42*). Bottom left figure shows expression patterns of 30 genes encoding mitochondrial ribosomal proteins, expression of which is significantly increased in both populations of *TERT*^(+)^ human embryonic MLME cells identified in two independent validation data sets. Bottom right figure documents distinct genomic origins of 241 up-regulated (blue bars) and 622 down-regulated (red bars) genes comprising the consensus gene expression signature of the *TERT*^(+)^ human embryonic MLME cells. Note that transcription of 87.6% up-regulated genes originates from the embryonic genome, while transcriptional origins of 82.9% down-regulated genes were assigned to the maternal genome. The assignments of genomic origins of 863 genes were performed based on single cell gene expression profiling of twenty-six human embryonic cells reported in the validation data set 4 (Supplemental Table S1).

To further test the validity of our hypothesis that MLME cells are created during human preimplantation embryogenesis, we carried-out several additional follow-up analytical experiments. We performed the formal statistical analysis to determine whether the expression levels of the 46 genes comprising the MLME gene expression signature are statistically different in 158 *TERT* ^*(+)*^ MLME-like cells compared with the remaining 661 *TERT* ^*(+)*^ cells of the Petropolous et al. validation data set that are not resembling the MLME cells. This analysis identifies twenty three genes (50% of 46 genes) manifesting statistically distinct expression levels in *TERT* ^*(+)*^ MLME-like cells (Fig. 5A). We thought to utilize this 23-gene expression signature for more stringent definition of the MLME-like cells in independent validation data sets of single-cell expression profiling of human preimplantation embryos [13, 25]. We reasoned that the cut-off number for selection of top-scoring MLME-resembling *TERT* ^*(+)*^ cells should be sufficient to estimate that at least one MLME-like cell would emerge in individual human embryos at any embryogenesis stage prior to the lineage segregation. Therefore, in subsequent validation analyses we thought to restrict the selection of putative MLME-like cells in a data set to the minimum number of *TERT* ^*(+)*^ cells displaying the highest 23-gene signature correlation scores, provided the above criterion is satisfied. Using the expression profile of 23-gene signature (Fig. 5A; Supplemental Table S3) as a tool for more stringent definition of the MLME-resembling cells, we identified top-scoring *TERT* ^*(+)*^ MLME-like embryonic cells in two independent validation data sets comprising 1,529 and 124 individual human embryonic cells and designated as validation data set 1 and 2, respectively (Supplemental Table S1; refs. 25 & 13).

Next, the similarity patterns of gene expression signatures (GES) defining the MLME cells (putative immortal multi-lineage precursor cells) were ascertained by comparisons of three populations of MLME cells which were independently identified in our discovery data set (Supplemental Fig. S7F; top two figures), in the Petropolous et al. [25] validation data set 1 (Supplemental Fig. S7F; bottom left figure), and in the Yan et al. [13] validation data set 2 (Supplemental Fig. S7F; bottom right figure). The genes comprising the MLME cells' GES were identified in the single cell expression profiling experiments of VHB, grouped into relevant functional categories and placed in the same orders in all four images shown in the Supplemental Fig. S7F. The top two figures show the MLME GES profiles of the *HPAT2pos* cells (left) and *TERT* ^*(+)*^ cells (right) identified in our discovery data set. The bottom left figure shows the MLME GES profile of the top-scoring *TERT* ^*(+)*^ cells identified in the Petropolous et al. [25] validation data set 1 (Supplemental Table S1) based on the correlation scores of the 23-gene signature. The bottom right figure shows the MLME gene expression profile of the top-scoring *TERT* ^*(+)*^ cells identified in the Yan et al. [13] validation data set 2 (Supplemental Table S1) based on the correlation scores of the 23-gene signature. The numerical values corresponding to each gene indicate the percentage of positive cells which were identified using defined expression thresholds within the corresponding populations and plotted to observe the images of signature patterns (Supplemental Fig. S7F). Based on these analyses, we concluded that gene expression profiles' visualization experiments demonstrated clearly discernable similarity patterns of GES in independently-defined populations of *TERT* ^*(+)*^ MLME cells isolated from human preimplantation embryos.

### 2.12 Genome-wide screens of single-cell expression profiles of embryonic cells identify consensus gene expression signatures and estimate creation timelines of MLME cells in human and mouse embryos

Identification of the MLME-like cells in independent validation data sets of human preimplantation embryogenesis afforded the opportunity to utilize genome-wide gene expression data of individual embryonic MLME-like cells for definition of a consensus gene expression signature of *TERT* ^*(+)*^ MLME cells in human embryos. We accomplished this task by identifying differentially expressed genes distinguishing the MLME-like cells in the Petropolous et al. [25] and Yan et al. [13] data sets and selecting the genes manifesting the concordant statistically significant up- and down-regulation in both data sets. This approach identifies the consensus 863-gene expression signature of the *TERT* ^*(+)*^ MLME cells of human embryos comprising 241 up-regulated and 622 down-regulated genes (Fig. 5B). Notably, the genomic origin of a majority (82.9%) of down-regulated genes of the consensus *TERT* ^*(+)*^ MLME cells' expression signature could be traced to the maternal genome (Fig. 5B; red bars in the bottom right image), suggesting that the efficient transcriptional repression of maternally-expressed genes represents one of the key features of the human embryonic MLME population. In striking contrast, the genomic origin of a vast majority (87.6%) of up-regulated genes could be traced to the embryonic genome (Fig. 5B; blue bars in the bottom right image), indicating that genes transcriptionally active in the embryonic genome make a significant contribution to the creation of the *TERT* ^*(+)*^ MLME cells in human embryos.

We thought to employ the 23-gene expression signature of *TERT* ^*(+)*^ MLME-like cells of human embryos to identify the putative MLME-like cells among the mouse embryonic cells that were isolated during various stages of the mouse preimplantation embryogenesis [41]. Analysis of gene expression profiles of 259 mouse embryonic cells (mouse embryo validation data set; Supplemental Table S1) identifies top-scoring *TERT* ^*(+)*^ MLME-like mouse embryonic cells comprising ∼5% of mouse embryonic cells. Using genome-wide statistical screens of single-cell gene expression profiling of embryonic cells, we were able to identify individual MLME-like cells in a data set which were assigned to the particular stages of human and mouse embryonic development. Using these data and reported numbers of analyzed embryonic cells assigned to the corresponding embryogenesis stage in a data set, we estimated the likelihood of emergence and expected creation timelines of the *TERT* ^*(+)*^ MLME cells in individual human and mouse embryos (Supplemental Fig. S7H). We observed a sustained presence of the *TERT* ^*(+)*^ MLME-resembling cells during the E4-E6 stages of human preimplantation embryonic development in the Petropolous et al. [25] validation data set 1 (Supplemental Fig. S7H; top two images). In the Yan et al. [13] validation dataset 2, the creation timelines of the *TERT* ^*(+)*^ MLME cells in human embryos were defined as the morulae and blastocyst stages (Supplemental Fig. S7H; the bottom left image). In contrast to human embryos, the *TERT* ^*(+)*^ MLME-like cells appear to emerge as early as the 4-cell and 8-cell stages of the mouse embryonic development (Supplemental Fig. S7H; the bottom right image). Therefore, the MLME-like cells seem to emerge earlier during mouse preimplantation embryogenesis compared with human embryos (Supplemental Fig. S7H; the bottom right image). These observations are consistent with the model of embryonic development that was proposed based on the mouse preimplantation embryogenesis studies, where the TE and ICM fate is initiated in a positional and cell polarization-dependent manner within the morulae [42] and is followed by a progressive maturation of the EPI and PE lineages in the blastocyst [43]. Consistent with this line of reasoning, human morulae compaction occurs at the 16-cell stage [44] whereas mouse morulae compaction takes place at the 8-cell stage, explaining a delay in lineage segregation observed in the human embryos compared with the mouse [25].

Gene ontology analysis of the consensus GES of the *TERT* ^*(+)*^ MLME cells strongly supports definition of these cells as putative immature multi-lineage precursors lacking gene expression features of specialized cells (Supplemental Fig. S8). Consistent with the proposed role of *HPAT* lincRNAs in the biogenesis of the MLME cells, the most statistically enriched biological processes among genes up-regulated in the MLME cells are non-coding RNA metabolism and non-coding RNA processing (Supplemental Fig. S8). Other significantly enriched biological processes among genes up-regulated in the MLME cells are translation, tRNA metabolic processes, establishment of protein localization, protein transports, ribonucleoprotein complex biogenesis (Supplemental Fig. S8). Therefore, gene ontology analysis appears to indicate that gene expression networks of the MLME cells are devoted to establishing key fundamental building blocks of an essential intracellular infrastructure that is required for proper biological functions of all cells. We noted that genes encoding mitochondrial ribosomal proteins represent one of the largest families of transcripts up-regulated in the MLME cells (Fig. 5B; bottom left image). Since mRNAs for all 13 proteins that are necessary to assemble mitochondria are encoded by mitochondrial DNA and translated on mitochondrial ribosomes, these data suggest that activation of genomic networks supporting the energy-producing infrastructure in a cell is one of the key features of the MLME cells. Furthermore, mitochondrial ribosomal proteins were implicated in ribosomal RNA-mediated protein folding by facilitating the release of proteins in a folding competent state [45].

In striking contrast, biological processes that are significantly enriched among genes down-regulated in the MLME cells appear linked with cellular differentiation and fine specialization of differentiated cells (Supplemental Fig. S8). They were defined by gene ontology analysis as processes of cell adhesion, biological adhesion, cell motion, cell motility, localization of cells, regulation of response to external stimulus, cell migration, response to wounding, and gland development. Collectively, these results are highly consistent with the hypothesis that the MLME cells represent a transitory cell type during embryogenesis that differentiate into trophectoderm, primitive endoderm and pluripotent epiblast lineages.

The results of extensive validation analyses using single cell expression profiles of the 1,708 individual embryonic cells recovered from more than 100 human embryos and 259 individual mouse embryonic cells at different stages of preimplantation embryonic development appear to support the validity of the model that during embryonic development the *TERT* ^*(+)*^ MLME population is created. Based on the previously published studies [3, 8, 15, 17, 24, 46] and experimental evidence reported in this contribution, we propose a model depicting molecular and cellular events contributing to regulation of human embryogenesis *in vivo* during the transition from the *TERT* ^*(+)*^ MLME phenotype to pluripotent epiblast cells and hESCs (Fig. 1G). The key elements of this two-wave embryogenesis progression model highlight the putative central regulatory role of the primate-specific retrotransposon-derived lincRNAs and the *MTTH/HPAT* lincRNAs/*SALL4/SOX2* axis in promoting a genome-wide transition to the embryonic chromatin state in epiblasts [24], thus facilitating the induction of the ground-state naïve pluripotency phenotype (Fig. 1G).

### 2.13 Validation of the association of the MLME and telomerase expression phenotypes in human embryonic cells using distinct analytical strategies

During the follow-up validation analyses using the external validation data sets, we utilized gene expression signatures of the MLME cells identified in our discovery data set of 241 embryonic cells isolated from VHB for the molecular definition of the MLME-resembling cells within heterogeneous populations of human embryos (see above). It was important to confirm the molecular identity of embryonic cells manifesting the MLME phenotype and its association with telomerase expression during preimplantation embryogenesis using the alternative analytical metrics. Therefore, in the next set of the digital lineage tracing experiments, we set out to validate the association of the MLME phenotype and telomerase expression in embryonic cells isolated from human and mouse embryos using different analytical metrics for definition of the MLME phenotype.

Detailed results of these analyses, including descriptions of the set of lineage-specific genetic markers of mouse embryos and the panel of genetic markers of human embryonic and extraembryonic lineages analyzed in these experiments, are listed in the Supplemental Tables S4.1–S4.4. Importantly, the extended sets of lineage-specific genetic markers employed in these experiments were not utilized for identification of the MLME cells implemented in this study so far. These lineage-specific genetic markers were originally defined in independent studies and reported elsewhere [47, 48]. Significantly, methodologically similar analyses were carried out on 1,529 human and 259 mouse embryonic cells, however, the species-specific genetic panels of human and mouse lineage-specific markers were substantially distinct and resulted in largely non-overlapping multi-lineage markers' expression profiles of the MLME cells identified in the mouse and human embryonic cells (Supplemental Tables S4.1–S4.4). These results indicate that the MLME cells identified in human and mouse embryos are likely to represent transcriptionally and, perhaps, biologically distinct populations.

Initial digital lineage-specific genetic markers' expression tracing experiments were performed on telomerase positive embryonic cells. To this end, a total of 819 *TERTpos* individual human embryonic cells were analyzed (Table 2). Each individual embryonic cell was evaluated and designated as the MLME cell if it expressed at least four genetic markers of each of the three major embryonic lineages (epiblast, EPI; throphectoderm, TE; and primitive endoderm, PE) including all three (*NANOG; POU5F1; SOX2*) pluripotent state master regulators. Thus, in these analyses the MLME phenotype in individual human embryonic cells was defined as co-expression of at least four markers of each of the three major embryonic lineages (EPI; TE; and PE), including three main master pluripotency transcription factors. The relative ranks of cells in the data set were defined by the value of the 23-gene signature score (Table 2). In these analyses we utilized a panel of 58 genetic markers of human embryonic lineages, including the extended set of genetic markers of human placenta and TE to assess the expression of extraembryonic markers, including 13 transcription factors comprising a core TE regulatory circuitry [47]. The expression levels of 58 genetic markers of human embryonic lineages were considered individually in a particular single cell by comparing the expression values of the markers in a given cell and the median expression value of the marker in the population of single cells of human embryos. The marker was considered expressed when the expression value in a cell exceeded the median expression value. To ascertain the statistical significance of the findings, p values were estimated using the hypergeometric distribution test (Table 2).

**Table 2:** Prevalence of single cells manifesting MLME phenotypes within populations of *TERTpos* human embryonic cells.

Rank of human embryonic cells	Median 23-gene signature score	Cut-off scores	Number of cells	MLME phenotype, n	Non-MLME, n	MLME phenotype, %	P value
Cells # 1–25	0.638	>0.550	25	17	8	68.0	3.961E-07
Cells # 26–41	0.531	0.510–0.550	16	7	9	43.8	0.025942
Cells # 42–82	0.464	0.429–0.510	41	12	29	29.3	0.0666513
Cells # 26–82	0.474	0.429–0.550	57	19	38	33.3	0.0110939
Top 3% of cells (n = 25)	0.638	>0.550	25	17	8	68.0	3.961E-07
Top 5% of cells (n = 41)	0.580	>0.510	41	24	17	58.5	8.95E-08
Top 10% of cells (n = 82)	0.510	>0.429	82	36	46	43.9	7.259E-07
Bottom 90% of cells (n = 737)	0.005	-0.832 to 0.428	737	139	598	18.9	7.259E-07
All *TERTpos* cells (n = 819)	0.047	-0.832 to 0.822	819	175	644	21.4	

A total of 819 *TERTpos* individual human embryonic cells were analyzed and each single cell was identified as the putative immortal multi-lineage markers expressing (MLME) cell if it expressed at least 4 genetic markers of each of the 3 major lineages (epiblast, EPI; throphectoderm, TE; and primitive endoderm, PE) including all three (*NANOG; POU5F1; SOX2*) pluripotent state master regulators. Thus, the MLME phenotype in individual human embryonic cells was defined as co-expression of at least four markers of each of the three major embryonic lineages (EPI; TE; and PE) and three main master pluripotency transcription factors. The relative ranks of individual cells were defined by the value of the 23-gene signature score. The expression levels of 58 genetic markers of human embryonic lineages were considered individually in a particular single cell by comparing the expression values of the markers in a given cell and the median expression value of the marker in the population of single cells of human embryos. The marker was considered expressed when the expression value in a cell exceeded the median expression value. The set of genetic markers of human embryonic and extraembryonic lineages analyzed in these experiments is listed in the Supplemental Table S4 and was originally reported elsewhere [47, 48]. P values were estimated using the hypergeometric distribution test.

The evaluation of 819 *TERT*^*(+)*^ human embryonic cells revealed that cells manifesting the MLME phenotype are significantly more prevalent among telomerase-positive human embryonic cells having higher values of the 23-gene expression signature scores (Table 2). Using the same analytical protocol, the prevalence of the MLME phenotype was analyzed in all human and mouse embryonic cells regardless the *TERT* expression status (1,519 individual human embryonic cells and 259 individual mouse embryonic cells). The results of these analyses reported in Table 3 conclusively demonstrate that cells manifesting the MLME phenotype are significantly more prevalent among *TERT*^*(+)*^ human embryonic cells compared with telomerase-negative cells. Analyses of mouse embryonic cells revealed a similar trend, although the difference between the telomerase-positive and telomerase-negative cells in the prevalence of the MLME cells did not reach the threshold of statistical significance (Table 3).

**Table 3:** Prevalence of single cells manifesting MLME phenotypes within populations of *TERTneg* and *TERTpos* cells of human and mouse embryos.

Classification category	Number of cells, n	MLME phenotype, n	MLME, %	P value
Human embryo (n = 1529)	1529	267	17.5	
TERT negative (n = 710)	710	92	13.0	4.2338E-06
TERT positive (n = 819)	819	175	21.4	4.2338E-06
Top 3% TERTpos (n = 25)	25	17	68.0	2.1534E-08
Bottom 97% TERTpos (n = 794)	794	158	19.9	0.00178042
Top 5% TERTpos (n = 41)	41	24	58.5	1.993E-09
Bottom 95% TERTpos (n = 778)	778	151	19.4	0.00671443
Top 10% TERTpos (n = 82)	82	36	43.9	5.9454E-09
Bottom 90% TERTpos (n = 737)	737	139	18.9	0.02051266
Mouse embryo (n = 259)	259	52	20.1	
TERT negative (n = 178)	178	32	18.0	0.06008626
TERT positive (n = 81)	81	20	24.7	0.06008626
Top 9% TERTpos (n = 7)	7	2	28.6	0.27984881
Top 16% TERTpos (n = 13)	13	3	23.1	0.25279801

The MLME phenotype was defined in individual human and mouse embryonic cells as described in the legend to the Table 2. The relative ranks of individual cells were defined by the 23-gene signature score. P values were estimated using the hypergeometric distribution test.

Based on these analyses, we conclude that the MLME cells can be readily identified by directly scoring the expression of genetic markers of distinct embryonic lineages in individual cells isolated from heterogeneous populations of the human and mouse preimplantation embryos. Consistent with their proposed role in embryonic development, the MLME cells appear before the lineage segregation in both human and mouse embryos. However, the association of the MLME phenotype with telomerase expression seems statistically significant only in human embryonic cells and the results of detailed expression profiling analyses of species-specific genetic markers of distinct lineages support the hypothesis that the MLME cells identified in human and mouse embryos represent transcriptionally and phenotypically distinct populations (Supplemental Tables S4.1–S4.4).

## 3 Discussion

**Fig. 6:**
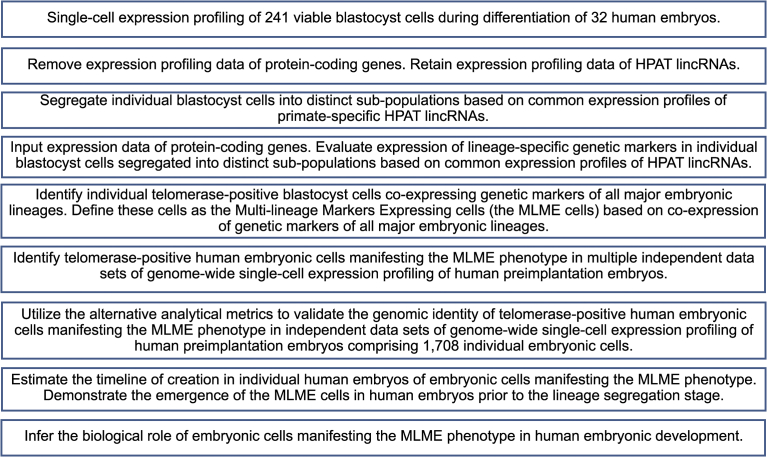
Summary of key experimental and analytical steps of identification and characterization of telomerase-positive MLME cells in human preimplantation embryos. Detailed descriptions of multiple lines of experimental observations providing the foundation of the hypothesis that MLME cells may function as putatively immortal multi-lineage precursor cells (iMPC) during human embryogenesis are reported in the main text, Figs. 1–5; Tables 1, 2 and 3; and in the Supplemental information available online.

A brief summary of key experimental and analytical steps revealing the presence in human preimplantation embryos of telomerase-positive cells co-expressing genetic markers of all major embryonic lineages as well as extraembryonic tissues is shown in Fig. 6. Importantly, telomerase-positive MLME cells appear to emerge in human embryos prior to the lineage segregation stage, which is consistent with their role as putatively immortal multi-lineage precursor cells. Collectively, the experimental findings and theoretical considerations presented in this contribution support the hypothesis of a developmental pathway creating embryonic lineages and extraembryonic tissues from pre-lineage cells manifesting multi-lineage precursor phenotype (the MLME cells). Because the MLME cells appear to emerge at the E4 stage of human preimplantation embryogenesis, it seems reasonable to infer that these observations support the hypothesis of a developmental pathway of the creation of major embryonic lineages as well as extraembryonic tissues from the MLME cells prior to the formation of the Inner Cell Mass (ICM). In contrast to the classical view of the ICM as the lineage segregation stage, the ICM formation may reflect a critically-important embryonic stage of the creation of a singular coherent multicellular structure within which cells of distinct embryonic lineages congregate and initiate the collaborative effort of implementation of high-complexity developmental programs leading to the creation of highly sophisticated multicellular organism.

Several molecular and genetic features assigned in this study to the human embryonic MLME cells were previously characterized as critical regulatory elements of human preimplantation embryogenesis and/or associated with the naïve pluripotency state of hESC. Examples of these commonly defined features include high expression of lincRNAs encoded by genetic loci derived from the primate-specific retrovirus HERVH; high expression of known genetic markers of the naïve pluripotency state, including pluripotency inducers and/or regulators; high expression of *TERT* gene; increased expression of master pluripotency and developmental regulatory genes *OCT4; NANOG; SOX2* (ONS); sustained activity of both SALL4/NANOG and SOX2/POU5F1 enhancers' networks designed to maintain the stemness regulatory circuitry; transcriptional control of the HPAT lincRNAs by NANOG interacting with LTR7/HERVH promoter. Taken together, these observations are consistent with the idea that the MLME cells appear to resemble embryonic cells acquiring the naïve pluripotency phenotype in vivo during early-stages of human preimplantation embryogenesis.

The key novel element of the experimental approach implemented in this study constitutes the selection of human embryos with the prospectively-defined high likelihood of progression to the viable blastocyst stage. Isolation of individual human embryonic cells from the VHB separated into early and late blastocyst cell populations based on strict morphological criteria defined by the classic embryology was important to ensure the high likelihood of the validity of experimental observations. We believe that this approach provides a more stringent basis to reliably control human embryonic cells' viability by essentially excluding the presence of aneuploid cells. High level of certainty that we analyzed the euploid viable human embryonic cells should mitigate potential limitations of previous studies utilizing the single cell expression profiling approaches of the analysis of human preimplantation embryogenesis and relying solely on segregation of individual cells based on the time after fertilization. One of the obvious flaws of this timeline-based approach is that integration of the single cell analyses from different human embryos would inevitably generate the developmentally heterogeneous populations simply because the lack of synchronization of embryonic development among individual human embryos.

Application of this experimental strategy in combination with the state-of-the-art single cell gene expression analysis and spatiotemporal reconstruction of human blastocyst differentiation facilitated the development of a three-dimensional model in which individual cells were faithfully mapped into distinct expression domains [24]. Using this approach, we were able to extract and describe the key novel mechanistic information emanated by the single-cell expression analyses of protein-coding genes [24]. Specifically, these experiments revealed a previously uncharacterized role for *MCRS1*, *TET1*, and *THAP11* genes in epiblast formation and their ability to induce naive pluripotency *in vitro*. Identification and characterization of the MLME cells highlighted a key role of *MTTH/HPAT* lincRNAs regulatory axis in a two-wave progression sequence of human embryonic development (Fig. 1G). Consistent with the proposed model, it has been reported that Sall4 interacts with Nanog and co-occupies Nanog binding sites in embryonic stem cells [49]. It has been suggested that Sall4 and Nanog proteins form a regulatory circuit similar to that of Oct4 and Sox2 proteins to maintain the phenotypic identity of embryonic stem cells [49]. Our experiments indicate that HPAT lincRNAs may contribute to the sustained activity of both SALL4/NANOG- and SOX2/POU5F1-associated stemness regulatory circuitry in human embryonic cells, in part, by increasing the expression levels of the *SALL4* and *SOX2* genes (Fig. 1F and G). Significantly, sequence conservation analyses of *HPAT* lincRNAs, expression profiling of which facilitated identification of the MLME cells, indicated that at least some transcripts represent human-specific (unique to human) regulatory molecules (Supplemental Table S5). A hallmark feature of human-specific integration sites of stem cell-associated retroviral sequences, including specific *HPAT* lincRNAs (Supplemental Table S5), is deletions of ancestral DNA [46].

It has been shown that activation of specific HERV loci, including *HPAT3* and *HPAT4* lincRNAs, occurs in human zygotes following *in vitro* fertilization of mature oocytes [50]. Gene expression signature analysis of early-stage human preimplantation embryogenesis revealed that “premature” activation of HERVH expression appears associated with increased likelihood of failure to produce viable blastocysts by human fertilized oocytes [50]. These observations support the hypothesis that premature activation of HERV expression in early-stage human embryos may reflect failed attempts to create the MLME cells. Collectively, this analysis suggests that premature activation of HERV expression in human embryonic cells may cause cell cycle arrest and loss of cell viability, leading to exceedingly high level of cellular extinction events.

Observations supporting the hypothesis that the MLME cells are created during the human blastocysts differentiation were facilitated by the convergence of two synergistic analytical advances: identification and initial structural-functional characterization of genomic loci encoding *HPAT* lincRNAs and technical implementations of the idea of performing a single-cell analysis of VHB differentiation by segregating early-stage and late-stage blastocyst cells. Segregation and transcriptional profiling of individual VHB cells separated in time by just few critical hours of embryonic development enabled to dissect the dynamics of human blastocyst differentiation with unprecedented precision which was validated by the accurate classification of distinct blastocyst lineages based on the expression profiles of just three HPAT lincRNAs, namely *HPAT21*; *HPAT15*; and *HPAT2*.

Additional experiments would be required to conclusively distinguish between the telomerase-negative cells being eliminated during the preimplantation embryogenesis and unsynchronized telomerase expression in individual blastomeres. It would require the lineage tracing analysis either by time lapse imaging or genetic tracing strategies in individual human embryos. Based on the results of present analyses, these additional experiments seem feasible and fully justified to demonstrate that putatively immortal *TERT*^*(+)*^ multi-lineage precursor cells are created in cleavage-stage human embryos which later will differentiate into trophectoderm, primitive endoderm, and epiblast cells whereas a majority of all other cells will die and disappear.

Specific experimental techniques will be required to analyze if some aneuploid blastomeres (or embryonic cells with point mutations) will stop divide and die in a cleavage-stage embryo and only the euploid blastomeres will further develop and finally a chimeric embryo will become a healthy euploid blastocyst. Development and application of the single cell DNA-RNA simultaneous sequencing technique will help to determine conclusively if the gene expression pattern of an aneuploid blastomere in a chimeric embryo is abnormal and different from that of euploid blastomeres in the same embryo. In this type of experiments the link could be observed between the abnormal gene expression in an aneuploidy blastomere, cell cycle arrest, activation of apoptosis and death.

Discovery of HPAT lincRNAs was facilitated by application of a hybrid RNA sequencing technique enabling the identification of more than 2,000 new lincRNA transcript isoforms, of which 146 were specifically expressed in pluripotent hESCs [51]. Follow-up experiments revealed that 23 genomic loci encoding transposon-derived transcripts and termed *HPAT1-HPAT23* lincRNAs are most abundantly and specifically expressed in human pluripotent cells [17]. It has been demonstrated that three primate-specific transposon-derived HPAT lincRNAs (*HPAT2, HPAT3* and *HPAT5*) may modulate cell fate in human preimplantation embryo development and during nuclear reprogramming [17]. Our experiments identified five most abundant HPAT lincRNAs (*HPAT21; HPAT15; HPAT5; HPAT2;* and *HPAT3*) expression of which was observed most frequently in human blastocyst cells. Interestingly, expression of the *HPAT21* lincRNA was detected in 92% and 100% of cells recovered from early and late stage-developing blastocysts, respectively. Additional experiments will be required to conclusively establish that this lincRNA could be defined as the pan marker of human blastocyst cells.

The conclusive unequivocal validation of the hypothesis that during human blastocyst differentiation the unique populations of putatively immortal multi-lineage precursor cells are created, which then fuels the emergence of the trophectoderm, primitive endoderm, and epiblast lineages, as well as induction of the naïve pluripotency state *in vivo*, may prove challenging. It would require the direct isolation and recovery of these unique cells from the human blastocysts (or, perhaps, earlier stages of human embryogenesis) and their comprehensive molecular and functional characterization. However, our observations that expression of HPAT lincRNAs implicated in human blastocyst differentiation is markedly up-regulated in human cells acquiring the genetically-induced naïve pluripotency state suggest that it might be possible to design the experimental protocols for genetic engineering of the MLME-resembling cells. It will be of interest to investigate the developmental potential and translational utility of the MLME cells for regenerative medicine.

Collectively, our observations demonstrate that during human preimplantation embryogenesis the MLME cells are created and suggest that subsequent differentiation and specialization of these cells may contribute to the emergence of the trophectoderm, primitive endoderm, and epiblast lineages, as well as transition to the naïve pluripotency state. Our initial observations and conclusions are based on single cell expression analyses of 241 individual cells recovered from 32 human embryos at early and late stages of blastocyst differentiation. We validated our principal findings and the key predictions of our model by the *in silico* follow-up analyses on 1,708 individual embryonic cells recovered from more than 100 human embryos at different stages of preimplantation development (Fig. 5), thus providing a solid foundation supporting the hypothesis of a developmental pathway of creation major embryonic lineages from putatively immortal multi-lineage precursor cells.

## 4 Conclusions

Cell cycle arrest, loss of cell viability, and developmental failures occur in 50–80% of cleavage-stage embryos, indicating that a majority of in vitro fertilization attempts would fail to generate VHB. Multiple lines of compelling experimental evidence documenting the high probability of developmental failures during the early-stage of human embryogenesis challenge the validity of recent analyses of individual cells isolated from human embryos at the early-stage of preimplantation embryonic development. To address this significant experimental problem, we carried-out single-cell expression profiling of individual embryonic cells isolated from VHB. One of the main outcomes of the analysis of individual viable cells isolated from human embryos was identification and initial characterization of the telomerase-positive subpopulation of embryonic cells co-expressing genetic markers of the naïve pluripotency induction and three major embryonic lineages. We then utilized gene expression signatures of telomerase-positive cells co-expressing genetic markers of all embryonic lineages to validate our main findings by analyzing 1,708 individual cells recovered from more than 100 human embryos and 259 mouse cells isolated at different stages of preimplantation embryogenesis. Employing this approach, we have made several novel experimental observations supporting the following main conclusions:

i) Our experiments identify five most abundant *HPAT lincRNAs* (*HPAT21; HPAT15; HPAT5; HPAT2;* and *HPAT3*) which were detected in 25% to 97% of VHB cells;
ii) We demonstrate that segregation of human blastocyst cells into distinct populations based on a single-cell expression profiling of three *HPATs* (*HPAT21/HPAT2/HPAT15*) appears to inform specific molecular and cellular events of naïve pluripotency induction (MTTH/HPAT lincRNA/SALL4/SOX2 axis) and accurately captures some of the key features of cellular diversity during human blastocyst differentiation;
iii) We observe that at the E4 stage of human preimplantation embryogenesis, telomerase-positive cells are created co-expressing genetic markers of all three major embryonic lineages;
iv) We propose that telomerase-positive cells co-expressing genetic markers of three embryonic lineages may function in human embryos as putative multi-lineage precursor cells to then differentiate into trophectoderm, primitive endoderm and pluripotent epiblast lineages.

Our experimental findings and conclusions are in agreement with recent reports suggesting that single-cell gene expression profiling of cellular differentiation events during human preimplantation embryogenesis and the analysis of created *in vivo* naive pluripotent embryonic stem cells represent some of the most promising approaches to reliably benchmark ground state pluripotency regulatory networks in human cells [17, 24, 52, 53]. One of the potentially important practical implications of our experiments is that expression analysis of three primate-specific retrotransposon-derived HPAT lincRNAs (*HPAT21; HPAT2; HPAT15*) could be utilized as informative genetic markers of human embryonic lineages.

Taken together, observations reported in this contribution appear to challenge the prevailing views on the biological, molecular and genetic mechanisms underlying early embryonic development of Modern Humans which define the formation of the Inner Cell Mass (ICM) as the main and only path to the creation of distinct embryonic lineages. The emergence and sustained presence of embryonic cells manifesting the Multi-Lineage Markers Expression phenotype (the MLME cells) at the E4 through E7 stages of human preimplantation embryogenesis may reflect a developmental pathway of the creation of major embryonic lineages as well as extraembryonic tissues which begin to operate prior to the ICM stage. In this context, the ICM formation may represent a critically-important stage of the creation of a singular coherent embryonic structure within which cells of distinct embryonic lineages congregate to continue differentiation into more specialized cell types and begin a multidimensional cooperation on implementation of high-complexity instructions from the compendium of developmental programs with the ultimate goal of the creation of highly sophisticated multicellular organism.

## 5 Materials & methods

### 5.1 Source and procurement of human embryos

Supernumerary human blastocysts from successful in vitro fertilized cycles were donated for basic research and were obtained with written informed consent from all subjects from the Stanford University RENEW Biobank.

De-identification was performed according to the Stanford University Institutional Review Board approved protocol no. 10466 entitled “The RENEW Biobank”, and all methods, experimental protocols, and the molecular analysis of the embryos were in compliance with all relevant institutional guidelines and regulations.

### 5.2 Human blastocyst retrieval and disaggregation into single cells

Frozen human blastocysts at day 5 and day 6 post fertilization of preimplantation development were thawed according to Quinn's advantage thaw kit (CooperSurgical, Trumbull, CT) as previously described (46). Briefly, cryocontainers were removed from liquid nitrogen and exposed to air for 10 sec before incubating in a water bath at 37 °C until thawed. Next, embryos were transferred to 0.5 M and 0.2 M sucrose solution for 10 min each and washed in diluent solution for 10 min at room temperature (RT). Blastocysts were then incubated in Quinn's Advantage Blastocyst Medium supplemented with 10% serum protein substitute under mineral oil at 37 °C with 6% CO2, 5% O2 and 89% N2, standard human embryo culture conditions for 5 or 12 hours respectively. Only morphological normal looking blastocysts with Gardner grading 1-3AABB (early blastocyst) and 4-6AABB (late blastocyst) (Gardner et al., 2000) were used for the further analysis. Blastocysts were treated with acidic Tyrode's solution and washed in Dulbecco's PBS (DPBS) substituted with 1 mM EDTA and 0.5% BSA. After incubation in 0.005% trypsin-EDTA for 15 mins blastocyst were washed in DPBS and collected for the following C1-analysis. The *MTTH* model of induced human naïve pluripotent cells was established and analyzed using the OCT4-ΔPE-GFP line exactly as previously described [24]. Individual gene overexpression and gene targeting experiments were performed as previously reported using previously characterized reagents and cell lines [17, 24].

### 5.3 Sequence conservation analyses of *HPAT* lincRNAs

The sequence conservation analyses of HPAT lincRNAs were performed as previously described [3, 54, 55]. In brief, the analysis is based on the University of California Santa Cruz (UCSC) LiftOver conversion of the coordinates of human blocks to corresponding non-human genomes using chain files of pre-computed whole-genome BLASTZ alignments with the MinMatch of 0.1 (for human-specific sequences) and 0.95 (for highly conserved sequences) and other search parameters in a default setting (http://genome.ucsc.edu/cgi-bin/hgLiftOver). Both direct and reciprocal conversions were performed for each analyzed *HPAT* lincRNA sequence. Extraction of BLASTZ alignments by the LiftOver algorithm for a human query generates a LiftOver output “Deleted in new”, which indicates that a human sequence does not intersect with any chains in a given non-human genome. This indicates the absence of the query sequence in the subject genome and was used to infer the presence or absence of the human sequence in the non-human reference genome. Human-specific regulatory sequences were manually curated to validate their identities and genomic features using a BLAST algorithm and the latest releases of the corresponding reference genome databases for time periods between April, 2013 and December, 2016. Sequences of HPAT lincRNAs (accession numbers LT999881; LT999883; LT999884; LT999885; LT999886; LT999887; LT999888; LT999889; LT999890; LT999891; LT999892; LT999893; LT999894; LT999895; LT999896; LT999897; LT999898) were deposited at the European Nucleotide Archive (ENA) and will be permanently publicly available from the ENA browser at http://www.ebi.ac.uk/ena/ after the public release date on June 24, 2018.

### 5.4 Sequence of the core promoter of LTR7 reporter that was cloned upstream of Tag-RFP

Sequence in red depicts forward and reverse primer for amplification from genomic DNA. Sequence shaded green depicts the core promoter region including TATA box, GC/GT box and the Inr element.

TGTCAGGCCTCTGAGCCCAAGCTAAGCCATCGCATCCCCTGTGACTTGCACGTATAAGCCCAGATGGCCTGAAGTAACTGAAGAATCACAAAAGAAGTGAATATGCCCTGCCCCATCTTAACTGATGACATTCCACCACAAAAGAAGTGTAAATGGCCAGTTCTTGCCTTAAGTGATGACATTACCTTGTGAAAGTCCTTTTCCTGGCTCATCCTGGCTCAAAAGGCACCCCCACTGAGCACCTTGTGACCCCCACTCCTGCCCGCCAGAGAACAAACCCCCTTTGACTGTAATTTTCCTTTACCTACCCAAATCCTATAAAACGGCCCCACCCTTATCTCCGTTTGCTGACTCTTTTCGGACTCAGCCTGCCTGCACCCAGGTGAAATAAACAGCCTTATTGCTCACACAAAGCCTG.

### 5.5 Promoter analysis of HPAT transcripts

By comparing NANOG DNA occupancy from our ChIP-seq data, we predicted three putative binding motifs within our LTR7 reporter cassette and compared them against databases of known motifs (TomTom). However, we failed to identify similar motifs bound by known transcription factors, suggesting that the predicted motifs are noncanonical NANOG motifs (Fig. 3). To identify the regulatory regions of the LTR7 core promoter, we then deleted the predicted motifs in our LTR7 reporter cassette and generated lentiviral reporter constructs (Fig. 3) that were used to infect BJ fibroblasts. With transient NANOG overexpression, all three deletions significantly impaired Tag-RFP reporter expression, with Motif 2 revealing the lowest reporter activity (similar to uninfected negative control) (Fig. 3). Thus, ChIP-Seq analysis followed by deletion mapping of the LTR7 core promoter element identified three binding motifs that are crucial for transcriptional activation and are occupied by NANOG.

### 5.6 Description of two public datasets analyzed for HPAT lincRNA expression in human preimplantation embryos

**SRP018525**: Xue et al [12]: Sample inventory: 29 cells total (mouse or exome sequences not subject to analysis), made up of pronuclei (3), oocytes (3), zygotes (2), 2-cell (3), 4-cell (4), 8-cell (11, one of which was pooled rather than single cell), and morulae (3). cDNA was generated from single cells with the exception of one pooled 8-cell sample and cDNA was subjected to Illumina HiSeq 2000 paired end sequencing.

**SRP011546**: Yan et al. [13]: Sample inventory: 124 cells total, made up of oocytes (3), zygotes (3), 2-cell (6), 4-cell (12), 8-cell (20), morulae (16), late blastocyst (30), and hESC cultured cells (34). cDNA was generated from single cells, then subjected to 10 cycles of PCR prior to Illumina HiSeq 2000 single end sequencing.

### 5.7 RNA-seq data processing pipeline

#### 5.7.1 Fit samples to a format required by SpliceMap

Samples from SRP018525 containing 49 base pair read lengths were extended by one unknown “N” base prior to SpliceMap (SpliceMap requires at least 50 bp). For those samples SpliceMap was run with an additional mismatch allowed, and an additional seed mismatch allowed beyond the default parameters.

#### 5.7.2 Remove overrepresented sequences

Over represented sequences (exact matching sequences comprising more than 0.1% of the total sequence content) were removed.

#### 5.7.3 Use SpliceMap to align reads

SpliceMap was used with default settings (except for where mentioned above) to map reads to chromosomes 1–22, X and Y on hg19 with reference annotations plus *HPAT* annotations.

#### 5.7.4 Calculate per-gene and per-transcript FPKMs

Cufflinks was called with the -G option to calculate FPKM for reference transcripts and *HPATs* only.

#### 5.7.5 Exclude sequences based on anomalous FPKM

MicroRNAs were excluded from the analysis due to problems with cufflinks calculating FPKM on the very short reference sequences.

#### 5.7.6 Generate FPKM heatmaps

For plotting purposes, FPKM was set to zero when Cufflink status was not “OK”. FPKM was log2 transformed for counts greater than 2 and FPKM/2 for values less than or equal to 2. Negligible random values greater than zero and less than 0.00001 were added to FPKM values to simplify the clustering computation for tie values. Heatmaps were generated using the R {gplots} “heatmap.2” enhanced heatmap function.

To reduce clutter in the heatmaps (other than *HPAT* only heatmaps), the least informative genes, or what was mainly unexpressed genes, could be removed by iteratively removing the gene with the smallest contribution to the variance accounted for by eigenvectors accounting for 95% of the variance in the dataset. Halt the removal of the least informative genes when the entropy of the dataset had changed by 10% seemed effective, with large datasets. However for the embryonic gene panel plotted with the much smaller SRP018525 dataset, a threshold of a 7% change appeared to retain a more reasonable proportion of relevant information.

The list of embryo-specific genes refers to expression analyses of the following 89 genes:

*AFP, BRIX1, CD34, CD9, CDH5, CDX2, COL1A1, COMMD3, CRABP2, DDX4, DES, DIAPH2, DNMT3B, DPPA3, EDNRB, EOMES, FGF4, FGF5, FLT1, FN1, FOXA2, FOXD3, GABRB3, GAL, GATA4, GATA6, GBX2, GCG, GCM1, GDF3, GRB7, HBB, HBZ, HCK, IAPP, IFITM1, IFITM2, IGF2BP2, IL6ST, INS, KIT, KRT1, LAMA1, LAMB1, LAMC1, LEFTY1, LEFTY2, LIFR, LIN28A, LIN28B, MAPK7, MYF5, MYOD1, NANOG, NES, NEUROD1, NODAL, NOG, NR5A2, NR6A1, NUMB, OLIG2, PAX4, PAX6, PDX1, PECAM1, PODXL, POU5F1, PTEN, PTF1A, REST, RUNX2, SALL4, SEMA3A, SERPINA1, SFRP2, SOX17, SOX2, SST, SYCP3, T, TAT, TDGF1, TERT, TFAP2C, TFCP2L1, UTF1, WT1, ZFP42*.

After excluding miRNAs, the reference annotation file contained 21120 Refseq genes and 25934 Refseq transcripts, along with 23 *HPAT* genes and 42 *HPAT* transcripts.

### 5.8 ChIP-seq experiments

ChIP assays were performed from approximately 10^7^ cells per experiment, according to previously described protocol with slight modifications [17]. Briefly, cells were crosslinked with 1% formaldehyde for 10 min at room temperature and formaldehyde was quenched by addition of glycine to a final concentration of 0.125 M. Chromatin was sonicated to an average size of 0.5–2 kb, using Bioruptor (Diagenode). 50–75 μL of protein G dynal beads (Invitrogen) were used to capture 3–5 μg of antibody in phosphate citrate buffer pH = 5.0 (2.4 mM citric acid, 5.16 mM Na_2_HPO_4_) for 30 min at 27 °C. Antibody bead complexes were rinsed 2x with PBS and added to sonicated chromatin and rotated at 4 °C overnight. 10% of chromatin was reserved as “input” DNA. Magnetic beads were washed and chromatin eluted, followed by reversal of the crosslinkings and DNA purification. Resultant ChIP DNA was dissolved in TE. Results were verified with qPCR of 7 selected regions [17].

### 5.9 Design of LTR7-reporter (mutant) construct, lentiviral transduction and establishment of reporter cell lines

An LTR7 promoter element was amplified using genomic DNA and two specific primers (see section 5.4). Amplified regions were gel purified and cloned into a pENTR 5′TOPO vector (Life Technologies) to continue with the MultiSite Gateway Technology. Deleted LTR7 fragments were ordered as individual gBlocks and amplified with specific primers according to the manufacturer's instructions. Amplified LTR7 fragments were cloned into a pENTR 5′TOPO vector (Life Technologies) to continue with the MultiSite Gateway Technology. Clones were transformed into One Shot Competent *E. coli*, DNA was purified and sequenced and positive clones were used for and cloned into a destination vector by recombination with a Tag-RFP-vector (reporter) and a lentiviral backbone vector (destination vector p2k7). Recombined vector product was transformed into Stbl3 One Shot Competent *E. coli*, DNA was purified and sequenced. Next, lentivirus production was achieved with the delta 8.9 (containing gag, pol, rev genes) and the vsv-g plasmid system as described [17]. Briefly, lentivirus backbone and helper vectors were transfected into 293FT cells (Life Technologies) for viral packaging. Virus was collected at 48 h and 72 h after transfection, virus was collected, filtered and concentrated with Lenti-X Concentrator (Clontech). Target cells (BJ fibroblasts, BJ.iPSCs) were infected in the presence of polybrene (8 mg/ml; Sigma-Aldrich) and cells were selected either for 2 weeks under selection pressure (G418; 500 μg/ml final concentration) or through Fluorescence-activated cell sorting (FACS) of Tag-RFP positive cells.

### 5.10 Flow cytometry

Cells were treated with pre-warmed Accutase (Innovative Cell Technologies), washed once in PBS and resuspended in PBS + 5% FBS prior sorting. Cells were sorted onto pre-coated growth factor reduced matrigel (BD Biosciences) plates.

### 5.11 Statistical analysis

For single-cell analysis individual cells were considered as biological replicates (n = 241). Calculated primer efficiencies revealed a normal distribution determined by the Shapiro-Wilk test. For normal distributed data we used the two-tailed Student's t-test for significance calculations. Nonparametric statistical approaches were applied for data not following a normal distribution. Specifically, we chose the Kurskal-Wallis test for independent and unequal sized sample calculations. Statistical significance was set to p < 0.05 for gene expression analysis (n > 3). Only Bayesian network connections with p < 0.05 are shown. Correlation analysis revealed only significant correlations with p < 0.05. Resulting values (for each experiment) were subject to two-tailed Student's t-test and two-tailed Fisher's exact test. Error bars represent SEM in all statistical significance tests. Follow-up validation analyses were performed using five independent validation data sets of single-cell genome-wide expression profiling analyses of human and mouse preimplantation embryogenesis (Supplemental Table S1), including genome-wide expression profiles of 1,708 individual human cells [12, 13, 25, 26] and 259 individual mouse cells [41].

## Declarations

### Author contribution statement

Gennadi Glinsky: Conceived and designed the experiments; Analyzed and interpreted the data; Contributed reagents, materials, analysis tools or data; Wrote the paper.

Jens Durruthy-Durruthy, Mark Wossidlo, Vittorio Sebastiano: Conceived and designed the experiments; Performed the experiments; Analyzed and interpreted the data; Contributed reagents, materials, analysis tools or data; Wrote the paper.

Edward J. Grow: Conceived and designed the experiments; Performed the experiments; Analyzed and interpreted the data; Contributed reagents, materials, analysis tools or data.

Jason L. Weirather: Conceived and designed the experiments; Performed the experiments; Analyzed and interpreted the data.

Kin Fai Au, Joanna Wysocka: Conceived and designed the experiments; Analyzed and interpreted the data; Contributed reagents, materials, analysis tools or data.

### Funding statement

This work was supported by the grants: U54-1U54HD068158-01 (Stanford University Center for Reproductive and Stem Cell Biology), RB3-2209 (California Institute of Regenerative Medicine), start-up funds to V.S. V.S. is also supported by Siebel Stem Cells Scholarship. J.D.D. is supported by Fritz Thyssen Fellowship.

### Competing interest statement

The authors declare no conflict of interest.

### Additional information

No additional information is available for this paper.

## Appendix A Supplementary data

Supplemental Note 1 Supplemental Note 1

Supplemental Table S5 Supplemental Table S5

Supplemental Data Set S1 Supplemental Data Set S1

Supplemental Data Set S2 Supplemental Data Set S2

Supplemental Information Text Supplemental Information Text

Supplemental Movie Visualization of telomerase-positive embryonic cells in the human blastocyst viewer. Web-based online tool to study early cell fate decisions in the human blastocyst visualizes telomerase-positive human embryonic cells (http://web.stanford.edu/∼sunilpai/HumanBlastocystViewer.html). Supplemental Movie
