# Supplementary material for: Single cell expression analysis of primate-specific retroviruses-derived HPAT lincRNAs in viable human blastocysts identifies embryonic cells co-expressing genetic markers of multiple lineages
Source: Heliyon. 2018 Jun 28;4(6):e00667. doi: 10.1016/j.heliyon.2018.e00667 (PMC6039856; doi:10.1016/j.heliyon.2018.e00667)
Supplement: Supplemental Note 1 [file mmc1.docx]

**SUPPLEMENTAL NOTE 1**

**Single cell expression analysis of primate-specific retroviruses-derived HPAT lincRNAs in viable human blastocysts identifies embryonic cells co-expressing genetic markers of multiple lineages**

Gennadi Glinsky^1†*^, Jens Durruthy-Durruthy^2,†^, Mark Wossidlo^3,†^, Edward J. Grow^4^, Jason L. Weirather^5^, Kin Fai Au^5^, Joanna Wysocka^4^, Vittorio Sebastiano^2,^*

^†^contributed equally

*correspondence

**Affiliations**

^1^Institute of Engineering in Medicine, University of California, San Diego

9500 Gilman Dr. MC 0435

La Jolla, CA 92093-0435, USA

^2^Department of Obstetrics and Gynecology, Institute for Stem Cell Biology & Regenerative Medicine, Stanford University, Stanford, CA 94305.

^3^Department of Cell- and Developmental Biology, Center of Anatomy and Cell Biology, Schwarzspanierstrasse 17, 1090 Vienna, Austria

^4^Department of Chemical and Systems Biology, Stanford University, Stanford, California, USA

^5^Department of Internal Medicine & Department of Biostatistics, University of Iowa, Iowa City, IA, USA

Correspondence:

Gennadi Glisnky, MD, Ph.D.

Institute of Engineering in Medicine

University of California, San Diego

9500 Gilman Dr. MC 0435

La Jolla, CA 92093-0435, USA

Email: [gglinskii@ucsd.edu](mailto:gglinskii@ucsd.edu)

Web: <http://iem.ucsd.edu/people/profiles/guennadi-v-glinskii.html>

**HPAT expression-guided classification of 241 individual human embryonic cells recovered during differentiation of viable blastocysts**

**HPAT lincRNA expression-guided stratification of human blastocyst cells**

Using a hybrid RNA sequencing technique we have previously identified and characterized 23 novel TGE-derived lincRNAs that are highly expressed in hESC and termed human pluripotency-associated transcripts (*HPATs*) [17; 36]. We have developed and implemented qPCR-based assays for sixteen HPATs [17; 18] and thought to begin our analytical effort by focusing on the exploration of the expression patterns of these sixteen TGE-derived lincRNAs during human blastocyst differentiation (Fig. 1). This analysis identifies five TGE-derived lincRNAs (*HPAT21; HPAT15; HPAT5; HPAT2;* and *HPAT3*) which were observed most frequently and detected in at least 25% of analyzed human blastocyst cells (Supplemental Fig. S1). Notably, three out of these five lincRNAs were recently implicated in regulation of nuclear reprogramming and pluripotency induction during human preimplantation embryo development [17]. Expression of *HPAT21*, the most abundant lincRNA, was detected in 92% of cells recovered from the early blastocysts and 100% of the late blastocyst cells, whereas expression of the second-ranked lincRNA *HPAT15* was observed in 56% and 48% of the early and late blastocyst cells, respectively. Notably, the expression patterns of the vast majority (94%) of lincRNAs did not manifest significant changes in the early and late blastocyst cells (Supplemental Fig. S1), indicating that expression of individual TGE-derived lincRNAs remains relatively stable and may serve as a reliable marker of distinct sub-populations emerging during human blastocyst differentiation.

The detailed analysis of expression patterns of all individual lincRNAs in 241 individual human blastocyst cells revealed that there is only one cell manifesting *HPAT21/HPAT2/HPAT15*-null phenotype and remaining human blastocyst cells express varying combinations of these three lincRNAs (Fig. 1B). The results of the present analyses suggest that expression profiles of just three lincRNAs capture a full spectrum of cellular phenotypes created during differentiation of human blastocysts. It was of interest to determine whether detailed analysis of sub-populations of cells defined by the expression patterns of these 3 genetic markers would inform events of naïve pluripotency induction and faithfully describe the evolution of cellular diversity during human blastocyst differentiation.


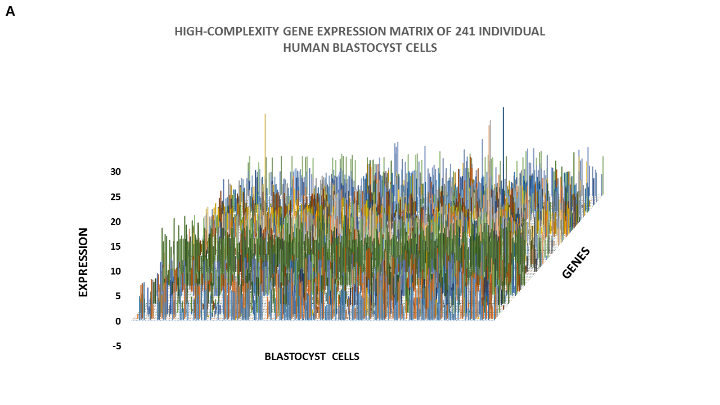

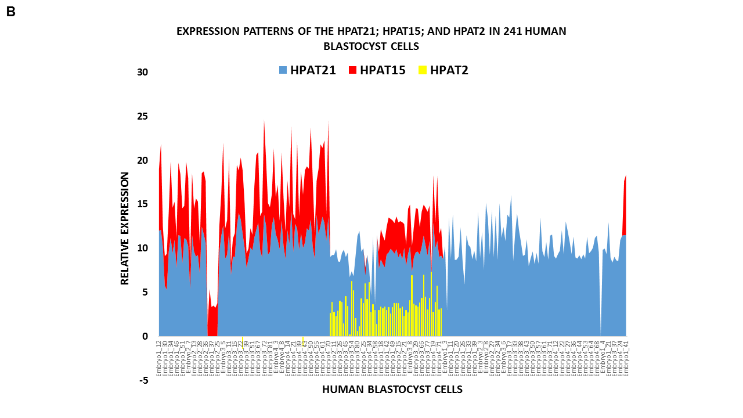

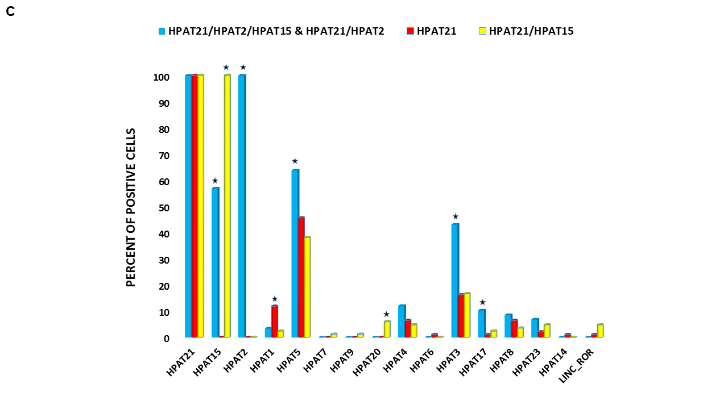

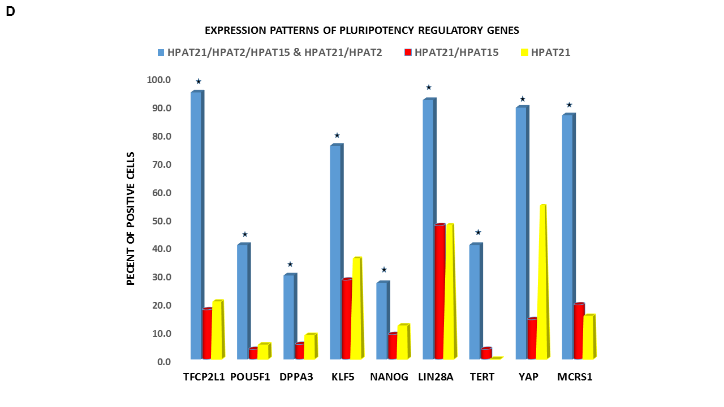

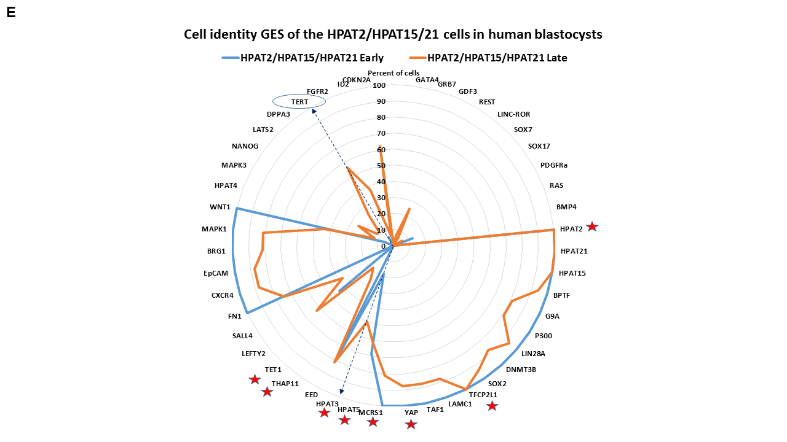

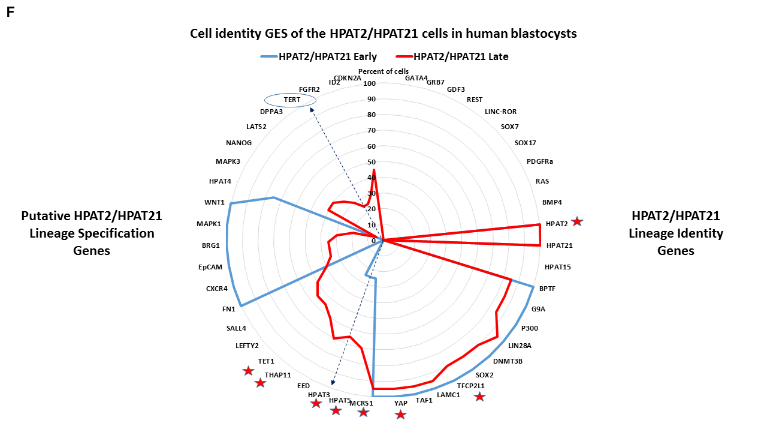


**Fig. 1.** **Single-cell gene expression analysis of HPAT lincRNAs captures the entire spectrum of a cellular diversity during human blastocyst differentiation.**

1. A high complexity expression matrix of 89 genes expression of which were measured in 241 individual human blastocyst cells.
2. Expression profiles of the *HPAT21; HPAT15*; and *HAPT2* lincRNAs define major sub-populations of human blastocyst cells. A color-coded stacked area plot depicts relative expression values of designated HPAT lincRNAs in each individual blastocyst cell. Note that there is only one cell manifesting *HPAT21/HPAT2/HPAT15*-null phenotype.
3. Expression patterns of HPAT lincRNAs in distinct sub-populations of human blastocyst cells. Stars designate sub-populations harboring the significantly enriched numbers of cells expressing defined genetic markers. P values were estimated using two-tailed Fisher’s exact test and reported in the Supplemental Table S2.
4. Expression patterns of pluripotency regulatory genes in distinct sub-populations of human blastocyst cells. Stars designate sub-populations harboring the significantly enriched numbers of cells expressing defined genetic markers.
5. Cell identity gene expression signature (GES) of the *HPAT21 ^(+)^ HPAT15 ^(+)^ HPAT2 ^(+)^* cells recovered from human blastocysts. Stars designate transcripts encoded by the genomic loci that were implicated in the regulation of the human pluripotency state. Positions of colored lines’ heights inside the circle reflect the percentage of cells within a population that express transcripts encoded by the corresponding genes names of which are listed on the circle.
6. Cell identity gene expression signature (GES) of the *HPAT21 ^(+)^ HPAT15 ^(-)^ HPAT2 ^(+)^* cells recovered from human blastocysts. Stars designate transcripts encoded by the genomic loci that were implicated in the regulation of the human pluripotency state. Positions of colored lines’ heights inside the circle depict the percentage of cells within a population that express transcripts encoded by the corresponding genes listed on the circle.

**Single-cell expression profiling of HPAT expression-defined sub-populations during human blastocyst differentiation**

Early and late blastocyst cells were segregated into distinct sub-populations based on expression patterns of three lincRNAs (*HPAT21; HPAT2;* and *HPAT15*) and gene expression signatures (GES) of these *HPAT* expression-defined sub-populations of human blastocyst cells were identified and analyzed. The results of these analyses are shown in the Figs 1-5. All human blastocyst cells are segregated into sub-groups based on common *HPAT*s’ expression patterns (Figs. 1 and 2E). The numbers of cells comprising the corresponding *HPAT*’s expression-defined sub-populations of early (Fig. 2F) and late (Fig. 2G) blastocysts are shown.

Importantly, the overall structure of the *HPAT*’s expression-defined sub-populations remains similar during the transition from the early to late blastocysts (Figs. 1; 2E-2G). The only sub-population that appears to exist exclusively among the early blastocyst cells is represented by the single-positive *HPAT21 ^(-)^ HPAT2 ^(-)^ HPAT15 ^(+)^* cells (Figs. 1B, 2E-2G) comprising a minority of the early blastocyst cell population (in total, 6 cells; 6.8%). Another single-positive sub-population is represented by the ninety-two *HPAT21 ^(+)^ HPAT2 ^(-)^ HPAT15 ^(-)^* cells comprising 37.5% (33 cells) and 38.6% (59 cells) of the early and late blastocyst cell populations, respectively (Figs. 2E-2G). The remaining sub-populations of the both early and late blastocysts are represented by the triple-positive *HPAT21 ^(+)^ HPAT2 ^(+)^ HPAT15 ^(+)^* cells and two distinct double-positive sub-populations of *HPAT21 ^(+)^ HPAT2 ^(+)^ HPAT15 ^(-)^* and *HPAT21 ^(+)^ HPAT2 ^(-)^ HPAT15 ^(+)^* cells (Figs. 1; 2E-2G). Notably, expression patterns of seven TGE-derived lincRNAs appear significantly different in these sub-populations (Fig. 1C) and reliably distinguish three sub-populations: *HPAT21 ^(+)^ HPAT2 ^(+)^ HPAT15 ^(+)^ & HPAT21 ^(+)^ HPAT2 ^(+)^ HPAT15 ^(-)^* cells*; HPAT21 ^(+)^ HPAT2 ^(-)^ HPAT15 ^(-)^* cells; and *HPAT21 ^(+)^ HPAT2 ^(-)^ HPAT15 ^(+)^* cells (Fig. 1). Significantly, the expression of the pluripotency regulatory genes (also defined as the genetic markers of the epiblast-like sub-populations in human blastocysts) appears markedly enriched in the *HPAT21 ^(+)^ HPAT2 ^(+)^ HPAT15 ^(+)^ & HPAT21 ^(+)^ HPAT2 ^(+)^ HPAT15 ^(-)^* cells compared with the *HPAT21 ^(+)^ HPAT2 ^(-)^ HPAT15 ^(-)^* and *HPAT21 ^(+)^ HPAT2 ^(-)^ HPAT15 ^(+)^* cells (Fig. 1D).


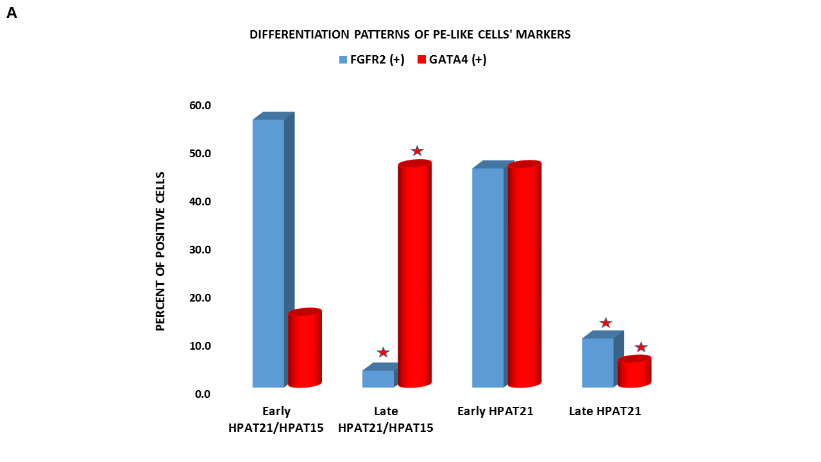

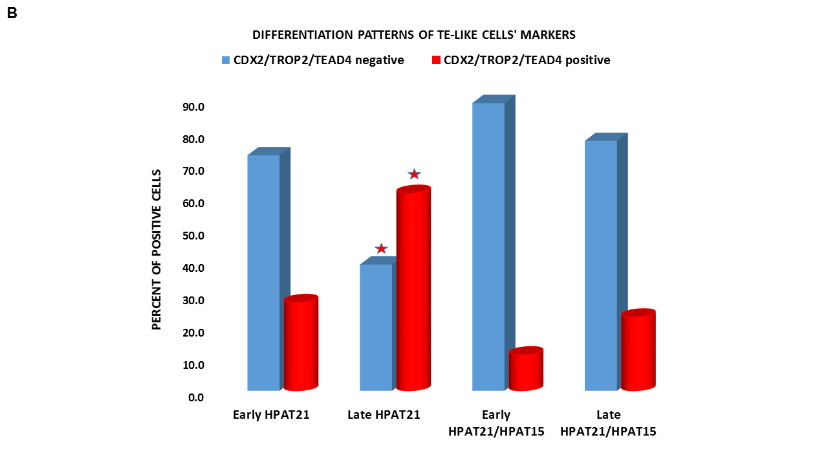

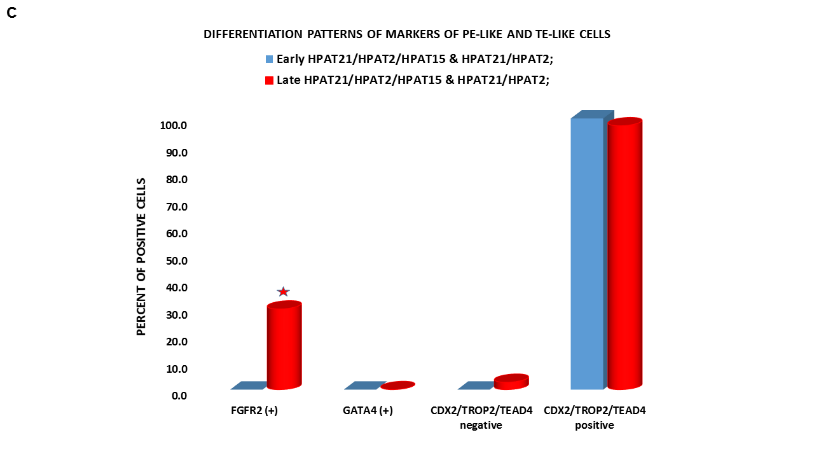

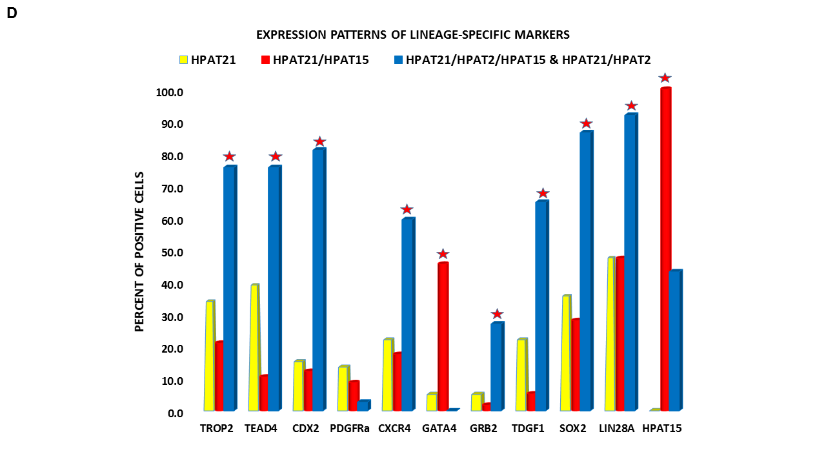

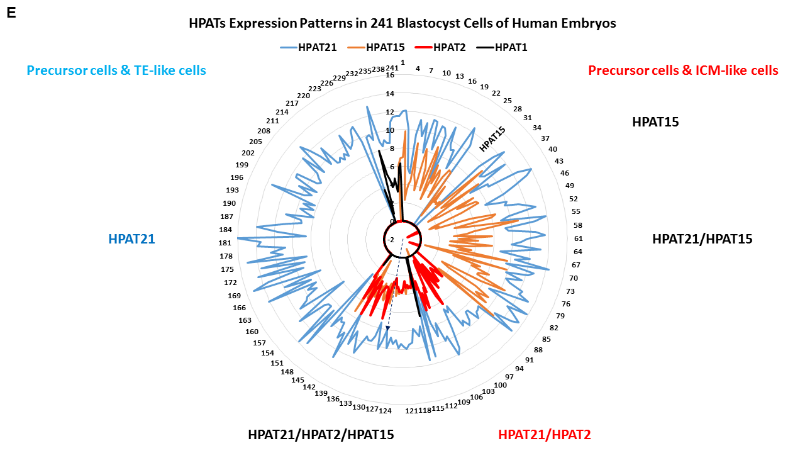

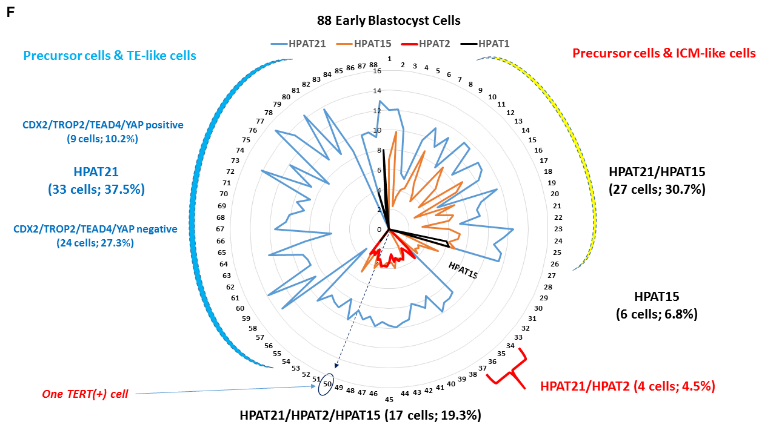

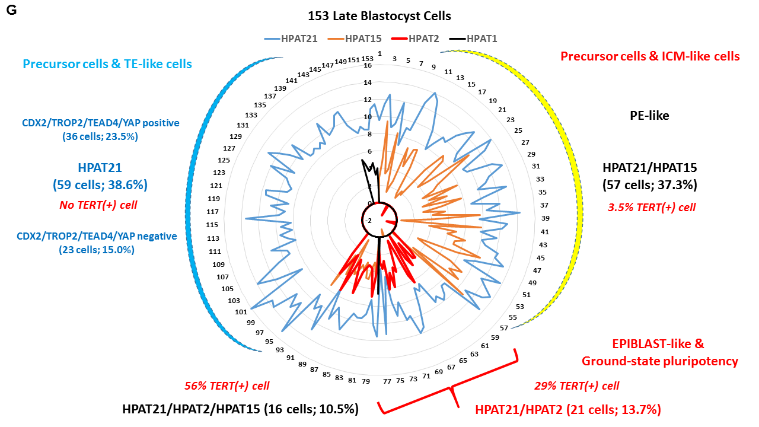


**Figure 2.** **Single-cell analysis of expression patterns of the lineage-specific genetic markers in HPAT lincRNA expression-defined sub-populations during human blastocyst differentiation.**

Expression changes of designated lineage-specific genetic markers (see text for details) were evaluated in individual cells comprising the corresponding HPAT expression-defined sub-populations that were recovered from the early-stage and late-stage human blastocysts. Differentiation patterns in human blastocyst cells of genetic markers of PE-like cells are shown in (A) and (C). Differentiation patterns of genetic markers of TE-like cells are shown in (B). Note that HPAT21 ^(+)^ HPAT15 (+ HPAT2 ^(-)^ cells manifest gene expression differentiation patterns typical of PE-like cells (A), while HPAT21 ^(+)^ HPAT15 ^(-)^ HPAT2 ^(-)^ cells exhibit gene expression differentiation patterns typical of TE-like cells (B). In contrast, no gene expression differentiation patterns resembling either PE-like or TE-like cells were observed in HPAT2 ^(+)^ cell sub-populations (C).

(D) HPAT2 ^(+)^ cell sub-populations manifest significant enrichment of cells expressing genetic markers of all three major lineages created during human blastocyst differentiation. .

(E) HPAT lincRNA expression patterns in 241 individual human blastocyst cells. Each number on the circle designates one randomly placed blastocyst cell.

(F) HPAT lincRNA expression patterns in 88 individual cells recovered from the early-stage human blastocysts. Each number on the circle designates one randomly placed early-stage blastocyst cell.

(G) HPAT lincRNA expression patterns in 153 individual cells recovered from the late-stage human blastocysts. Each number on the circle designates one randomly placed late-stage blastocyst cell.

In the Figs. 2E-G each number on the circle corresponds to a single blastocyst cell. The colored lines inside the circle depict the corresponding *HPAT* lincRNAs and positions of the lines’ heights reflect the *HPAT* lincRNAs’ expression values in corresponding cells. All human blastocyst cells are segregated into sub-groups based on common *HPAT*s’ expression patterns (Fig. 1). The numbers of cells comprising the corresponding *HPAT*’s expression-defined sub-populations of the early (Fig. 2F) and late (Fig. 2G) blastocysts are shown. The statistical significance of the observed expression changes between sub-populations or within a sub-population during differentiation were estimated based on comparisons of the numbers of positive and negative cells using two-tailed Fisher’s exact test. Stars designate sub-populations harboring the significantly different numbers of cells expressing defined genetic markers. P values reported in the Supplemental Table S2.

We focused our subsequent analytical effort (please see main text for details) on characterization of the following three HPAT expression-defined sub-populations of human blastocyst cells:

1. *HPAT21 ^(+)^ HPAT2 ^(-)^ HPAT15 ^(-)^* cells, which were designated for clarity as the single-positive *HPAT21* cells (*spHPAT21*);
2. *HPAT21 ^(+)^ HPAT2 ^(-)^ HPAT15 ^(+)^* cells, which were designated for clarity as the double-positive *HPAT15* cells (*dpHPAT15*);
3. *HPAT21 ^(+)^ HPAT2 ^(+)^ HPAT15 ^(-)^* & *HPAT21 ^(+)^ HPAT2 ^(+)^ HPAT15 ^(+)^* cells, which were designated for clarity as the *HPAT2* positive cells (*HPAT2pos*);

**Supplemental Figure S1 (Supplemental Note 1)**


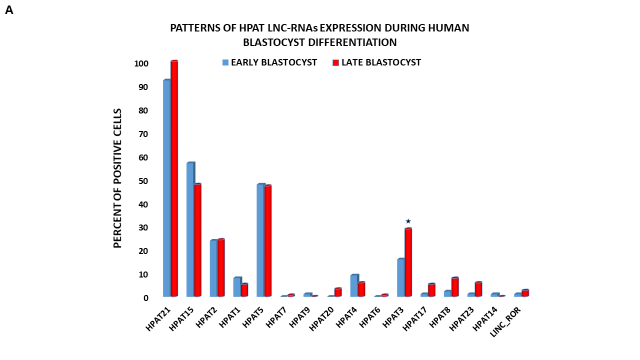


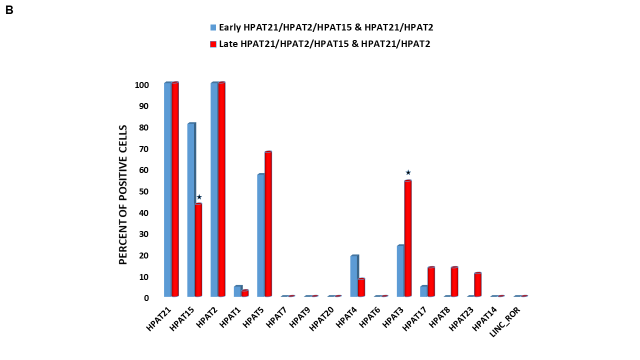

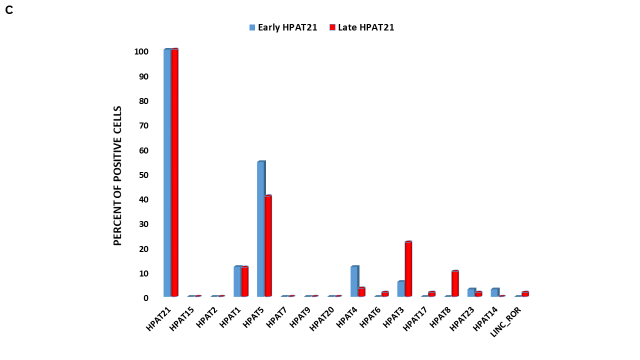

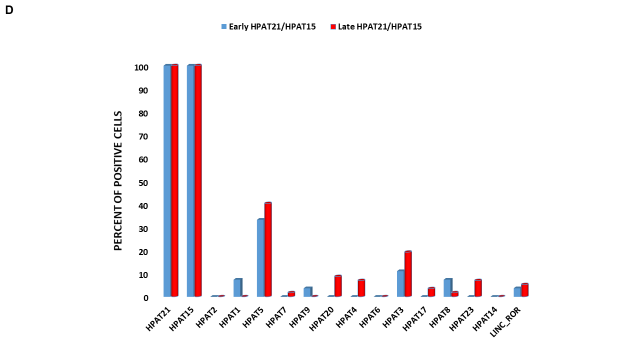


**Supplemental Figure S1 (Supplemental Note 1).** Single cell analysis of expression patterns of HPAT lincRNAs during human blastocyst differentiation. Related to the Figure 1 (Supplemental Note 1).

Expression patterns of HPAT lincRNAs in distinct sub-populations of human blastocyst cells. Stars designate sub-populations harboring the significantly enriched numbers of cells expressing defined genetic markers. P values were estimated using two-tailed Fisher’s exact test and reported in the Supplemental Table S1.
