# Supplementary material for: Single cell expression analysis of primate-specific retroviruses-derived HPAT lincRNAs in viable human blastocysts identifies embryonic cells co-expressing genetic markers of multiple lineages
Source: Heliyon. 2018 Jun 28;4(6):e00667. doi: 10.1016/j.heliyon.2018.e00667 (PMC6039856; doi:10.1016/j.heliyon.2018.e00667)
Supplement: Supplemental Figure S1 [file mmc2.pptx]

## Slide 1
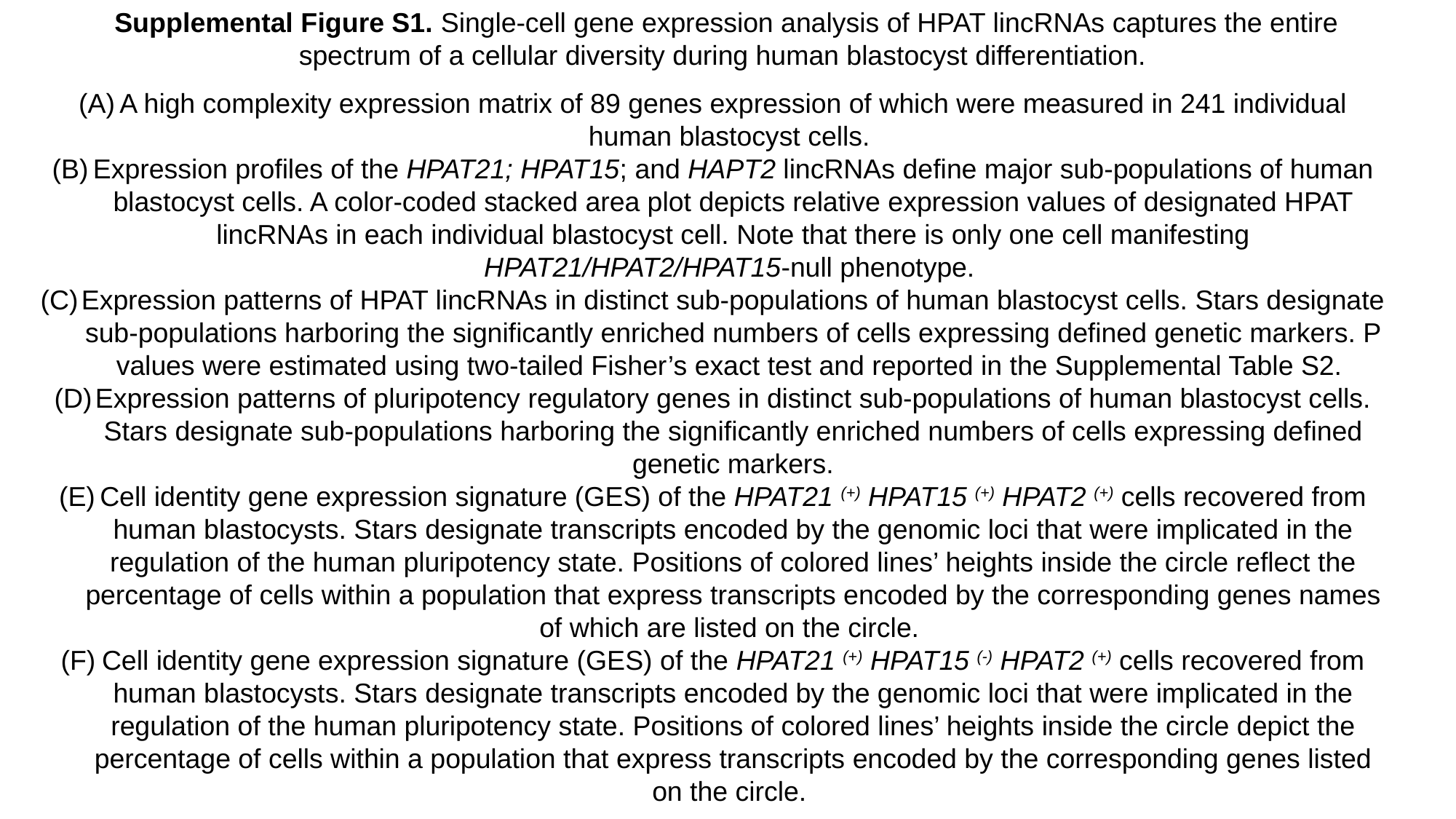

Supplemental Figure S1. Single-cell gene expression analysis of HPAT lincRNAs captures the entire spectrum of a cellular diversity during human blastocyst differentiation.
A high complexity expression matrix of 89 genes expression of which were measured in 241 individual human blastocyst cells.
Expression profiles of the HPAT21; HPAT15; and HAPT2 lincRNAs define major sub-populations of human blastocyst cells. A color-coded stacked area plot depicts relative expression values of designated HPAT lincRNAs in each individual blastocyst cell. Note that there is only one cell manifesting HPAT21/HPAT2/HPAT15-null phenotype.
Expression patterns of HPAT lincRNAs in distinct sub-populations of human blastocyst cells. Stars designate sub-populations harboring the significantly enriched numbers of cells expressing defined genetic markers. P values were estimated using two-tailed Fisher’s exact test and reported in the Supplemental Table S2.
Expression patterns of pluripotency regulatory genes in distinct sub-populations of human blastocyst cells. Stars designate sub-populations harboring the significantly enriched numbers of cells expressing defined genetic markers.
Cell identity gene expression signature (GES) of the HPAT21 (+) HPAT15 (+) HPAT2 (+) cells recovered from human blastocysts. Stars designate transcripts encoded by the genomic loci that were implicated in the regulation of the human pluripotency state. Positions of colored lines’ heights inside the circle reflect the percentage of cells within a population that express transcripts encoded by the corresponding genes names of which are listed on the circle.
Cell identity gene expression signature (GES) of the HPAT21 (+) HPAT15 (-) HPAT2 (+) cells recovered from human blastocysts. Stars designate transcripts encoded by the genomic loci that were implicated in the regulation of the human pluripotency state. Positions of colored lines’ heights inside the circle depict the percentage of cells within a population that express transcripts encoded by the corresponding genes listed on the circle.

## Slide 2
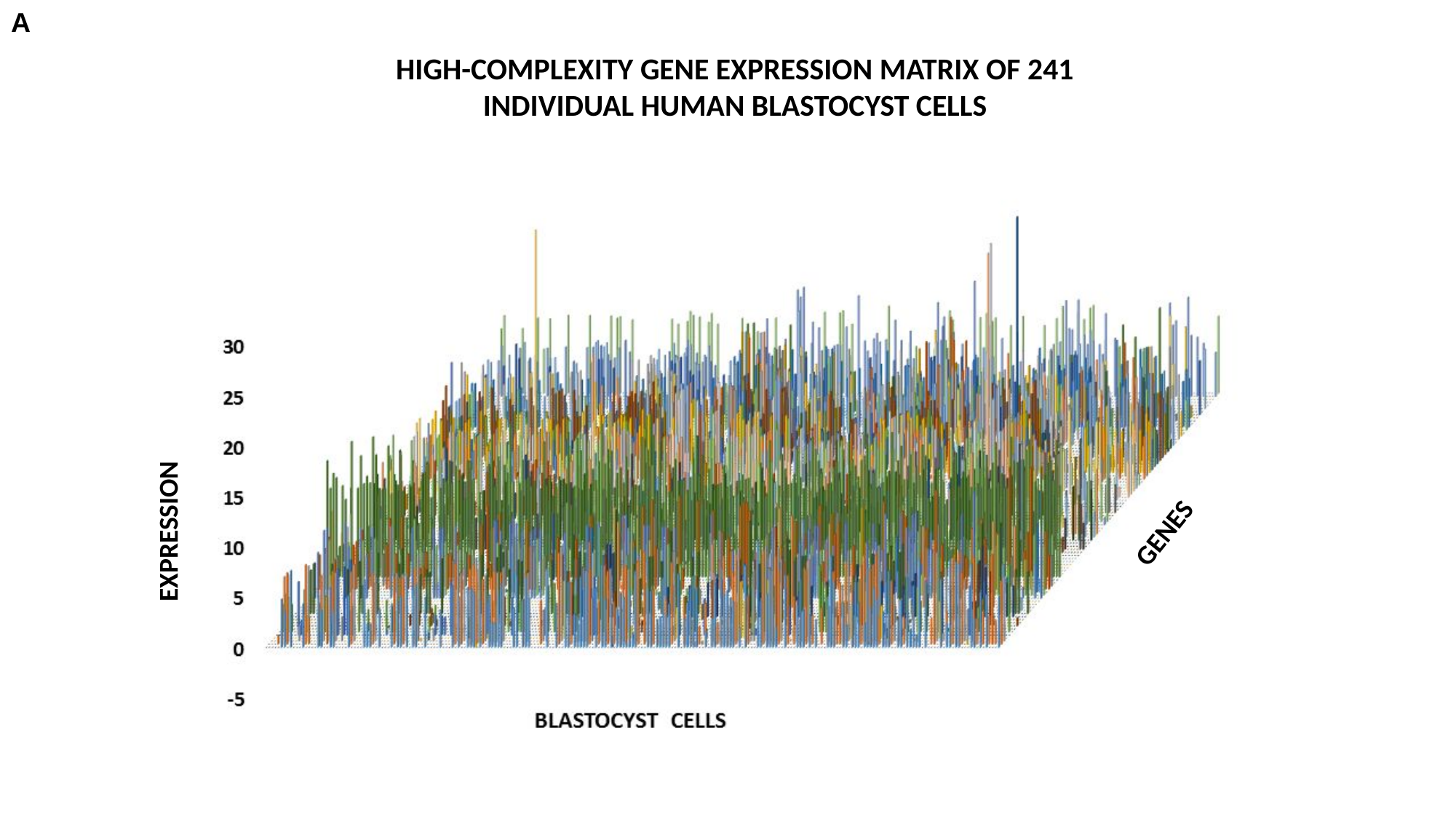

A
HIGH-COMPLEXITY GENE EXPRESSION MATRIX OF 241 INDIVIDUAL HUMAN BLASTOCYST CELLS
EXPRESSION
GENES

## Slide 3
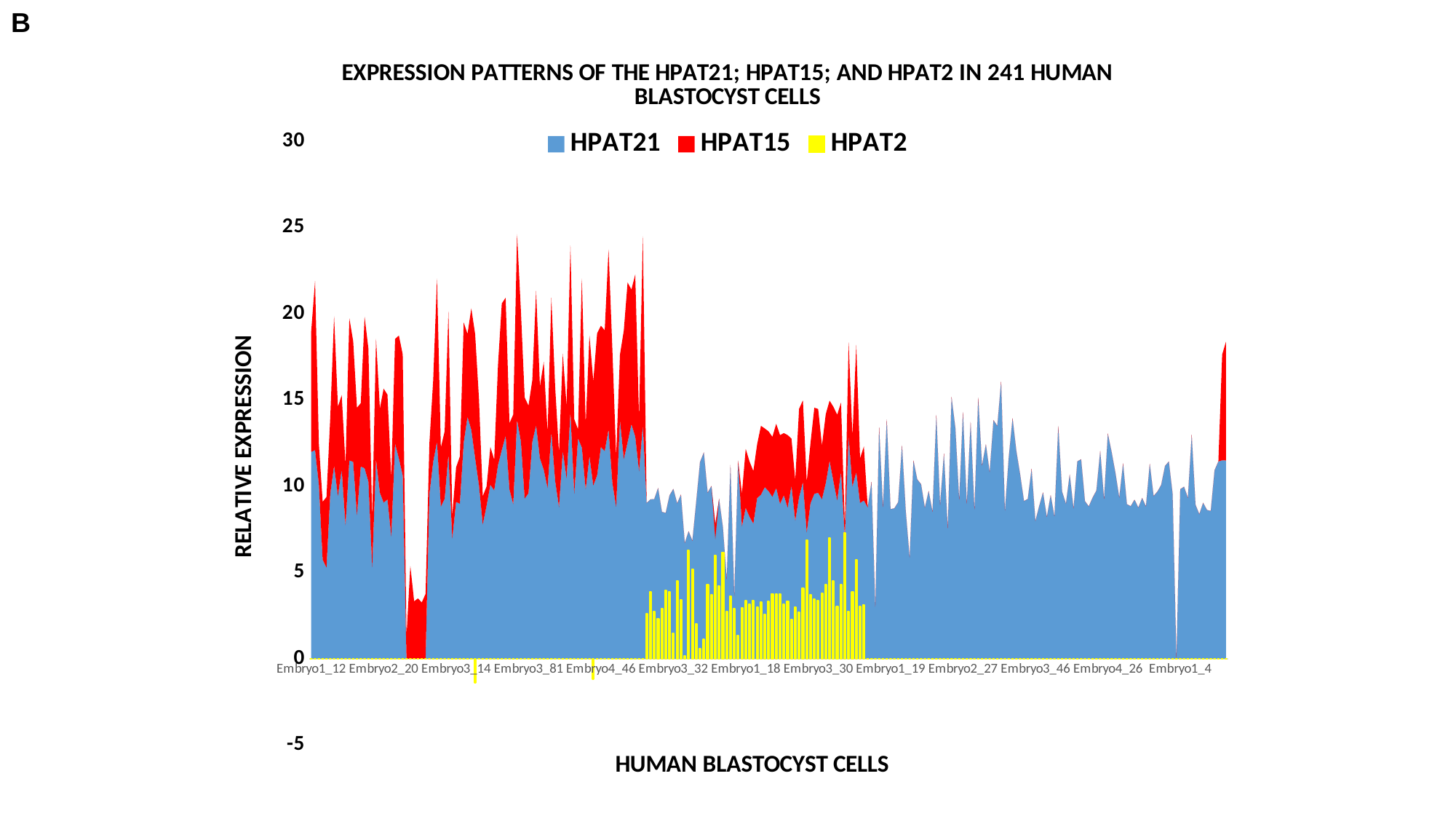

B
### Chart: EXPRESSION PATTERNS OF THE HPAT21; HPAT15; AND HPAT2 IN 241 HUMAN BLASTOCYST CELLS
| Category | HPAT21 | HPAT15 | HPAT2 |
|---|---|---|---|
| Embryo1_12 | 11.99873328 | 6.999644486 | 0.0 |
| Embryo1_17 | 12.09656665 | 9.813847643 | 0.0 |
| Embryo1_23 | 10.13427742 | 2.344604301 | 0.0 |
| Embryo1_30 | 5.754599522 | 3.35245381 | 0.0 |
| Embryo1_31 | 5.287306204 | 4.093157857 | 0.0 |
| Embryo1_32 | 9.574106248 | 4.425182724 | 0.0 |
| Embryo1_34 | 11.1913057 | 8.666960099 | 0.0 |
| Embryo1_40 | 9.44133588 | 5.169612625 | 0.0 |
| Embryo1_43 | 10.97115032 | 4.320395435 | 0.0 |
| Embryo1_46 | 7.742344586 | 3.504687137 | 0.0 |
| Embryo1_47 | 11.50356021 | 8.235206997 | 0.0 |
| Embryo1_48 | 11.41893282 | 6.999644486 | 0.0 |
| Embryo1_51 | 8.329905938 | 6.225676974 | 0.0 |
| Embryo2_1 | 11.14788624 | 3.677020836 | 0.0 |
| Embryo2_6 | 11.04963373 | 8.780766755 | 0.0 |
| Embryo2_7 | 10.27876473 | 7.713667593 | 0.0 |
| Embryo2_10 | 5.315456706 | 2.807745118 | 0.0 |
| Embryo2_12 | 11.5565427 | 6.999644486 | 0.0 |
| Embryo2_13 | 9.638926496 | 4.87472492 | 0.0 |
| Embryo2_20 | 9.058659432 | 6.606763509 | 0.0 |
| Embryo2_23 | 9.241412852 | 6.068600087 | 0.0 |
| Embryo2_28 | 7.109562728 | 3.360837596 | 0.0 |
| Embryo2_31 | 12.48149197 | 6.060982836 | 0.0 |
| Embryo2_33 | 11.61118426 | 7.127579823 | 0.0 |
| Embryo2_35 | 10.6454496 | 6.999644486 | 0.0 |
| Embryo1_8 | 0.0 | 1.320742124 | 0.0 |
| Embryo1_10 | 0.0 | 5.391175979 | 0.0 |
| Embryo1_37 | 0.0 | 3.333144198 | 0.0 |
| Embryo1_50 | 0.0 | 3.504687137 | 0.0 |
| Embryo1_53 | 0.0 | 3.264902478 | 0.0 |
| Embryo2_25 | 0.0 | 3.771597479 | 0.0 |
| Embryo3_1 | 9.558829867 | 2.940964042 | 0.0 |
| Embryo3_2 | 11.41972851 | 4.728409592 | 0.0 |
| Embryo3_5 | 12.59500261 | 9.450451764 | 0.0 |
| Embryo3_7 | 8.826564321 | 3.422435712 | 0.0 |
| Embryo3_10 | 9.27525597 | 3.91838008 | 0.0 |
| Embryo3_11 | 11.86805801 | 8.272559701 | 0.0 |
| Embryo3_12 | 6.971267732 | 1.344515584 | 0.0 |
| Embryo3_14 | 9.092474245 | 2.007855905 | 0.0 |
| Embryo3_15 | 8.99433664 | 2.739357077 | 0.0 |
| Embryo3_18 | 12.52986891 | 6.965362361 | 0.0 |
| Embryo3_20 | 14.03333605 | 4.800599859 | 0.0 |
| Embryo3_22 | 13.27635206 | 7.038680975 | 0.0 |
| Embryo3_27 | 11.63699366 | 7.190724234 | -1.372325551 |
| Embryo3_35 | 10.07597512 | 5.01429947 | 0.0 |
| Embryo3_39 | 7.795049203 | 1.637225178 | 0.0 |
| Embryo3_44 | 8.869564511 | 1.119289369 | 0.0 |
| Embryo3_49 | 10.09043319 | 2.193679317 | 0.0 |
| Embryo3_51 | 9.765475655 | 1.785428457 | 0.0 |
| Embryo3_55 | 11.22844835 | 5.797896165 | 0.0 |
| Embryo3_62 | 12.10672969 | 8.493335314 | 0.0 |
| Embryo3_67 | 12.94679892 | 7.984709387 | 0.0 |
| Embryo3_68 | 9.825124493 | 3.834658106 | 0.0 |
| Embryo3_70 | 8.961381358 | 5.200561061 | 0.0 |
| Embryo3_72 | 13.85670767 | 10.75884697 | 0.0 |
| Embryo3_73 | 12.60755625 | 7.57836944 | 0.0 |
| Embryo3_78 | 9.293311204 | 5.854900215 | 0.0 |
| Embryo3_81 | 9.600917569 | 5.075978964 | 0.0 |
| Embryo3_82 | 12.57935156 | 3.575442967 | 0.0 |
| Embryo4_2 | 13.5442982 | 7.793378649 | 0.0 |
| Embryo4_3 | 11.62852467 | 4.172761118 | 0.0 |
| Embryo4_4 | 10.9684515 | 6.249133772 | 0.0 |
| Embryo4_5 | 9.906762252 | 3.205029641 | 0.0 |
| Embryo4_8 | 13.14952558 | 7.785659508 | 0.0 |
| Embryo4_10 | 10.30622556 | 5.437470883 | 0.0 |
| Embryo4_11 | 8.794763869 | 3.156163892 | 0.0 |
| Embryo4_14 | 12.06291432 | 5.633609591 | 0.0 |
| Embryo4_15 | 10.46334108 | 4.201812648 | 0.0 |
| Embryo4_16 | 14.29852828 | 9.665617414 | 0.0 |
| Embryo4_21 | 9.573035261 | 4.325882086 | 0.0 |
| Embryo4_32 | 12.78332699 | 0.51203456 | 0.0 |
| Embryo4_37 | 12.24670217 | 9.803165699 | 0.0 |
| Embryo4_39 | 9.818480447 | 3.796978022 | 0.0 |
| Embryo4_40 | 11.74037174 | 6.999644486 | 0.0 |
| Embryo4_41 | 10.0473369 | 6.044426253 | -1.161014903 |
| Embryo4_43 | 10.64002268 | 8.225792389 | 0.0 |
| Embryo4_46 | 12.27984078 | 7.031826214 | 0.0 |
| Embryo4_47 | 12.04419204 | 6.999644486 | 0.0 |
| Embryo4_50 | 13.29778217 | 10.43692477 | 0.0 |
| Embryo4_52 | 10.20815657 | 7.7112409 | 0.0 |
| Embryo4_54 | 8.818821763 | 2.929659754 | 0.0 |
| Embryo4_55 | 13.81619759 | 3.823960209 | 0.0 |
| Embryo4_59 | 11.57953383 | 7.449746331 | 0.0 |
| Embryo4_60 | 12.54647313 | 9.268253195 | 0.0 |
| Embryo4_61 | 13.59995793 | 7.806672512 | 0.0 |
| Embryo4_62 | 12.89354855 | 9.380393292 | 0.0 |
| Embryo4_65 | 10.87578243 | 3.049702529 | 0.0 |
| Embryo4_70 | 13.53578914 | 10.95931928 | 0.0 |
| Embryo1_6 | 9.033064504 | 0.0 | 2.634594948 |
| Embryo1_13 | 9.245223238 | 0.0 | 3.885442059 |
| Embryo2_11 | 9.260489458 | 0.0 | 2.731501793 |
| Embryo2_24 | 9.907077152 | 0.0 | 2.324282882 |
| Embryo3_21 | 8.521000634 | 0.0 | 2.906737711 |
| Embryo3_26 | 8.458184215 | 0.0 | 3.982059036 |
| Embryo3_28 | 9.474693441 | 0.0 | 3.891950834 |
| Embryo3_32 | 9.853790123 | 0.0 | 1.472060372 |
| Embryo3_45 | 9.015141933 | 0.0 | 4.511253463 |
| Embryo3_48 | 9.518614742 | 0.0 | 3.40292233 |
| Embryo3_52 | 6.71537474 | 0.0 | 0.167107029 |
| Embryo3_54 | 7.398356747 | 0.0 | 6.285010357 |
| Embryo3_60 | 6.841912395 | 0.0 | 5.178095773 |
| Embryo3_64 | 9.10593594 | 0.0 | 2.043617438 |
| Embryo3_80 | 11.41001146 | 0.0 | 0.593419375 |
| Embryo4_7 | 11.96716417 | 0.0 | 1.116580112 |
| Embryo4_20 | 9.630849802 | 0.0 | 4.284310208 |
| Embryo4_25 | 10.02052555 | 0.0 | 3.725418455 |
| Embryo4_28 | 7.79438844 | -0.869131379 | 6.001215206 |
| Embryo4_29 | 9.274552856 | 0.0 | 4.227446748 |
| Embryo4_34 | 7.635718345 | 0.0 | 6.148614006 |
| Embryo4_35 | 4.615115977 | 0.0 | 2.75225298 |
| Embryo4_49 | 11.2459727 | 0.0 | 3.64255185 |
| Embryo4_58 | 3.653584127 | 0.0 | 2.925609168 |
| Embryo4_18 | 11.49217643 | 0.0 | 1.35850542 |
| Embryo1_7 | 7.678497736 | 1.910425554 | 2.957976007 |
| Embryo1_18 | 8.751339296 | 3.419642754 | 3.355331988 |
| Embryo1_22 | 8.239929371 | 3.199008789 | 3.168324941 |
| Embryo1_35 | 7.842115889 | 3.044451098 | 3.384836229 |
| Embryo1_42 | 9.320875354 | 3.121824864 | 2.975747455 |
| Embryo1_44 | 9.517481307 | 3.989568765 | 3.292409123 |
| Embryo1_45 | 9.949993194 | 3.40232397 | 2.576126822 |
| Embryo1_49 | 9.717921588 | 3.465217243 | 3.319104948 |
| Embryo2_9 | 9.396081376 | 3.471785474 | 3.775259341 |
| Embryo2_14 | 9.901740887 | 3.723916017 | 3.743173927 |
| Embryo2_15 | 8.986121946 | 3.97932822 | 3.772361413 |
| Embryo2_16 | 9.477008574 | 3.610382099 | 3.171370682 |
| Embryo2_18 | 8.763619981 | 4.207914506 | 3.332566483 |
| Embryo2_21 | 10.03357658 | 2.732681683 | 2.287046579 |
| Embryo2_22 | 8.007339926 | 2.369931054 | 2.984202125 |
| Embryo2_32 | 9.371799334 | 5.118904173 | 2.696931444 |
| Embryo3_8 | 10.24437818 | 4.739330053 | 4.102886678 |
| Embryo3_23 | 7.324374079 | 2.934852552 | 6.890472033 |
| Embryo3_24 | 9.015067783 | 3.499062144 | 3.702736267 |
| Embryo3_29 | 9.570352278 | 4.989173683 | 3.453241424 |
| Embryo3_30 | 9.622109205 | 4.862469888 | 3.362820726 |
| Embryo3_65 | 9.25429144 | 3.117314401 | 3.805301144 |
| Embryo3_66 | 10.18147076 | 4.004128035 | 4.320395435 |
| Embryo3_74 | 11.4703229 | 3.497247353 | 6.993741077 |
| Embryo3_75 | 10.30474567 | 4.30859141 | 4.502968081 |
| Embryo3_77 | 9.187041144 | 4.962131502 | 3.05046107 |
| Embryo3_79 | 10.83800592 | 4.013012984 | 4.320395435 |
| Embryo4_1 | 7.054833379 | 0.648403971 | 7.28101729 |
| Embryo4_19 | 12.92020327 | 5.428218911 | 2.744698662 |
| Embryo4_30 | 9.993002739 | 2.919410882 | 3.890562912 |
| Embryo4_45 | 10.82369712 | 7.378965361 | 5.729183087 |
| Embryo4_71 | 9.038490771 | 2.586774112 | 3.022672268 |
| Embryo1_28 | 9.177809734 | 3.130119372 | 3.129121409 |
| Embryo1_2 | 8.787934638 | 0.0 | 0.0 |
| Embryo1_3 | 10.25437555 | 0.0 | 0.0 |
| Embryo1_5 | 3.005732898 | 0.0 | 0.0 |
| Embryo1_9 | 13.42242129 | 0.0 | 0.0 |
| Embryo1_11 | 8.799476777 | 0.0 | 0.0 |
| Embryo1_16 | 13.87465034 | 0.0 | 0.0 |
| Embryo1_19 | 8.674295391 | 0.0 | 0.0 |
| Embryo1_20 | 8.730354358 | 0.0 | 0.0 |
| Embryo1_24 | 9.083564239 | 0.0 | 0.0 |
| Embryo1_25 | 12.38362599 | 0.0 | 0.0 |
| Embryo1_26 | 8.524745249 | 0.0 | 0.0 |
| Embryo1_27 | 5.866091498 | 0.0 | 0.0 |
| Embryo1_29 | 11.50230284 | 0.0 | 0.0 |
| Embryo1_33 | 10.40543704 | 0.0 | 0.0 |
| Embryo1_36 | 10.12186759 | 0.0 | 0.0 |
| Embryo1_38 | 8.764851705 | 0.0 | 0.0 |
| Embryo1_39 | 9.725631945 | 0.0 | 0.0 |
| Embryo1_52 | 8.522288066 | 0.0 | 0.0 |
| Embryo2_2 | 14.14588307 | 0.0 | 0.0 |
| Embryo2_3 | 8.934945526 | 0.0 | 0.0 |
| Embryo2_4 | 11.89505561 | 0.0 | 0.0 |
| Embryo2_5 | 7.569917726 | 0.0 | 0.0 |
| Embryo2_8 | 15.1767082 | 0.0 | 0.0 |
| Embryo2_19 | 13.38243367 | 0.0 | 0.0 |
| Embryo2_26 | 9.235763203 | 0.0 | 0.0 |
| Embryo2_27 | 14.31483726 | 0.0 | 0.0 |
| Embryo2_29 | 8.983659342 | 0.0 | 0.0 |
| Embryo2_30 | 13.73097211 | 0.0 | 0.0 |
| Embryo2_34 | 8.659942056 | 0.0 | 0.0 |
| Embryo3_3 | 15.15021438 | 0.0 | 0.0 |
| Embryo3_4 | 11.2064789 | 0.0 | 0.0 |
| Embryo3_6 | 12.4402782 | 0.0 | 0.0 |
| Embryo3_9 | 10.82324621 | 0.0 | 0.0 |
| Embryo3_16 | 13.83920966 | 0.0 | 0.0 |
| Embryo3_17 | 13.49463211 | 0.0 | 0.0 |
| Embryo3_19 | 16.0872559 | 0.0 | 0.0 |
| Embryo3_31 | 8.581479683 | 0.0 | 0.0 |
| Embryo3_33 | 11.66762662 | 0.0 | 0.0 |
| Embryo3_34 | 13.95157394 | 0.0 | 0.0 |
| Embryo3_36 | 12.05683786 | 0.0 | 0.0 |
| Embryo3_38 | 10.6800382 | 0.0 | 0.0 |
| Embryo3_41 | 9.144882707 | 0.0 | 0.0 |
| Embryo3_42 | 9.267585143 | 0.0 | 0.0 |
| Embryo3_43 | 11.01545707 | 0.0 | 0.0 |
| Embryo3_46 | 7.995355818 | 0.0 | 0.0 |
| Embryo3_47 | 8.865137094 | 0.0 | 0.0 |
| Embryo3_50 | 9.649937139 | 0.0 | 0.0 |
| Embryo3_53 | 8.224094333 | 0.0 | 0.0 |
| Embryo3_56 | 9.493432842 | 0.0 | 0.0 |
| Embryo3_57 | 8.25688029 | 0.0 | 0.0 |
| Embryo3_58 | 13.49369866 | 0.0 | 0.0 |
| Embryo3_59 | 9.688199255 | 0.0 | 0.0 |
| Embryo3_61 | 8.994670352 | 0.0 | 0.0 |
| Embryo3_63 | 10.69319989 | 0.0 | 0.0 |
| Embryo3_69 | 8.713738436 | 0.0 | 0.0 |
| Embryo3_71 | 11.45222818 | 0.0 | 0.0 |
| Embryo4_6 | 11.5651113 | 0.0 | 0.0 |
| Embryo4_9 | 9.138396132 | 0.0 | 0.0 |
| Embryo4_12 | 8.838619591 | 0.0 | 0.0 |
| Embryo4_13 | 9.34092825 | 0.0 | 0.0 |
| Embryo4_17 | 9.754007655 | 0.0 | 0.0 |
| Embryo4_22 | 12.05908817 | 0.0 | 0.0 |
| Embryo4_23 | 9.265759641 | 0.0 | 0.0 |
| Embryo4_26 | 13.0551395 | 0.0 | 0.0 |
| Embryo4_27 | 11.97987575 | 0.0 | 0.0 |
| Embryo4_31 | 10.76630201 | 0.0 | 0.0 |
| Embryo4_33 | 9.352218139 | 0.0 | 0.0 |
| Embryo4_36 | 11.33146695 | 0.0 | 0.0 |
| Embryo4_38 | 8.966346629 | 0.0 | 0.0 |
| Embryo4_42 | 8.848573294 | 0.0 | 0.0 |
| Embryo4_44 | 9.224533756 | 0.0 | 0.0 |
| Embryo4_48 | 8.765777742 | 0.0 | 0.0 |
| Embryo4_51 | 9.32539489 | 0.0 | 0.0 |
| Embryo4_53 | 8.839379363 | 0.0 | 0.0 |
| Embryo4_57 | 11.31452889 | 0.0 | 0.0 |
| Embryo4_63 | 9.436737908 | 0.0 | 0.0 |
| Embryo4_64 | 9.690996429 | 0.0 | 0.0 |
| Embryo4_66 | 10.07995235 | 0.0 | 0.0 |
| Embryo4_67 | 11.17803099 | 0.0 | 0.0 |
| Embryo4_68 | 11.44363475 | 0.0 | 0.0 |
| Embryo4_69 | 9.554988975 | 0.0 | 0.0 |
| Embryo2_17 | 0.0 | 0.0 | 0.0 |
| Embryo1_4 | 9.832756611 | 0.0 | 0.0 |
| Embryo1_14 | 9.977625103 | 0.0 | 0.0 |
| Embryo1_15 | 9.335942869 | 0.0 | 0.0 |
| Embryo1_21 | 12.98731777 | 0.0 | 0.0 |
| Embryo3_13 | 8.930659061 | 0.0 | 0.0 |
| Embryo3_25 | 8.38549196 | 0.0 | 0.0 |
| Embryo3_37 | 9.043235561 | 0.0 | 0.0 |
| Embryo3_40 | 8.63216944 | 0.0 | 0.0 |
| Embryo3_76 | 8.573426229 | 0.0 | 0.0 |
| Embryo4_24 | 10.92942806 | 0.0 | 0.0 |
| Embryo4_56 | 11.44340375 | 0.0 | 0.0 |
| Embryo1_1 | 11.50952022 | 6.131324157 | 0.0 |
| Embryo1_41 | 11.49715307 | 6.888494201 | 0.0 |

## Slide 4
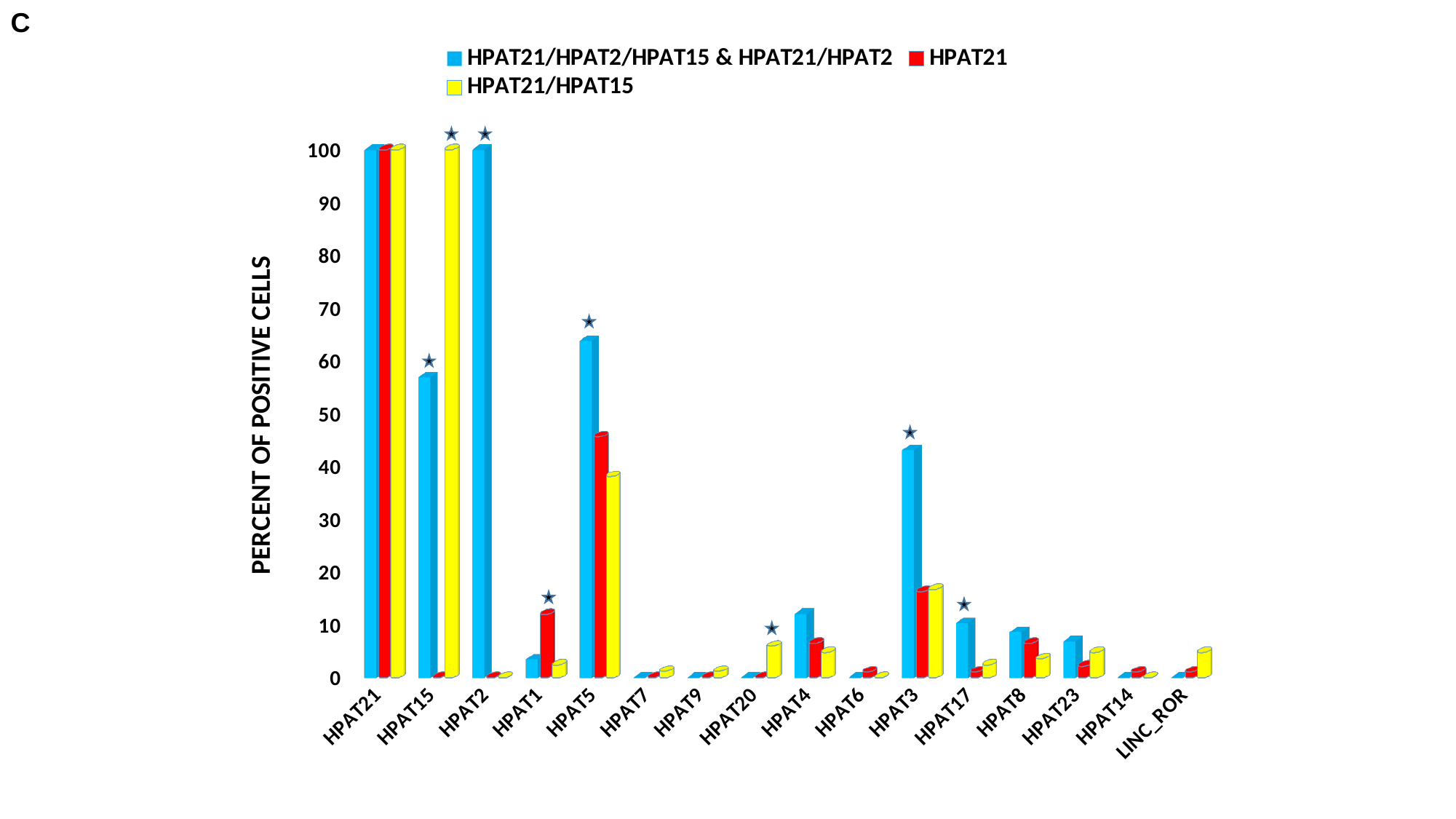

C
[unsupported chart]

## Slide 5
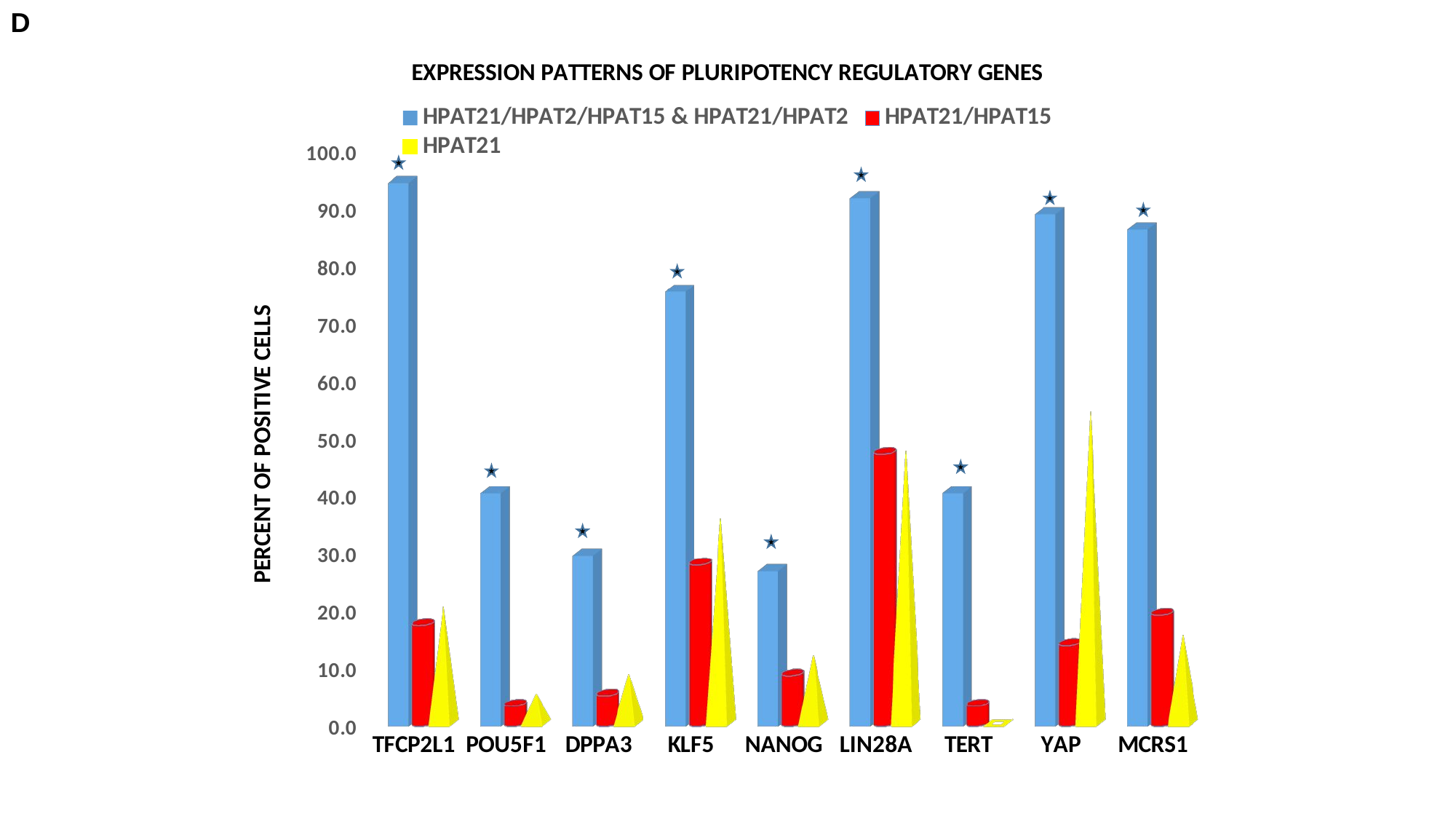

D
[unsupported chart]

## Slide 6
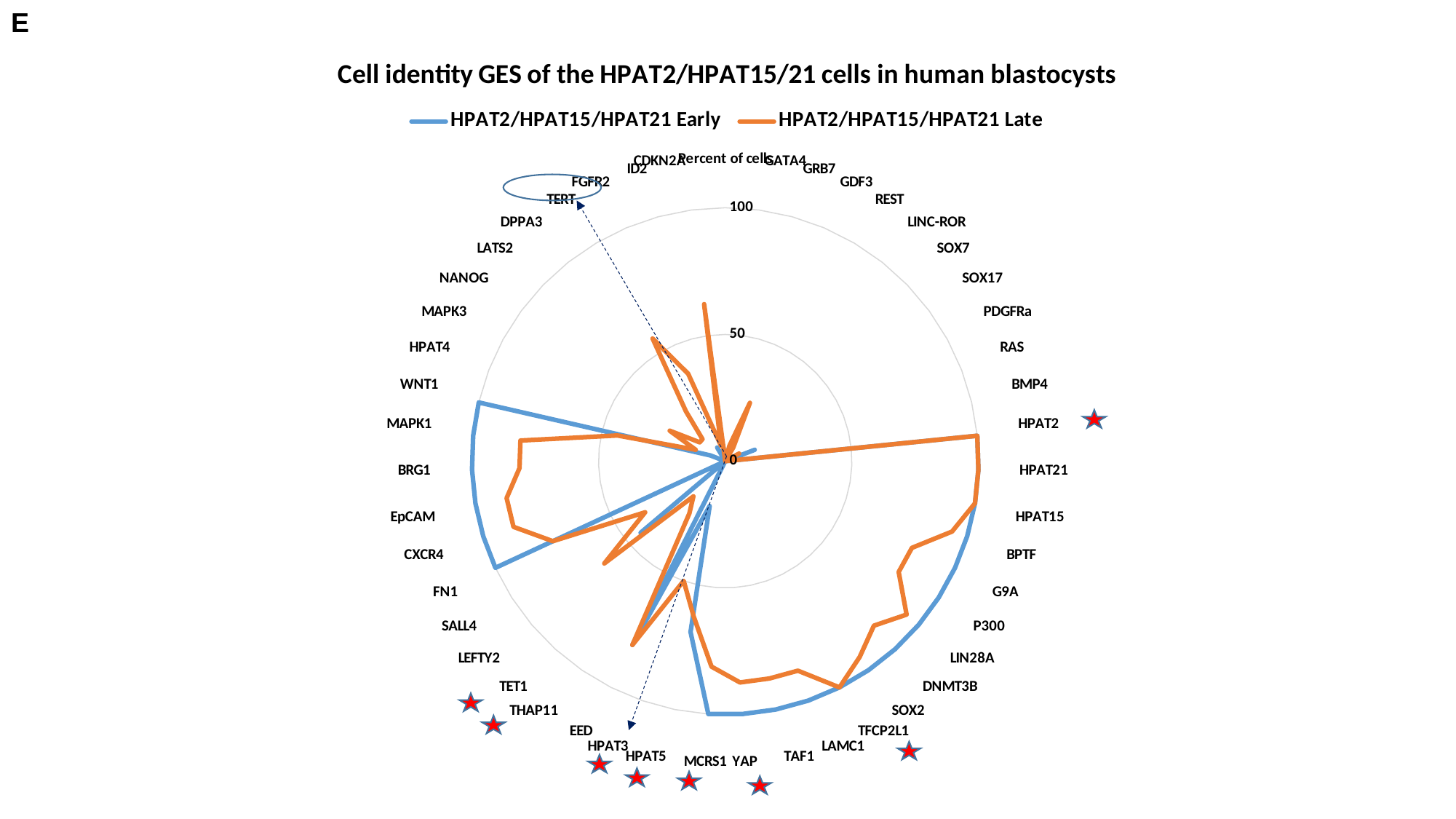

E
### Chart: Cell identity GES of the HPAT2/HPAT15/21 cells in human blastocysts
| Category | HPAT2/HPAT15/HPAT21 | HPAT2/HPAT15/HPAT21 |
|---|---|---|
| Percent of cells | 0.0 | 0.0 |
| GATA4 | 0.0 | 0.0 |
| GRB7 | 0.0 | 6.25 |
| GDF3 | 0.0 | 25.0 |
| REST | 0.0 | 6.25 |
| LINC-ROR | 0.0 | 0.0 |
| SOX7 | 0.0 | 0.0 |
| SOX17 | 0.0 | 0.0 |
| PDGFRa | 0.0 | 6.25 |
| RAS | 12.5 | 0.0 |
| BMP4 | 0.0 | 0.0 |
| HPAT2 | 100.0 | 100.0 |
| HPAT21 | 100.0 | 100.0 |
| HPAT15 | 100.0 | 100.0 |
| BPTF | 100.0 | 93.75 |
| G9A | 100.0 | 81.25 |
| P300 | 100.0 | 81.25 |
| LIN28A | 100.0 | 93.75 |
| DNMT3B | 100.0 | 87.5 |
| SOX2 | 100.0 | 93.75 |
| TFCP2L1 | 100.0 | 100.0 |
| LAMC1 | 100.0 | 87.5 |
| TAF1 | 100.0 | 87.5 |
| YAP | 100.0 | 87.5 |
| MCRS1 | 100.0 | 81.25 |
| HPAT5 | 68.75 | 62.5 |
| HPAT3 | 18.75 | 50.0 |
| EED | 81.25 | 81.25 |
| THAP11 | 0.0 | 25.0 |
| TET1 | 0.0 | 18.75 |
| LEFTY2 | 43.75 | 62.5 |
| SALL4 | 0.0 | 37.5 |
| FN1 | 100.0 | 75.0 |
| CXCR4 | 100.0 | 87.5 |
| EpCAM | 100.0 | 87.5 |
| BRG1 | 100.0 | 81.25 |
| MAPK1 | 100.0 | 81.25 |
| WNT1 | 100.0 | 43.75 |
| HPAT4 | 6.25 | 12.5 |
| MAPK3 | 0.0 | 25.0 |
| NANOG | 0.0 | 12.5 |
| LATS2 | 0.0 | 12.5 |
| DPPA3 | 0.0 | 25.0 |
| TERT | 6.25 | 56.25 |
| FGFR2 | 0.0 | 37.5 |
| ID2 | 0.0 | 6.25 |
| CDKN2A | 6.25 | 62.5 |

## Slide 7
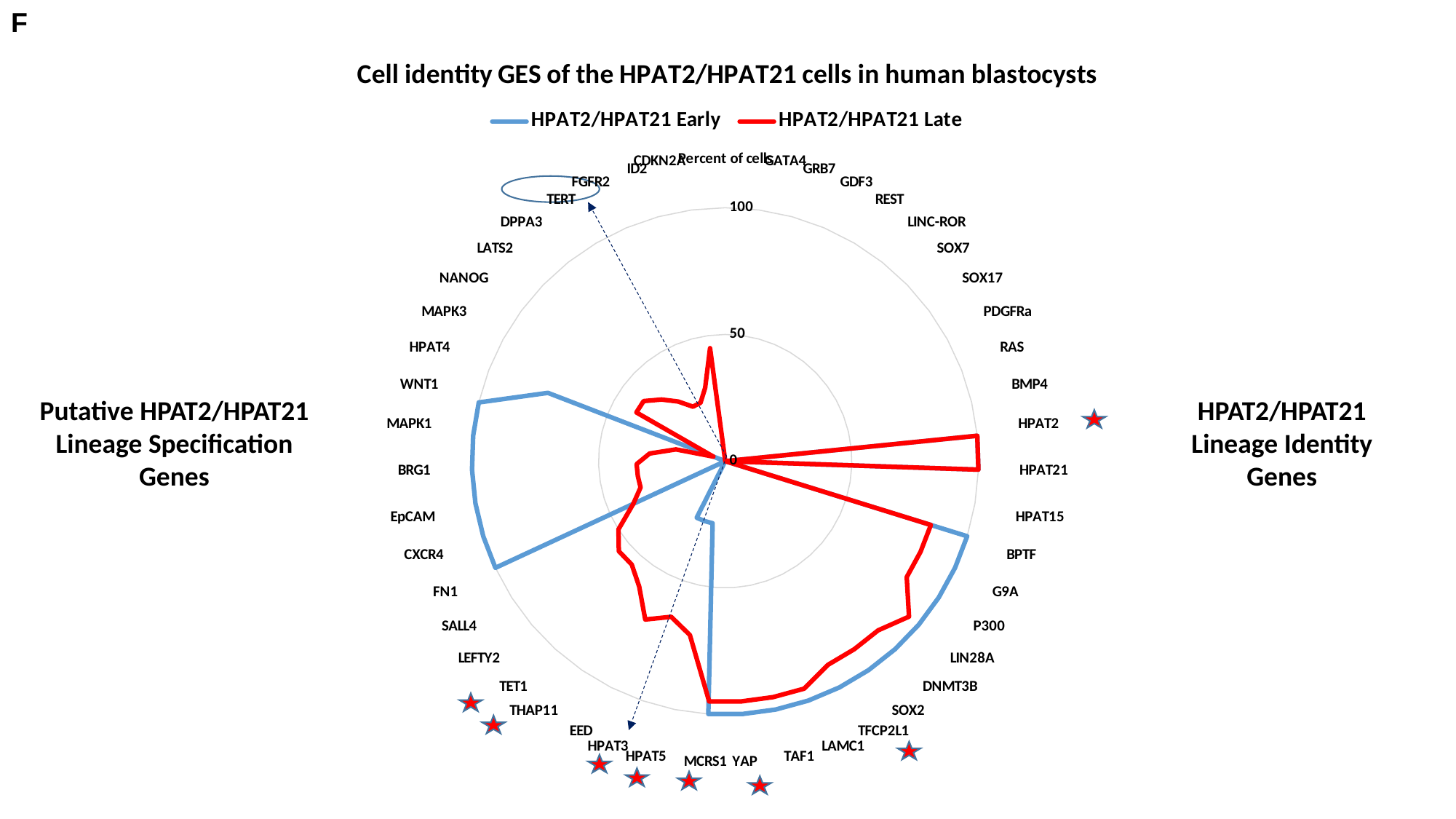

F
### Chart: Cell identity GES of the HPAT2/HPAT21 cells in human blastocysts
| Category | HPAT2/HPAT21 | HPAT2/HPAT21 |
|---|---|---|
| Percent of cells | 0.0 | 0.0 |
| GATA4 | 0.0 | 0.0 |
| GRB7 | 0.0 | 0.0 |
| GDF3 | 0.0 | 0.0 |
| REST | 0.0 | 0.0 |
| LINC-ROR | 0.0 | 0.0 |
| SOX7 | 0.0 | 0.0 |
| SOX17 | 0.0 | 0.0 |
| PDGFRa | 0.0 | 0.0 |
| RAS | 0.0 | 0.0 |
| BMP4 | 0.0 | 0.0 |
| HPAT2 | 100.0 | 100.0 |
| HPAT21 | 100.0 | 100.0 |
| HPAT15 | 0.0 | 0.0 |
| BPTF | 100.0 | 85.0 |
| G9A | 100.0 | 85.0 |
| P300 | 100.0 | 85.0 |
| LIN28A | 100.0 | 95.0 |
| DNMT3B | 100.0 | 90.0 |
| SOX2 | 100.0 | 90.0 |
| TFCP2L1 | 100.0 | 90.0 |
| LAMC1 | 100.0 | 95.0 |
| TAF1 | 100.0 | 95.0 |
| YAP | 100.0 | 95.0 |
| MCRS1 | 100.0 | 95.0 |
| HPAT5 | 25.0 | 70.0 |
| HPAT3 | 25.0 | 65.0 |
| EED | 25.0 | 70.0 |
| THAP11 | 0.0 | 60.0 |
| TET1 | 0.0 | 55.00000000000001 |
| LEFTY2 | 0.0 | 55.00000000000001 |
| SALL4 | 0.0 | 50.0 |
| FN1 | 100.0 | 40.0 |
| CXCR4 | 100.0 | 35.0 |
| EpCAM | 100.0 | 35.0 |
| BRG1 | 100.0 | 35.0 |
| MAPK1 | 100.0 | 30.0 |
| WNT1 | 100.0 | 20.0 |
| HPAT4 | 75.0 | 5.0 |
| MAPK3 | 0.0 | 40.0 |
| NANOG | 0.0 | 40.0 |
| LATS2 | 0.0 | 35.0 |
| DPPA3 | 0.0 | 30.0 |
| TERT | 0.0 | 25.0 |
| FGFR2 | 0.0 | 25.0 |
| ID2 | 0.0 | 30.0 |
| CDKN2A | 0.0 | 45.0 |
Putative HPAT2/HPAT21
Lineage Specification Genes
HPAT2/HPAT21
Lineage Identity Genes
