# Supplementary material for: Single cell expression analysis of primate-specific retroviruses-derived HPAT lincRNAs in viable human blastocysts identifies embryonic cells co-expressing genetic markers of multiple lineages
Source: Heliyon. 2018 Jun 28;4(6):e00667. doi: 10.1016/j.heliyon.2018.e00667 (PMC6039856; doi:10.1016/j.heliyon.2018.e00667)
Supplement: Supplemental Figure S1.1 [file mmc3.pptx]

## Slide 1
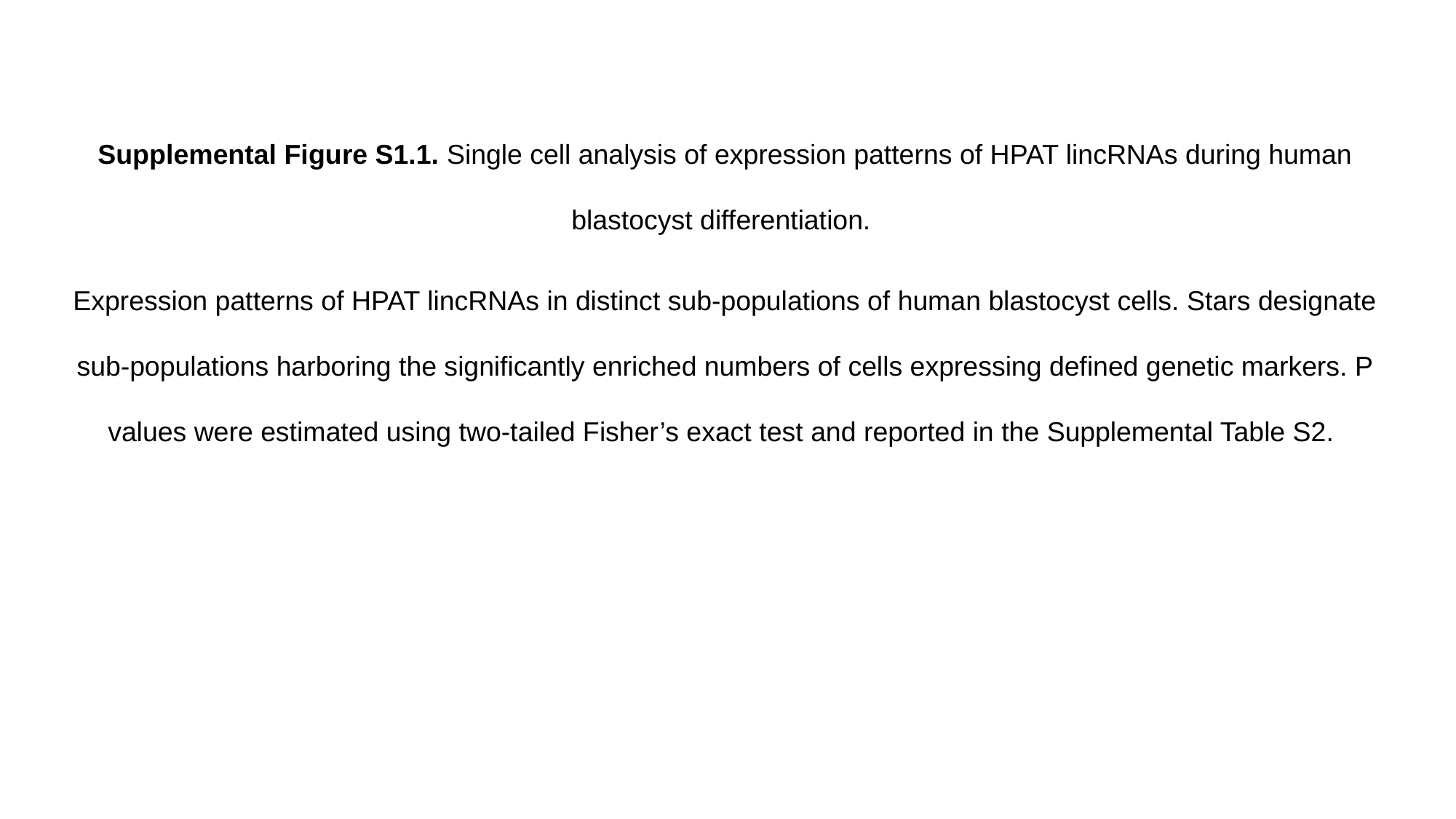

Supplemental Figure S1.1. Single cell analysis of expression patterns of HPAT lincRNAs during human blastocyst differentiation.
Expression patterns of HPAT lincRNAs in distinct sub-populations of human blastocyst cells. Stars designate sub-populations harboring the significantly enriched numbers of cells expressing defined genetic markers. P values were estimated using two-tailed Fisher’s exact test and reported in the Supplemental Table S2.

## Slide 2
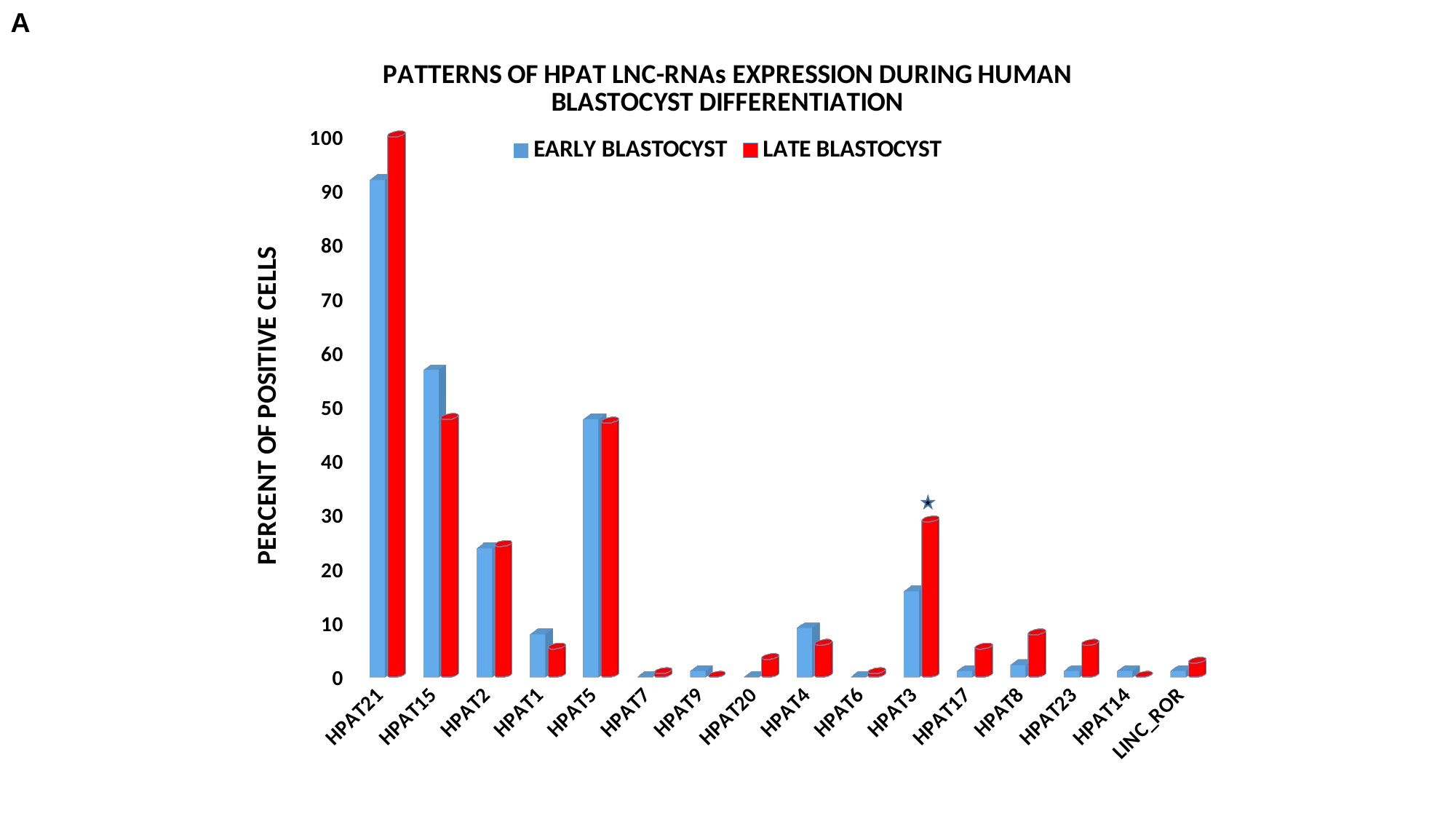

A
[unsupported chart]

## Slide 3
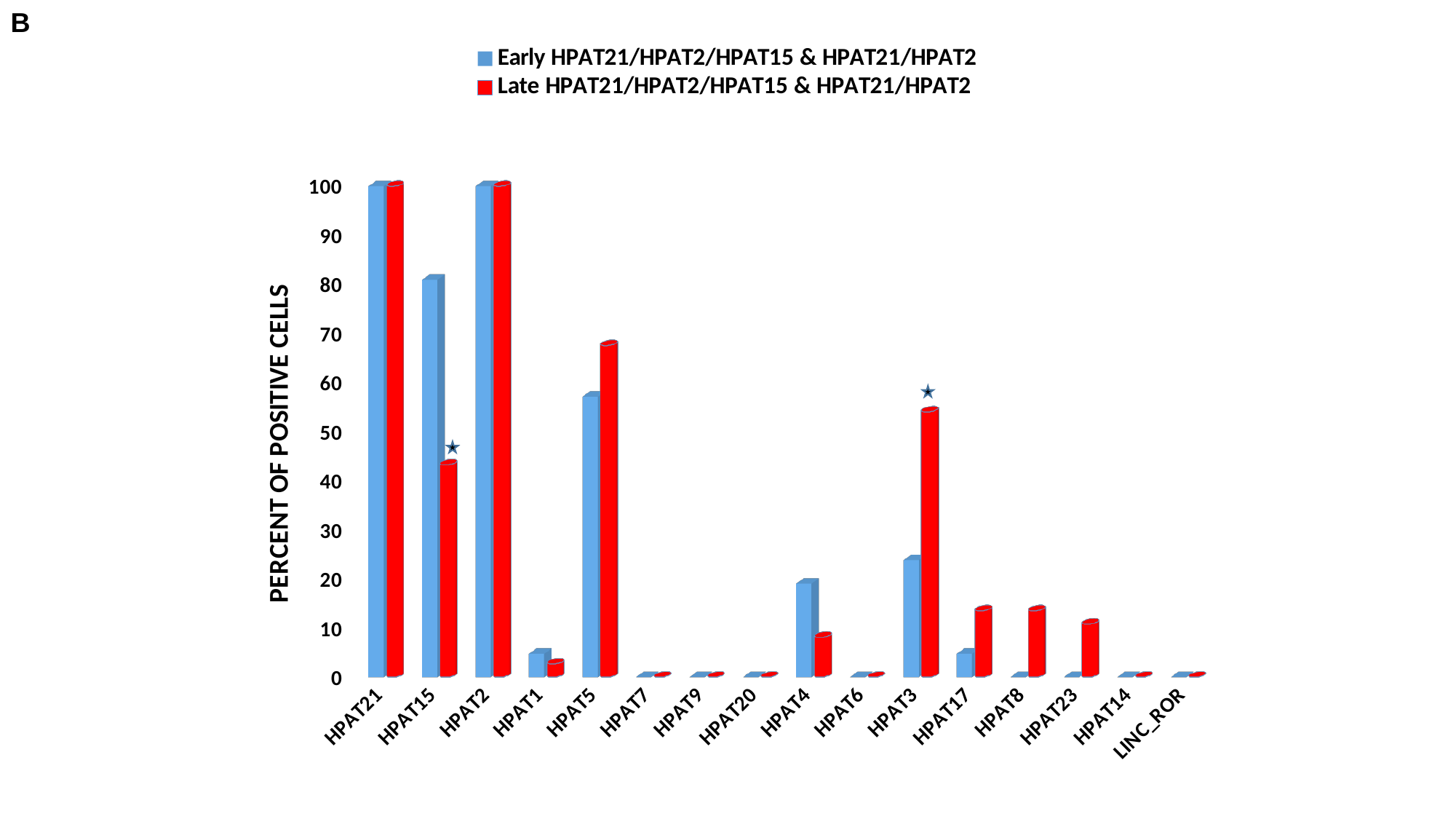

B
[unsupported chart]

## Slide 4
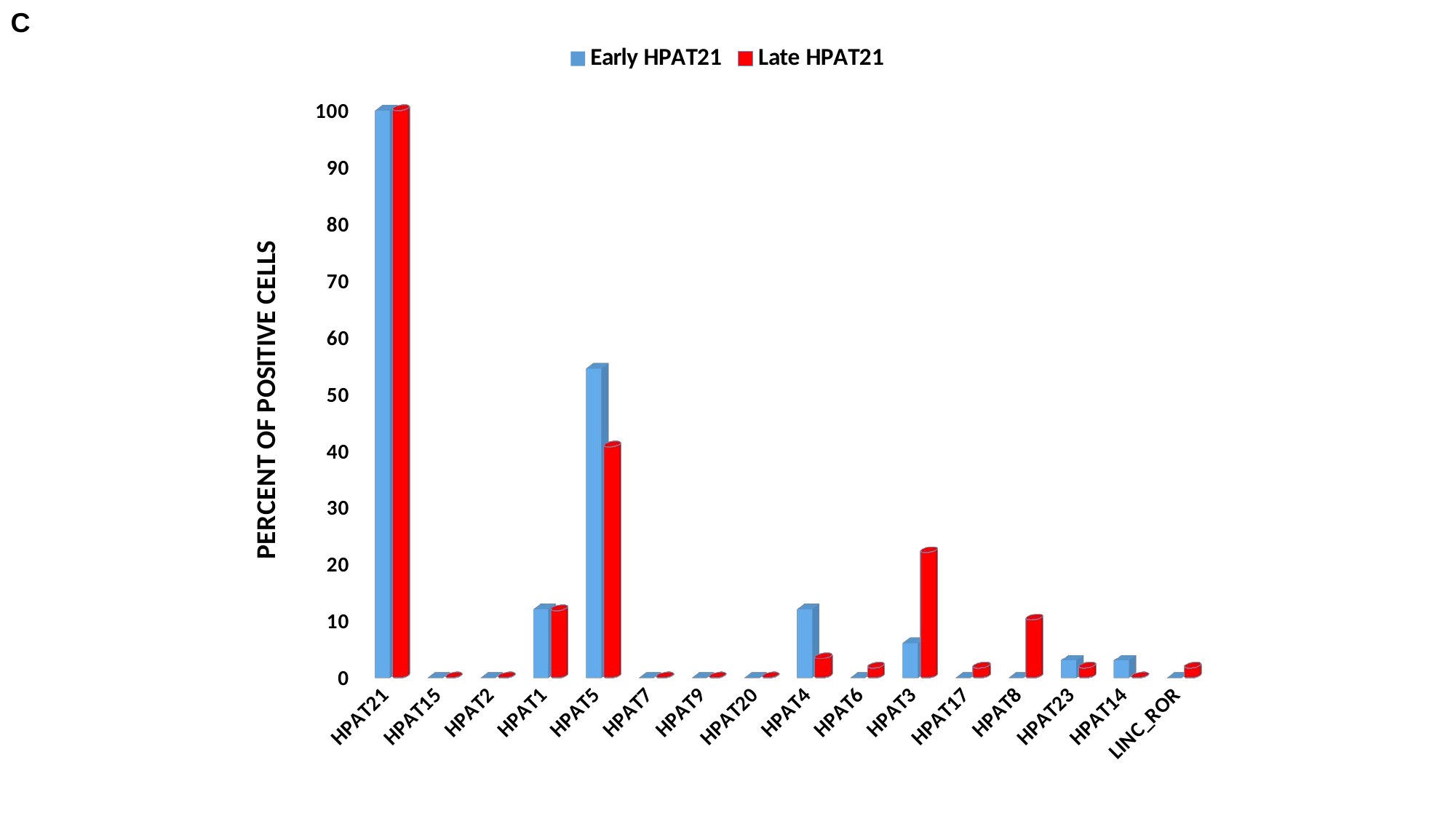

C
[unsupported chart]

## Slide 5
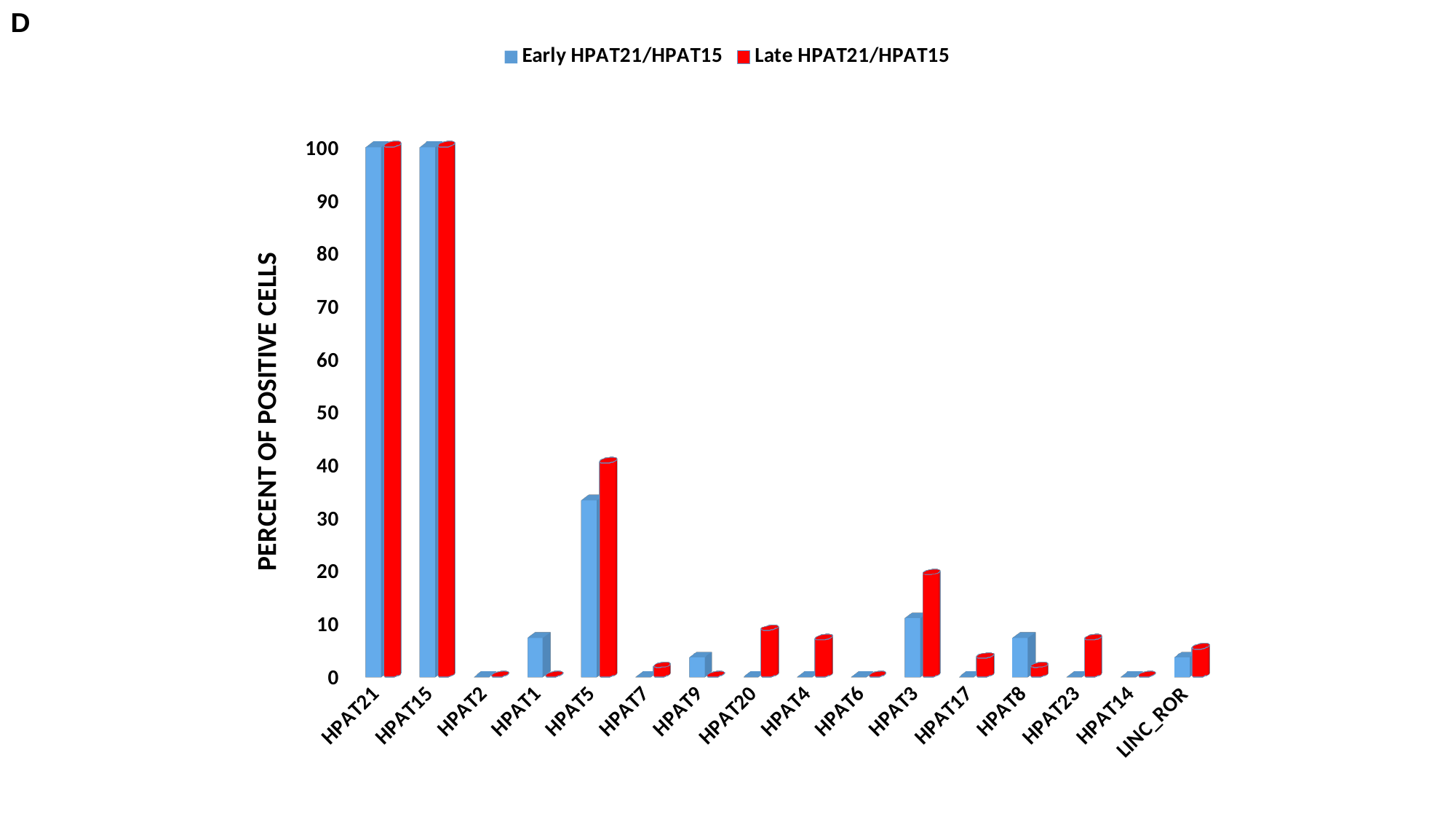

D
[unsupported chart]
