# Supplementary material for: Single cell expression analysis of primate-specific retroviruses-derived HPAT lincRNAs in viable human blastocysts identifies embryonic cells co-expressing genetic markers of multiple lineages
Source: Heliyon. 2018 Jun 28;4(6):e00667. doi: 10.1016/j.heliyon.2018.e00667 (PMC6039856; doi:10.1016/j.heliyon.2018.e00667)
Supplement: Supplemental Figure S2 [file mmc4.pptx]

## Slide 1
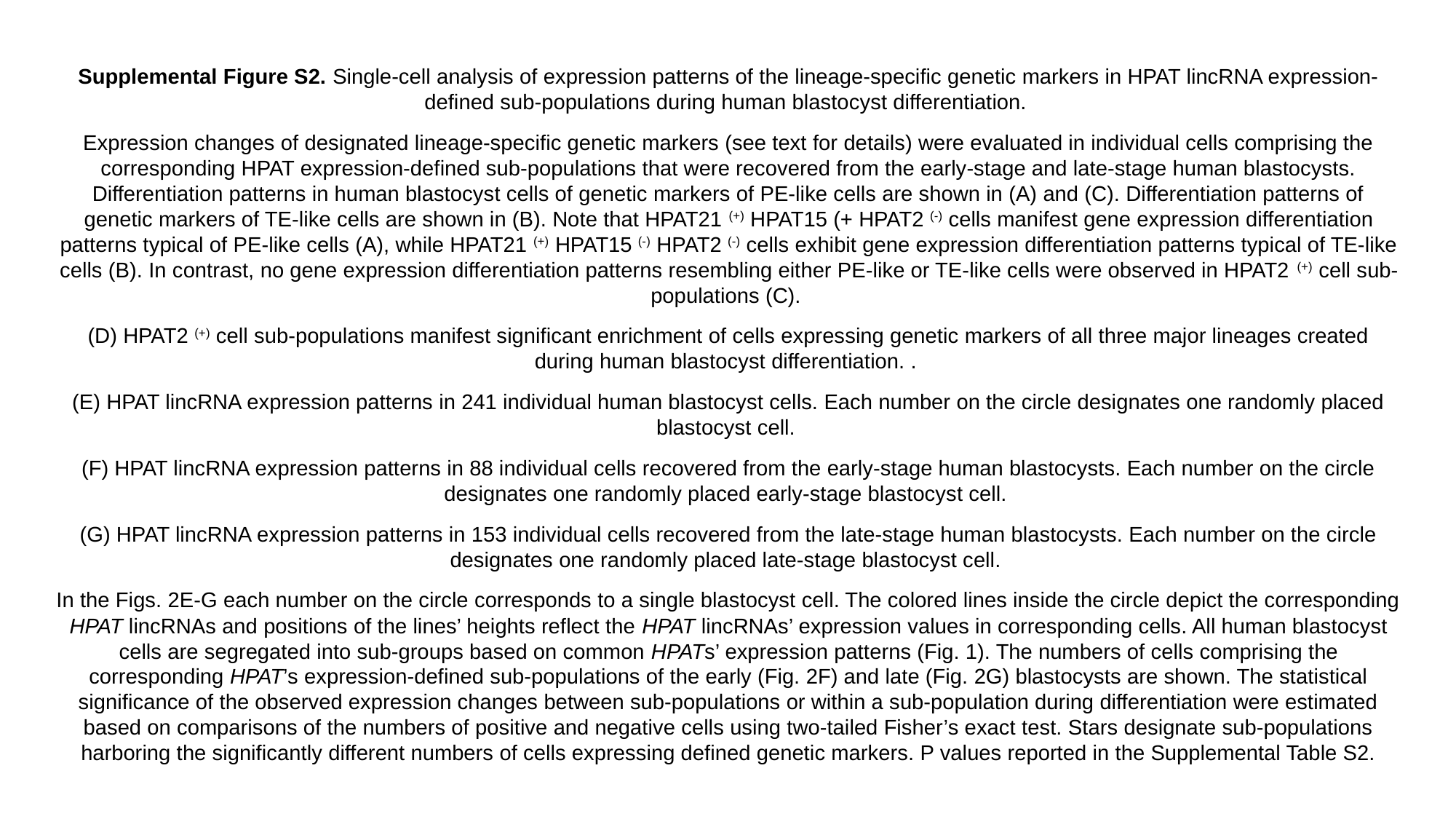

Supplemental Figure S2. Single-cell analysis of expression patterns of the lineage-specific genetic markers in HPAT lincRNA expression-defined sub-populations during human blastocyst differentiation.
Expression changes of designated lineage-specific genetic markers (see text for details) were evaluated in individual cells comprising the corresponding HPAT expression-defined sub-populations that were recovered from the early-stage and late-stage human blastocysts. Differentiation patterns in human blastocyst cells of genetic markers of PE-like cells are shown in (A) and (C). Differentiation patterns of genetic markers of TE-like cells are shown in (B). Note that HPAT21 (+) HPAT15 (+ HPAT2 (-) cells manifest gene expression differentiation patterns typical of PE-like cells (A), while HPAT21 (+) HPAT15 (-) HPAT2 (-) cells exhibit gene expression differentiation patterns typical of TE-like cells (B). In contrast, no gene expression differentiation patterns resembling either PE-like or TE-like cells were observed in HPAT2 (+) cell sub-populations (C).
(D) HPAT2 (+) cell sub-populations manifest significant enrichment of cells expressing genetic markers of all three major lineages created during human blastocyst differentiation. .
(E) HPAT lincRNA expression patterns in 241 individual human blastocyst cells. Each number on the circle designates one randomly placed blastocyst cell.
(F) HPAT lincRNA expression patterns in 88 individual cells recovered from the early-stage human blastocysts. Each number on the circle designates one randomly placed early-stage blastocyst cell.
(G) HPAT lincRNA expression patterns in 153 individual cells recovered from the late-stage human blastocysts. Each number on the circle designates one randomly placed late-stage blastocyst cell.
In the Figs. 2E-G each number on the circle corresponds to a single blastocyst cell. The colored lines inside the circle depict the corresponding HPAT lincRNAs and positions of the lines’ heights reflect the HPAT lincRNAs’ expression values in corresponding cells. All human blastocyst cells are segregated into sub-groups based on common HPATs’ expression patterns (Fig. 1). The numbers of cells comprising the corresponding HPAT’s expression-defined sub-populations of the early (Fig. 2F) and late (Fig. 2G) blastocysts are shown. The statistical significance of the observed expression changes between sub-populations or within a sub-population during differentiation were estimated based on comparisons of the numbers of positive and negative cells using two-tailed Fisher’s exact test. Stars designate sub-populations harboring the significantly different numbers of cells expressing defined genetic markers. P values reported in the Supplemental Table S2.

## Slide 2
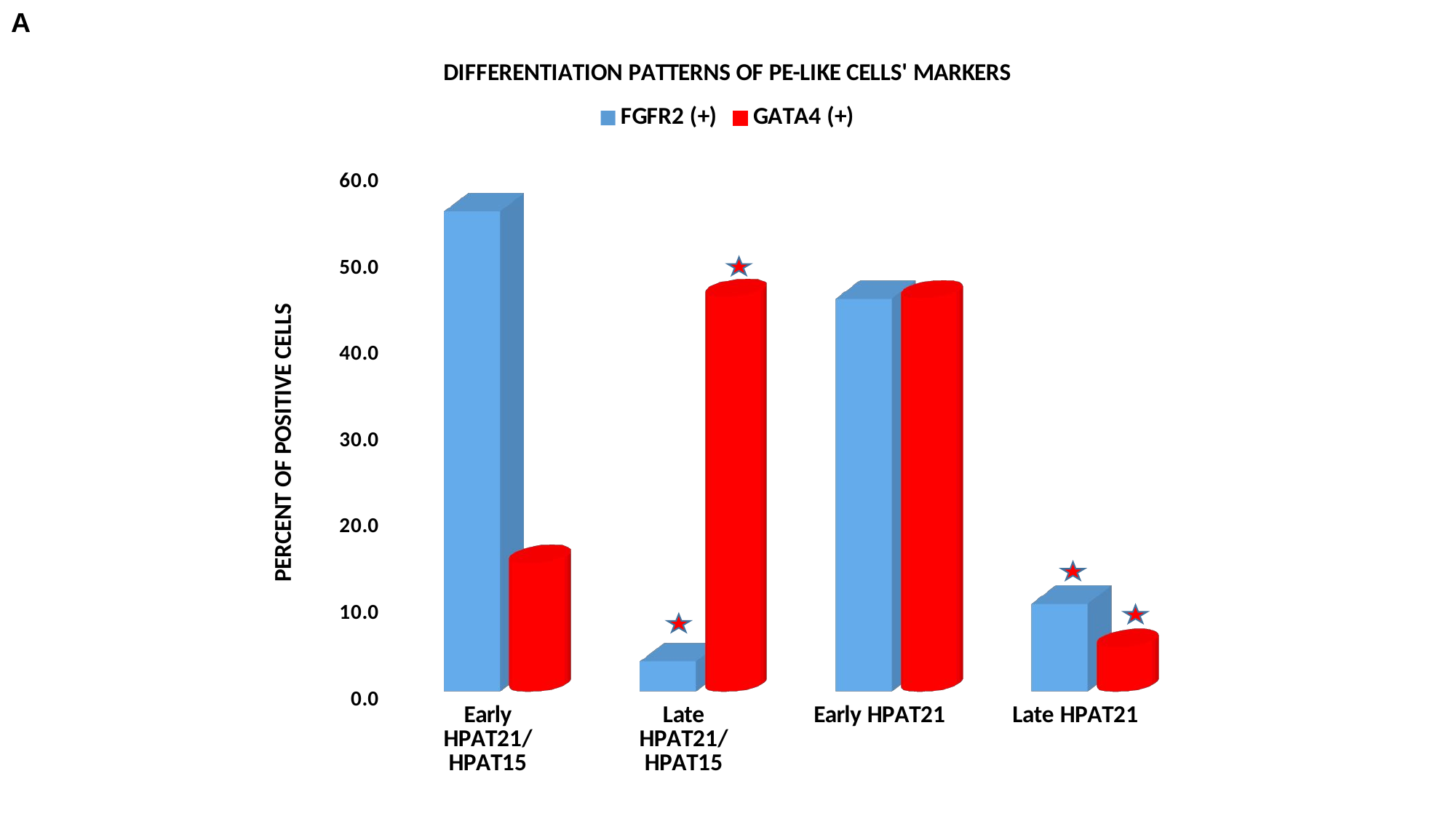

A
[unsupported chart]

## Slide 3
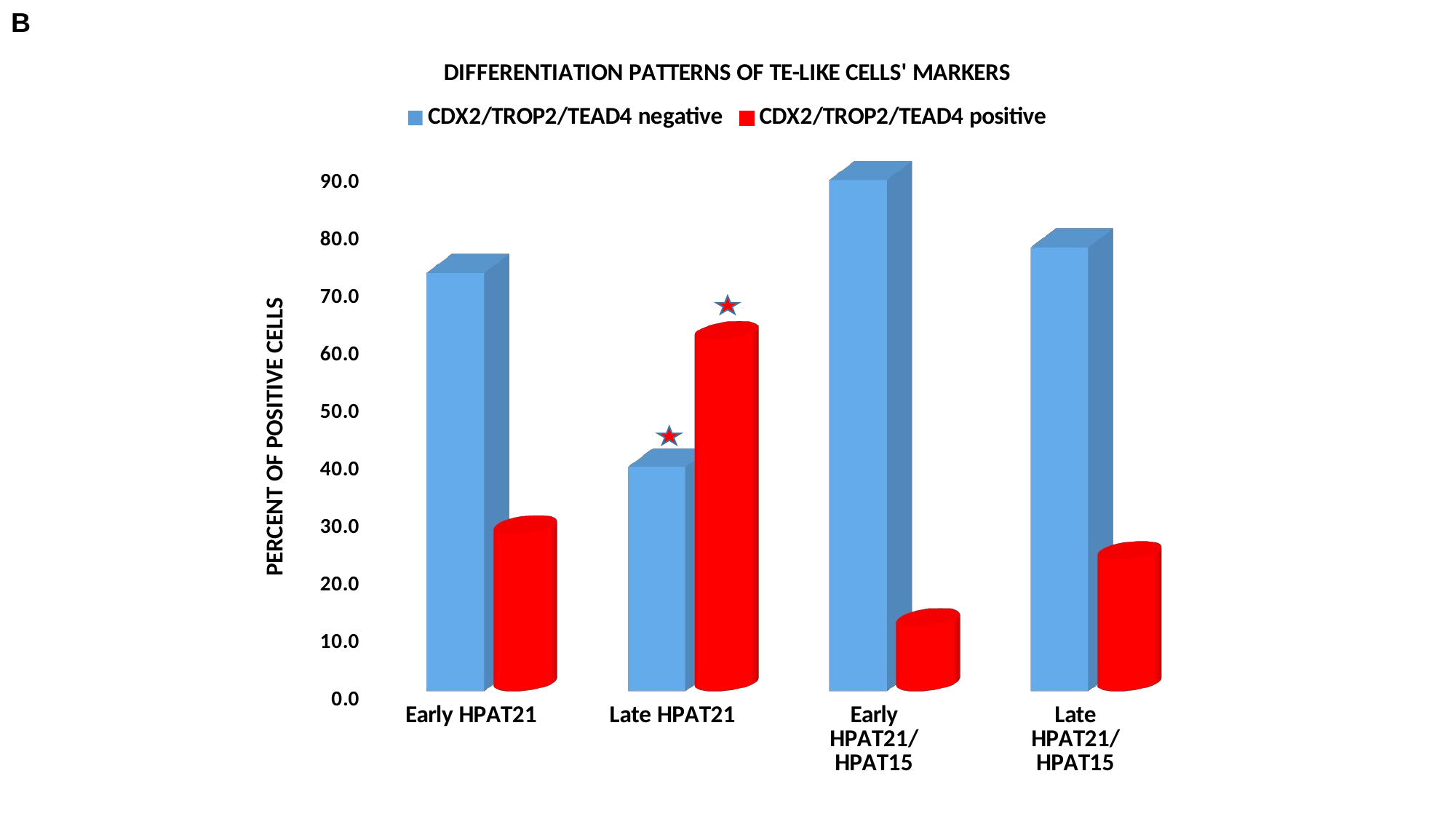

B
[unsupported chart]

## Slide 4
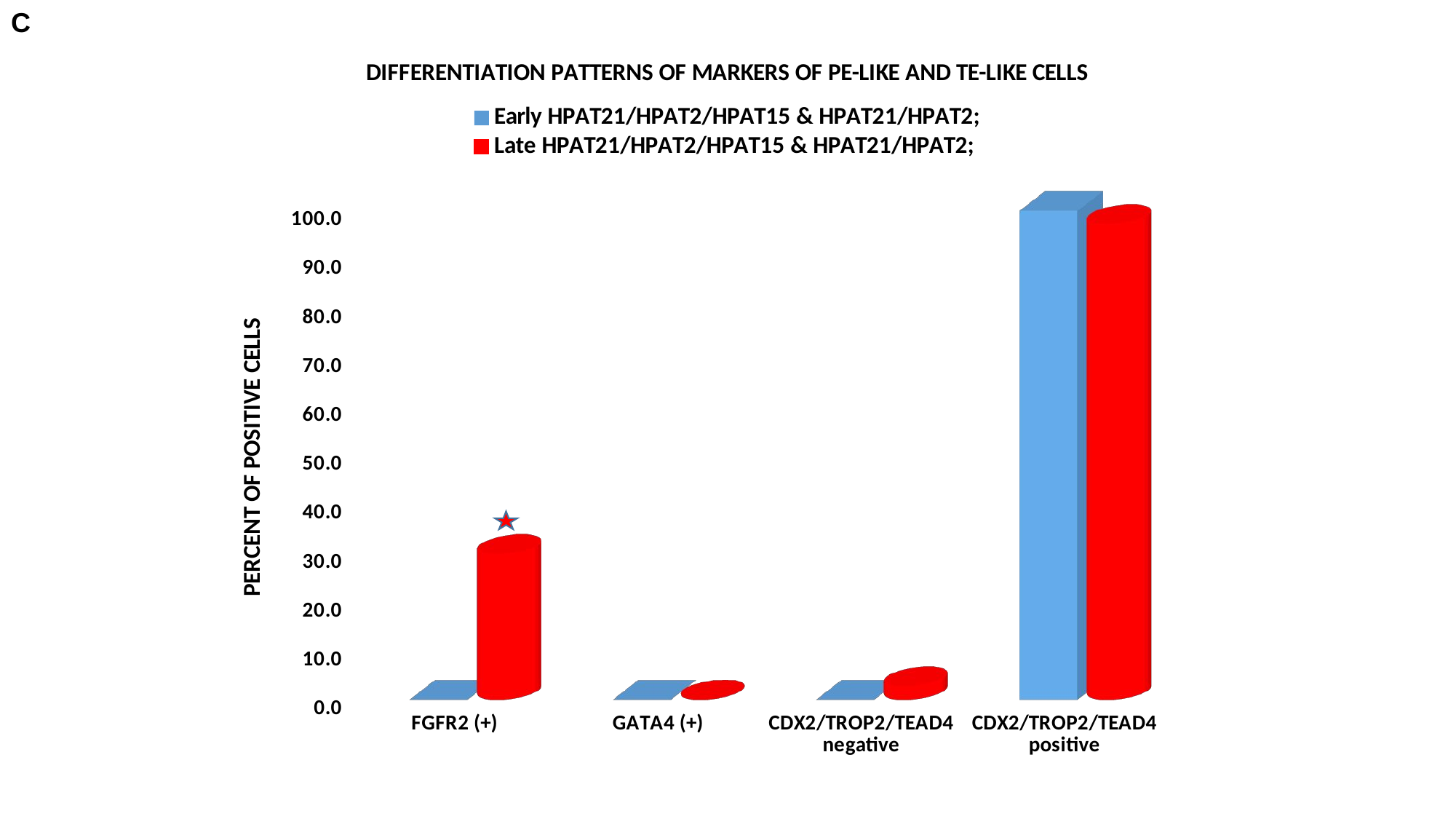

C
[unsupported chart]

## Slide 5
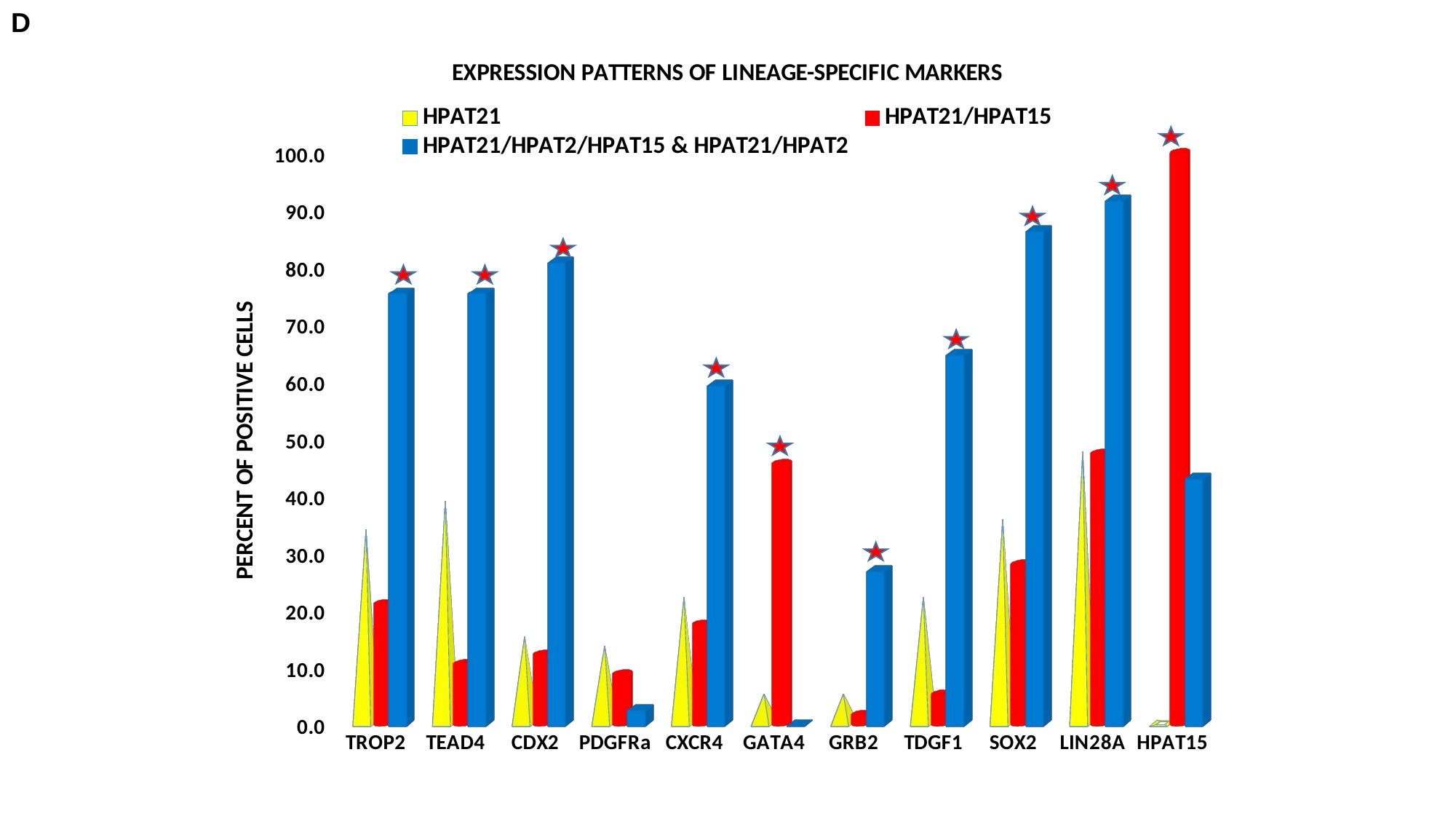

D
[unsupported chart]

## Slide 6
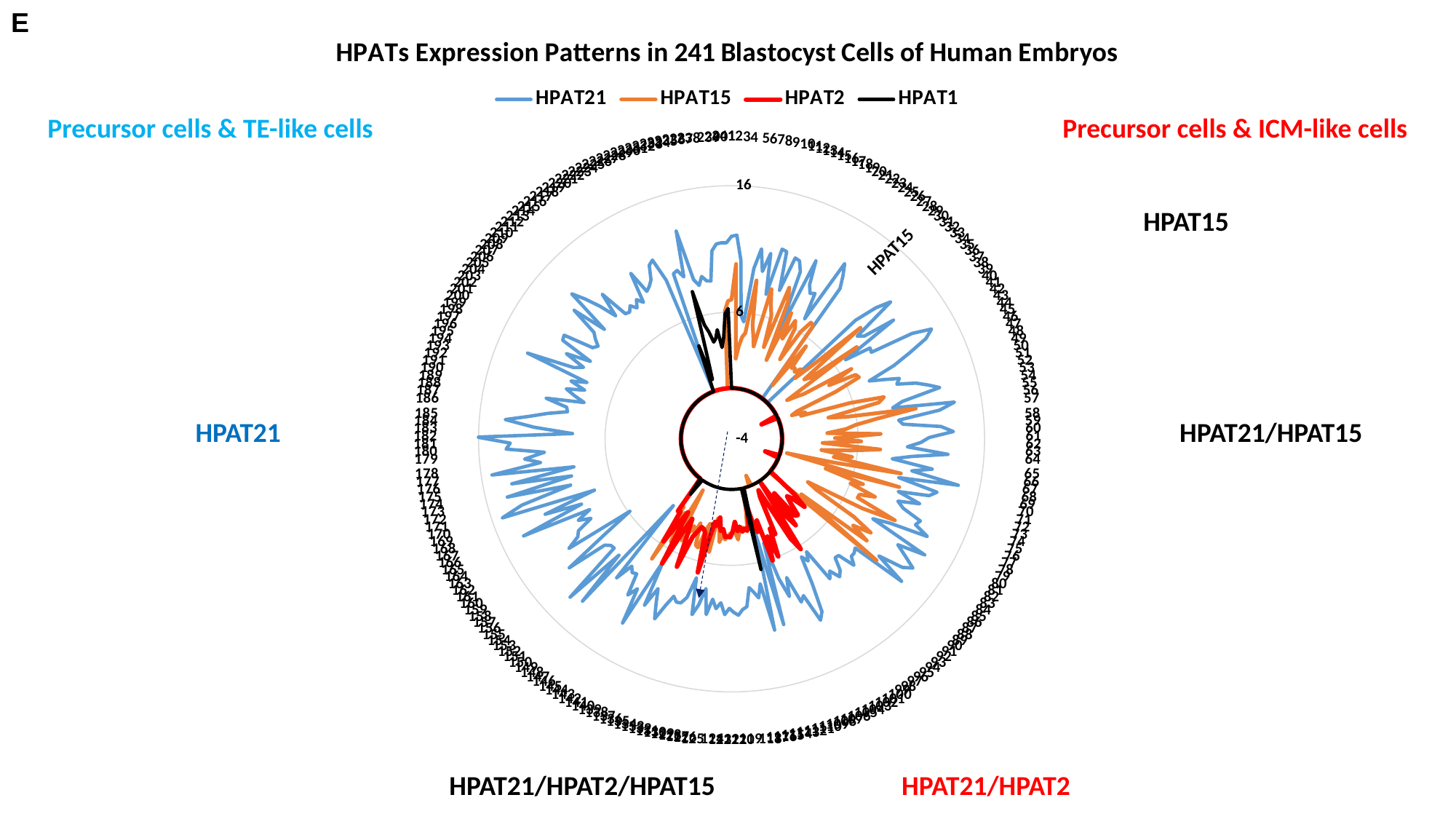

E
### Chart: HPATs Expression Patterns in 241 Blastocyst Cells of Human Embryos
| Category | HPAT21 | HPAT15 | HPAT2 | HPAT1 |
|---|---|---|---|---|Precursor cells & TE-like cells
Precursor cells & ICM-like cells
HPAT15
HPAT15
HPAT21
HPAT21/HPAT15
HPAT21/HPAT2/HPAT15
HPAT21/HPAT2

## Slide 7
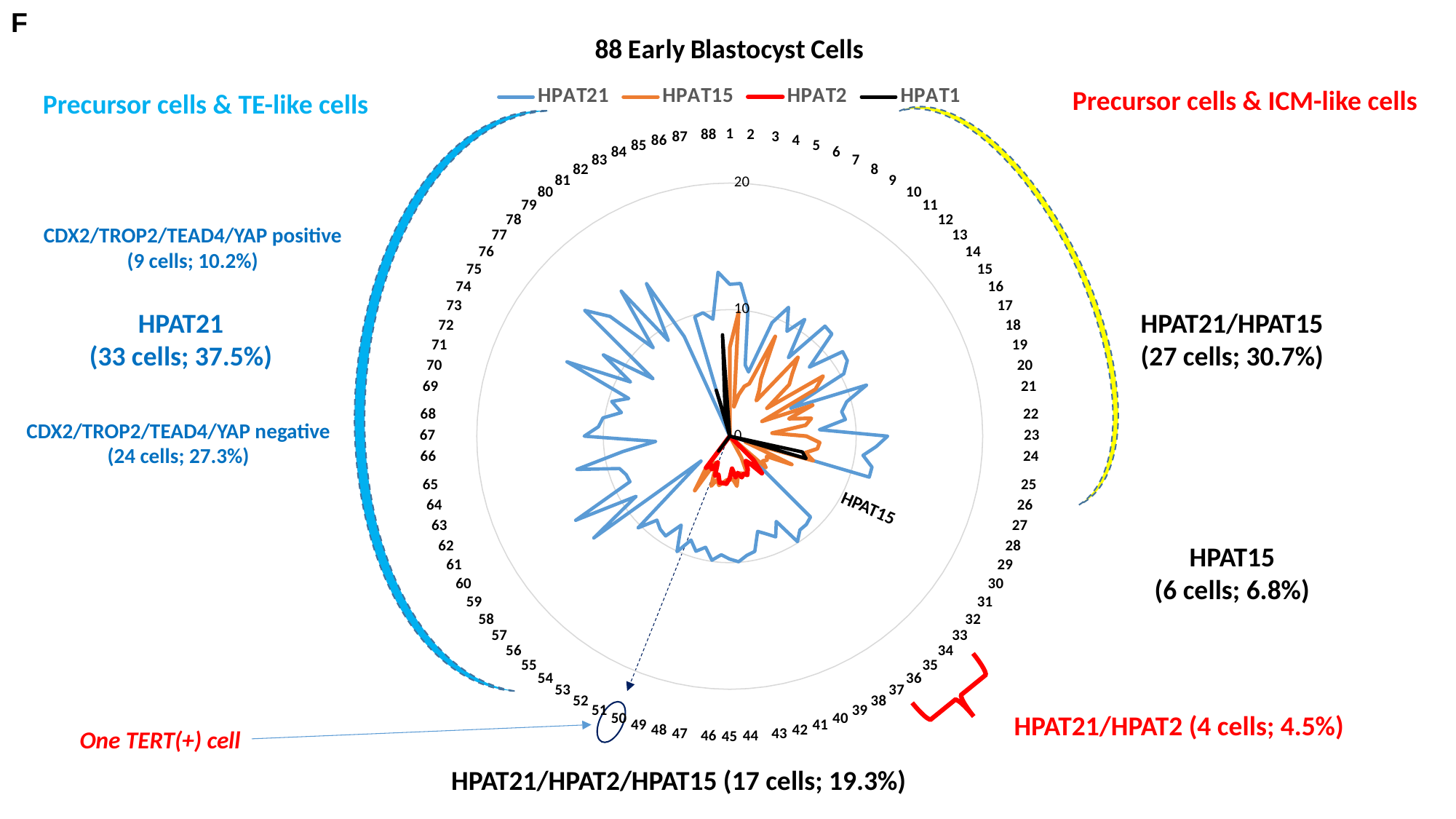

F
### Chart: 88 Early Blastocyst Cells
| Category | HPAT21 | HPAT15 | HPAT2 | HPAT1 |
|---|---|---|---|---|
Precursor cells & ICM-like cells
Precursor cells & TE-like cells
CDX2/TROP2/TEAD4/YAP positive
(9 cells; 10.2%)
HPAT21
(33 cells; 37.5%)
HPAT21/HPAT15
(27 cells; 30.7%)
CDX2/TROP2/TEAD4/YAP negative
(24 cells; 27.3%)
HPAT15
HPAT15
(6 cells; 6.8%)
HPAT21/HPAT2 (4 cells; 4.5%)
One TERT(+) cell
HPAT21/HPAT2/HPAT15 (17 cells; 19.3%)

## Slide 8
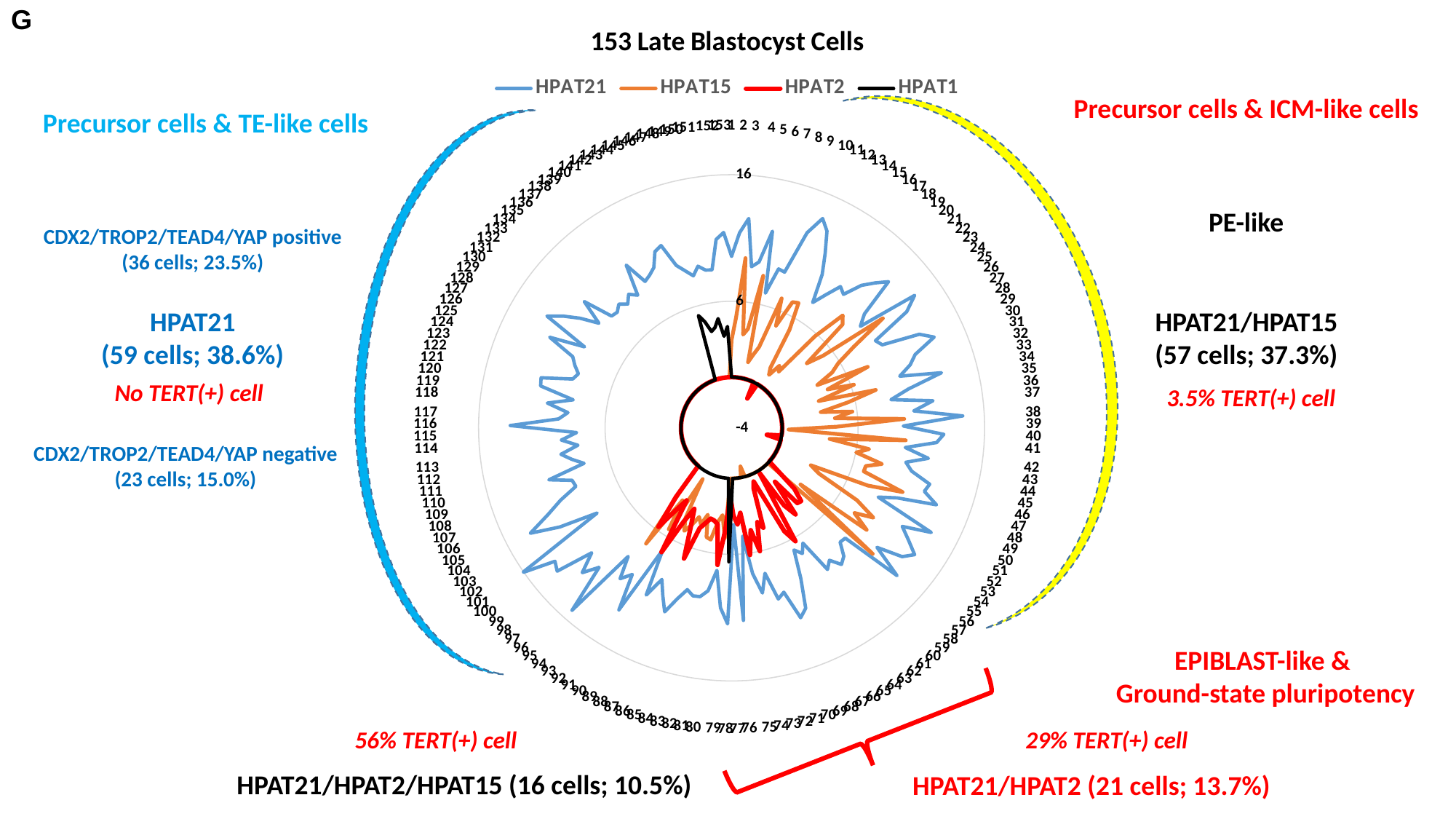

G
### Chart: 153 Late Blastocyst Cells
| Category | HPAT21 | HPAT15 | HPAT2 | HPAT1 |
|---|---|---|---|---|
Precursor cells & ICM-like cells
Precursor cells & TE-like cells
PE-like
CDX2/TROP2/TEAD4/YAP positive
(36 cells; 23.5%)
HPAT21/HPAT15
(57 cells; 37.3%)
HPAT21
(59 cells; 38.6%)
No TERT(+) cell
3.5% TERT(+) cell
CDX2/TROP2/TEAD4/YAP negative
(23 cells; 15.0%)
EPIBLAST-like &
Ground-state pluripotency
56% TERT(+) cell
29% TERT(+) cell
HPAT21/HPAT2/HPAT15 (16 cells; 10.5%)
HPAT21/HPAT2 (21 cells; 13.7%)
