# Supplementary material for: Single cell expression analysis of primate-specific retroviruses-derived HPAT lincRNAs in viable human blastocysts identifies embryonic cells co-expressing genetic markers of multiple lineages
Source: Heliyon. 2018 Jun 28;4(6):e00667. doi: 10.1016/j.heliyon.2018.e00667 (PMC6039856; doi:10.1016/j.heliyon.2018.e00667)
Supplement: Supplemental Figure S2.2 [file mmc5.pptx]

## Slide 1
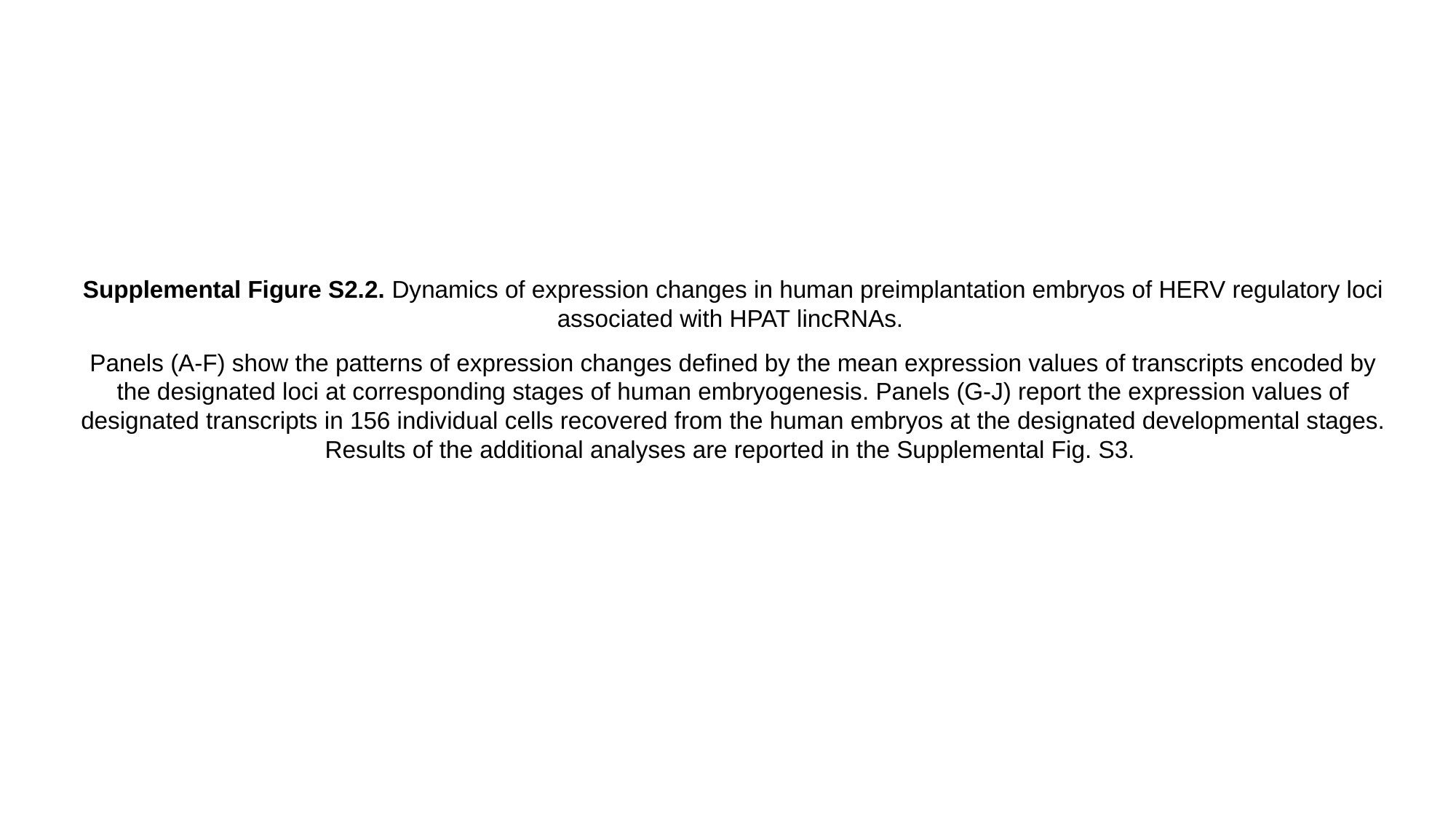

Supplemental Figure S2.2. Dynamics of expression changes in human preimplantation embryos of HERV regulatory loci associated with HPAT lincRNAs.
Panels (A-F) show the patterns of expression changes defined by the mean expression values of transcripts encoded by the designated loci at corresponding stages of human embryogenesis. Panels (G-J) report the expression values of designated transcripts in 156 individual cells recovered from the human embryos at the designated developmental stages. Results of the additional analyses are reported in the Supplemental Fig. S3.

## Slide 2
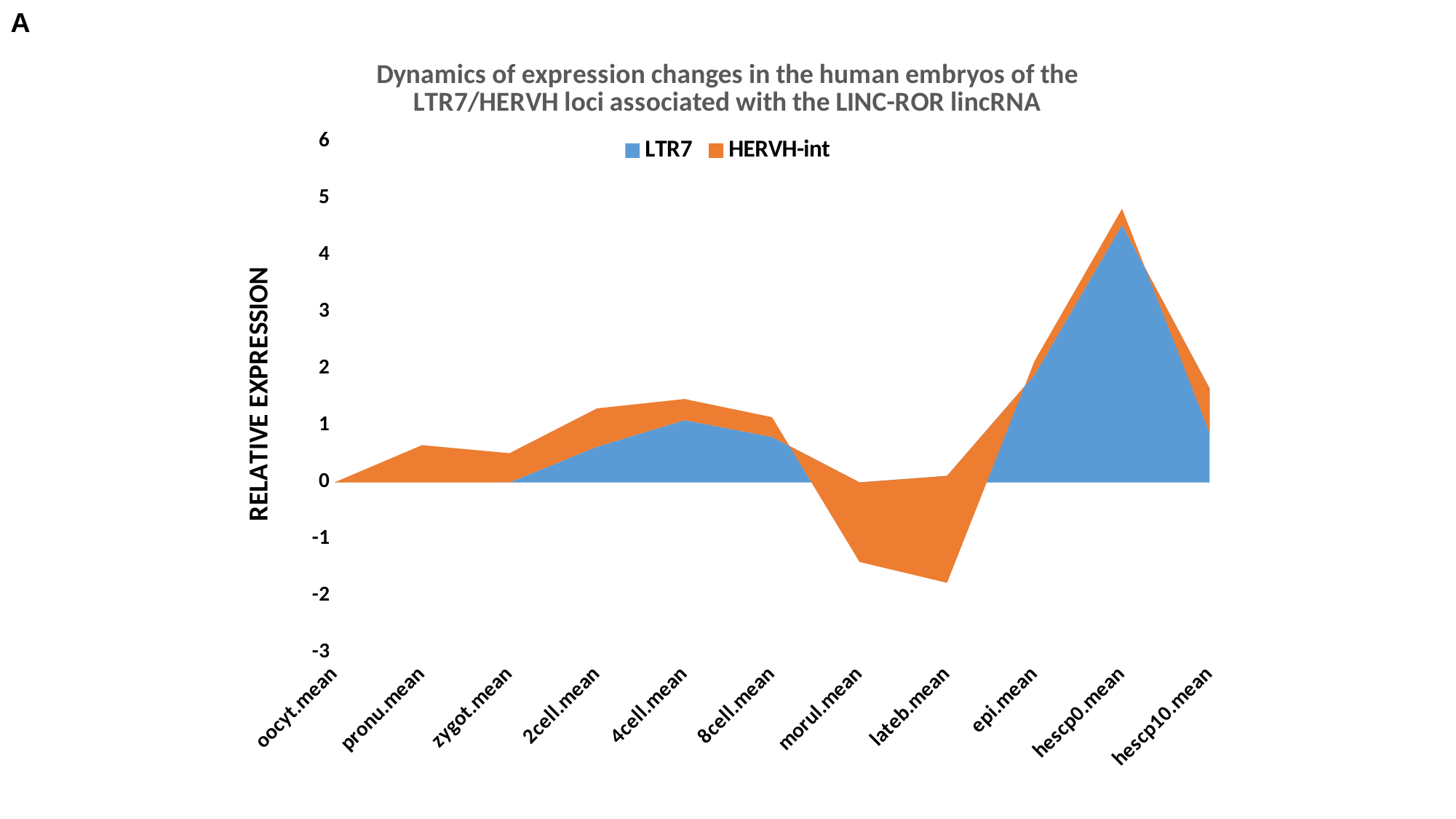

A
### Chart: Dynamics of expression changes in the human embryos of the LTR7/HERVH loci associated with the LINC-ROR lincRNA
| Category | LTR7 | HERVH-int |
|---|---|---|
| oocyt.mean | 0.0 | 0.0 |
| pronu.mean | 0.0 | 0.6557102962523 |
| zygot.mean | 0.0 | 0.51358644483393 |
| 2cell.mean | 0.6285179235327201 | 0.67489887333509 |
| 4cell.mean | 1.0949624925445902 | 0.3731479767146799 |
| 8cell.mean | 0.80106955734266 | 0.34638123337446003 |
| morul.mean | 0.0 | -1.4002646134703398 |
| lateb.mean | 0.11438015164681037 | -1.8825741672689 |
| epi.mean | 1.8940951961134602 | 0.24353901782951004 |
| hescp0.mean | 4.53326676322428 | 0.2835689938961701 |
| hescp10.mean | 1.6553053710758099 | -0.7800301099268898 |

## Slide 3
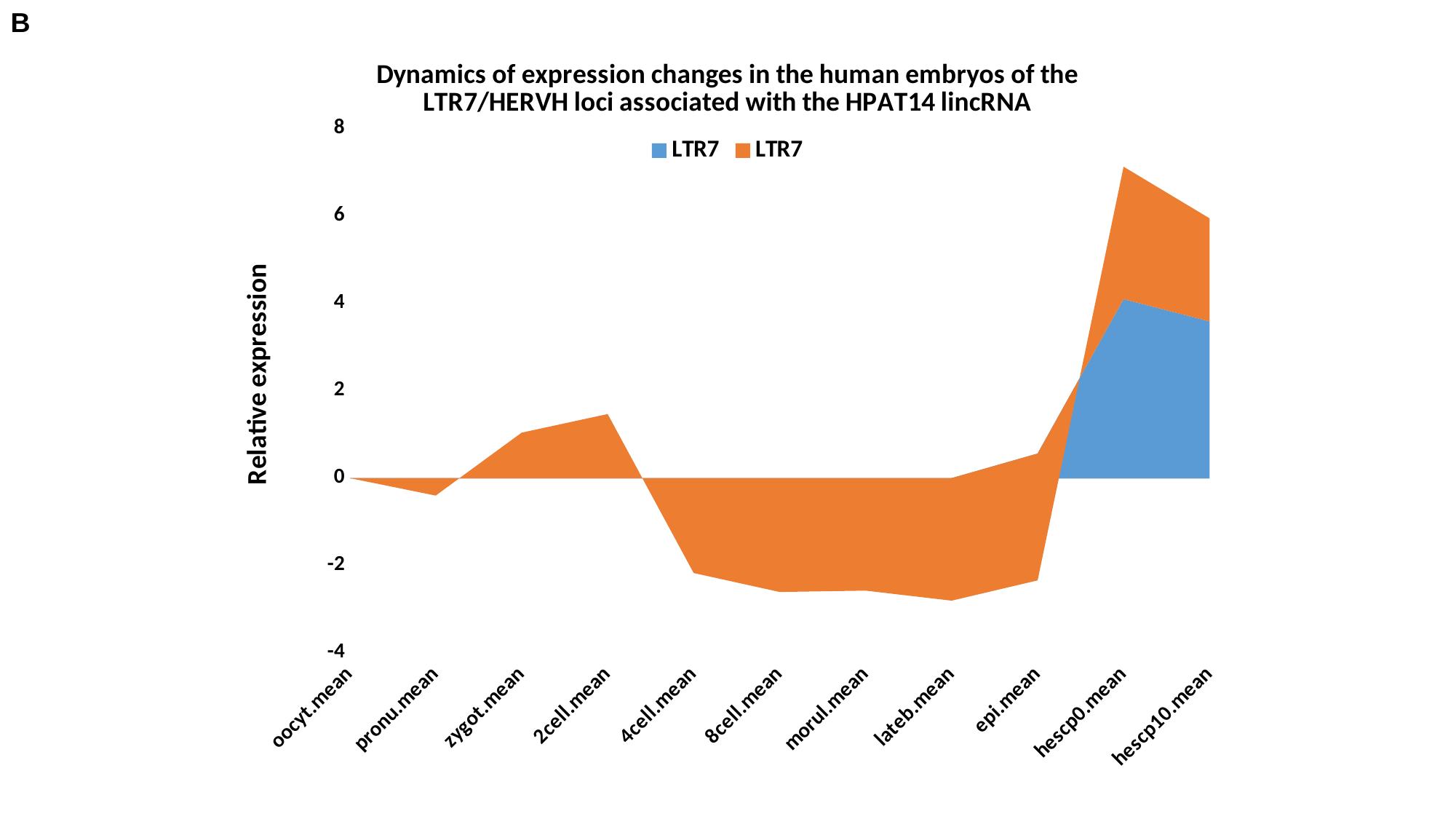

B
### Chart: Dynamics of expression changes in the human embryos of the LTR7/HERVH loci associated with the HPAT14 lincRNA
| Category | LTR7 | LTR7 |
|---|---|---|
| oocyt.mean | 0.0 | 0.0 |
| pronu.mean | 0.0 | -0.4002615400693301 |
| zygot.mean | 0.0 | 1.0399725430391777 |
| 2cell.mean | 0.0 | 1.466289862000549 |
| 4cell.mean | 0.0 | -2.17262014512914 |
| 8cell.mean | 0.0 | -2.6071498759261797 |
| morul.mean | 0.0 | -2.57631717062189 |
| lateb.mean | 0.0 | -2.8058866578133004 |
| epi.mean | 0.5629222421662599 | -2.90470397334481 |
| hescp0.mean | 4.098295640369977 | 3.03140619398007 |
| hescp10.mean | 3.58846489115345 | 2.3591686820455697 |

## Slide 4
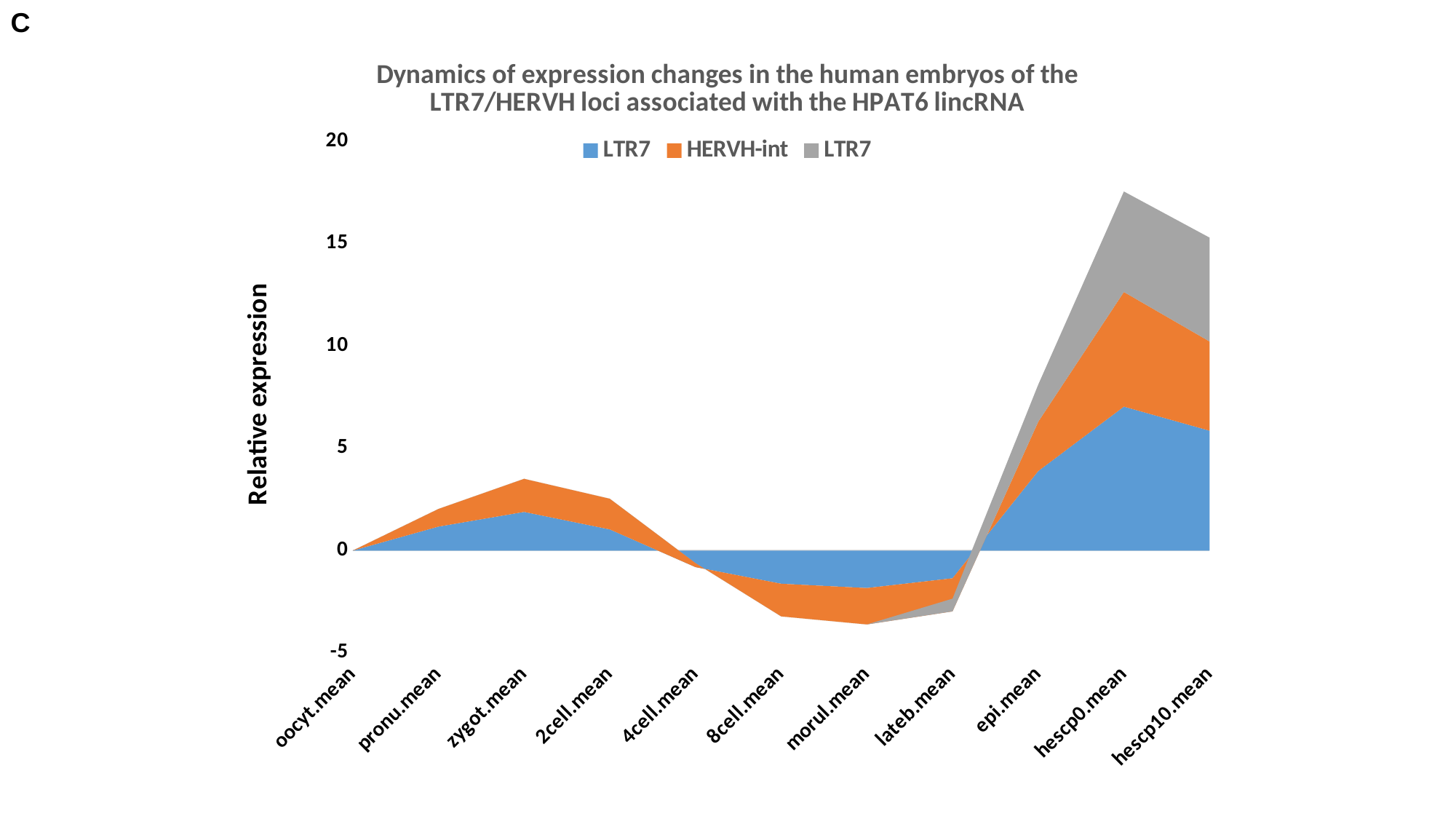

C
### Chart: Dynamics of expression changes in the human embryos of the LTR7/HERVH loci associated with the HPAT6 lincRNA
| Category | LTR7 | HERVH-int | LTR7 |
|---|---|---|---|
| oocyt.mean | 0.0 | 0.0 | 0.0 |
| pronu.mean | 1.1714776529550202 | 0.86414645036259 | 0.0 |
| zygot.mean | 1.8898259165293192 | 1.618997317382569 | 0.0 |
| 2cell.mean | 1.03352631822949 | 1.500723674184424 | 0.0 |
| 4cell.mean | -0.8100771064480798 | 0.19809275077799993 | 0.0 |
| 8cell.mean | -1.62338086200828 | -1.5974201745658698 | 0.0 |
| morul.mean | -1.8338239874187696 | -1.7796271201841498 | 0.0 |
| lateb.mean | -1.3563344277447098 | -1.6178998074158302 | 0.6224959705428601 |
| epi.mean | 3.8867775345674804 | 2.411101937447671 | 1.8140040055661601 |
| hescp0.mean | 7.04017664831321 | 5.60631035732946 | 4.912453668789497 |
| hescp10.mean | 5.85999377131928 | 4.36523012360891 | 5.076171091274579 |

## Slide 5
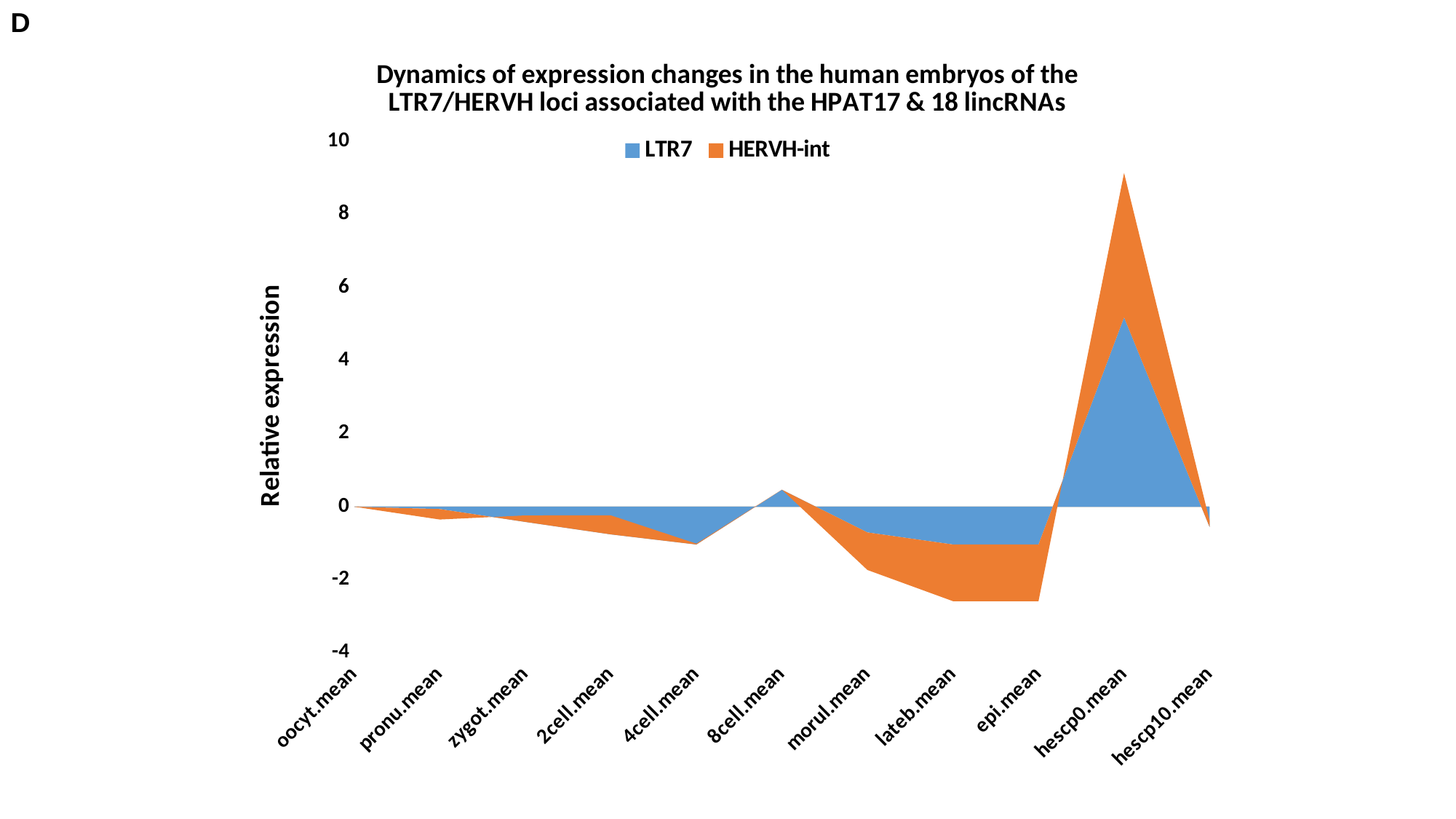

D
### Chart: Dynamics of expression changes in the human embryos of the LTR7/HERVH loci associated with the HPAT17 & 18 lincRNAs
| Category | LTR7 | HERVH-int |
|---|---|---|
| oocyt.mean | 0.0 | 0.0 |
| pronu.mean | -0.06445012385061011 | -0.28581117520279964 |
| zygot.mean | -0.41648147478311026 | 0.1784723304233502 |
| 2cell.mean | -0.7578647570624404 | 0.5203258669878501 |
| 4cell.mean | -1.0344476193984202 | 0.02245548467646019 |
| 8cell.mean | 0.4640737429339796 | 0.0029307019739501783 |
| morul.mean | -0.7032484592753305 | -1.0291287397914002 |
| lateb.mean | -1.0344476193984202 | -1.5513440289185598 |
| epi.mean | -1.0344476193984202 | -1.5513440289185598 |
| hescp0.mean | 5.18600905339905 | 3.955630086779477 |
| hescp10.mean | -0.55213440908117 | 0.17403552397654032 |

## Slide 6
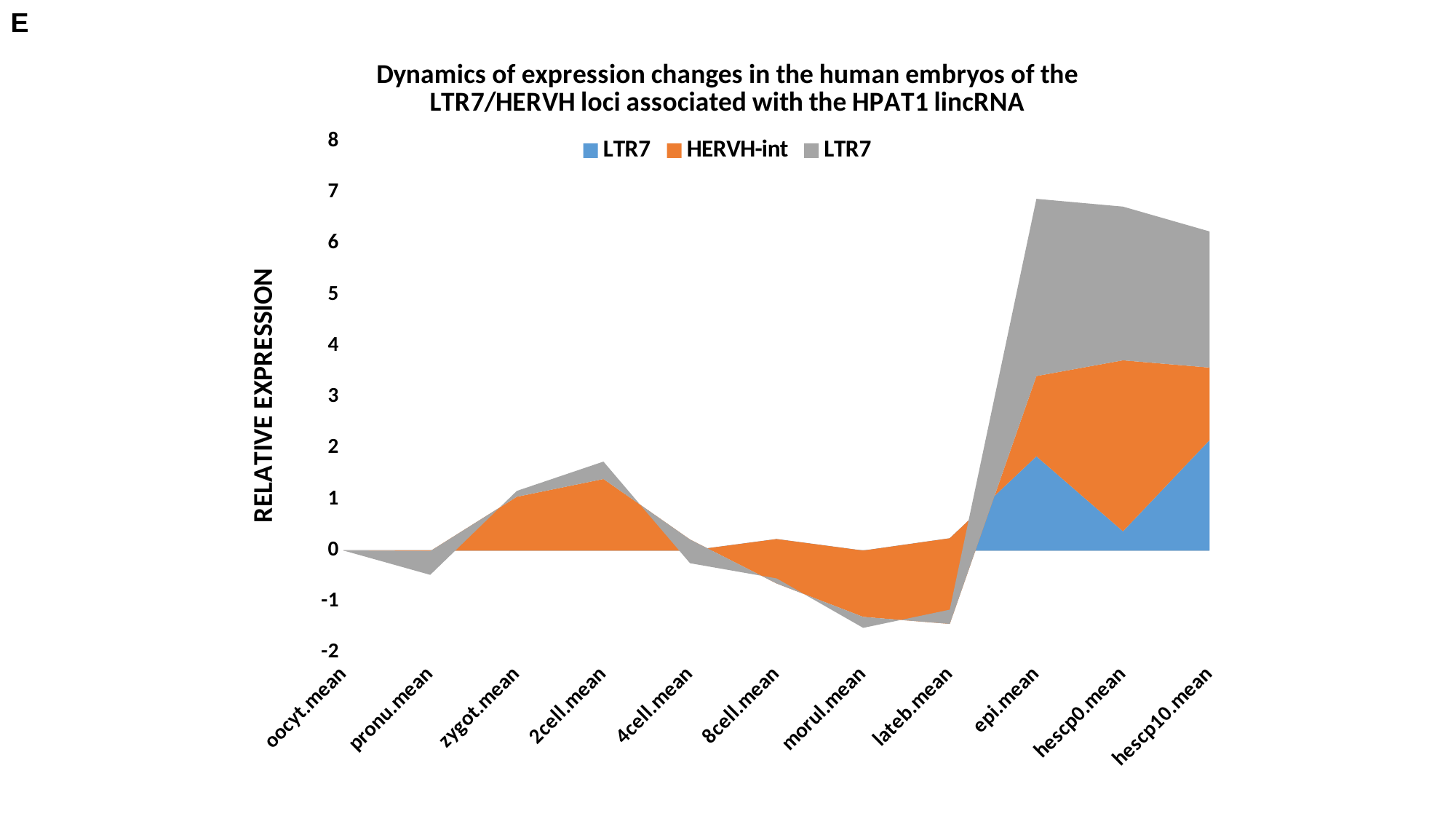

E
### Chart: Dynamics of expression changes in the human embryos of the LTR7/HERVH loci associated with the HPAT1 lincRNA
| Category | LTR7 | HERVH-int | LTR7 |
|---|---|---|---|
| oocyt.mean | 0.0 | 0.0 | 0.0 |
| pronu.mean | 0.0 | -0.012141352241290093 | -0.46292419843665034 |
| zygot.mean | 0.0 | 1.0513826761921399 | 0.11457306643635956 |
| 2cell.mean | 0.0 | 1.3989807304934498 | 0.3409057717436599 |
| 4cell.mean | 0.0 | 0.2159249216606498 | -0.46292419843665034 |
| 8cell.mean | 0.2249661573396704 | -0.8692378112702204 | 0.09913924546606001 |
| morul.mean | 0.0 | -1.2970756602919304 | -0.21493843406644064 |
| lateb.mean | 0.24026212540166014 | -1.67481942870675 | 0.2784423568081795 |
| epi.mean | 1.84421239492909 | 1.5695001010250897 | 3.465704498201936 |
| hescp0.mean | 0.3763352979640304 | 3.345400347344879 | 3.0060646081999494 |
| hescp10.mean | 2.16143415080018 | 1.4182331917924598 | 2.66142635256991 |

## Slide 7
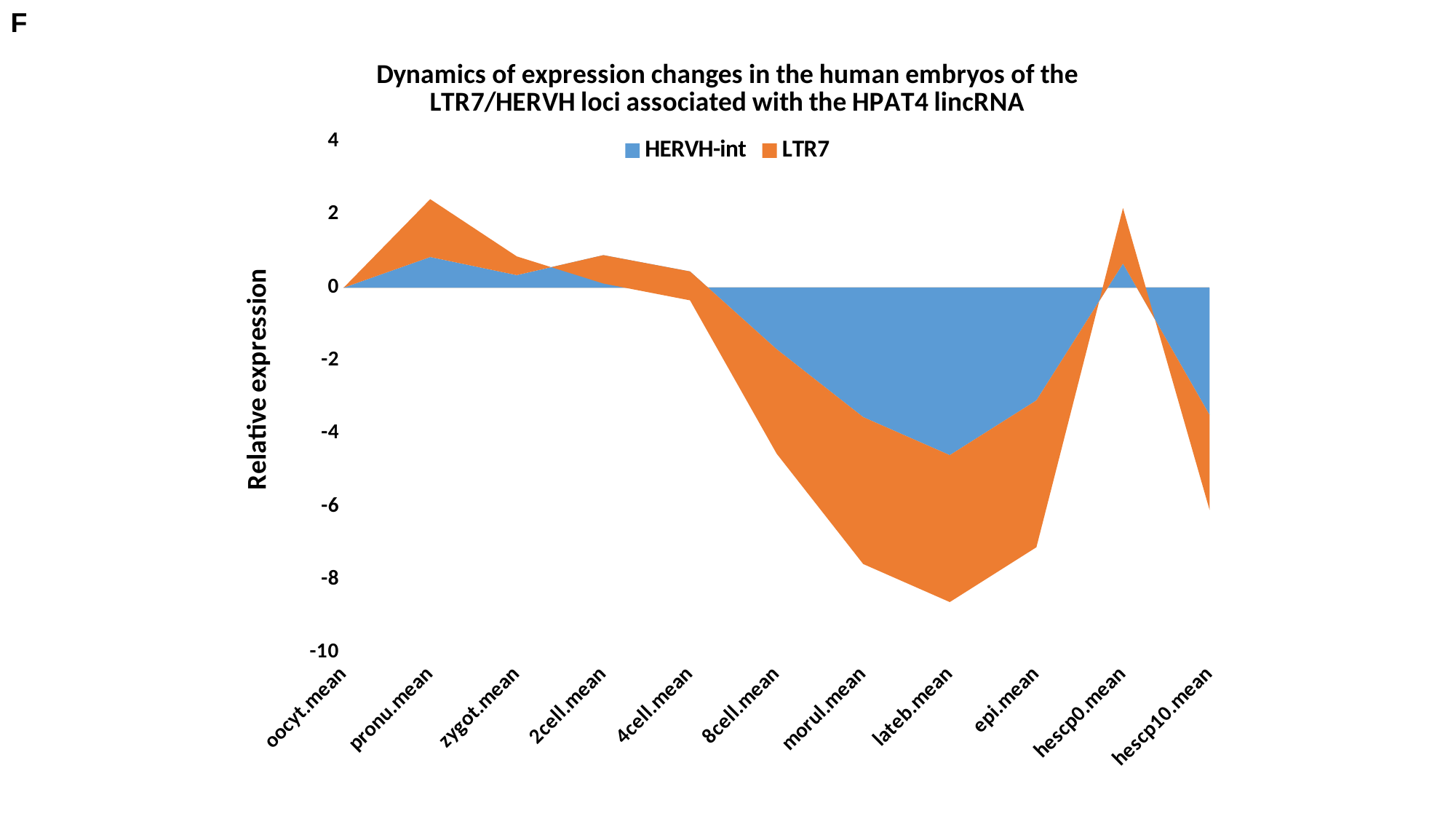

F
### Chart: Dynamics of expression changes in the human embryos of the LTR7/HERVH loci associated with the HPAT4 lincRNA
| Category | HERVH-int | LTR7 |
|---|---|---|
| oocyt.mean | 0.0 | 0.0 |
| pronu.mean | 0.8386506673215641 | 1.583154481076979 |
| zygot.mean | 0.343030480522122 | 0.5121828875587819 |
| 2cell.mean | 0.889480827298694 | -0.7766880734076909 |
| 4cell.mean | 0.443647362475936 | -0.791684486938611 |
| 8cell.mean | -1.6856897713994958 | -2.859594046809771 |
| morul.mean | -3.545947524977536 | -4.022983214749301 |
| lateb.mean | -4.587762310917056 | -4.022983214749301 |
| epi.mean | -3.0866821211003463 | -4.022983214749301 |
| hescp0.mean | 0.660101312091314 | 1.518232428977508 |
| hescp10.mean | -3.490362794888426 | -2.603193424917171 |

## Slide 8
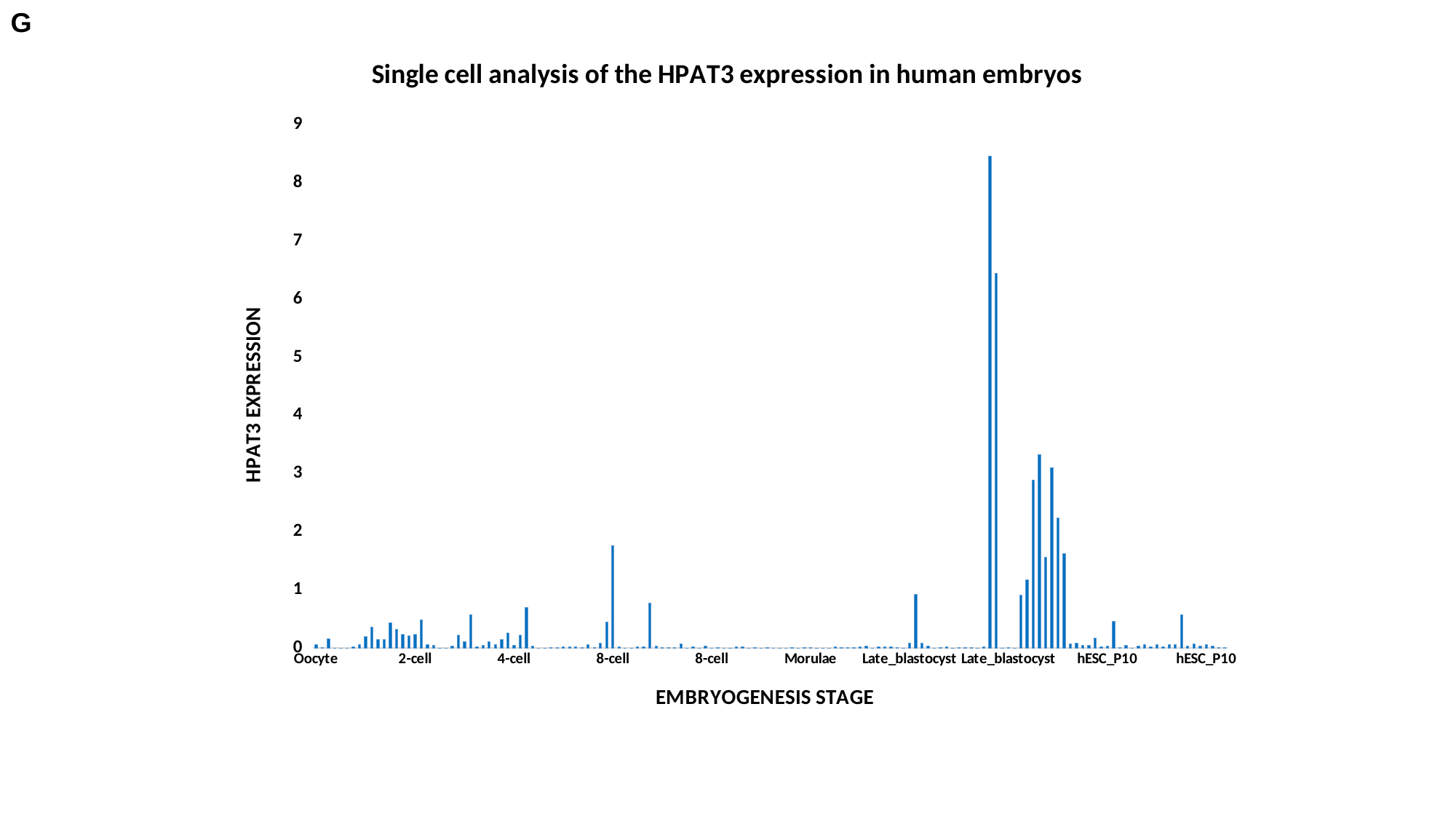

G
### Chart: Single cell analysis of the HPAT3 expression in human embryos
| Category | HPAT3 |
|---|---|
| Oocyte | 0.0541 |
| Oocyte | 0.0116 |
| Oocyte | 0.1603 |
| Oocyte | 0.0 |
| Oocyte | 0.0 |
| Oocyte | 0.0 |
| Pronuclei | 0.014 |
| Pronuclei | 0.0535 |
| Pronuclei | 0.1952 |
| Zygote | 0.3608 |
| Zygote | 0.1463 |
| Zygote | 0.1388 |
| Zygote | 0.4375 |
| Zygote | 0.3187 |
| 2-cell | 0.2379 |
| 2-cell | 0.2091 |
| 2-cell | 0.2354 |
| 2-cell | 0.4815 |
| 2-cell | 0.0512 |
| 2-cell | 0.0391 |
| 2-cell | 0.0 |
| 2-cell | 0.0 |
| 2-cell | 0.0363 |
| 4-cell | 0.2211 |
| 4-cell | 0.1055 |
| 4-cell | 0.5721 |
| 4-cell | 0.0169 |
| 4-cell | 0.046 |
| 4-cell | 0.1023 |
| 4-cell | 0.0588 |
| 4-cell | 0.1445 |
| 4-cell | 0.2543 |
| 4-cell | 0.0473 |
| 4-cell | 0.2198 |
| 4-cell | 0.6942 |
| 4-cell | 0.0318 |
| 4-cell | 0.0 |
| 4-cell | 0.0 |
| 4-cell | 0.0073 |
| 8-cell | 0.0112 |
| 8-cell | 0.0235 |
| 8-cell | 0.0161 |
| 8-cell | 0.0194 |
| 8-cell | 0.011 |
| 8-cell | 0.0567 |
| 8-cell | 0.0056 |
| 8-cell | 0.0836 |
| 8-cell | 0.4401 |
| 8-cell | 1.7546 |
| 8-cell | 0.0239 |
| 8-cell | 0.0 |
| 8-cell | 0.0 |
| 8-cell | 0.0197 |
| 8-cell | 0.0146 |
| 8-cell | 0.773 |
| 8-cell | 0.03 |
| 8-cell | 0.0053 |
| 8-cell | 0.0041 |
| 8-cell | 0.0091 |
| 8-cell | 0.0695 |
| 8-cell | 0.0 |
| 8-cell | 0.0169 |
| 8-cell | 0.0 |
| 8-cell | 0.0329 |
| 8-cell | 0.0 |
| Morulae | 0.012 |
| Morulae | 0.0 |
| Morulae | 0.0 |
| Morulae | 0.0137 |
| Morulae | 0.021 |
| Morulae | 0.0 |
| Morulae | 0.0073 |
| Morulae | 0.0 |
| Morulae | 0.0046 |
| Morulae | 0.0 |
| Morulae | 0.0 |
| Morulae | 0.0 |
| Morulae | 0.0053 |
| Morulae | 0.0 |
| Morulae | 0.0067 |
| Morulae | 0.0096 |
| Morulae | 0.0 |
| Morulae | 0.0 |
| Morulae | 0.0 |
| Late_blastocyst | 0.0159 |
| Late_blastocyst | 0.0083 |
| Late_blastocyst | 0.0107 |
| Late_blastocyst | 0.0049 |
| Late_blastocyst | 0.021 |
| Late_blastocyst | 0.0368 |
| Late_blastocyst | 0.0 |
| Late_blastocyst | 0.0189 |
| Late_blastocyst | 0.0166 |
| Late_blastocyst | 0.0199 |
| Late_blastocyst | 0.0099 |
| Late_blastocyst | 0.0 |
| Late_blastocyst | 0.0782 |
| Late_blastocyst | 0.9239 |
| Late_blastocyst | 0.0848 |
| Late_blastocyst | 0.0351 |
| Late_blastocyst | 0.0 |
| Late_blastocyst | 0.0128 |
| Late_blastocyst | 0.0192 |
| Late_blastocyst | 0.0 |
| Late_blastocyst | 0.0052 |
| Late_blastocyst | 0.0051 |
| Late_blastocyst | 0.0036 |
| Late_blastocyst | 0.0 |
| Late_blastocyst | 0.0178 |
| Late_blastocyst | 8.4583 |
| Late_blastocyst | 6.4488 |
| Late_blastocyst | 0.0 |
| Late_blastocyst | 0.0045 |
| Late_blastocyst | 0.0 |
| hESC_P0 | 0.9137 |
| hESC_P0 | 1.1702 |
| hESC_P0 | 2.8818 |
| hESC_P0 | 3.3313 |
| hESC_P0 | 1.5542 |
| hESC_P0 | 3.0977 |
| hESC_P0 | 2.2349 |
| hESC_P0 | 1.6201 |
| hESC_P10 | 0.0678 |
| hESC_P10 | 0.0846 |
| hESC_P10 | 0.046 |
| hESC_P10 | 0.0462 |
| hESC_P10 | 0.1642 |
| hESC_P10 | 0.0162 |
| hESC_P10 | 0.0322 |
| hESC_P10 | 0.4579 |
| hESC_P10 | 0.0055 |
| hESC_P10 | 0.042 |
| hESC_P10 | 0.0 |
| hESC_P10 | 0.0317 |
| hESC_P10 | 0.0629 |
| hESC_P10 | 0.0247 |
| hESC_P10 | 0.0556 |
| hESC_P10 | 0.0253 |
| hESC_P10 | 0.0584 |
| hESC_P10 | 0.0619 |
| hESC_P10 | 0.5711 |
| hESC_P10 | 0.0306 |
| hESC_P10 | 0.0638 |
| hESC_P10 | 0.0264 |
| hESC_P10 | 0.0566 |
| hESC_P10 | 0.034 |
| hESC_P10 | 0.0113 |
| hESC_P10 | 0.0066 |

## Slide 9
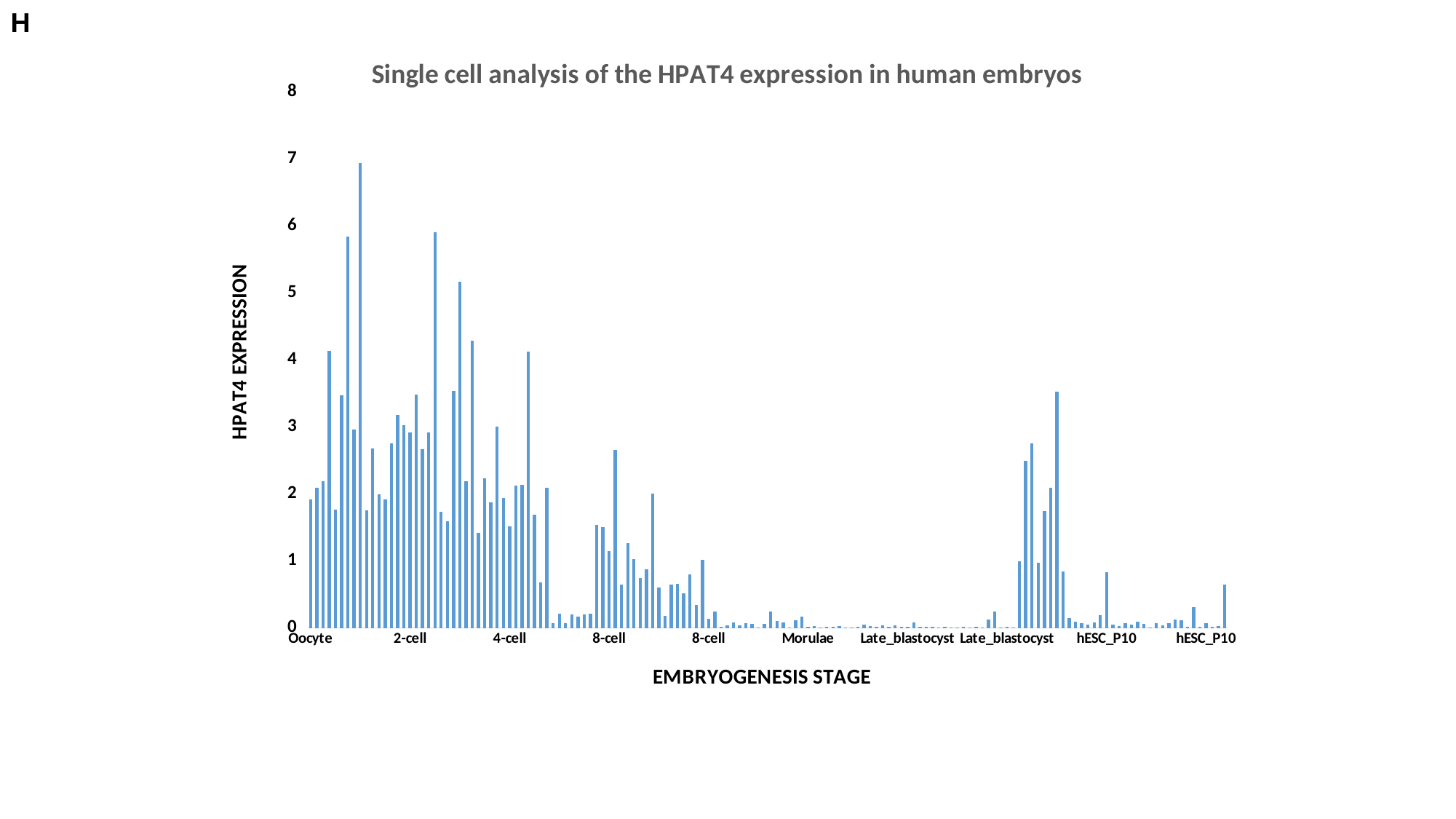

H
### Chart: Single cell analysis of the HPAT4 expression in human embryos
| Category | HPAT4 |
|---|---|
| Oocyte | 1.9085 |
| Oocyte | 2.0855 |
| Oocyte | 2.1885 |
| Oocyte | 4.1314 |
| Oocyte | 1.7603 |
| Oocyte | 3.4692 |
| Pronuclei | 5.8395 |
| Pronuclei | 2.9509 |
| Pronuclei | 6.9355 |
| Zygote | 1.7478 |
| Zygote | 2.6704 |
| Zygote | 1.992 |
| Zygote | 1.9111 |
| Zygote | 2.7441 |
| 2-cell | 3.1756 |
| 2-cell | 3.0207 |
| 2-cell | 2.9151 |
| 2-cell | 3.4807 |
| 2-cell | 2.6569 |
| 2-cell | 2.9126 |
| 2-cell | 5.8993 |
| 2-cell | 1.7285 |
| 2-cell | 1.5892 |
| 4-cell | 3.5367 |
| 4-cell | 5.1662 |
| 4-cell | 2.1792 |
| 4-cell | 4.2844 |
| 4-cell | 1.4123 |
| 4-cell | 2.2251 |
| 4-cell | 1.8701 |
| 4-cell | 2.9985 |
| 4-cell | 1.9331 |
| 4-cell | 1.5141 |
| 4-cell | 2.1234 |
| 4-cell | 2.126 |
| 4-cell | 4.1214 |
| 4-cell | 1.6816 |
| 4-cell | 0.676 |
| 4-cell | 2.085 |
| 8-cell | 0.0608 |
| 8-cell | 0.2082 |
| 8-cell | 0.0632 |
| 8-cell | 0.1991 |
| 8-cell | 0.1642 |
| 8-cell | 0.1944 |
| 8-cell | 0.2026 |
| 8-cell | 1.531 |
| 8-cell | 1.5027 |
| 8-cell | 1.1432 |
| 8-cell | 2.6523 |
| 8-cell | 0.6399 |
| 8-cell | 1.2588 |
| 8-cell | 1.0211 |
| 8-cell | 0.7406 |
| 8-cell | 0.8638 |
| 8-cell | 2.0008 |
| 8-cell | 0.5975 |
| 8-cell | 0.1756 |
| 8-cell | 0.6378 |
| 8-cell | 0.6535 |
| 8-cell | 0.5055 |
| 8-cell | 0.7915 |
| 8-cell | 0.3348 |
| 8-cell | 1.0112 |
| 8-cell | 0.1245 |
| Morulae | 0.2356 |
| Morulae | 0.0113 |
| Morulae | 0.029 |
| Morulae | 0.0745 |
| Morulae | 0.0286 |
| Morulae | 0.0603 |
| Morulae | 0.0532 |
| Morulae | 0.0 |
| Morulae | 0.0538 |
| Morulae | 0.2353 |
| Morulae | 0.0965 |
| Morulae | 0.0709 |
| Morulae | 0.0 |
| Morulae | 0.1064 |
| Morulae | 0.1572 |
| Morulae | 0.0131 |
| Morulae | 0.0206 |
| Morulae | 0.0 |
| Morulae | 0.0147 |
| Late_blastocyst | 0.0072 |
| Late_blastocyst | 0.0227 |
| Late_blastocyst | 0.0 |
| Late_blastocyst | 0.0 |
| Late_blastocyst | 0.0143 |
| Late_blastocyst | 0.0401 |
| Late_blastocyst | 0.0249 |
| Late_blastocyst | 0.0057 |
| Late_blastocyst | 0.027 |
| Late_blastocyst | 0.012 |
| Late_blastocyst | 0.0269 |
| Late_blastocyst | 0.0073 |
| Late_blastocyst | 0.0109 |
| Late_blastocyst | 0.0793 |
| Late_blastocyst | 0.007 |
| Late_blastocyst | 0.0127 |
| Late_blastocyst | 0.0063 |
| Late_blastocyst | 0.0 |
| Late_blastocyst | 0.0087 |
| Late_blastocyst | 0.0 |
| Late_blastocyst | 0.0 |
| Late_blastocyst | 0.0093 |
| Late_blastocyst | 0.0 |
| Late_blastocyst | 0.0121 |
| Late_blastocyst | 0.0 |
| Late_blastocyst | 0.118 |
| Late_blastocyst | 0.24 |
| Late_blastocyst | 0.0 |
| Late_blastocyst | 0.0123 |
| Late_blastocyst | 0.0034 |
| hESC_P0 | 0.9847 |
| hESC_P0 | 2.488 |
| hESC_P0 | 2.7515 |
| hESC_P0 | 0.9634 |
| hESC_P0 | 1.735 |
| hESC_P0 | 2.0837 |
| hESC_P0 | 3.5236 |
| hESC_P0 | 0.8345 |
| hESC_P10 | 0.1347 |
| hESC_P10 | 0.0883 |
| hESC_P10 | 0.065 |
| hESC_P10 | 0.0419 |
| hESC_P10 | 0.0723 |
| hESC_P10 | 0.1857 |
| hESC_P10 | 0.8251 |
| hESC_P10 | 0.039 |
| hESC_P10 | 0.015 |
| hESC_P10 | 0.0667 |
| hESC_P10 | 0.0375 |
| hESC_P10 | 0.0863 |
| hESC_P10 | 0.0571 |
| hESC_P10 | 0.0 |
| hESC_P10 | 0.0616 |
| hESC_P10 | 0.0275 |
| hESC_P10 | 0.0636 |
| hESC_P10 | 0.1166 |
| hESC_P10 | 0.1054 |
| hESC_P10 | 0.0056 |
| hESC_P10 | 0.3052 |
| hESC_P10 | 0.012 |
| hESC_P10 | 0.0654 |
| hESC_P10 | 0.0103 |
| hESC_P10 | 0.0206 |
| hESC_P10 | 0.6414 |

## Slide 10
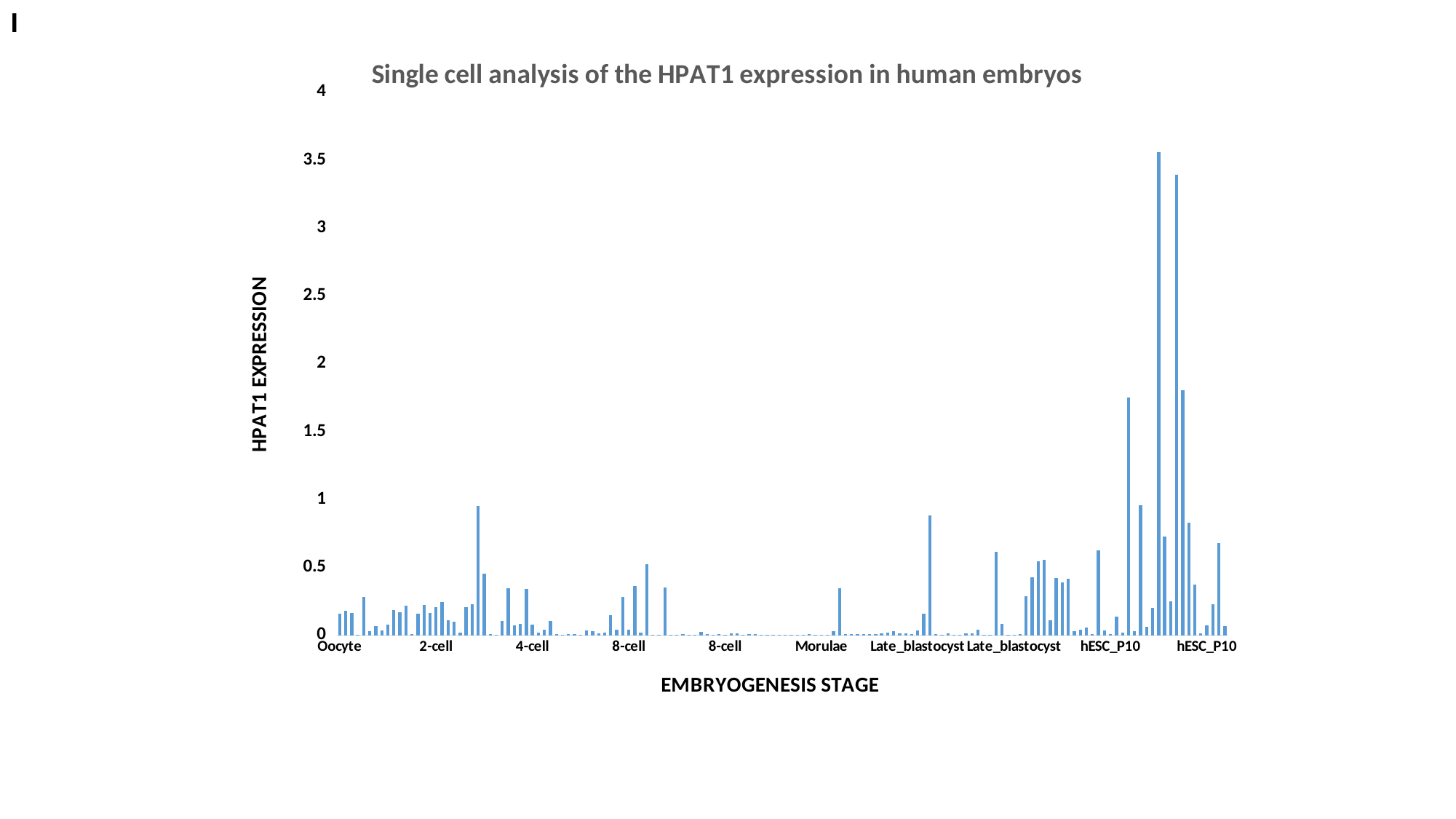

I
### Chart: Single cell analysis of the HPAT1 expression in human embryos
| Category | HPAT1 |
|---|---|
| Oocyte | 0.1566 |
| Oocyte | 0.1797 |
| Oocyte | 0.1601 |
| Oocyte | 0.0 |
| Oocyte | 0.2815 |
| Oocyte | 0.028 |
| Pronuclei | 0.0666 |
| Pronuclei | 0.0329 |
| Pronuclei | 0.0758 |
| Zygote | 0.1802 |
| Zygote | 0.1678 |
| Zygote | 0.2123 |
| Zygote | 0.0074 |
| Zygote | 0.1549 |
| 2-cell | 0.2202 |
| 2-cell | 0.1609 |
| 2-cell | 0.2062 |
| 2-cell | 0.2439 |
| 2-cell | 0.1095 |
| 2-cell | 0.0992 |
| 2-cell | 0.0159 |
| 2-cell | 0.2035 |
| 2-cell | 0.2266 |
| 4-cell | 0.9488 |
| 4-cell | 0.4488 |
| 4-cell | 0.0038 |
| 4-cell | 0.0 |
| 4-cell | 0.1012 |
| 4-cell | 0.3428 |
| 4-cell | 0.0697 |
| 4-cell | 0.0821 |
| 4-cell | 0.3398 |
| 4-cell | 0.0776 |
| 4-cell | 0.0183 |
| 4-cell | 0.0397 |
| 4-cell | 0.1043 |
| 4-cell | 0.0043 |
| 4-cell | 0.0 |
| 4-cell | 0.0073 |
| 8-cell | 0.0056 |
| 8-cell | 0.0 |
| 8-cell | 0.0321 |
| 8-cell | 0.0258 |
| 8-cell | 0.011 |
| 8-cell | 0.0189 |
| 8-cell | 0.1449 |
| 8-cell | 0.0365 |
| 8-cell | 0.2802 |
| 8-cell | 0.036 |
| 8-cell | 0.358 |
| 8-cell | 0.0148 |
| 8-cell | 0.5222 |
| 8-cell | 0.0 |
| 8-cell | 0.0 |
| 8-cell | 0.3487 |
| 8-cell | 0.0 |
| 8-cell | 0.0 |
| 8-cell | 0.0041 |
| 8-cell | 0.0 |
| 8-cell | 0.0 |
| 8-cell | 0.0238 |
| 8-cell | 0.0084 |
| 8-cell | 0.0 |
| 8-cell | 0.0041 |
| 8-cell | 0.0 |
| Morulae | 0.012 |
| Morulae | 0.0124 |
| Morulae | 0.0 |
| Morulae | 0.0046 |
| Morulae | 0.0052 |
| Morulae | 0.0 |
| Morulae | 0.0 |
| Morulae | 0.0 |
| Morulae | 0.0 |
| Morulae | 0.0 |
| Morulae | 0.0 |
| Morulae | 0.0 |
| Morulae | 0.0 |
| Morulae | 0.0049 |
| Morulae | 0.0 |
| Morulae | 0.0 |
| Morulae | 0.0 |
| Morulae | 0.0272 |
| Morulae | 0.3434 |
| Late_blastocyst | 0.0079 |
| Late_blastocyst | 0.0083 |
| Late_blastocyst | 0.0053 |
| Late_blastocyst | 0.0048 |
| Late_blastocyst | 0.0053 |
| Late_blastocyst | 0.0074 |
| Late_blastocyst | 0.0109 |
| Late_blastocyst | 0.0189 |
| Late_blastocyst | 0.0265 |
| Late_blastocyst | 0.0133 |
| Late_blastocyst | 0.0099 |
| Late_blastocyst | 0.0081 |
| Late_blastocyst | 0.03 |
| Late_blastocyst | 0.1559 |
| Late_blastocyst | 0.8782 |
| Late_blastocyst | 0.007 |
| Late_blastocyst | 0.0 |
| Late_blastocyst | 0.0128 |
| Late_blastocyst | 0.0 |
| Late_blastocyst | 0.0 |
| Late_blastocyst | 0.0105 |
| Late_blastocyst | 0.0102 |
| Late_blastocyst | 0.0363 |
| Late_blastocyst | 0.0 |
| Late_blastocyst | 0.0 |
| Late_blastocyst | 0.6111 |
| Late_blastocyst | 0.0809 |
| Late_blastocyst | 0.0 |
| Late_blastocyst | 0.0 |
| Late_blastocyst | 0.0037 |
| hESC_P0 | 0.2852 |
| hESC_P0 | 0.4246 |
| hESC_P0 | 0.5406 |
| hESC_P0 | 0.5535 |
| hESC_P0 | 0.1093 |
| hESC_P0 | 0.4186 |
| hESC_P0 | 0.389 |
| hESC_P0 | 0.4112 |
| hESC_P10 | 0.0296 |
| hESC_P10 | 0.038 |
| hESC_P10 | 0.0562 |
| hESC_P10 | 0.0051 |
| hESC_P10 | 0.6215 |
| hESC_P10 | 0.0323 |
| hESC_P10 | 0.0064 |
| hESC_P10 | 0.1358 |
| hESC_P10 | 0.0165 |
| hESC_P10 | 1.7504 |
| hESC_P10 | 0.0275 |
| hESC_P10 | 0.9551 |
| hESC_P10 | 0.0571 |
| hESC_P10 | 0.1978 |
| hESC_P10 | 3.5528 |
| hESC_P10 | 0.7222 |
| hESC_P10 | 0.2451 |
| hESC_P10 | 3.3899 |
| hESC_P10 | 1.8035 |
| hESC_P10 | 0.8249 |
| hESC_P10 | 0.3707 |
| hESC_P10 | 0.0132 |
| hESC_P10 | 0.0719 |
| hESC_P10 | 0.2264 |
| hESC_P10 | 0.6733 |
| hESC_P10 | 0.066 |

## Slide 11
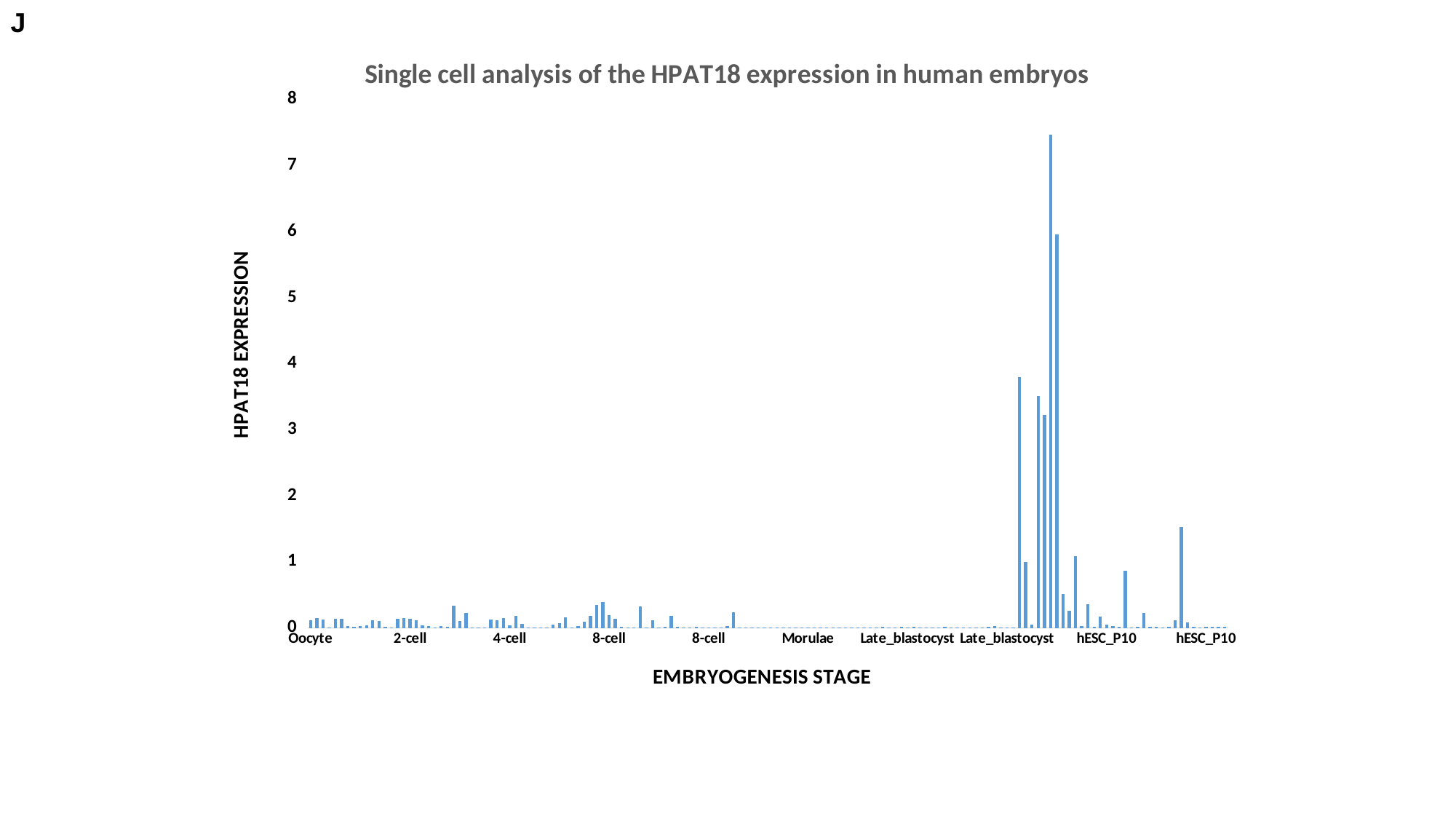

J
### Chart: Single cell analysis of the HPAT18 expression in human embryos
| Category | HPAT17&18 |
|---|---|
| Oocyte | 0.1114 |
| Oocyte | 0.1444 |
| Oocyte | 0.1166 |
| Oocyte | 0.0041 |
| Oocyte | 0.1263 |
| Oocyte | 0.1297 |
| Pronuclei | 0.0196 |
| Pronuclei | 0.0124 |
| Pronuclei | 0.0171 |
| Zygote | 0.0335 |
| Zygote | 0.1139 |
| Zygote | 0.0968 |
| Zygote | 0.0048 |
| Zygote | 0.0 |
| 2-cell | 0.1366 |
| 2-cell | 0.1471 |
| 2-cell | 0.1259 |
| 2-cell | 0.1106 |
| 2-cell | 0.0329 |
| 2-cell | 0.0181 |
| 2-cell | 0.0 |
| 2-cell | 0.0203 |
| 2-cell | 0.0058 |
| 4-cell | 0.3282 |
| 4-cell | 0.0989 |
| 4-cell | 0.2245 |
| 4-cell | 0.0024 |
| 4-cell | 0.0026 |
| 4-cell | 0.0 |
| 4-cell | 0.1182 |
| 4-cell | 0.1082 |
| 4-cell | 0.1445 |
| 4-cell | 0.0304 |
| 4-cell | 0.1788 |
| 4-cell | 0.0577 |
| 4-cell | 0.0039 |
| 4-cell | 0.0018 |
| 4-cell | 0.0 |
| 4-cell | 0.0 |
| 8-cell | 0.0455 |
| 8-cell | 0.0681 |
| 8-cell | 0.1516 |
| 8-cell | 0.0 |
| 8-cell | 0.0212 |
| 8-cell | 0.0865 |
| 8-cell | 0.1772 |
| 8-cell | 0.3407 |
| 8-cell | 0.3895 |
| 8-cell | 0.1892 |
| 8-cell | 0.1367 |
| 8-cell | 0.0096 |
| 8-cell | 0.0018 |
| 8-cell | 0.0 |
| 8-cell | 0.3189 |
| 8-cell | 0.0018 |
| 8-cell | 0.1094 |
| 8-cell | 0.0023 |
| 8-cell | 0.0071 |
| 8-cell | 0.1723 |
| 8-cell | 0.0085 |
| 8-cell | 0.0 |
| 8-cell | 0.0 |
| 8-cell | 0.007 |
| 8-cell | 0.0 |
| 8-cell | 0.0 |
| Morulae | 0.0 |
| Morulae | 0.0 |
| Morulae | 0.0165 |
| Morulae | 0.2328 |
| Morulae | 0.0 |
| Morulae | 0.0026 |
| Morulae | 0.0 |
| Morulae | 0.0026 |
| Morulae | 0.0039 |
| Morulae | 0.0 |
| Morulae | 0.0 |
| Morulae | 0.0019 |
| Morulae | 0.0 |
| Morulae | 0.0 |
| Morulae | 0.0029 |
| Morulae | 0.0021 |
| Morulae | 0.0 |
| Morulae | 0.0 |
| Morulae | 0.0 |
| Late_blastocyst | 0.0 |
| Late_blastocyst | 0.0 |
| Late_blastocyst | 0.0 |
| Late_blastocyst | 0.0 |
| Late_blastocyst | 0.0 |
| Late_blastocyst | 0.0032 |
| Late_blastocyst | 0.0024 |
| Late_blastocyst | 0.0 |
| Late_blastocyst | 0.0085 |
| Late_blastocyst | 0.0 |
| Late_blastocyst | 0.0 |
| Late_blastocyst | 0.0069 |
| Late_blastocyst | 0.0026 |
| Late_blastocyst | 0.0107 |
| Late_blastocyst | 0.0 |
| Late_blastocyst | 0.003 |
| Late_blastocyst | 0.003 |
| Late_blastocyst | 0.0 |
| Late_blastocyst | 0.0083 |
| Late_blastocyst | 0.0034 |
| Late_blastocyst | 0.0022 |
| Late_blastocyst | 0.0022 |
| Late_blastocyst | 0.0016 |
| Late_blastocyst | 0.0 |
| Late_blastocyst | 0.0026 |
| Late_blastocyst | 0.0103 |
| Late_blastocyst | 0.0208 |
| Late_blastocyst | 0.0 |
| Late_blastocyst | 0.0 |
| Late_blastocyst | 0.0 |
| hESC_P0 | 3.7958 |
| hESC_P0 | 0.9961 |
| hESC_P0 | 0.0392 |
| hESC_P0 | 3.5065 |
| hESC_P0 | 3.222 |
| hESC_P0 | 7.4595 |
| hESC_P0 | 5.947 |
| hESC_P0 | 0.5073 |
| hESC_P10 | 0.2492 |
| hESC_P10 | 1.0835 |
| hESC_P10 | 0.0176 |
| hESC_P10 | 0.3481 |
| hESC_P10 | 0.015 |
| hESC_P10 | 0.1641 |
| hESC_P10 | 0.0387 |
| hESC_P10 | 0.0184 |
| hESC_P10 | 0.0071 |
| hESC_P10 | 0.8623 |
| hESC_P10 | 0.0039 |
| hESC_P10 | 0.0068 |
| hESC_P10 | 0.2209 |
| hESC_P10 | 0.0106 |
| hESC_P10 | 0.0106 |
| hESC_P10 | 0.0 |
| hESC_P10 | 0.01 |
| hESC_P10 | 0.1042 |
| hESC_P10 | 1.5245 |
| hESC_P10 | 0.0735 |
| hESC_P10 | 0.0075 |
| hESC_P10 | 0.0 |
| hESC_P10 | 0.0088 |
| hESC_P10 | 0.0073 |
| hESC_P10 | 0.0073 |
| hESC_P10 | 0.0085 |
