# Supplementary material for: Single cell expression analysis of primate-specific retroviruses-derived HPAT lincRNAs in viable human blastocysts identifies embryonic cells co-expressing genetic markers of multiple lineages
Source: Heliyon. 2018 Jun 28;4(6):e00667. doi: 10.1016/j.heliyon.2018.e00667 (PMC6039856; doi:10.1016/j.heliyon.2018.e00667)
Supplement: Supplemental Figure S3 [file mmc6.pptx]

## Slide 1
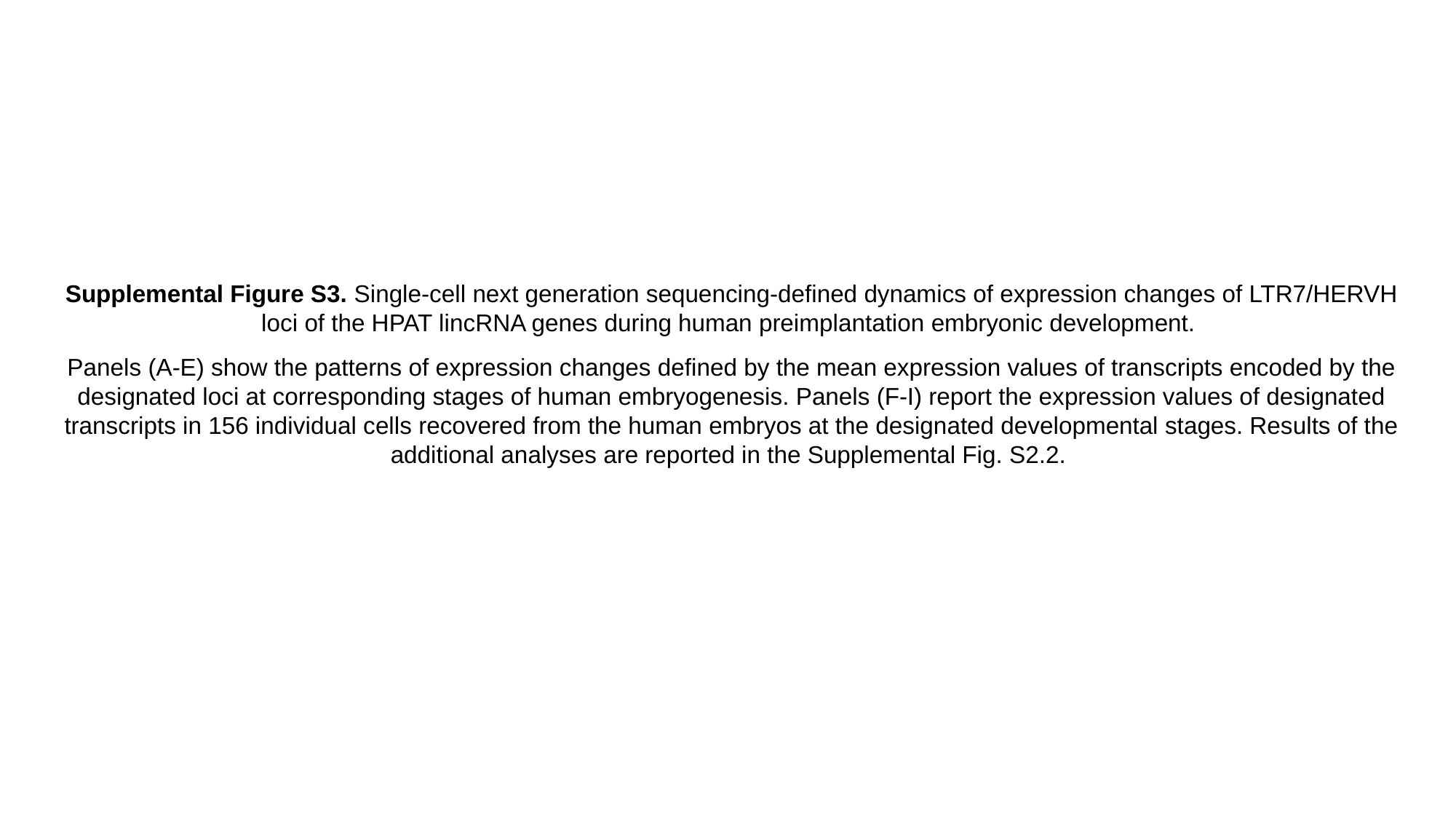

Supplemental Figure S3. Single-cell next generation sequencing-defined dynamics of expression changes of LTR7/HERVH loci of the HPAT lincRNA genes during human preimplantation embryonic development.
Panels (A-E) show the patterns of expression changes defined by the mean expression values of transcripts encoded by the designated loci at corresponding stages of human embryogenesis. Panels (F-I) report the expression values of designated transcripts in 156 individual cells recovered from the human embryos at the designated developmental stages. Results of the additional analyses are reported in the Supplemental Fig. S2.2.

## Slide 2
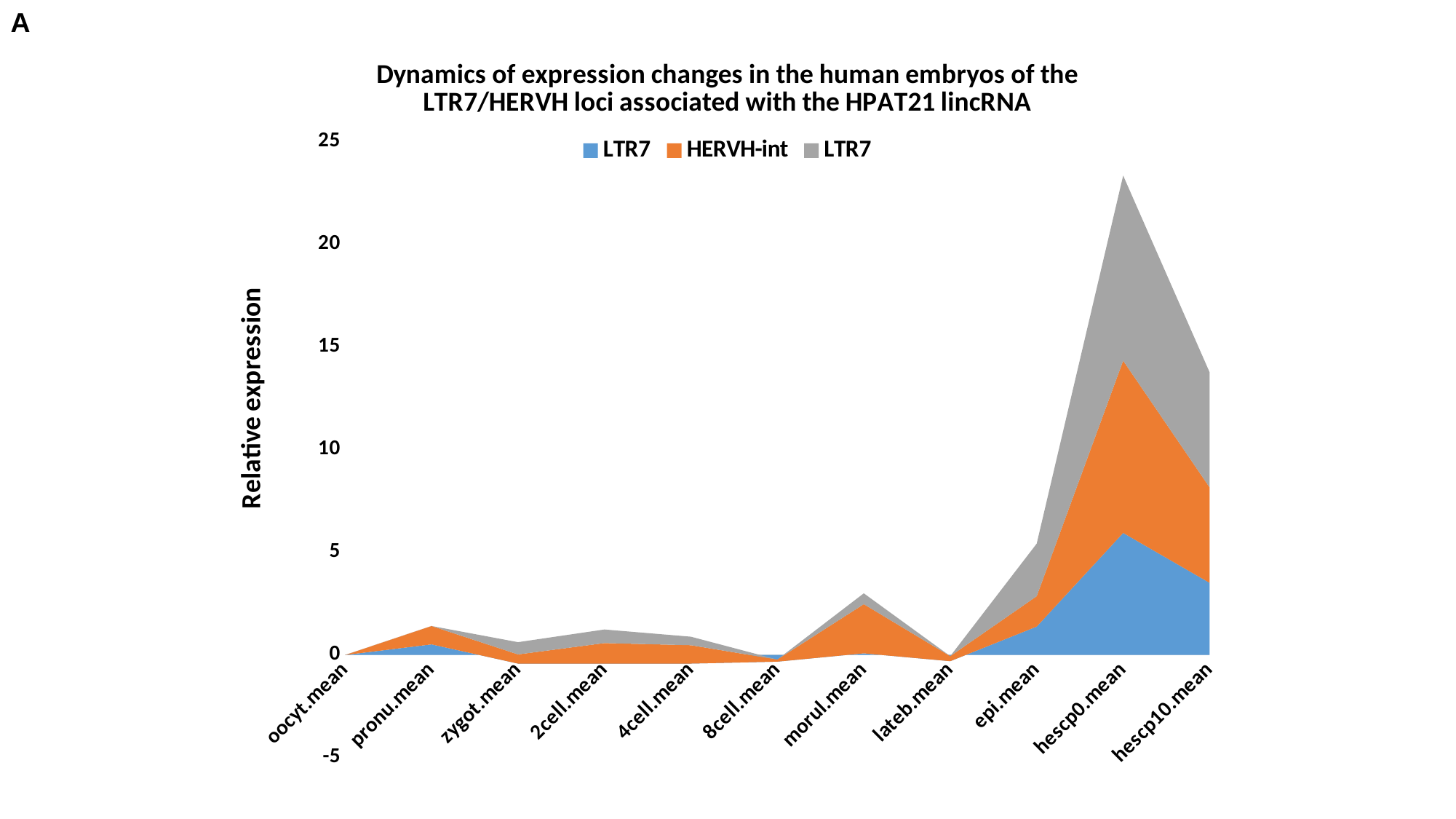

A
### Chart: Dynamics of expression changes in the human embryos of the LTR7/HERVH loci associated with the HPAT21 lincRNA
| Category | LTR7 | HERVH-int | LTR7 |
|---|---|---|---|
| oocyt.mean | 0.0 | 0.0 | 0.0 |
| pronu.mean | 0.5166851537634796 | 0.8942370444558203 | 0.0 |
| zygot.mean | -0.4183666325841404 | 0.4451473848932199 | 0.5967889766402701 |
| 2cell.mean | -0.4183666325841404 | 0.99786871316496 | 0.6605412346450801 |
| 4cell.mean | -0.4183666325841404 | 0.8936306415863502 | 0.41425877005 |
| 8cell.mean | -0.3252973860621102 | 0.10232111929733989 | 0.0 |
| morul.mean | 0.08001199468669995 | 2.3944947144098 | 0.5263116054409096 |
| lateb.mean | -0.3012654004440103 | 0.24437283649330022 | 0.0 |
| epi.mean | 1.3836050916376594 | 1.47805502213668 | 2.55860656431692 |
| hescp0.mean | 5.9430955596768795 | 8.38660566752811 | 9.013640449338691 |
| hescp10.mean | 3.51426677188912 | 4.646295929366202 | 5.6078361877576 |

## Slide 3
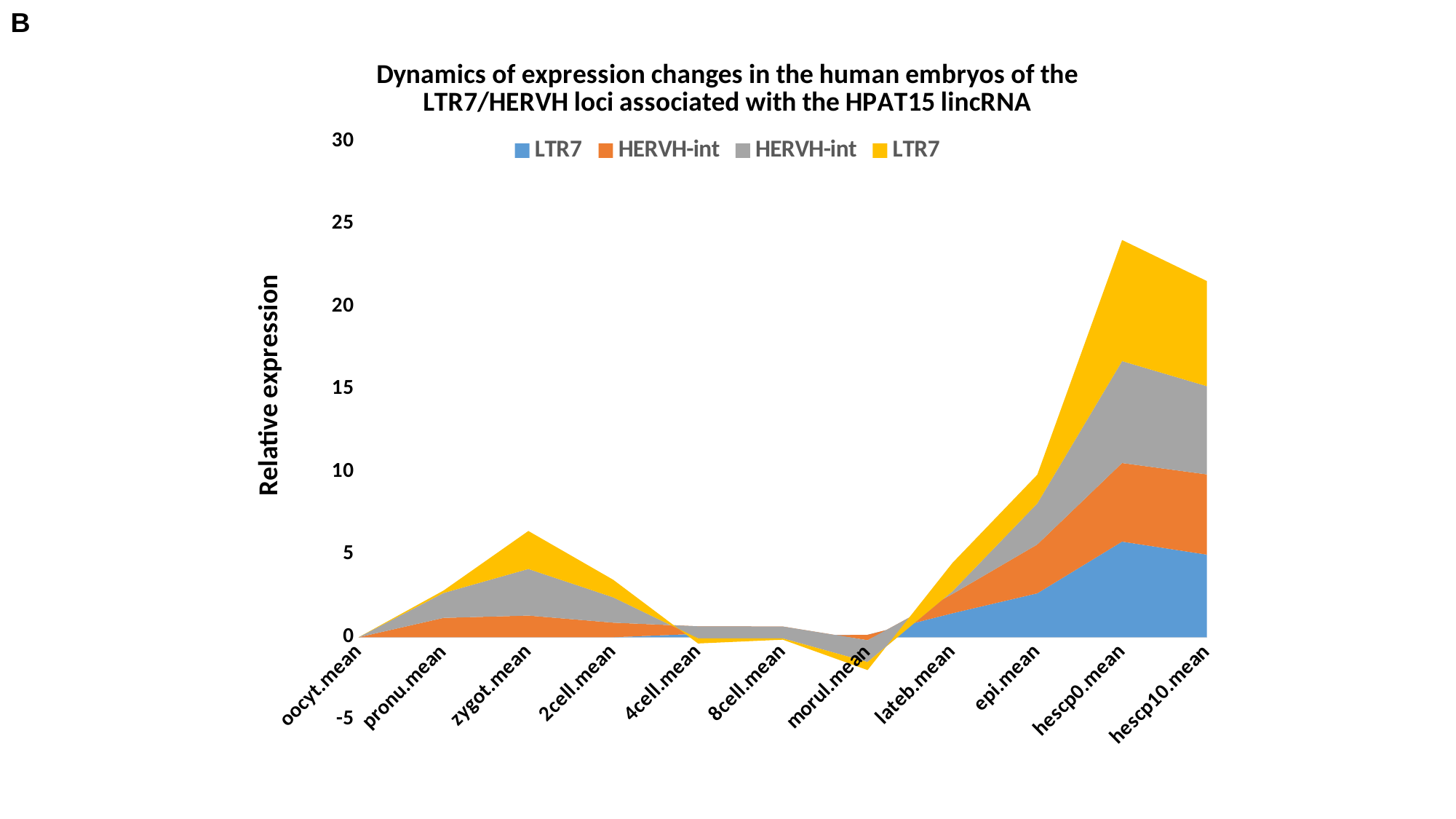

B
### Chart: Dynamics of expression changes in the human embryos of the LTR7/HERVH loci associated with the HPAT15 lincRNA
| Category | LTR7 | HERVH-int | HERVH-int | LTR7 |
|---|---|---|---|---|
| oocyt.mean | 0.0 | 0.0 | 0.0 | 0.0 |
| pronu.mean | 0.0 | 1.1699567824494501 | 1.5068760731872852 | 0.14889887885977027 |
| zygot.mean | 0.0 | 1.3111433366066119 | 2.8196310817214343 | 2.29908602907853 |
| 2cell.mean | 0.0 | 0.880475023550423 | 1.5327549100962852 | 1.0640480932912402 |
| 4cell.mean | 0.2042371416808999 | 0.454999972508358 | -0.7250060229739597 | -0.31218952786524 |
| 8cell.mean | 0.09403965323714036 | 0.5530207378506515 | -0.7210839926366597 | -0.07533316324376971 |
| morul.mean | 0.14470392093329032 | -0.32609247543032804 | -1.3217041468464998 | -0.4849515840753096 |
| lateb.mean | 1.44852816765522 | 1.164932703950904 | 0.16379071606592 | 1.7193492213019101 |
| epi.mean | 2.65669729940221 | 2.955576057697575 | 2.484254371794755 | 1.73469179261081 |
| hescp0.mean | 5.786574708566921 | 4.756704715001854 | 6.17016358898832 | 7.326684493187271 |
| hescp10.mean | 4.996692854078634 | 4.856272914379986 | 5.341205839947181 | 6.36124276230323 |

## Slide 4
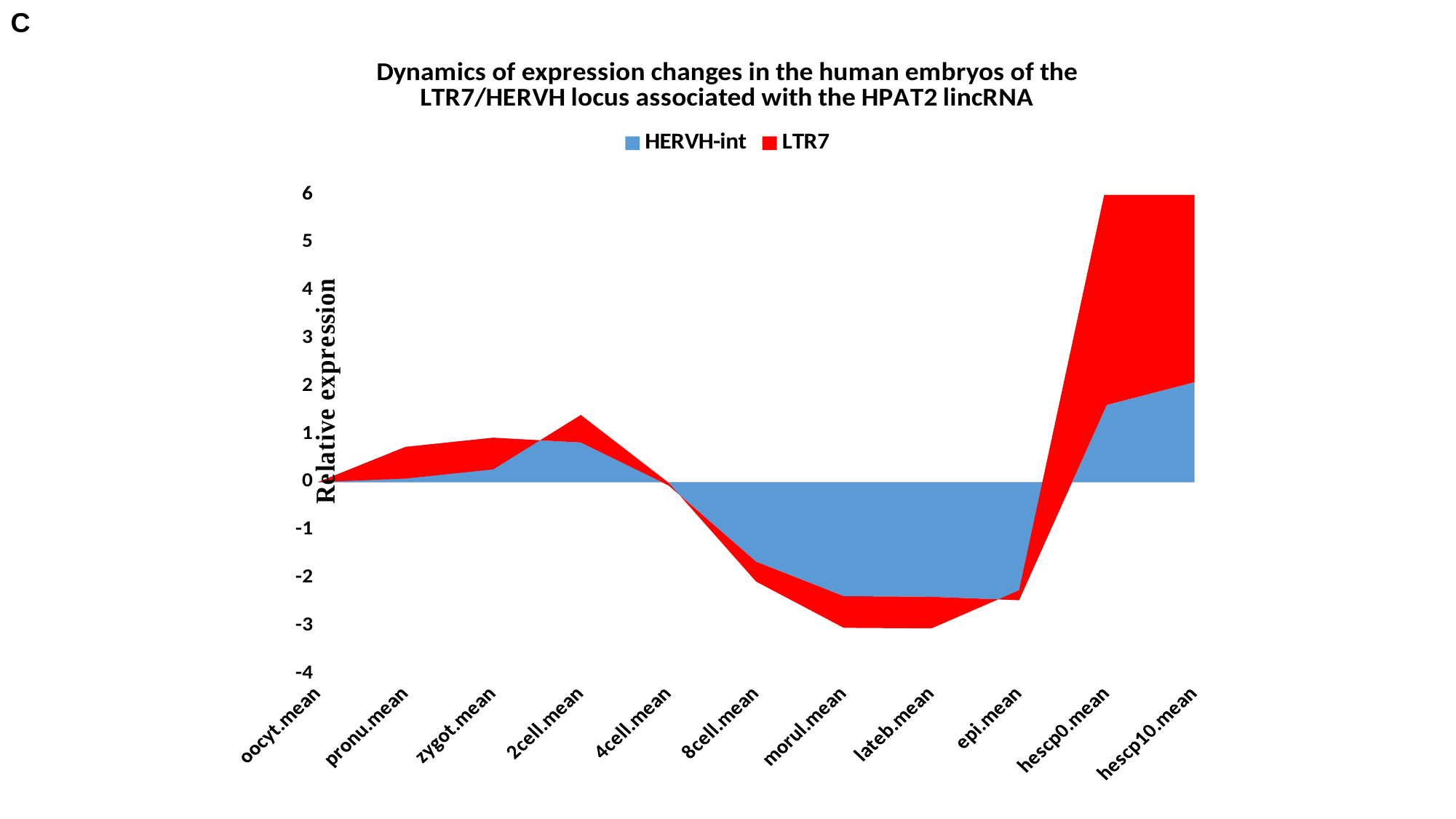

C
### Chart: Dynamics of expression changes in the human embryos of the LTR7/HERVH locus associated with the HPAT2 lincRNA
| Category | HERVH-int | LTR7 |
|---|---|---|
| oocyt.mean | 0.0 | 0.0 |
| pronu.mean | 0.7372353612034501 | -0.6603312634996898 |
| zygot.mean | 0.9281649313029801 | -0.6603312634996898 |
| 2cell.mean | 0.8288308127875401 | 0.57474505124287 |
| 4cell.mean | -0.06627424715330976 | 0.056632685510300096 |
| 8cell.mean | -1.65982009983416 | -0.41427658195348993 |
| morul.mean | -2.3793025784921 | -0.6603312634996898 |
| lateb.mean | -2.3919075195442496 | -0.6603312634996898 |
| epi.mean | -2.46167074221222 | 0.20601228032317032 |
| hescp0.mean | 1.6141311895834312 | 4.639797918600243 |
| hescp10.mean | 2.0868718902749697 | 4.261065743094067 |

## Slide 5
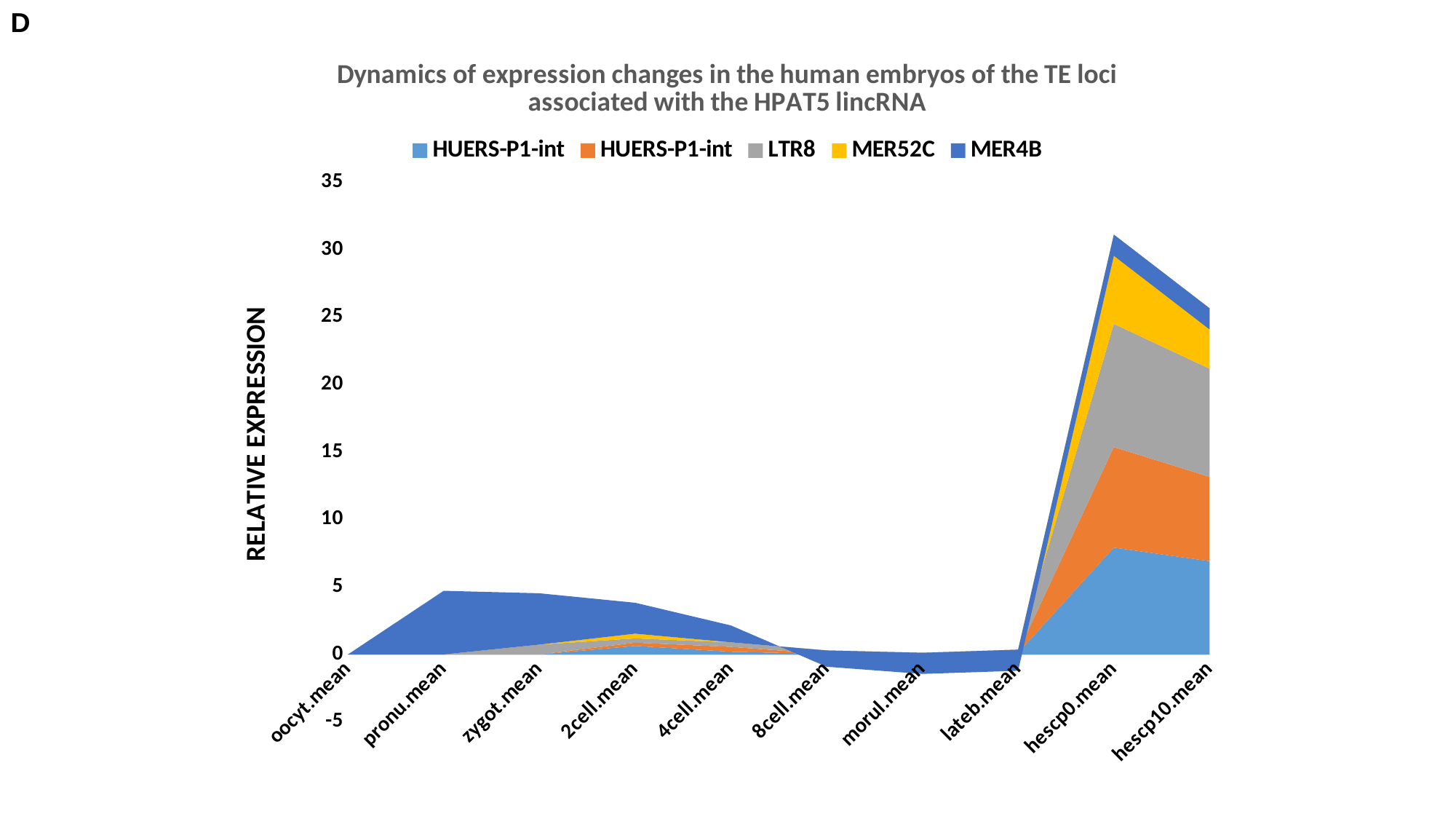

D
### Chart: Dynamics of expression changes in the human embryos of the TE loci associated with the HPAT5 lincRNA
| Category | HUERS-P1-int | HUERS-P1-int | LTR8 | MER52C | MER4B |
|---|---|---|---|---|---|
| oocyt.mean | 0.0 | 0.0 | 0.0 | 0.0 | 0.0 |
| pronu.mean | 0.0 | 0.0 | 0.0 | 0.0 | 4.71464238150748 |
| zygot.mean | 0.0 | 0.0 | 0.72947104029772 | 0.0 | 3.800019195405239 |
| 2cell.mean | 0.6286830194474899 | 0.24192878636652004 | 0.31720530682891024 | 0.3494744548618396 | 2.290329586037366 |
| 4cell.mean | 0.19916763429088036 | 0.36059493299906986 | 0.3362276924725798 | 0.0 | 1.2514314933733302 |
| 8cell.mean | 0.033992637537820336 | 0.0 | 0.2620350779427598 | 0.0 | -1.2094983875448904 |
| morul.mean | 0.12049616316086986 | 0.0 | 0.0 | 0.0 | -1.56616735357788 |
| lateb.mean | 0.1116209970621096 | 0.07068782038798016 | 0.17005234476341968 | 0.0 | -1.56616735357788 |
| hescp0.mean | 7.9182205503080905 | 7.44964915793238 | 9.13341187185199 | 6.61838107771226 | -1.56616735357788 |
| hescp10.mean | 6.91441785865231 | 6.24567985104834 | 8.01679832324091 | 4.480564194797659 | -1.56616735357788 |

## Slide 6
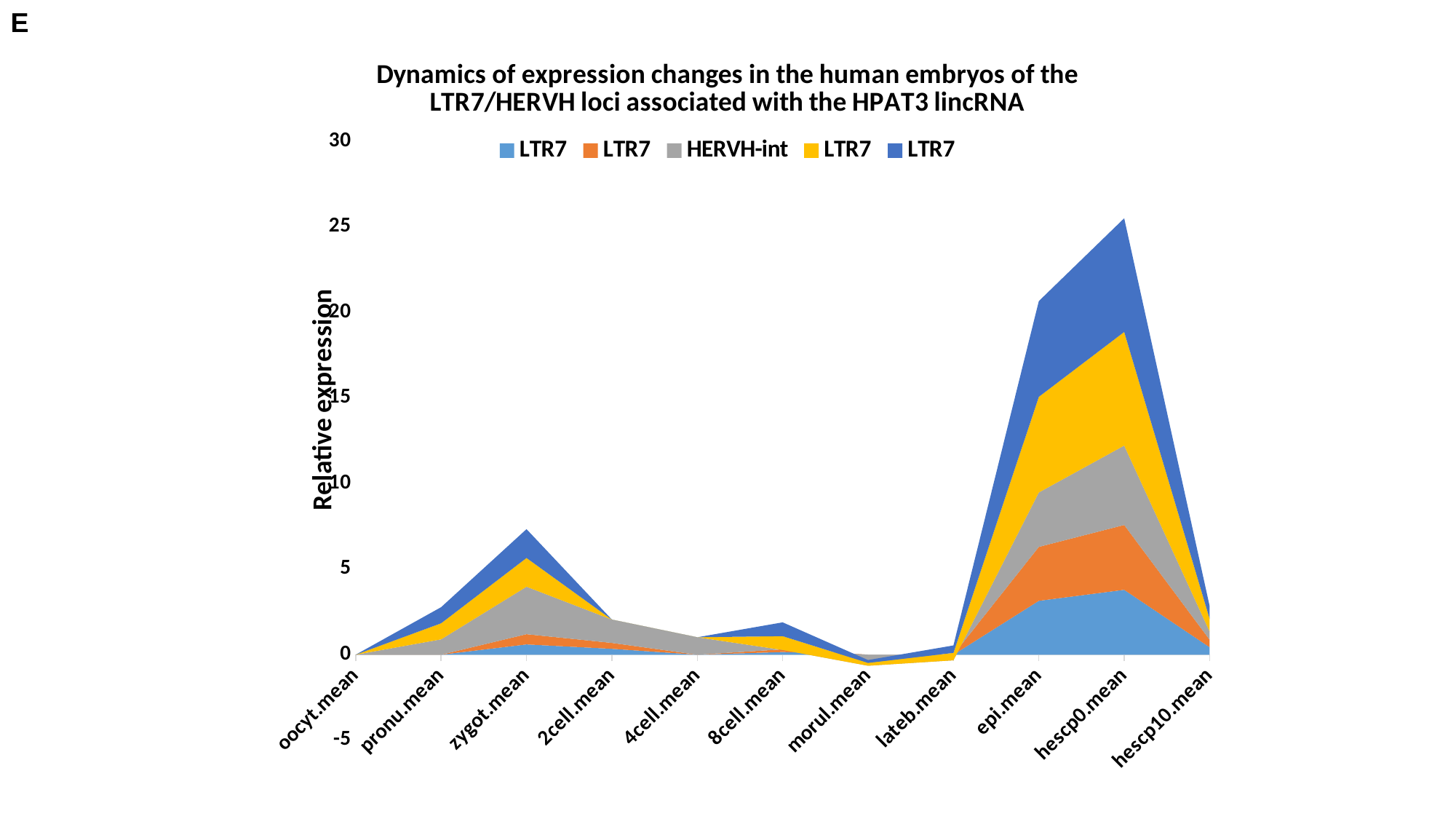

E
### Chart: Dynamics of expression changes in the human embryos of the LTR7/HERVH loci associated with the HPAT3 lincRNA
| Category | LTR7 | LTR7 | HERVH-int | LTR7 | LTR7 |
|---|---|---|---|---|---|
| oocyt.mean | 0.0 | 0.0 | 0.0 | 0.0 | 0.0 |
| pronu.mean | 0.0 | 0.0 | 0.8969951144164496 | 0.93968206367474 | 0.93968206367474 |
| zygot.mean | 0.5997275716995203 | 0.5997275716995203 | 2.775027807908904 | 1.6817367221719501 | 1.6817367221719501 |
| 2cell.mean | 0.34456147083700017 | 0.34456147083700017 | 1.3621477858184896 | 0.0 | 0.0 |
| 4cell.mean | 0.0 | 0.0 | 1.0181199821276397 | 0.0 | 0.0 |
| 8cell.mean | 0.15662512075869017 | 0.15662512075869017 | -0.03648838000356003 | 0.8063127927868101 | 0.8063127927868101 |
| morul.mean | 0.0 | 0.0 | -0.6455093052155498 | 0.16624951160414003 | 0.16624951160414003 |
| lateb.mean | 0.0 | 0.0 | -0.3284569692612802 | 0.4341337321143799 | 0.4341337321143799 |
| epi.mean | 3.14991478809289 | 3.14991478809289 | 3.1824717580117388 | 5.59536438662165 | 5.59536438662165 |
| hescp0.mean | 3.7918696220253842 | 3.7918696220253842 | 4.640141899546225 | 6.64610626027396 | 6.64610626027396 |
| hescp10.mean | 0.43688664829505974 | 0.43688664829505974 | 0.48214872162264966 | 0.7450702486512202 | 0.7450702486512202 |

## Slide 7
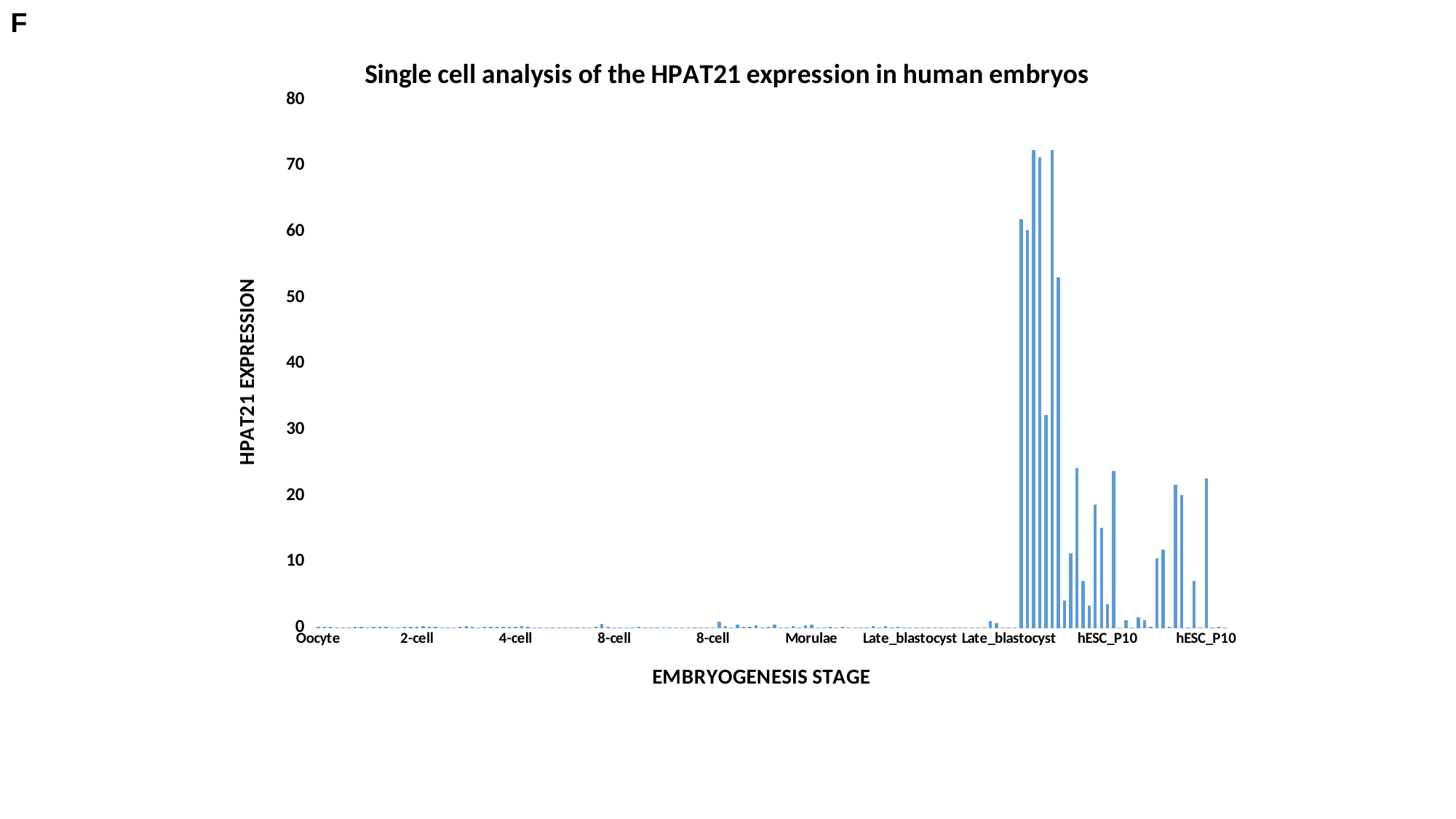

F
### Chart: Single cell analysis of the HPAT21 expression in human embryos
| Category | HPAT21 |
|---|---|
| Oocyte | 0.0887 |
| Oocyte | 0.1176 |
| Oocyte | 0.0606 |
| Oocyte | 0.0 |
| Oocyte | 0.008 |
| Oocyte | 0.0054 |
| Pronuclei | 0.1457 |
| Pronuclei | 0.0477 |
| Pronuclei | 0.0175 |
| Zygote | 0.0659 |
| Zygote | 0.0628 |
| Zygote | 0.067 |
| Zygote | 0.0036 |
| Zygote | 0.0 |
| 2-cell | 0.1149 |
| 2-cell | 0.0827 |
| 2-cell | 0.0629 |
| 2-cell | 0.167 |
| 2-cell | 0.0847 |
| 2-cell | 0.0639 |
| 2-cell | 0.0231 |
| 2-cell | 0.0137 |
| 2-cell | 0.0 |
| 4-cell | 0.126 |
| 4-cell | 0.2414 |
| 4-cell | 0.0981 |
| 4-cell | 0.0326 |
| 4-cell | 0.0919 |
| 4-cell | 0.1434 |
| 4-cell | 0.1383 |
| 4-cell | 0.1505 |
| 4-cell | 0.0925 |
| 4-cell | 0.0782 |
| 4-cell | 0.1812 |
| 4-cell | 0.1092 |
| 4-cell | 0.0044 |
| 4-cell | 0.0 |
| 4-cell | 0.0043 |
| 4-cell | 0.0 |
| 8-cell | 0.0 |
| 8-cell | 0.0057 |
| 8-cell | 0.0052 |
| 8-cell | 0.0187 |
| 8-cell | 0.0106 |
| 8-cell | 0.0182 |
| 8-cell | 0.07 |
| 8-cell | 0.5093 |
| 8-cell | 0.154 |
| 8-cell | 0.0347 |
| 8-cell | 0.0384 |
| 8-cell | 0.0107 |
| 8-cell | 0.0081 |
| 8-cell | 0.0667 |
| 8-cell | 0.007 |
| 8-cell | 0.008 |
| 8-cell | 0.0072 |
| 8-cell | 0.0103 |
| 8-cell | 0.0119 |
| 8-cell | 0.0 |
| 8-cell | 0.0 |
| 8-cell | 0.0 |
| 8-cell | 0.0122 |
| 8-cell | 0.0079 |
| 8-cell | 0.0 |
| 8-cell | 0.0 |
| Morulae | 0.8407 |
| Morulae | 0.1618 |
| Morulae | 0.0062 |
| Morulae | 0.4665 |
| Morulae | 0.0457 |
| Morulae | 0.1049 |
| Morulae | 0.311 |
| Morulae | 0.0058 |
| Morulae | 0.0837 |
| Morulae | 0.4218 |
| Morulae | 0.0205 |
| Morulae | 0.0209 |
| Morulae | 0.2143 |
| Morulae | 0.0424 |
| Morulae | 0.2958 |
| Morulae | 0.3812 |
| Morulae | 0.0 |
| Morulae | 0.0263 |
| Morulae | 0.1405 |
| Late_blastocyst | 0.023 |
| Late_blastocyst | 0.0884 |
| Late_blastocyst | 0.0052 |
| Late_blastocyst | 0.0094 |
| Late_blastocyst | 0.0 |
| Late_blastocyst | 0.0071 |
| Late_blastocyst | 0.1799 |
| Late_blastocyst | 0.0243 |
| Late_blastocyst | 0.2333 |
| Late_blastocyst | 0.0256 |
| Late_blastocyst | 0.1334 |
| Late_blastocyst | 0.0234 |
| Late_blastocyst | 0.0174 |
| Late_blastocyst | 0.0362 |
| Late_blastocyst | 0.0298 |
| Late_blastocyst | 0.0068 |
| Late_blastocyst | 0.0134 |
| Late_blastocyst | 0.0 |
| Late_blastocyst | 0.0093 |
| Late_blastocyst | 0.0076 |
| Late_blastocyst | 0.0 |
| Late_blastocyst | 0.0148 |
| Late_blastocyst | 0.0 |
| Late_blastocyst | 0.0 |
| Late_blastocyst | 0.0057 |
| Late_blastocyst | 0.9673 |
| Late_blastocyst | 0.6565 |
| Late_blastocyst | 0.0 |
| Late_blastocyst | 0.0175 |
| Late_blastocyst | 0.0036 |
| hESC_P0 | 61.808 |
| hESC_P0 | 60.2252 |
| hESC_P0 | 72.3572 |
| hESC_P0 | 71.2271 |
| hESC_P0 | 32.1937 |
| hESC_P0 | 72.2674 |
| hESC_P0 | 53.0444 |
| hESC_P0 | 4.109 |
| hESC_P10 | 11.2305 |
| hESC_P10 | 24.0819 |
| hESC_P10 | 7.0716 |
| hESC_P10 | 3.2814 |
| hESC_P10 | 18.6633 |
| hESC_P10 | 15.1158 |
| hESC_P10 | 3.5234 |
| hESC_P10 | 23.719 |
| hESC_P10 | 0.0373 |
| hESC_P10 | 1.0839 |
| hESC_P10 | 0.0443 |
| hESC_P10 | 1.5146 |
| hESC_P10 | 1.1209 |
| hESC_P10 | 0.0717 |
| hESC_P10 | 10.4738 |
| hESC_P10 | 11.7486 |
| hESC_P10 | 0.0959 |
| hESC_P10 | 21.6196 |
| hESC_P10 | 20.014 |
| hESC_P10 | 0.0059 |
| hESC_P10 | 7.019 |
| hESC_P10 | 0.0191 |
| hESC_P10 | 22.5608 |
| hESC_P10 | 0.0219 |
| hESC_P10 | 0.0602 |
| hESC_P10 | 0.0191 |

## Slide 8
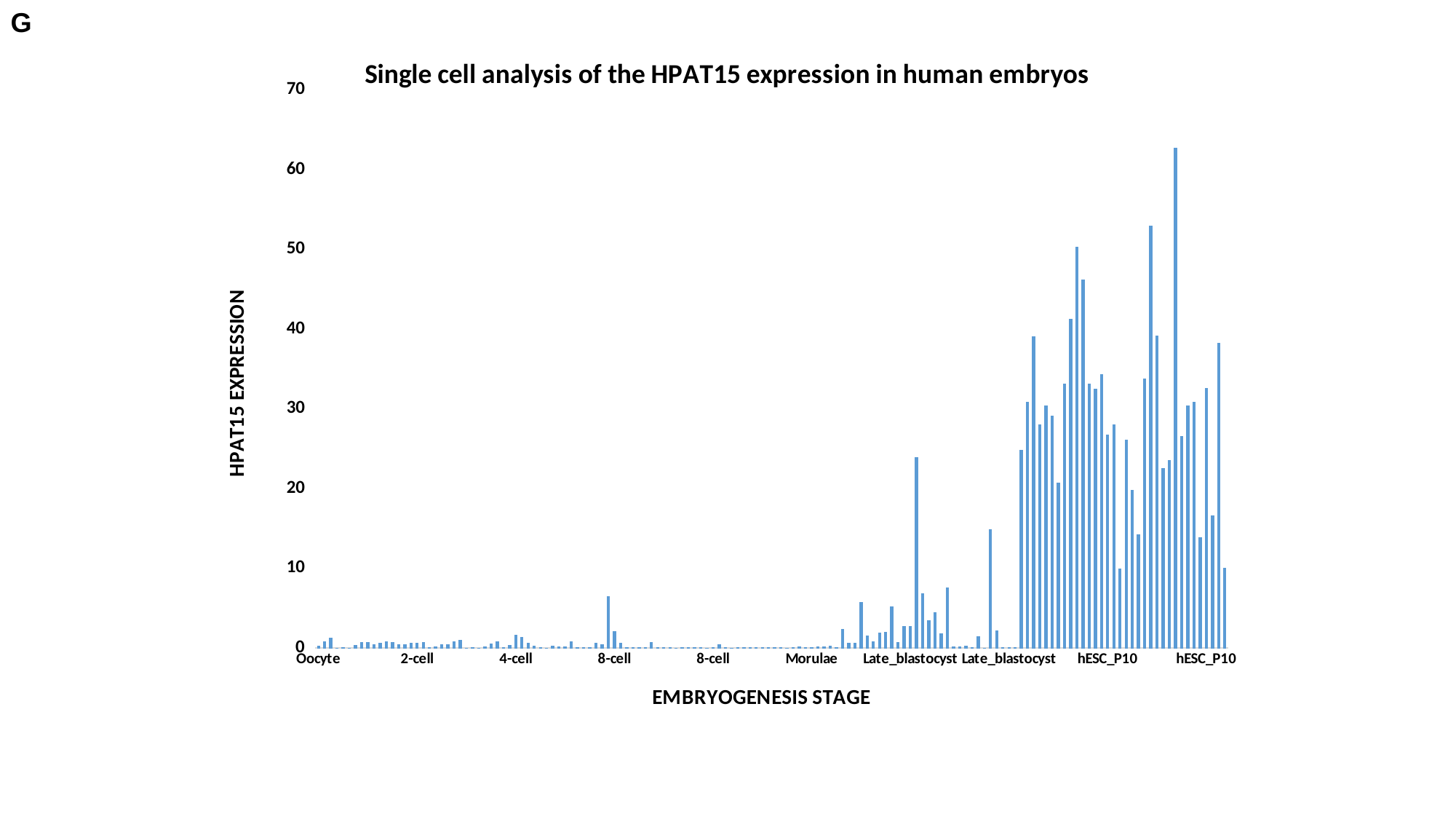

G
### Chart: Single cell analysis of the HPAT15 expression in human embryos
| Category | HPAT15 |
|---|---|
| Oocyte | 0.2775 |
| Oocyte | 0.7351 |
| Oocyte | 1.2101 |
| Oocyte | 0.0 |
| Oocyte | 0.0362 |
| Oocyte | 0.0 |
| Pronuclei | 0.3603 |
| Pronuclei | 0.7056 |
| Pronuclei | 0.7027 |
| Zygote | 0.3993 |
| Zygote | 0.6391 |
| Zygote | 0.791 |
| Zygote | 0.6518 |
| Zygote | 0.3951 |
| 2-cell | 0.4165 |
| 2-cell | 0.5652 |
| 2-cell | 0.6169 |
| 2-cell | 0.648 |
| 2-cell | 0.0639 |
| 2-cell | 0.0986 |
| 2-cell | 0.4219 |
| 2-cell | 0.4139 |
| 2-cell | 0.8223 |
| 4-cell | 0.958 |
| 4-cell | 0.0043 |
| 4-cell | 0.0123 |
| 4-cell | 0.0 |
| 4-cell | 0.1006 |
| 4-cell | 0.4937 |
| 4-cell | 0.8222 |
| 4-cell | 0.0557 |
| 4-cell | 0.3678 |
| 4-cell | 1.6411 |
| 4-cell | 1.285 |
| 4-cell | 0.6412 |
| 4-cell | 0.2182 |
| 4-cell | 0.0186 |
| 4-cell | 0.0 |
| 4-cell | 0.1924 |
| 8-cell | 0.1158 |
| 8-cell | 0.1413 |
| 8-cell | 0.7484 |
| 8-cell | 0.0564 |
| 8-cell | 0.0299 |
| 8-cell | 0.0206 |
| 8-cell | 0.5606 |
| 8-cell | 0.4506 |
| 8-cell | 6.4513 |
| 8-cell | 2.083 |
| 8-cell | 0.6261 |
| 8-cell | 0.0405 |
| 8-cell | 0.0138 |
| 8-cell | 0.0108 |
| 8-cell | 0.0239 |
| 8-cell | 0.6944 |
| 8-cell | 0.0082 |
| 8-cell | 0.0058 |
| 8-cell | 0.018 |
| 8-cell | 0.0 |
| 8-cell | 0.0108 |
| 8-cell | 0.0624 |
| 8-cell | 0.0092 |
| 8-cell | 0.009 |
| 8-cell | 0.0 |
| 8-cell | 0.0562 |
| Morulae | 0.4013 |
| Morulae | 0.061 |
| Morulae | 0.0 |
| Morulae | 0.0249 |
| Morulae | 0.0172 |
| Morulae | 0.0198 |
| Morulae | 0.024 |
| Morulae | 0.0066 |
| Morulae | 0.01 |
| Morulae | 0.021 |
| Morulae | 0.0077 |
| Morulae | 0.0047 |
| Morulae | 0.0058 |
| Morulae | 0.1067 |
| Morulae | 0.0946 |
| Morulae | 0.0263 |
| Morulae | 0.1484 |
| Morulae | 0.1665 |
| Morulae | 0.2473 |
| Late_blastocyst | 0.0391 |
| Late_blastocyst | 2.3454 |
| Late_blastocyst | 0.6193 |
| Late_blastocyst | 0.5618 |
| Late_blastocyst | 5.7291 |
| Late_blastocyst | 1.5122 |
| Late_blastocyst | 0.7839 |
| Late_blastocyst | 1.8518 |
| Late_blastocyst | 1.9482 |
| Late_blastocyst | 5.1384 |
| Late_blastocyst | 0.6572 |
| Late_blastocyst | 2.6595 |
| Late_blastocyst | 2.6606 |
| Late_blastocyst | 23.9263 |
| Late_blastocyst | 6.8465 |
| Late_blastocyst | 3.4316 |
| Late_blastocyst | 4.4816 |
| Late_blastocyst | 1.7919 |
| Late_blastocyst | 7.555 |
| Late_blastocyst | 0.155 |
| Late_blastocyst | 0.183 |
| Late_blastocyst | 0.2405 |
| Late_blastocyst | 0.0119 |
| Late_blastocyst | 1.4314 |
| Late_blastocyst | 0.0 |
| Late_blastocyst | 14.8329 |
| Late_blastocyst | 2.1332 |
| Late_blastocyst | 0.0414 |
| Late_blastocyst | 0.0346 |
| Late_blastocyst | 0.0737 |
| hESC_P0 | 24.831 |
| hESC_P0 | 30.8704 |
| hESC_P0 | 39.0875 |
| hESC_P0 | 27.9577 |
| hESC_P0 | 30.35 |
| hESC_P0 | 29.0806 |
| hESC_P0 | 20.7341 |
| hESC_P0 | 33.1358 |
| hESC_P10 | 41.2694 |
| hESC_P10 | 50.3021 |
| hESC_P10 | 46.1704 |
| hESC_P10 | 33.0958 |
| hESC_P10 | 32.5015 |
| hESC_P10 | 34.3355 |
| hESC_P10 | 26.6999 |
| hESC_P10 | 28.0 |
| hESC_P10 | 9.9586 |
| hESC_P10 | 26.1074 |
| hESC_P10 | 19.7781 |
| hESC_P10 | 14.23 |
| hESC_P10 | 33.7304 |
| hESC_P10 | 52.984 |
| hESC_P10 | 39.1272 |
| hESC_P10 | 22.5635 |
| hESC_P10 | 23.561 |
| hESC_P10 | 62.685 |
| hESC_P10 | 26.5221 |
| hESC_P10 | 30.3841 |
| hESC_P10 | 30.8194 |
| hESC_P10 | 13.8518 |
| hESC_P10 | 32.6179 |
| hESC_P10 | 16.6083 |
| hESC_P10 | 38.1996 |
| hESC_P10 | 9.9754 |

## Slide 9
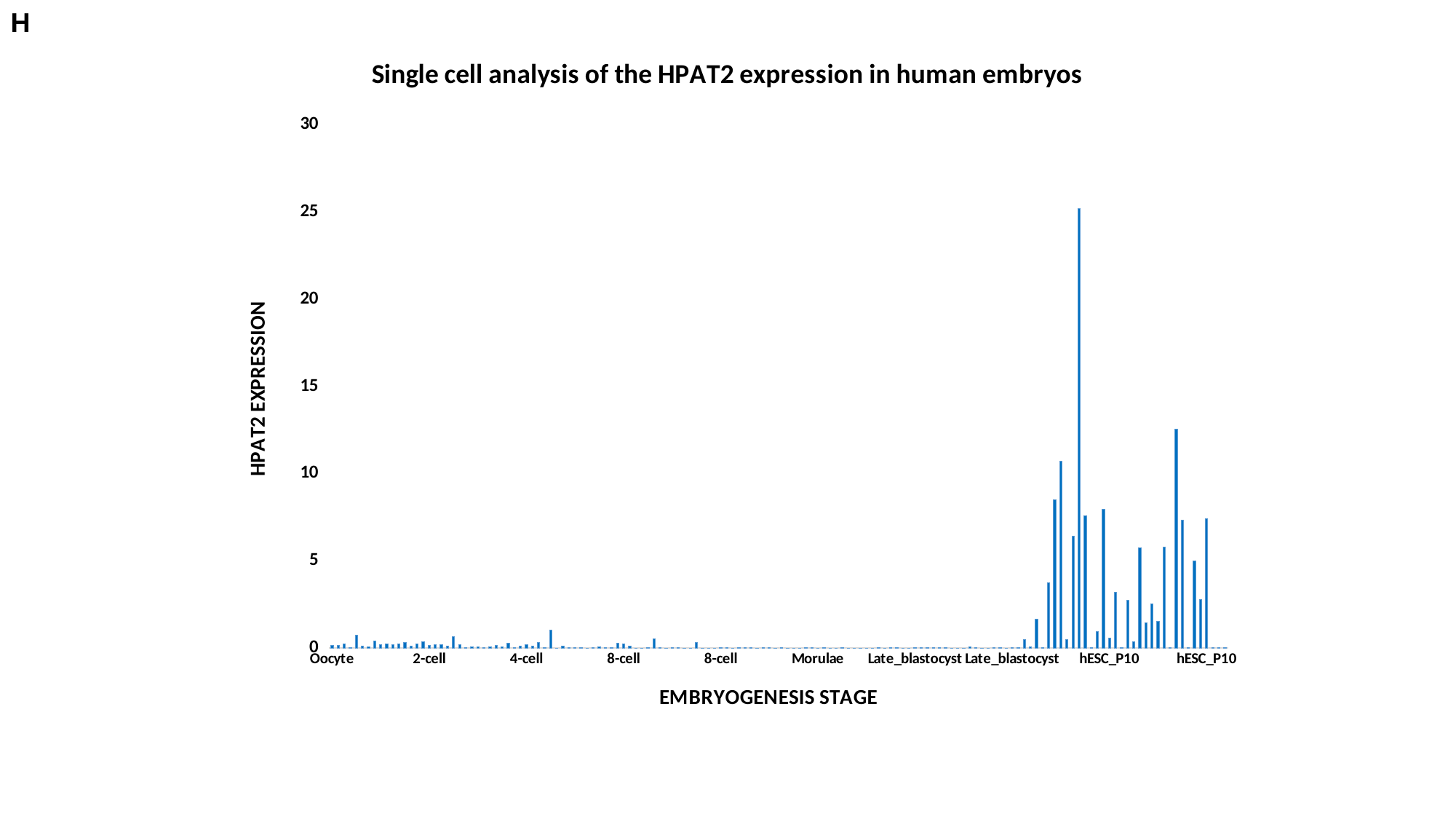

H
### Chart: Single cell analysis of the HPAT2 expression in human embryos
| Category | HPAT2 |
|---|---|
| Oocyte | 0.1347 |
| Oocyte | 0.1338 |
| Oocyte | 0.2122 |
| Oocyte | 0.0131 |
| Oocyte | 0.715 |
| Oocyte | 0.1136 |
| Pronuclei | 0.0648 |
| Pronuclei | 0.3835 |
| Pronuclei | 0.1801 |
| Zygote | 0.2429 |
| Zygote | 0.19 |
| Zygote | 0.2361 |
| Zygote | 0.3044 |
| Zygote | 0.0874 |
| 2-cell | 0.2228 |
| 2-cell | 0.3416 |
| 2-cell | 0.1503 |
| 2-cell | 0.2034 |
| 2-cell | 0.1787 |
| 2-cell | 0.1194 |
| 2-cell | 0.6394 |
| 2-cell | 0.2054 |
| 2-cell | 0.0126 |
| 4-cell | 0.0836 |
| 4-cell | 0.0685 |
| 4-cell | 0.0139 |
| 4-cell | 0.0467 |
| 4-cell | 0.1303 |
| 4-cell | 0.0792 |
| 4-cell | 0.2847 |
| 4-cell | 0.0288 |
| 4-cell | 0.1066 |
| 4-cell | 0.1714 |
| 4-cell | 0.0972 |
| 4-cell | 0.3132 |
| 4-cell | 0.0084 |
| 4-cell | 1.0218 |
| 4-cell | 0.0 |
| 4-cell | 0.0948 |
| 8-cell | 0.0051 |
| 8-cell | 0.0109 |
| 8-cell | 0.0099 |
| 8-cell | 0.0 |
| 8-cell | 0.0051 |
| 8-cell | 0.0814 |
| 8-cell | 0.0154 |
| 8-cell | 0.0337 |
| 8-cell | 0.2588 |
| 8-cell | 0.2407 |
| 8-cell | 0.1249 |
| 8-cell | 0.0 |
| 8-cell | 0.0 |
| 8-cell | 0.0046 |
| 8-cell | 0.521 |
| 8-cell | 0.0038 |
| 8-cell | 0.0 |
| 8-cell | 0.0098 |
| 8-cell | 0.0114 |
| 8-cell | 0.0 |
| 8-cell | 0.0 |
| 8-cell | 0.3119 |
| 8-cell | 0.0 |
| 8-cell | 0.0 |
| 8-cell | 0.0 |
| 8-cell | 0.0079 |
| Morulae | 0.0074 |
| Morulae | 0.0 |
| Morulae | 0.0118 |
| Morulae | 0.0042 |
| Morulae | 0.0145 |
| Morulae | 0.0 |
| Morulae | 0.0135 |
| Morulae | 0.0056 |
| Morulae | 0.0 |
| Morulae | 0.0044 |
| Morulae | 0.0 |
| Morulae | 0.0 |
| Morulae | 0.0 |
| Morulae | 0.0045 |
| Morulae | 0.0061 |
| Morulae | 0.0 |
| Morulae | 0.0157 |
| Morulae | 0.0 |
| Morulae | 0.0 |
| Late_blastocyst | 0.0037 |
| Late_blastocyst | 0.0 |
| Late_blastocyst | 0.0 |
| Late_blastocyst | 0.0 |
| Late_blastocyst | 0.0 |
| Late_blastocyst | 0.0 |
| Late_blastocyst | 0.0051 |
| Late_blastocyst | 0.0 |
| Late_blastocyst | 0.0061 |
| Late_blastocyst | 0.0122 |
| Late_blastocyst | 0.0 |
| Late_blastocyst | 0.0 |
| Late_blastocyst | 0.0056 |
| Late_blastocyst | 0.0058 |
| Late_blastocyst | 0.0071 |
| Late_blastocyst | 0.0065 |
| Late_blastocyst | 0.0064 |
| Late_blastocyst | 0.0059 |
| Late_blastocyst | 0.0 |
| Late_blastocyst | 0.0 |
| Late_blastocyst | 0.0 |
| Late_blastocyst | 0.0473 |
| Late_blastocyst | 0.0034 |
| Late_blastocyst | 0.0 |
| Late_blastocyst | 0.0 |
| Late_blastocyst | 0.0089 |
| Late_blastocyst | 0.005 |
| Late_blastocyst | 0.0 |
| Late_blastocyst | 0.0083 |
| Late_blastocyst | 0.0069 |
| hESC_P0 | 0.4847 |
| hESC_P0 | 0.0837 |
| hESC_P0 | 1.6302 |
| hESC_P0 | 0.0308 |
| hESC_P0 | 3.7498 |
| hESC_P0 | 8.4898 |
| hESC_P0 | 10.7242 |
| hESC_P0 | 0.4767 |
| hESC_P10 | 6.4104 |
| hESC_P10 | 25.2181 |
| hESC_P10 | 7.5743 |
| hESC_P10 | 0.0379 |
| hESC_P10 | 0.923 |
| hESC_P10 | 7.9655 |
| hESC_P10 | 0.5473 |
| hESC_P10 | 3.2145 |
| hESC_P10 | 0.0204 |
| hESC_P10 | 2.7348 |
| hESC_P10 | 0.364 |
| hESC_P10 | 5.7263 |
| hESC_P10 | 1.4458 |
| hESC_P10 | 2.5288 |
| hESC_P10 | 1.521 |
| hESC_P10 | 5.7653 |
| hESC_P10 | 0.027 |
| hESC_P10 | 12.5544 |
| hESC_P10 | 7.328 |
| hESC_P10 | 0.0282 |
| hESC_P10 | 4.9799 |
| hESC_P10 | 2.7903 |
| hESC_P10 | 7.4129 |
| hESC_P10 | 0.0157 |
| hESC_P10 | 0.0209 |
| hESC_P10 | 0.0244 |

## Slide 10
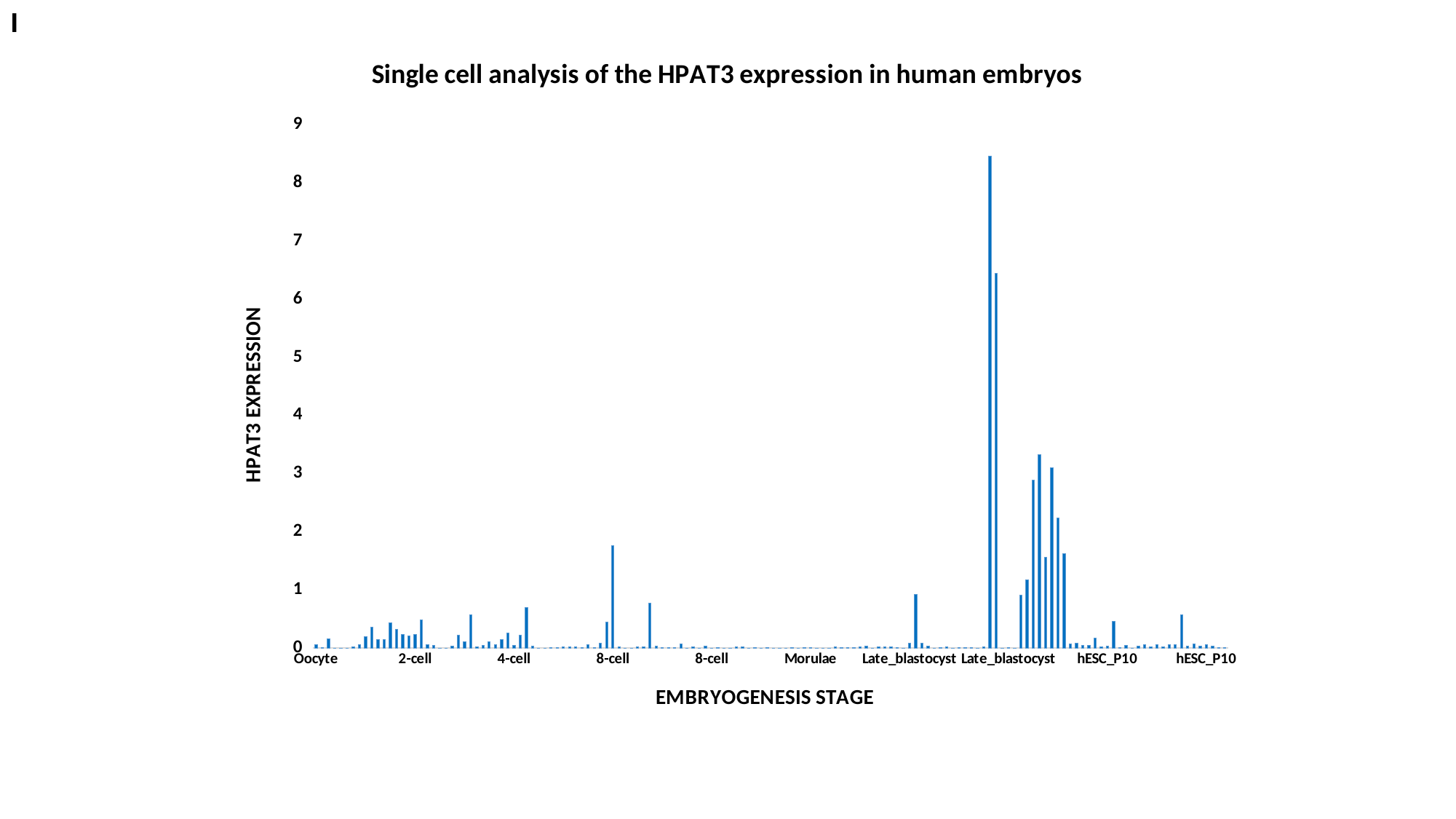

I
### Chart: Single cell analysis of the HPAT3 expression in human embryos
| Category | HPAT3 |
|---|---|
| Oocyte | 0.0541 |
| Oocyte | 0.0116 |
| Oocyte | 0.1603 |
| Oocyte | 0.0 |
| Oocyte | 0.0 |
| Oocyte | 0.0 |
| Pronuclei | 0.014 |
| Pronuclei | 0.0535 |
| Pronuclei | 0.1952 |
| Zygote | 0.3608 |
| Zygote | 0.1463 |
| Zygote | 0.1388 |
| Zygote | 0.4375 |
| Zygote | 0.3187 |
| 2-cell | 0.2379 |
| 2-cell | 0.2091 |
| 2-cell | 0.2354 |
| 2-cell | 0.4815 |
| 2-cell | 0.0512 |
| 2-cell | 0.0391 |
| 2-cell | 0.0 |
| 2-cell | 0.0 |
| 2-cell | 0.0363 |
| 4-cell | 0.2211 |
| 4-cell | 0.1055 |
| 4-cell | 0.5721 |
| 4-cell | 0.0169 |
| 4-cell | 0.046 |
| 4-cell | 0.1023 |
| 4-cell | 0.0588 |
| 4-cell | 0.1445 |
| 4-cell | 0.2543 |
| 4-cell | 0.0473 |
| 4-cell | 0.2198 |
| 4-cell | 0.6942 |
| 4-cell | 0.0318 |
| 4-cell | 0.0 |
| 4-cell | 0.0 |
| 4-cell | 0.0073 |
| 8-cell | 0.0112 |
| 8-cell | 0.0235 |
| 8-cell | 0.0161 |
| 8-cell | 0.0194 |
| 8-cell | 0.011 |
| 8-cell | 0.0567 |
| 8-cell | 0.0056 |
| 8-cell | 0.0836 |
| 8-cell | 0.4401 |
| 8-cell | 1.7546 |
| 8-cell | 0.0239 |
| 8-cell | 0.0 |
| 8-cell | 0.0 |
| 8-cell | 0.0197 |
| 8-cell | 0.0146 |
| 8-cell | 0.773 |
| 8-cell | 0.03 |
| 8-cell | 0.0053 |
| 8-cell | 0.0041 |
| 8-cell | 0.0091 |
| 8-cell | 0.0695 |
| 8-cell | 0.0 |
| 8-cell | 0.0169 |
| 8-cell | 0.0 |
| 8-cell | 0.0329 |
| 8-cell | 0.0 |
| Morulae | 0.012 |
| Morulae | 0.0 |
| Morulae | 0.0 |
| Morulae | 0.0137 |
| Morulae | 0.021 |
| Morulae | 0.0 |
| Morulae | 0.0073 |
| Morulae | 0.0 |
| Morulae | 0.0046 |
| Morulae | 0.0 |
| Morulae | 0.0 |
| Morulae | 0.0 |
| Morulae | 0.0053 |
| Morulae | 0.0 |
| Morulae | 0.0067 |
| Morulae | 0.0096 |
| Morulae | 0.0 |
| Morulae | 0.0 |
| Morulae | 0.0 |
| Late_blastocyst | 0.0159 |
| Late_blastocyst | 0.0083 |
| Late_blastocyst | 0.0107 |
| Late_blastocyst | 0.0049 |
| Late_blastocyst | 0.021 |
| Late_blastocyst | 0.0368 |
| Late_blastocyst | 0.0 |
| Late_blastocyst | 0.0189 |
| Late_blastocyst | 0.0166 |
| Late_blastocyst | 0.0199 |
| Late_blastocyst | 0.0099 |
| Late_blastocyst | 0.0 |
| Late_blastocyst | 0.0782 |
| Late_blastocyst | 0.9239 |
| Late_blastocyst | 0.0848 |
| Late_blastocyst | 0.0351 |
| Late_blastocyst | 0.0 |
| Late_blastocyst | 0.0128 |
| Late_blastocyst | 0.0192 |
| Late_blastocyst | 0.0 |
| Late_blastocyst | 0.0052 |
| Late_blastocyst | 0.0051 |
| Late_blastocyst | 0.0036 |
| Late_blastocyst | 0.0 |
| Late_blastocyst | 0.0178 |
| Late_blastocyst | 8.4583 |
| Late_blastocyst | 6.4488 |
| Late_blastocyst | 0.0 |
| Late_blastocyst | 0.0045 |
| Late_blastocyst | 0.0 |
| hESC_P0 | 0.9137 |
| hESC_P0 | 1.1702 |
| hESC_P0 | 2.8818 |
| hESC_P0 | 3.3313 |
| hESC_P0 | 1.5542 |
| hESC_P0 | 3.0977 |
| hESC_P0 | 2.2349 |
| hESC_P0 | 1.6201 |
| hESC_P10 | 0.0678 |
| hESC_P10 | 0.0846 |
| hESC_P10 | 0.046 |
| hESC_P10 | 0.0462 |
| hESC_P10 | 0.1642 |
| hESC_P10 | 0.0162 |
| hESC_P10 | 0.0322 |
| hESC_P10 | 0.4579 |
| hESC_P10 | 0.0055 |
| hESC_P10 | 0.042 |
| hESC_P10 | 0.0 |
| hESC_P10 | 0.0317 |
| hESC_P10 | 0.0629 |
| hESC_P10 | 0.0247 |
| hESC_P10 | 0.0556 |
| hESC_P10 | 0.0253 |
| hESC_P10 | 0.0584 |
| hESC_P10 | 0.0619 |
| hESC_P10 | 0.5711 |
| hESC_P10 | 0.0306 |
| hESC_P10 | 0.0638 |
| hESC_P10 | 0.0264 |
| hESC_P10 | 0.0566 |
| hESC_P10 | 0.034 |
| hESC_P10 | 0.0113 |
| hESC_P10 | 0.0066 |

## Slide 11
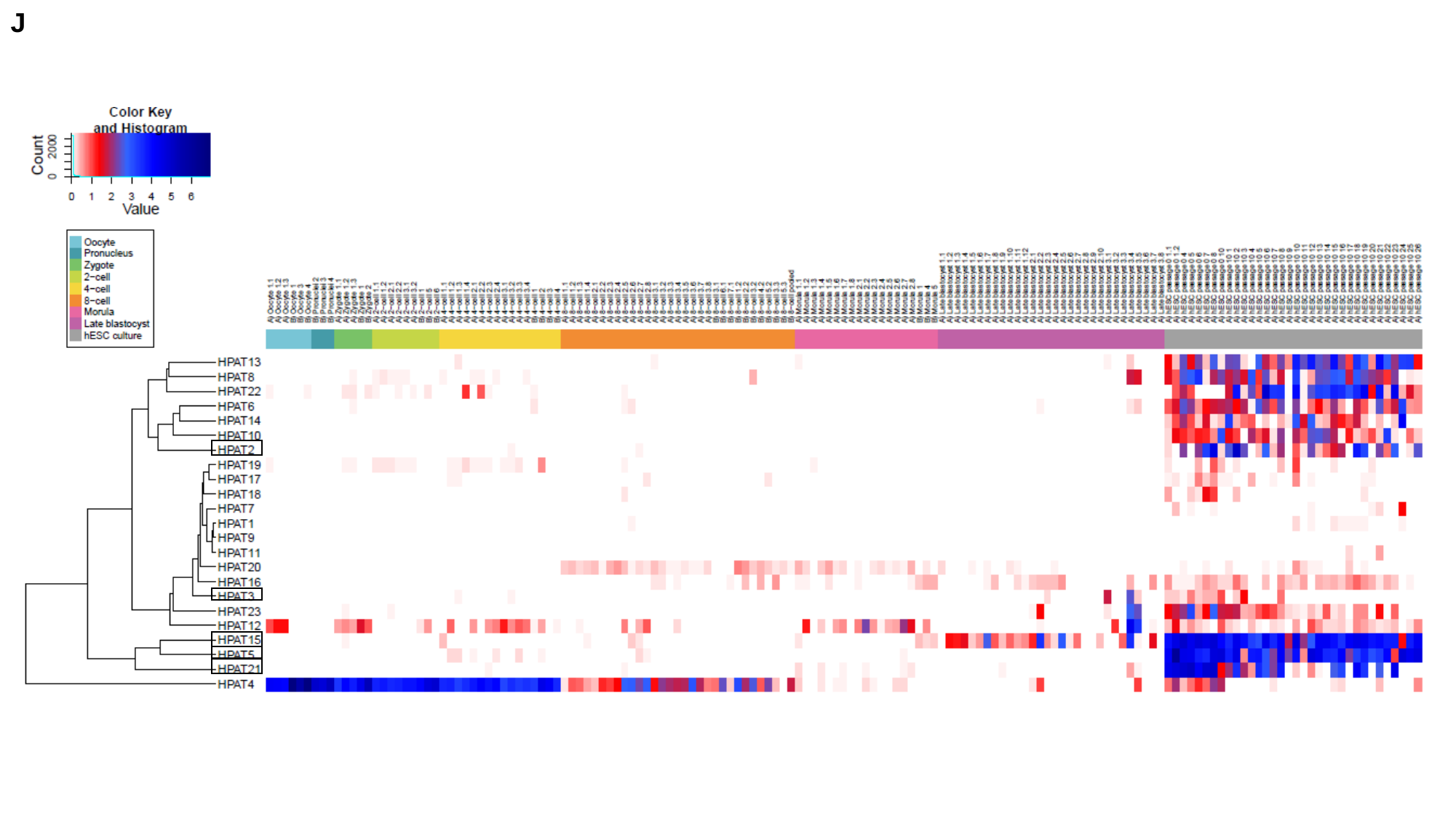

J
