# Supplementary material for: Single cell expression analysis of primate-specific retroviruses-derived HPAT lincRNAs in viable human blastocysts identifies embryonic cells co-expressing genetic markers of multiple lineages
Source: Heliyon. 2018 Jun 28;4(6):e00667. doi: 10.1016/j.heliyon.2018.e00667 (PMC6039856; doi:10.1016/j.heliyon.2018.e00667)
Supplement: Supplemental Figure S4 [file mmc7.pptx]

## Slide 1
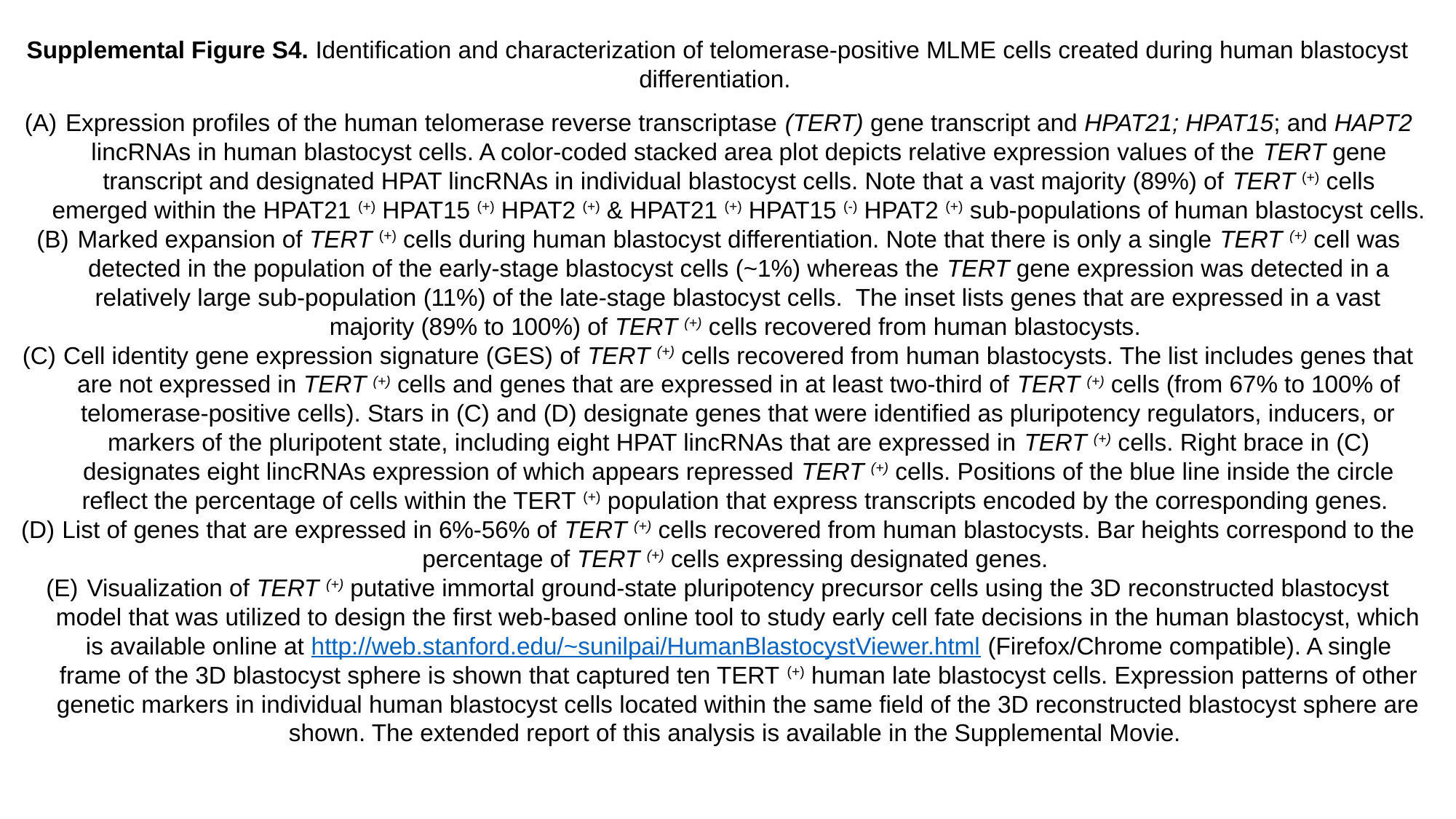

Supplemental Figure S4. Identification and characterization of telomerase-positive MLME cells created during human blastocyst differentiation.
Expression profiles of the human telomerase reverse transcriptase (TERT) gene transcript and HPAT21; HPAT15; and HAPT2 lincRNAs in human blastocyst cells. A color-coded stacked area plot depicts relative expression values of the TERT gene transcript and designated HPAT lincRNAs in individual blastocyst cells. Note that a vast majority (89%) of TERT (+) cells emerged within the HPAT21 (+) HPAT15 (+) HPAT2 (+) & HPAT21 (+) HPAT15 (-) HPAT2 (+) sub-populations of human blastocyst cells.
Marked expansion of TERT (+) cells during human blastocyst differentiation. Note that there is only a single TERT (+) cell was detected in the population of the early-stage blastocyst cells (~1%) whereas the TERT gene expression was detected in a relatively large sub-population (11%) of the late-stage blastocyst cells. The inset lists genes that are expressed in a vast majority (89% to 100%) of TERT (+) cells recovered from human blastocysts.
Cell identity gene expression signature (GES) of TERT (+) cells recovered from human blastocysts. The list includes genes that are not expressed in TERT (+) cells and genes that are expressed in at least two-third of TERT (+) cells (from 67% to 100% of telomerase-positive cells). Stars in (C) and (D) designate genes that were identified as pluripotency regulators, inducers, or markers of the pluripotent state, including eight HPAT lincRNAs that are expressed in TERT (+) cells. Right brace in (C) designates eight lincRNAs expression of which appears repressed TERT (+) cells. Positions of the blue line inside the circle reflect the percentage of cells within the TERT (+) population that express transcripts encoded by the corresponding genes.
List of genes that are expressed in 6%-56% of TERT (+) cells recovered from human blastocysts. Bar heights correspond to the percentage of TERT (+) cells expressing designated genes.
Visualization of TERT (+) putative immortal ground-state pluripotency precursor cells using the 3D reconstructed blastocyst model that was utilized to design the first web-based online tool to study early cell fate decisions in the human blastocyst, which is available online at http://web.stanford.edu/~sunilpai/HumanBlastocystViewer.html (Firefox/Chrome compatible). A single frame of the 3D blastocyst sphere is shown that captured ten TERT (+) human late blastocyst cells. Expression patterns of other genetic markers in individual human blastocyst cells located within the same field of the 3D reconstructed blastocyst sphere are shown. The extended report of this analysis is available in the Supplemental Movie.

## Slide 2
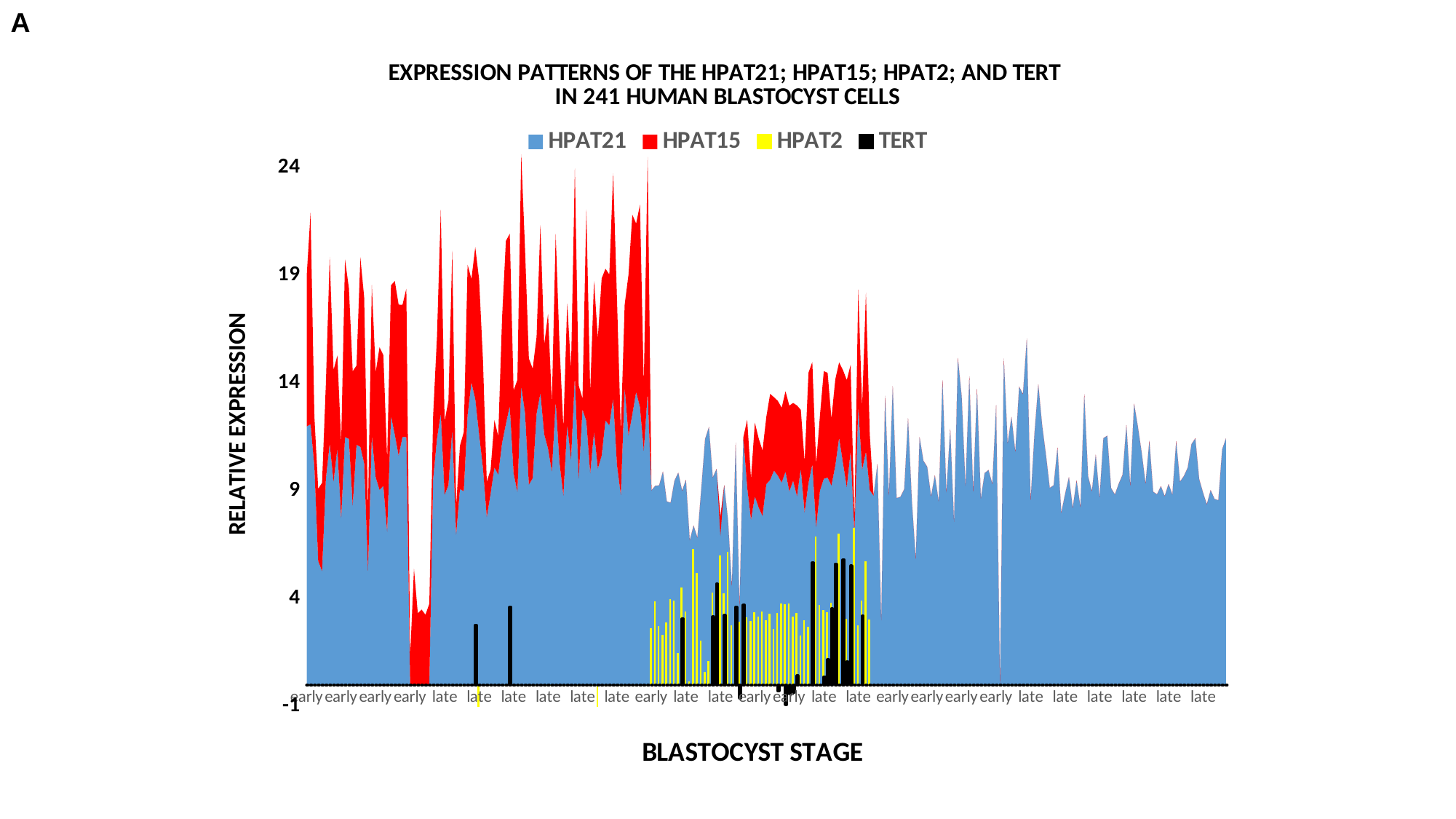

A
### Chart: EXPRESSION PATTERNS OF THE HPAT21; HPAT15; HPAT2; AND TERT
IN 241 HUMAN BLASTOCYST CELLS
| Category | HPAT21 | HPAT15 | HPAT2 | TERT |
|---|---|---|---|---|
| early | 11.99873328 | 6.999644486 | 0.0 | 0.0 |
| early | 12.09656665 | 9.813847643 | 0.0 | 0.0 |
| early | 10.13427742 | 2.344604301 | 0.0 | 0.0 |
| early | 5.754599522 | 3.35245381 | 0.0 | 0.0 |
| early | 5.287306204 | 4.093157857 | 0.0 | 0.0 |
| early | 9.574106248 | 4.425182724 | 0.0 | 0.0 |
| early | 11.1913057 | 8.666960099 | 0.0 | 0.0 |
| early | 9.44133588 | 5.169612625 | 0.0 | 0.0 |
| early | 10.97115032 | 4.320395435 | 0.0 | 0.0 |
| early | 7.742344586 | 3.504687137 | 0.0 | 0.0 |
| early | 11.50356021 | 8.235206997 | 0.0 | 0.0 |
| early | 11.41893282 | 6.999644486 | 0.0 | 0.0 |
| early | 8.329905938 | 6.225676974 | 0.0 | 0.0 |
| early | 11.14788624 | 3.677020836 | 0.0 | 0.0 |
| early | 11.04963373 | 8.780766755 | 0.0 | 0.0 |
| early | 10.27876473 | 7.713667593 | 0.0 | 0.0 |
| early | 5.315456706 | 2.807745118 | 0.0 | 0.0 |
| early | 11.5565427 | 6.999644486 | 0.0 | 0.0 |
| early | 9.638926496 | 4.87472492 | 0.0 | 0.0 |
| early | 9.058659432 | 6.606763509 | 0.0 | 0.0 |
| early | 9.241412852 | 6.068600087 | 0.0 | 0.0 |
| early | 7.109562728 | 3.360837596 | 0.0 | 0.0 |
| early | 12.48149197 | 6.060982836 | 0.0 | 0.0 |
| early | 11.61118426 | 7.127579823 | 0.0 | 0.0 |
| early | 10.6454496 | 6.999644486 | 0.0 | 0.0 |
| early | 11.50952022 | 6.131324157 | 0.0 | 0.0 |
| early | 11.49715307 | 6.888494201 | 0.0 | 0.0 |
| early | 0.0 | 1.320742124 | 0.0 | 0.0 |
| early | 0.0 | 5.391175979 | 0.0 | 0.0 |
| early | 0.0 | 3.333144198 | 0.0 | 0.0 |
| early | 0.0 | 3.504687137 | 0.0 | 0.0 |
| early | 0.0 | 3.264902478 | 0.0 | 0.0 |
| early | 0.0 | 3.771597479 | 0.0 | 0.0 |
| late | 9.558829867 | 2.940964042 | 0.0 | 0.0 |
| late | 11.41972851 | 4.728409592 | 0.0 | 0.0 |
| late | 12.59500261 | 9.450451764 | 0.0 | 0.0 |
| late | 8.826564321 | 3.422435712 | 0.0 | 0.0 |
| late | 9.27525597 | 3.91838008 | 0.0 | 0.0 |
| late | 11.86805801 | 8.272559701 | 0.0 | 0.0 |
| late | 6.971267732 | 1.344515584 | 0.0 | 0.0 |
| late | 9.092474245 | 2.007855905 | 0.0 | 0.0 |
| late | 8.99433664 | 2.739357077 | 0.0 | 0.0 |
| late | 12.52986891 | 6.965362361 | 0.0 | 0.0 |
| late | 14.03333605 | 4.800599859 | 0.0 | 0.0 |
| late | 13.27635206 | 7.038680975 | 0.0 | 2.774079458 |
| late | 11.63699366 | 7.190724234 | -1.372325551 | 0.0 |
| late | 10.07597512 | 5.01429947 | 0.0 | 0.0 |
| late | 7.795049203 | 1.637225178 | 0.0 | 0.0 |
| late | 8.869564511 | 1.119289369 | 0.0 | 0.0 |
| late | 10.09043319 | 2.193679317 | 0.0 | 0.0 |
| late | 9.765475655 | 1.785428457 | 0.0 | 0.0 |
| late | 11.22844835 | 5.797896165 | 0.0 | 0.0 |
| late | 12.10672969 | 8.493335314 | 0.0 | 0.0 |
| late | 12.94679892 | 7.984709387 | 0.0 | 3.615163184 |
| late | 9.825124493 | 3.834658106 | 0.0 | 0.0 |
| late | 8.961381358 | 5.200561061 | 0.0 | 0.0 |
| late | 13.85670767 | 10.75884697 | 0.0 | 0.0 |
| late | 12.60755625 | 7.57836944 | 0.0 | 0.0 |
| late | 9.293311204 | 5.854900215 | 0.0 | 0.0 |
| late | 9.600917569 | 5.075978964 | 0.0 | 0.0 |
| late | 12.57935156 | 3.575442967 | 0.0 | 0.0 |
| late | 13.5442982 | 7.793378649 | 0.0 | 0.0 |
| late | 11.62852467 | 4.172761118 | 0.0 | 0.0 |
| late | 10.9684515 | 6.249133772 | 0.0 | 0.0 |
| late | 9.906762252 | 3.205029641 | 0.0 | 0.0 |
| late | 13.14952558 | 7.785659508 | 0.0 | 0.0 |
| late | 10.30622556 | 5.437470883 | 0.0 | 0.0 |
| late | 8.794763869 | 3.156163892 | 0.0 | 0.0 |
| late | 12.06291432 | 5.633609591 | 0.0 | 0.0 |
| late | 10.46334108 | 4.201812648 | 0.0 | 0.0 |
| late | 14.29852828 | 9.665617414 | 0.0 | 0.0 |
| late | 9.573035261 | 4.325882086 | 0.0 | 0.0 |
| late | 12.78332699 | 0.51203456 | 0.0 | 0.0 |
| late | 12.24670217 | 9.803165699 | 0.0 | 0.0 |
| late | 9.818480447 | 3.796978022 | 0.0 | 0.0 |
| late | 11.74037174 | 6.999644486 | 0.0 | 0.0 |
| late | 10.0473369 | 6.044426253 | -1.161014903 | 0.0 |
| late | 10.64002268 | 8.225792389 | 0.0 | 0.0 |
| late | 12.27984078 | 7.031826214 | 0.0 | 0.0 |
| late | 12.04419204 | 6.999644486 | 0.0 | 0.0 |
| late | 13.29778217 | 10.43692477 | 0.0 | 0.0 |
| late | 10.20815657 | 7.7112409 | 0.0 | 0.0 |
| late | 8.818821763 | 2.929659754 | 0.0 | 0.0 |
| late | 13.81619759 | 3.823960209 | 0.0 | 0.0 |
| late | 11.57953383 | 7.449746331 | 0.0 | 0.0 |
| late | 12.54647313 | 9.268253195 | 0.0 | 0.0 |
| late | 13.59995793 | 7.806672512 | 0.0 | 0.0 |
| late | 12.89354855 | 9.380393292 | 0.0 | 0.0 |
| late | 10.87578243 | 3.049702529 | 0.0 | 0.0 |
| late | 13.53578914 | 10.95931928 | 0.0 | 0.0 |
| early | 9.033064504 | 0.0 | 2.634594948 | 0.0 |
| early | 9.245223238 | 0.0 | 3.885442059 | 0.0 |
| early | 9.260489458 | 0.0 | 2.731501793 | 0.0 |
| early | 9.907077152 | 0.0 | 2.324282882 | 0.0 |
| late | 8.521000634 | 0.0 | 2.906737711 | 0.0 |
| late | 8.458184215 | 0.0 | 3.982059036 | 0.0 |
| late | 9.474693441 | 0.0 | 3.891950834 | 0.0 |
| late | 9.853790123 | 0.0 | 1.472060372 | 0.0 |
| late | 9.015141933 | 0.0 | 4.511253463 | 3.078833031 |
| late | 9.518614742 | 0.0 | 3.40292233 | 0.0 |
| late | 6.71537474 | 0.0 | 0.167107029 | 0.0 |
| late | 7.398356747 | 0.0 | 6.285010357 | 0.0 |
| late | 6.841912395 | 0.0 | 5.178095773 | 0.0 |
| late | 9.10593594 | 0.0 | 2.043617438 | 0.0 |
| late | 11.41001146 | 0.0 | 0.593419375 | 0.0 |
| late | 11.96716417 | 0.0 | 1.116580112 | 0.0 |
| late | 9.630849802 | 0.0 | 4.284310208 | 3.161732538 |
| late | 10.02052555 | 0.0 | 3.725418455 | 4.694307042 |
| late | 7.79438844 | -0.869131379 | 6.001215206 | 0.0 |
| late | 9.274552856 | 0.0 | 4.227446748 | 3.230161941 |
| late | 7.635718345 | 0.0 | 6.148614006 | 0.0 |
| late | 4.615115977 | 0.0 | 2.75225298 | 0.0 |
| late | 11.2459727 | 0.0 | 3.64255185 | 3.620340097 |
| late | 3.653584127 | 0.0 | 2.925609168 | -0.587224516 |
| late | 11.49217643 | 0.0 | 1.35850542 | 3.714439424 |
| early | 9.177809734 | 3.130119372 | 3.129121409 | 0.002113229 |
| early | 7.678497736 | 1.910425554 | 2.957976007 | 0.0 |
| early | 8.751339296 | 3.419642754 | 3.355331988 | 0.0 |
| early | 8.239929371 | 3.199008789 | 3.168324941 | 0.0 |
| early | 7.842115889 | 3.044451098 | 3.384836229 | 0.0 |
| early | 9.320875354 | 3.121824864 | 2.975747455 | 0.0 |
| early | 9.517481307 | 3.989568765 | 3.292409123 | 0.0 |
| early | 9.949993194 | 3.40232397 | 2.576126822 | 0.0 |
| early | 9.717921588 | 3.465217243 | 3.319104948 | -0.266871417 |
| early | 9.396081376 | 3.471785474 | 3.775259341 | 0.0 |
| early | 9.901740887 | 3.723916017 | 3.743173927 | -0.898310463 |
| early | 8.986121946 | 3.97932822 | 3.772361413 | -0.408617338 |
| early | 9.477008574 | 3.610382099 | 3.171370682 | -0.332551286 |
| early | 8.763619981 | 4.207914506 | 3.332566483 | 0.431120958 |
| early | 10.03357658 | 2.732681683 | 2.287046579 | 0.0 |
| early | 8.007339926 | 2.369931054 | 2.984202125 | 0.0 |
| early | 9.371799334 | 5.118904173 | 2.696931444 | 0.0 |
| late | 10.24437818 | 4.739330053 | 4.102886678 | 5.663924274 |
| late | 7.324374079 | 2.934852552 | 6.890472033 | 0.0 |
| late | 9.015067783 | 3.499062144 | 3.702736267 | 0.0 |
| late | 9.570352278 | 4.989173683 | 3.453241424 | 0.388494351 |
| late | 9.622109205 | 4.862469888 | 3.362820726 | 1.180875766 |
| late | 9.25429144 | 3.117314401 | 3.805301144 | 3.551514238 |
| late | 10.18147076 | 4.004128035 | 4.320395435 | 5.621816945 |
| late | 11.4703229 | 3.497247353 | 6.993741077 | 0.0 |
| late | 10.30474567 | 4.30859141 | 4.502968081 | 5.816479375 |
| late | 9.187041144 | 4.962131502 | 3.05046107 | 1.094064958 |
| late | 10.83800592 | 4.013012984 | 4.320395435 | 5.522139142 |
| late | 7.054833379 | 0.648403971 | 7.28101729 | 0.0 |
| late | 12.92020327 | 5.428218911 | 2.744698662 | 0.0 |
| late | 9.993002739 | 2.919410882 | 3.890562912 | 3.203038203 |
| late | 10.82369712 | 7.378965361 | 5.729183087 | 0.0 |
| late | 9.038490771 | 2.586774112 | 3.022672268 | 0.0 |
| early | 8.787934638 | 0.0 | 0.0 | 0.0 |
| early | 10.25437555 | 0.0 | 0.0 | 0.0 |
| early | 3.005732898 | 0.0 | 0.0 | 0.0 |
| early | 13.42242129 | 0.0 | 0.0 | 0.0 |
| early | 8.799476777 | 0.0 | 0.0 | 0.0 |
| early | 13.87465034 | 0.0 | 0.0 | 0.0 |
| early | 8.674295391 | 0.0 | 0.0 | 0.0 |
| early | 8.730354358 | 0.0 | 0.0 | 0.0 |
| early | 9.083564239 | 0.0 | 0.0 | 0.0 |
| early | 12.38362599 | 0.0 | 0.0 | 0.0 |
| early | 8.524745249 | 0.0 | 0.0 | 0.0 |
| early | 5.866091498 | 0.0 | 0.0 | 0.0 |
| early | 11.50230284 | 0.0 | 0.0 | 0.0 |
| early | 10.40543704 | 0.0 | 0.0 | 0.0 |
| early | 10.12186759 | 0.0 | 0.0 | 0.0 |
| early | 8.764851705 | 0.0 | 0.0 | 0.0 |
| early | 9.725631945 | 0.0 | 0.0 | 0.0 |
| early | 8.522288066 | 0.0 | 0.0 | 0.0 |
| early | 14.14588307 | 0.0 | 0.0 | 0.0 |
| early | 8.934945526 | 0.0 | 0.0 | 0.0 |
| early | 11.89505561 | 0.0 | 0.0 | 0.0 |
| early | 7.569917726 | 0.0 | 0.0 | 0.0 |
| early | 15.1767082 | 0.0 | 0.0 | 0.0 |
| early | 13.38243367 | 0.0 | 0.0 | 0.0 |
| early | 9.235763203 | 0.0 | 0.0 | 0.0 |
| early | 14.31483726 | 0.0 | 0.0 | 0.0 |
| early | 8.983659342 | 0.0 | 0.0 | 0.0 |
| early | 13.73097211 | 0.0 | 0.0 | 0.0 |
| early | 8.659942056 | 0.0 | 0.0 | 0.0 |
| early | 9.832756611 | 0.0 | 0.0 | 0.0 |
| early | 9.977625103 | 0.0 | 0.0 | 0.0 |
| early | 9.335942869 | 0.0 | 0.0 | 0.0 |
| early | 12.98731777 | 0.0 | 0.0 | 0.0 |
| early | 0.0 | 0.0 | 0.0 | 0.0 |
| late | 15.15021438 | 0.0 | 0.0 | 0.0 |
| late | 11.2064789 | 0.0 | 0.0 | 0.0 |
| late | 12.4402782 | 0.0 | 0.0 | 0.0 |
| late | 10.82324621 | 0.0 | 0.0 | 0.0 |
| late | 13.83920966 | 0.0 | 0.0 | 0.0 |
| late | 13.49463211 | 0.0 | 0.0 | 0.0 |
| late | 16.0872559 | 0.0 | 0.0 | 0.0 |
| late | 8.581479683 | 0.0 | 0.0 | 0.0 |
| late | 11.66762662 | 0.0 | 0.0 | 0.0 |
| late | 13.95157394 | 0.0 | 0.0 | 0.0 |
| late | 12.05683786 | 0.0 | 0.0 | 0.0 |
| late | 10.6800382 | 0.0 | 0.0 | 0.0 |
| late | 9.144882707 | 0.0 | 0.0 | 0.0 |
| late | 9.267585143 | 0.0 | 0.0 | 0.0 |
| late | 11.01545707 | 0.0 | 0.0 | 0.0 |
| late | 7.995355818 | 0.0 | 0.0 | 0.0 |
| late | 8.865137094 | 0.0 | 0.0 | 0.0 |
| late | 9.649937139 | 0.0 | 0.0 | 0.0 |
| late | 8.224094333 | 0.0 | 0.0 | 0.0 |
| late | 9.493432842 | 0.0 | 0.0 | 0.0 |
| late | 8.25688029 | 0.0 | 0.0 | 0.0 |
| late | 13.49369866 | 0.0 | 0.0 | 0.0 |
| late | 9.688199255 | 0.0 | 0.0 | 0.0 |
| late | 8.994670352 | 0.0 | 0.0 | 0.0 |
| late | 10.69319989 | 0.0 | 0.0 | 0.0 |
| late | 8.713738436 | 0.0 | 0.0 | 0.0 |
| late | 11.45222818 | 0.0 | 0.0 | 0.0 |
| late | 11.5651113 | 0.0 | 0.0 | 0.0 |
| late | 9.138396132 | 0.0 | 0.0 | 0.0 |
| late | 8.838619591 | 0.0 | 0.0 | 0.0 |
| late | 9.34092825 | 0.0 | 0.0 | 0.0 |
| late | 9.754007655 | 0.0 | 0.0 | 0.0 |
| late | 12.05908817 | 0.0 | 0.0 | 0.0 |
| late | 9.265759641 | 0.0 | 0.0 | 0.0 |
| late | 13.0551395 | 0.0 | 0.0 | 0.0 |
| late | 11.97987575 | 0.0 | 0.0 | 0.0 |
| late | 10.76630201 | 0.0 | 0.0 | 0.0 |
| late | 9.352218139 | 0.0 | 0.0 | 0.0 |
| late | 11.33146695 | 0.0 | 0.0 | 0.0 |
| late | 8.966346629 | 0.0 | 0.0 | 0.0 |
| late | 8.848573294 | 0.0 | 0.0 | 0.0 |
| late | 9.224533756 | 0.0 | 0.0 | 0.0 |
| late | 8.765777742 | 0.0 | 0.0 | 0.0 |
| late | 9.32539489 | 0.0 | 0.0 | 0.0 |
| late | 8.839379363 | 0.0 | 0.0 | 0.0 |
| late | 11.31452889 | 0.0 | 0.0 | 0.0 |
| late | 9.436737908 | 0.0 | 0.0 | 0.0 |
| late | 9.690996429 | 0.0 | 0.0 | 0.0 |
| late | 10.07995235 | 0.0 | 0.0 | 0.0 |
| late | 11.17803099 | 0.0 | 0.0 | 0.0 |
| late | 11.44363475 | 0.0 | 0.0 | 0.0 |
| late | 9.554988975 | 0.0 | 0.0 | 0.0 |
| late | 8.930659061 | 0.0 | 0.0 | 0.0 |
| late | 8.38549196 | 0.0 | 0.0 | 0.0 |
| late | 9.043235561 | 0.0 | 0.0 | 0.0 |
| late | 8.63216944 | 0.0 | 0.0 | 0.0 |
| late | 8.573426229 | 0.0 | 0.0 | 0.0 |
| late | 10.92942806 | 0.0 | 0.0 | 0.0 |
| late | 11.44340375 | 0.0 | 0.0 | 0.0 |

## Slide 3
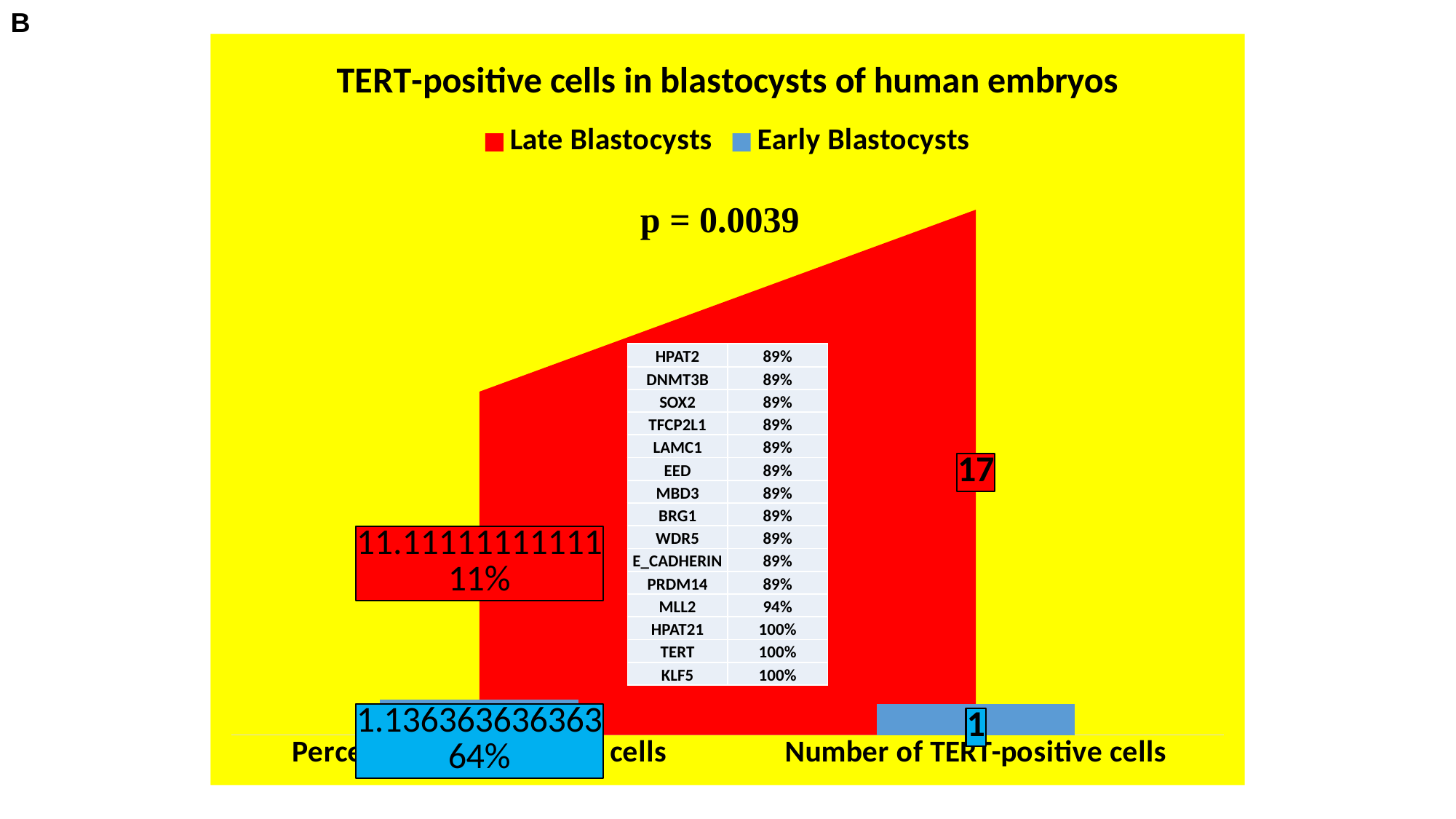

B
### Chart: TERT-positive cells in blastocysts of human embryos
| Category | Late Blastocysts | Early Blastocysts |
|---|---|---|
| Percent of TERT-positive cells | 11.11111111111111 | 1.1363636363636365 |
| Number of TERT-positive cells | 17.0 | 1.0 || HPAT2 | 89% |
| --- | --- |
| DNMT3B | 89% |
| SOX2 | 89% |
| TFCP2L1 | 89% |
| LAMC1 | 89% |
| EED | 89% |
| MBD3 | 89% |
| BRG1 | 89% |
| WDR5 | 89% |
| E\_CADHERIN | 89% |
| PRDM14 | 89% |
| MLL2 | 94% |
| HPAT21 | 100% |
| TERT | 100% |
| KLF5 | 100% |

## Slide 4
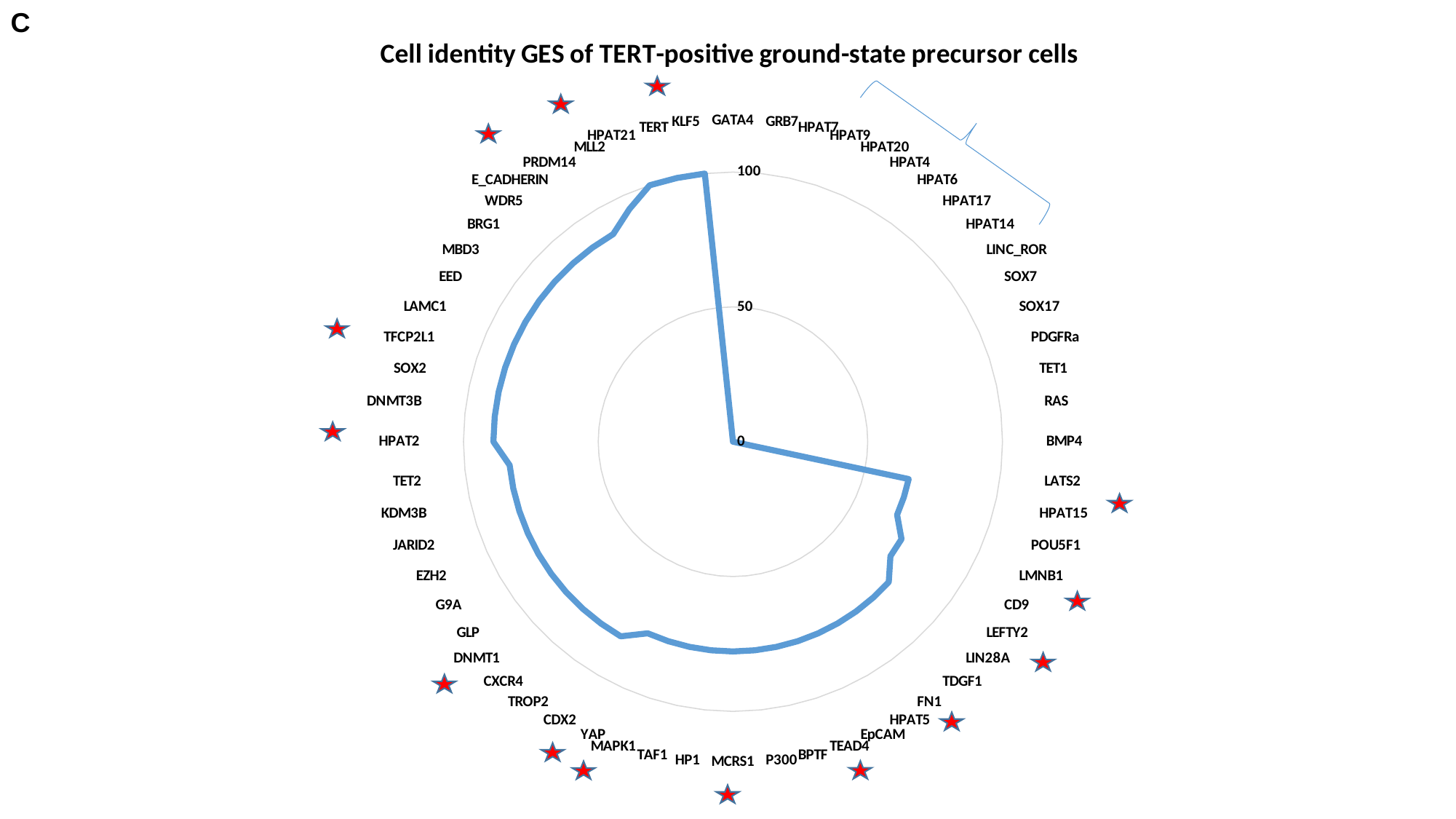

C
### Chart: Cell identity GES of TERT-positive ground-state precursor cells
| Category | Cell identity GES of TERT-positive multilineage precursor cells |
|---|---|
| GATA4 | 0.0 |
| GRB7 | 0.0 |
| HPAT7 | 0.0 |
| HPAT9 | 0.0 |
| HPAT20 | 0.0 |
| HPAT4 | 0.0 |
| HPAT6 | 0.0 |
| HPAT17 | 0.0 |
| HPAT14 | 0.0 |
| LINC_ROR | 0.0 |
| SOX7 | 0.0 |
| SOX17 | 0.0 |
| PDGFRa | 0.0 |
| TET1 | 0.0 |
| RAS | 0.0 |
| BMP4 | 0.0 |
| LATS2 | 0.0 |
| HPAT15 | 66.66666666666666 |
| POU5F1 | 66.66666666666666 |
| LMNB1 | 66.66666666666666 |
| CD9 | 72.22222222222221 |
| LEFTY2 | 72.22222222222221 |
| LIN28A | 77.77777777777779 |
| TDGF1 | 77.77777777777779 |
| FN1 | 77.77777777777779 |
| HPAT5 | 77.77777777777779 |
| EpCAM | 77.77777777777779 |
| TEAD4 | 77.77777777777779 |
| BPTF | 77.77777777777779 |
| P300 | 77.77777777777779 |
| MCRS1 | 77.77777777777779 |
| HP1 | 77.77777777777779 |
| TAF1 | 77.77777777777779 |
| MAPK1 | 77.77777777777779 |
| YAP | 77.77777777777779 |
| CDX2 | 83.33333333333334 |
| TROP2 | 83.33333333333334 |
| CXCR4 | 83.33333333333334 |
| DNMT1 | 83.33333333333334 |
| GLP | 83.33333333333334 |
| G9A | 83.33333333333334 |
| EZH2 | 83.33333333333334 |
| JARID2 | 83.33333333333334 |
| KDM3B | 83.33333333333334 |
| TET2 | 83.33333333333334 |
| HPAT2 | 88.88888888888889 |
| DNMT3B | 88.88888888888889 |
| SOX2 | 88.88888888888889 |
| TFCP2L1 | 88.88888888888889 |
| LAMC1 | 88.88888888888889 |
| EED | 88.88888888888889 |
| MBD3 | 88.88888888888889 |
| BRG1 | 88.88888888888889 |
| WDR5 | 88.88888888888889 |
| E_CADHERIN | 88.88888888888889 |
| PRDM14 | 88.88888888888889 |
| MLL2 | 94.44444444444444 |
| HPAT21 | 100.0 |
| TERT | 100.0 |
| KLF5 | 100.0 |

## Slide 5
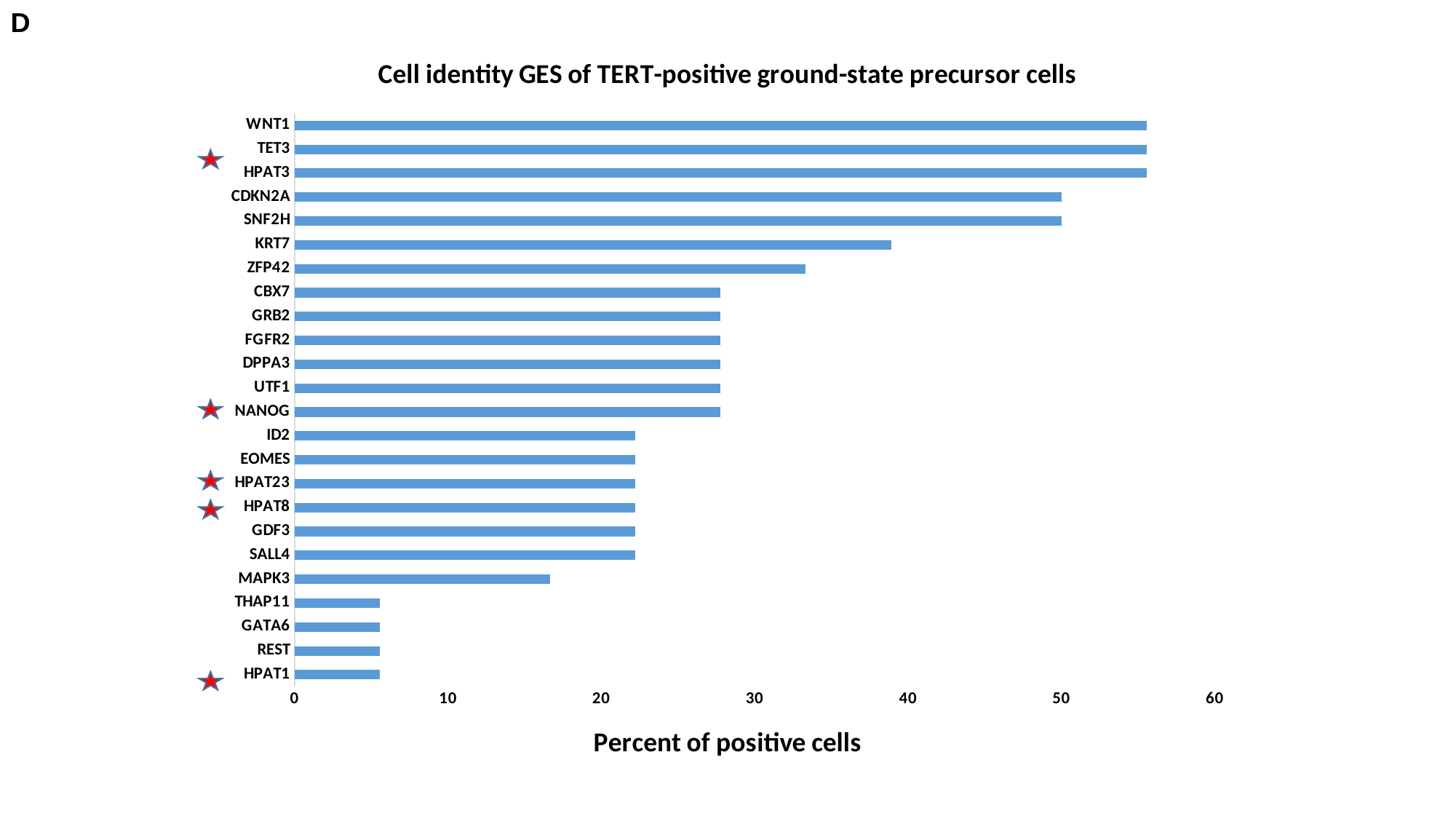

D
### Chart: Cell identity GES of TERT-positive ground-state precursor cells
| Category | Cell identity GES of TERT-positive multilineage precursor cells |
|---|---|
| HPAT1 | 5.555555555555555 |
| REST | 5.555555555555555 |
| GATA6 | 5.555555555555555 |
| THAP11 | 5.555555555555555 |
| MAPK3 | 16.666666666666664 |
| SALL4 | 22.22222222222222 |
| GDF3 | 22.22222222222222 |
| HPAT8 | 22.22222222222222 |
| HPAT23 | 22.22222222222222 |
| EOMES | 22.22222222222222 |
| ID2 | 22.22222222222222 |
| NANOG | 27.77777777777778 |
| UTF1 | 27.77777777777778 |
| DPPA3 | 27.77777777777778 |
| FGFR2 | 27.77777777777778 |
| GRB2 | 27.77777777777778 |
| CBX7 | 27.77777777777778 |
| ZFP42 | 33.33333333333333 |
| KRT7 | 38.88888888888889 |
| SNF2H | 50.0 |
| CDKN2A | 50.0 |
| HPAT3 | 55.55555555555556 |
| TET3 | 55.55555555555556 |
| WNT1 | 55.55555555555556 |

## Slide 6
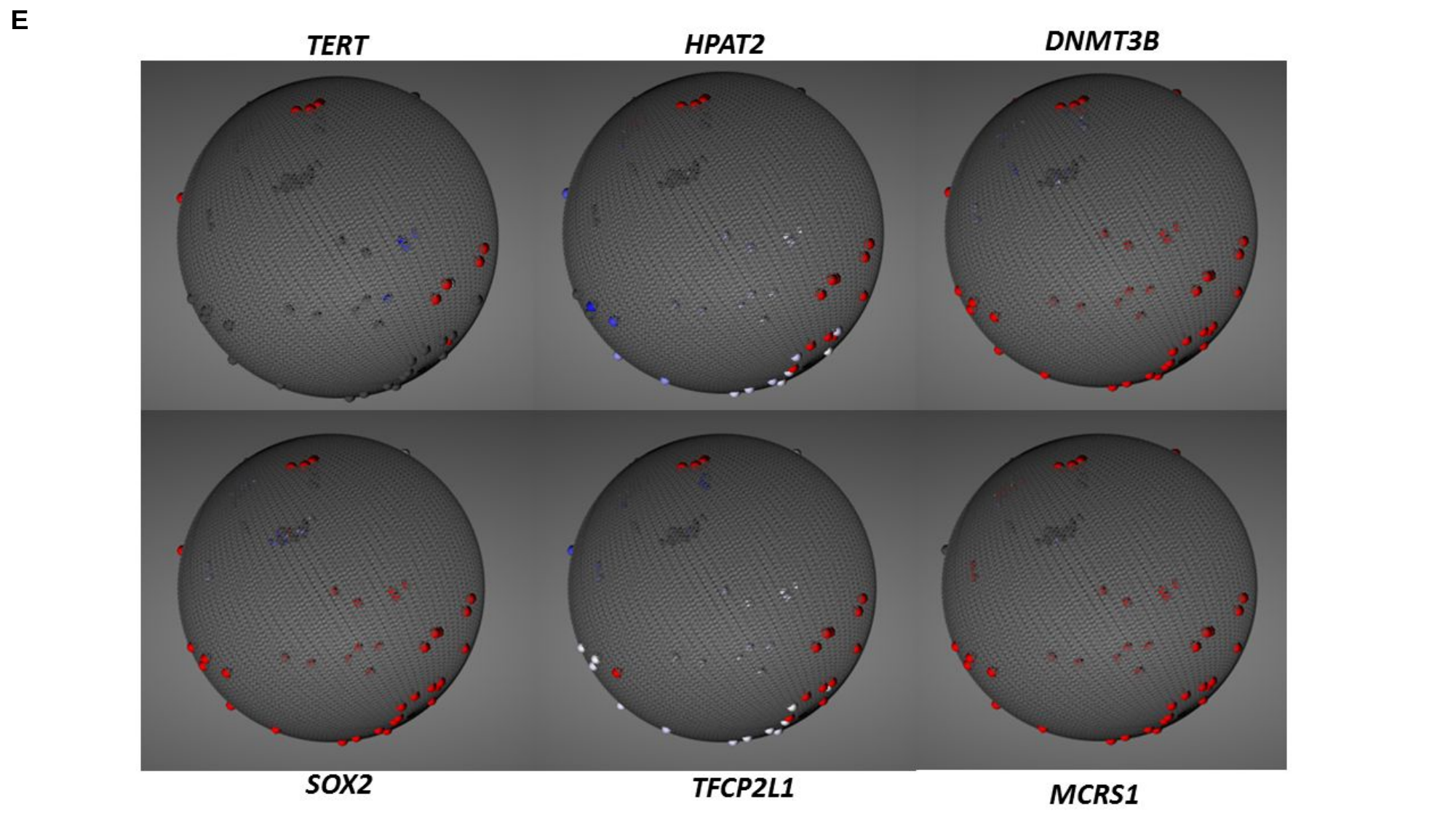

E
