# Supplementary material for: Single cell expression analysis of primate-specific retroviruses-derived HPAT lincRNAs in viable human blastocysts identifies embryonic cells co-expressing genetic markers of multiple lineages
Source: Heliyon. 2018 Jun 28;4(6):e00667. doi: 10.1016/j.heliyon.2018.e00667 (PMC6039856; doi:10.1016/j.heliyon.2018.e00667)

## Slide 1
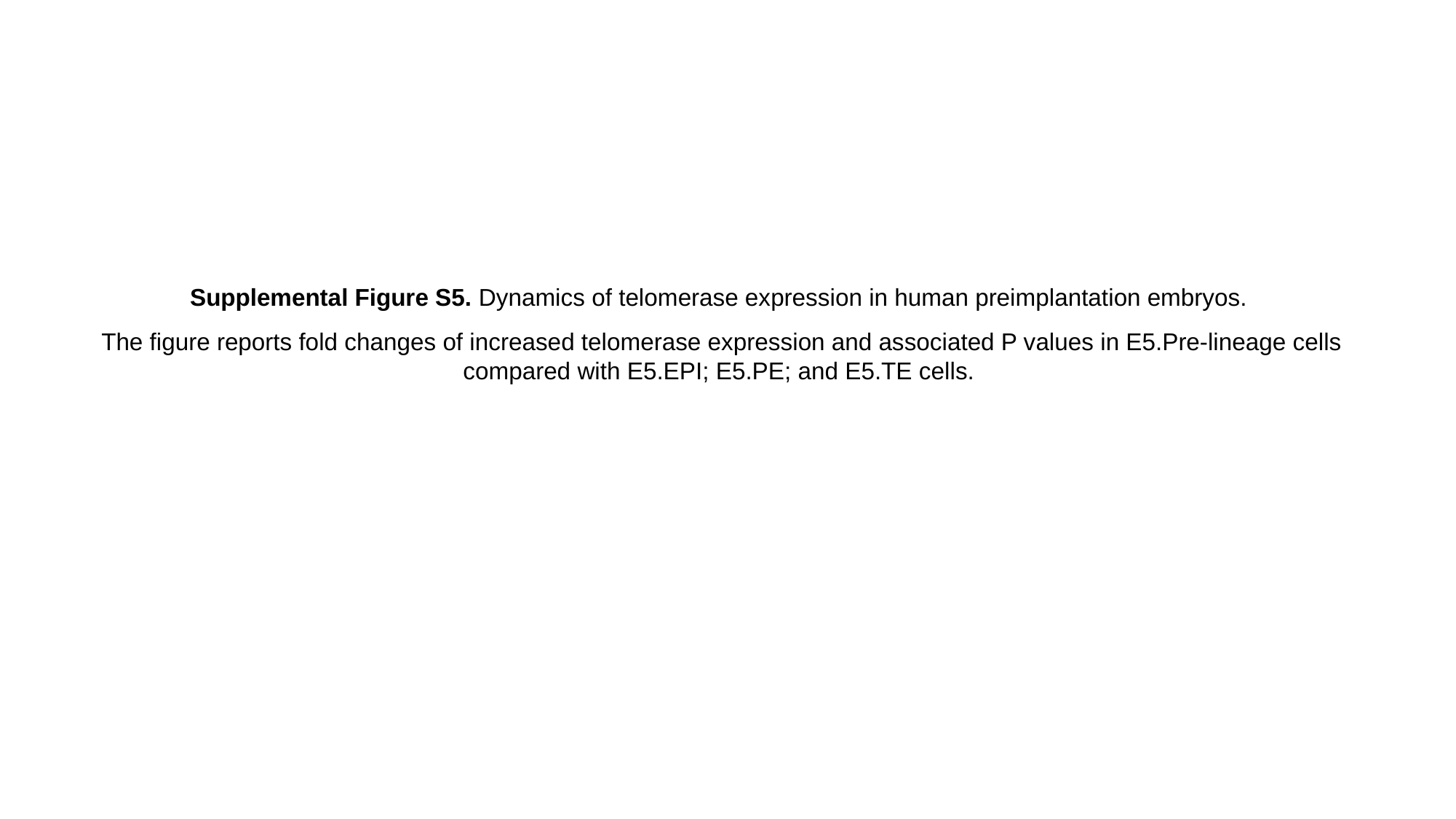

Supplemental Figure S5. Dynamics of telomerase expression in human preimplantation embryos.
The figure reports fold changes of increased telomerase expression and associated P values in E5.Pre-lineage cells compared with E5.EPI; E5.PE; and E5.TE cells.

## Slide 2
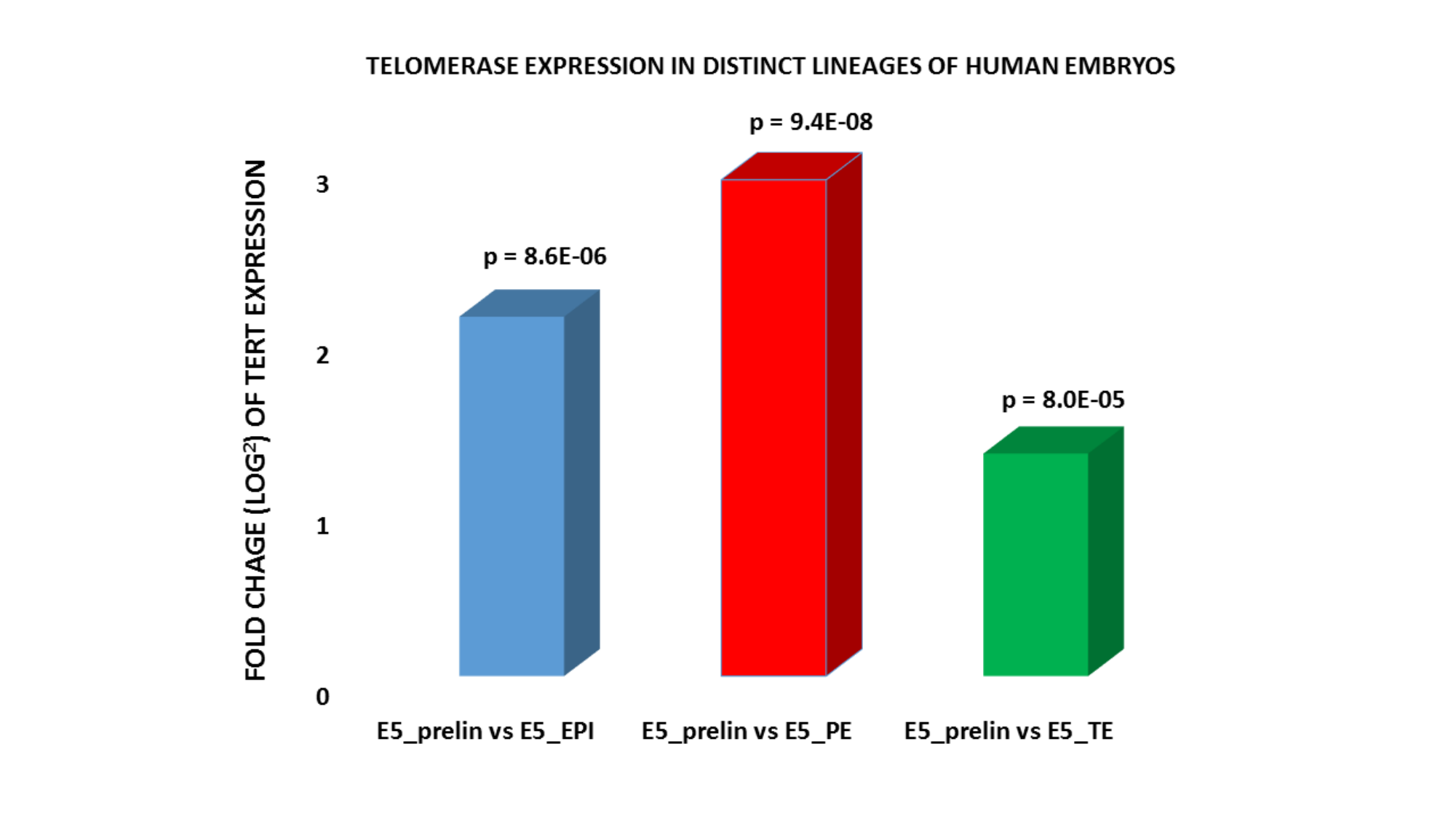

Supplement: Supplemental Figure S5 [file mmc8.pptx]
