# Supplementary material for: Single cell expression analysis of primate-specific retroviruses-derived HPAT lincRNAs in viable human blastocysts identifies embryonic cells co-expressing genetic markers of multiple lineages
Source: Heliyon. 2018 Jun 28;4(6):e00667. doi: 10.1016/j.heliyon.2018.e00667 (PMC6039856; doi:10.1016/j.heliyon.2018.e00667)
Supplement: Supplemental Figure S6 [file mmc9.pptx]

## Slide 1
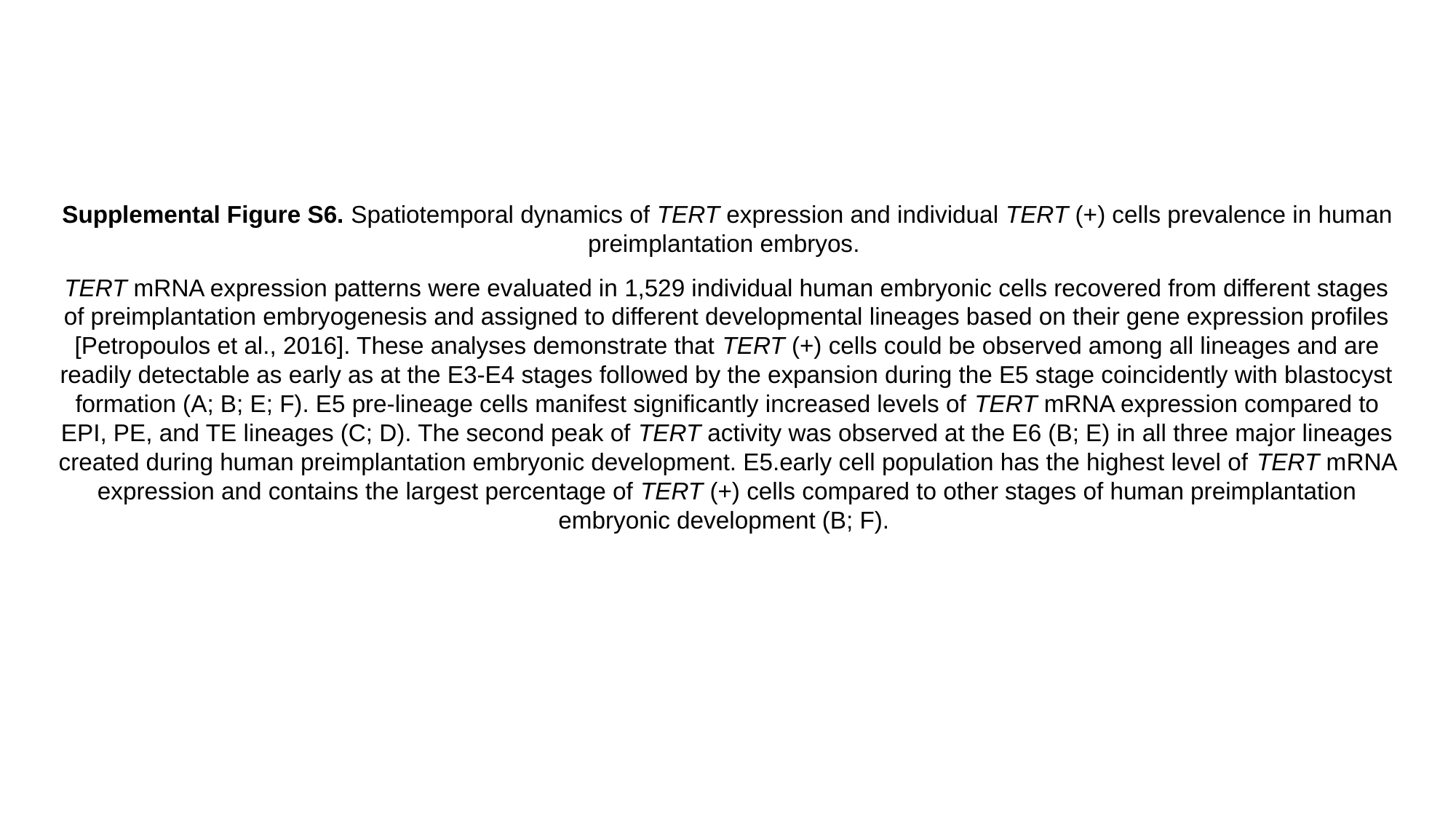

Supplemental Figure S6. Spatiotemporal dynamics of TERT expression and individual TERT (+) cells prevalence in human preimplantation embryos.
TERT mRNA expression patterns were evaluated in 1,529 individual human embryonic cells recovered from different stages of preimplantation embryogenesis and assigned to different developmental lineages based on their gene expression profiles [Petropoulos et al., 2016]. These analyses demonstrate that TERT (+) cells could be observed among all lineages and are readily detectable as early as at the E3-E4 stages followed by the expansion during the E5 stage coincidently with blastocyst formation (A; B; E; F). E5 pre-lineage cells manifest significantly increased levels of TERT mRNA expression compared to EPI, PE, and TE lineages (C; D). The second peak of TERT activity was observed at the E6 (B; E) in all three major lineages created during human preimplantation embryonic development. E5.early cell population has the highest level of TERT mRNA expression and contains the largest percentage of TERT (+) cells compared to other stages of human preimplantation embryonic development (B; F).

## Slide 2
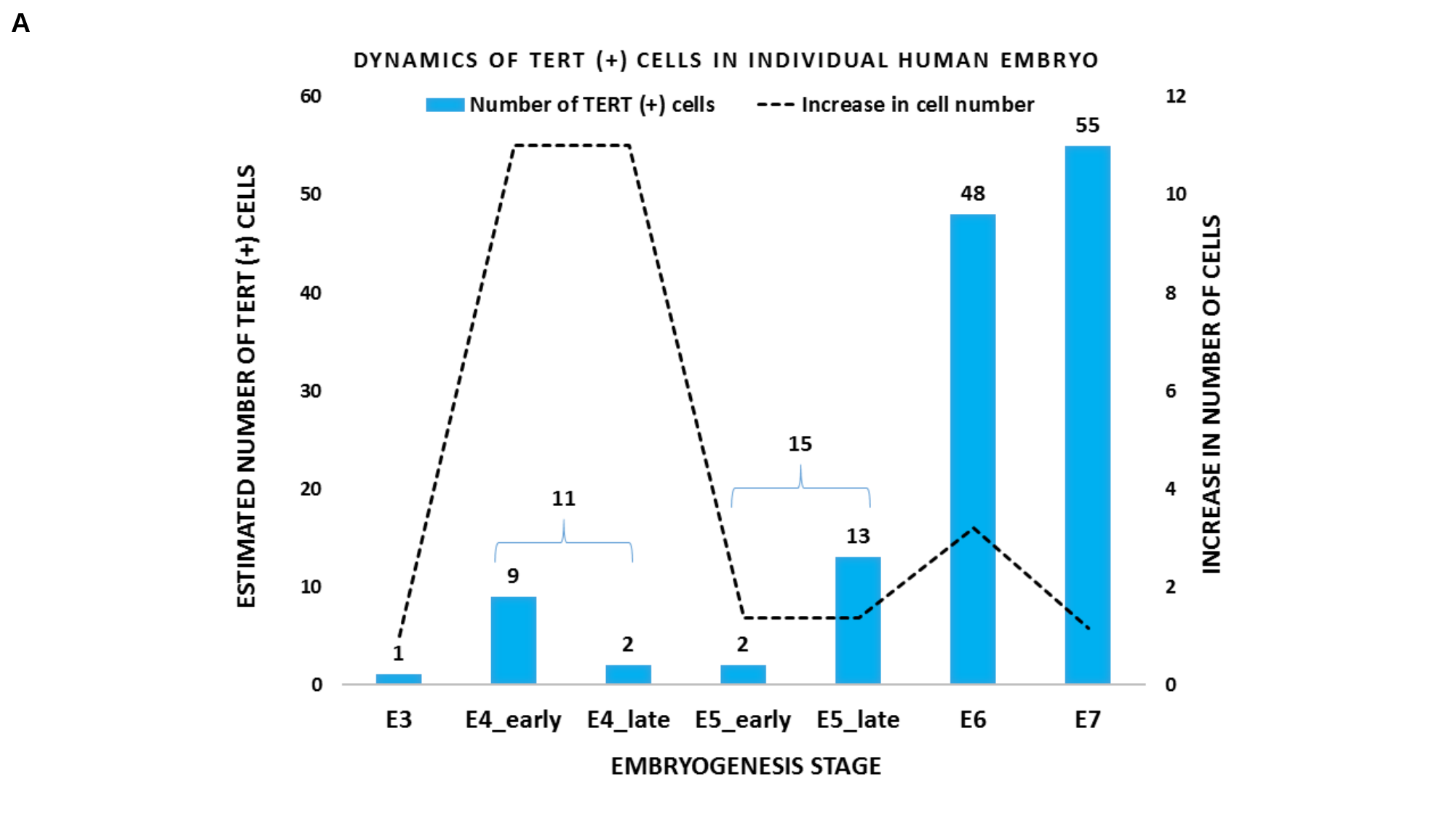

A

## Slide 3
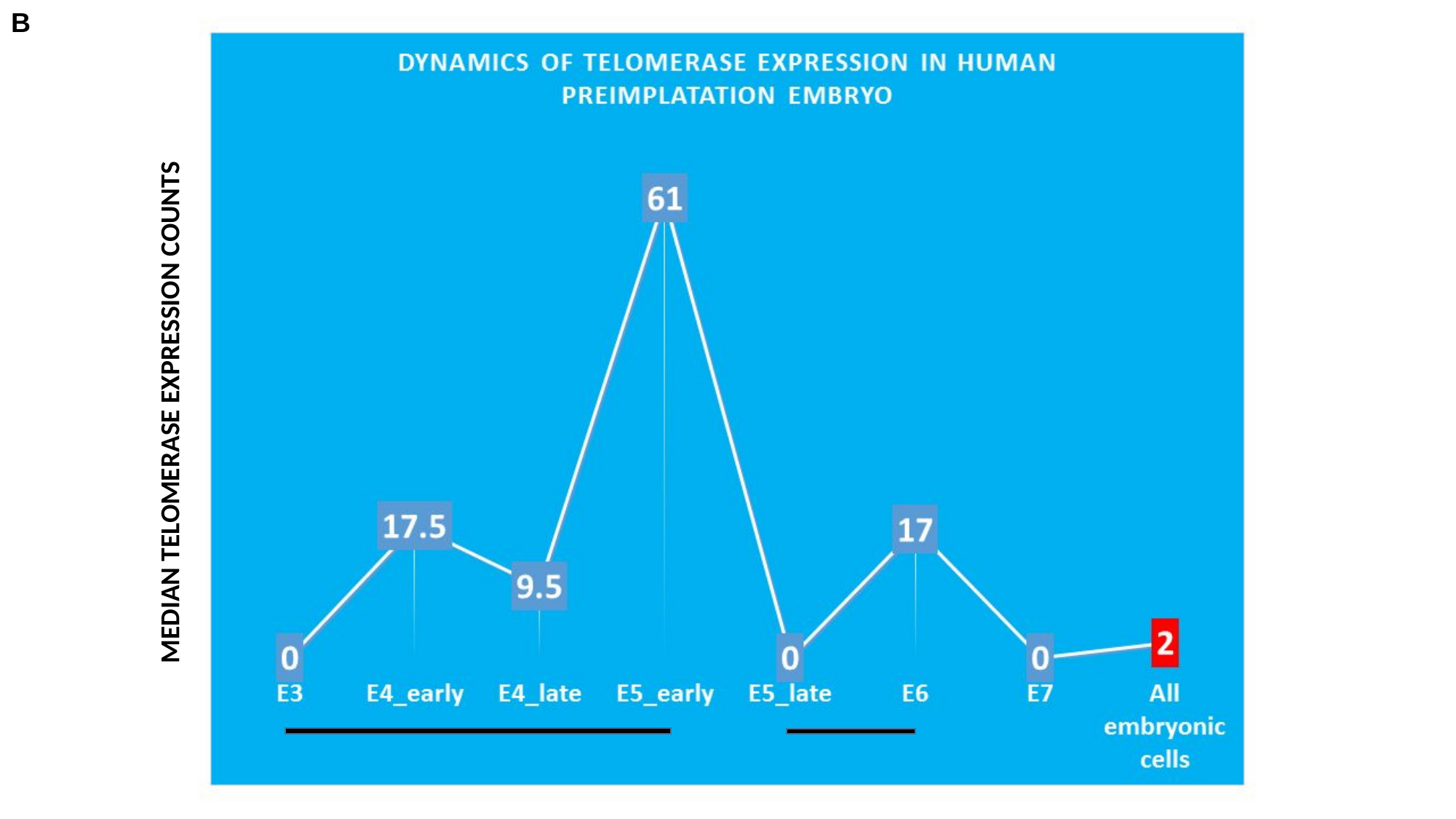

B
MEDIAN TELOMERASE EXPRESSION COUNTS

## Slide 4
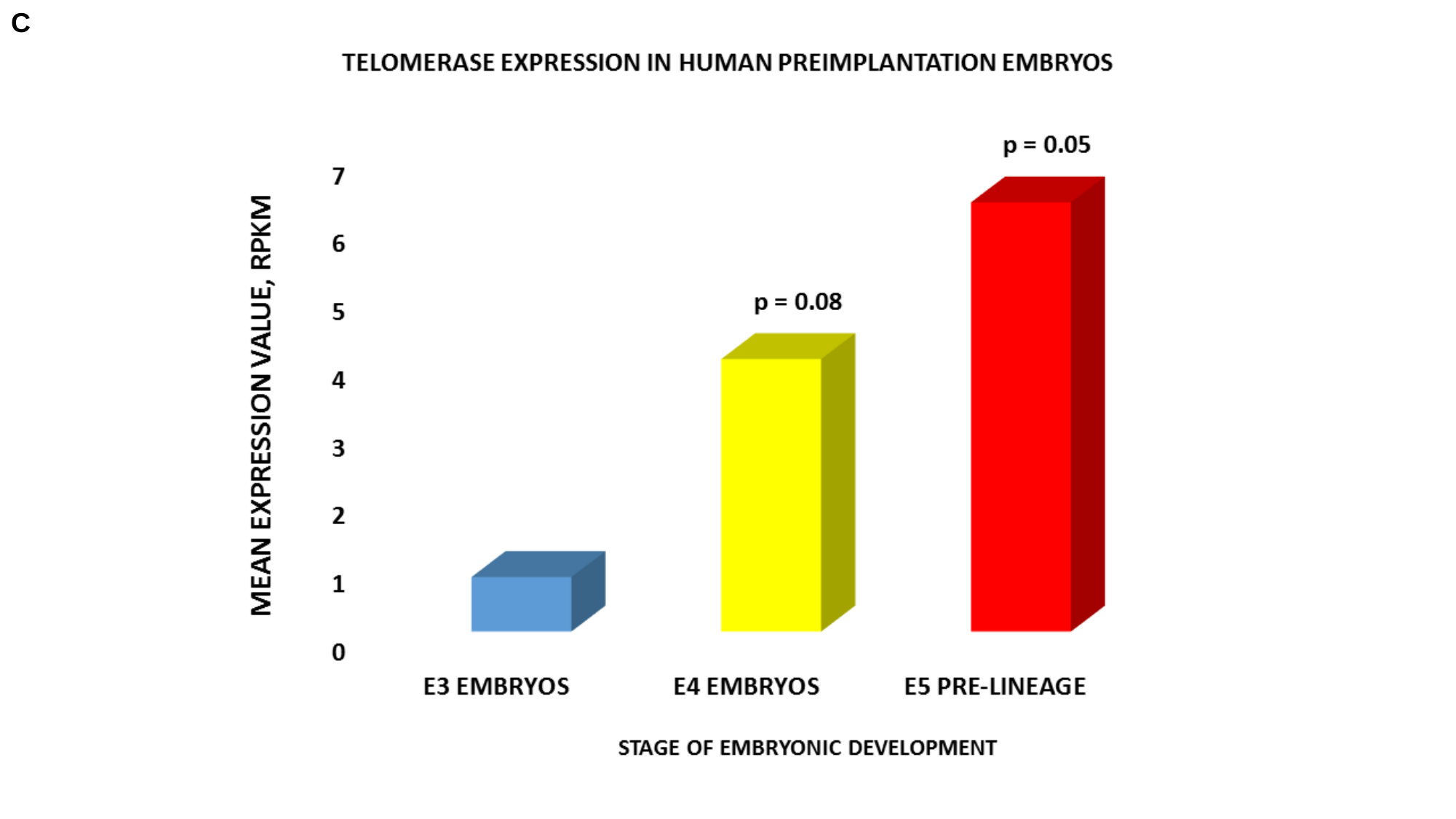

C

## Slide 5
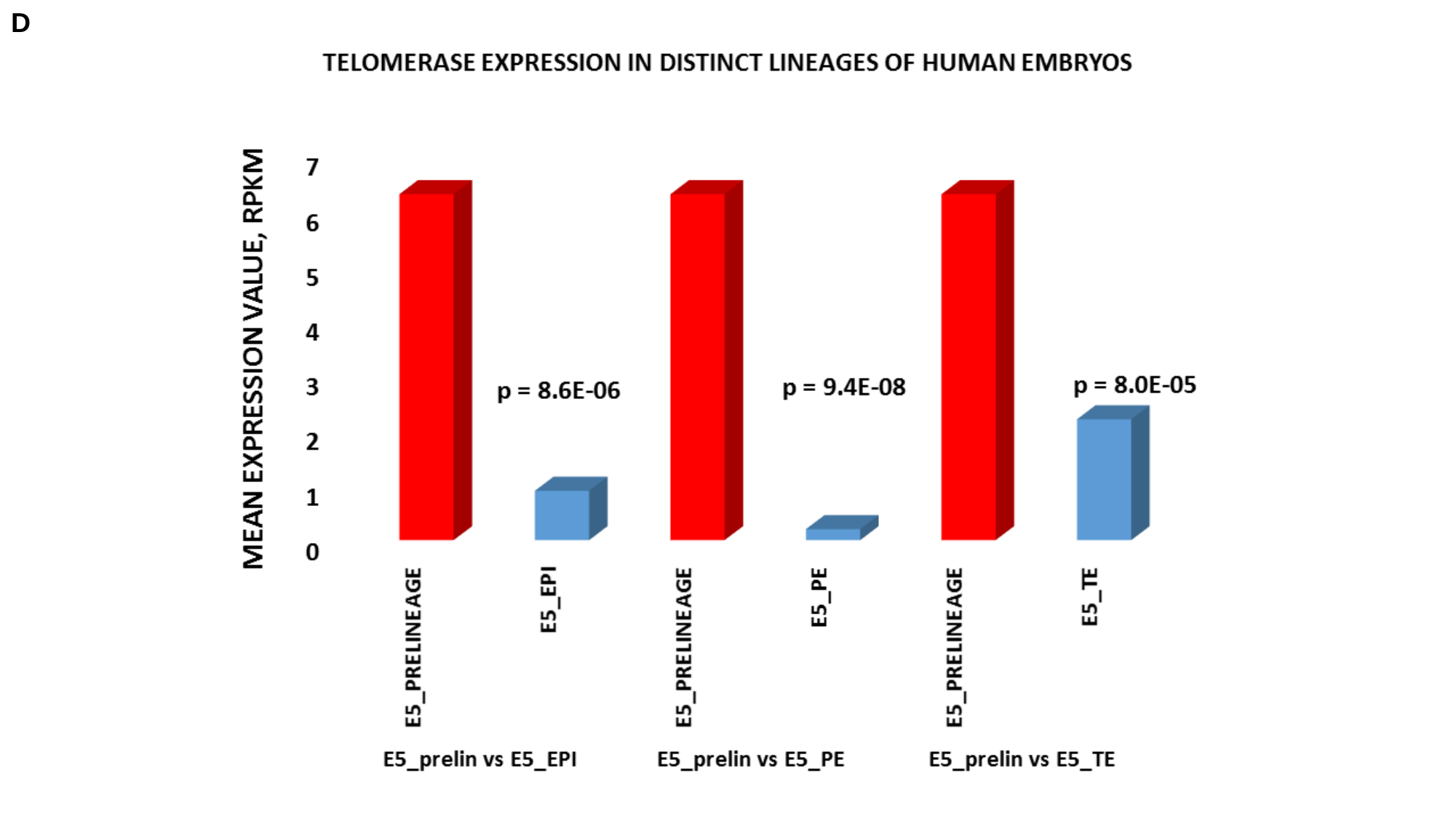

D

## Slide 6
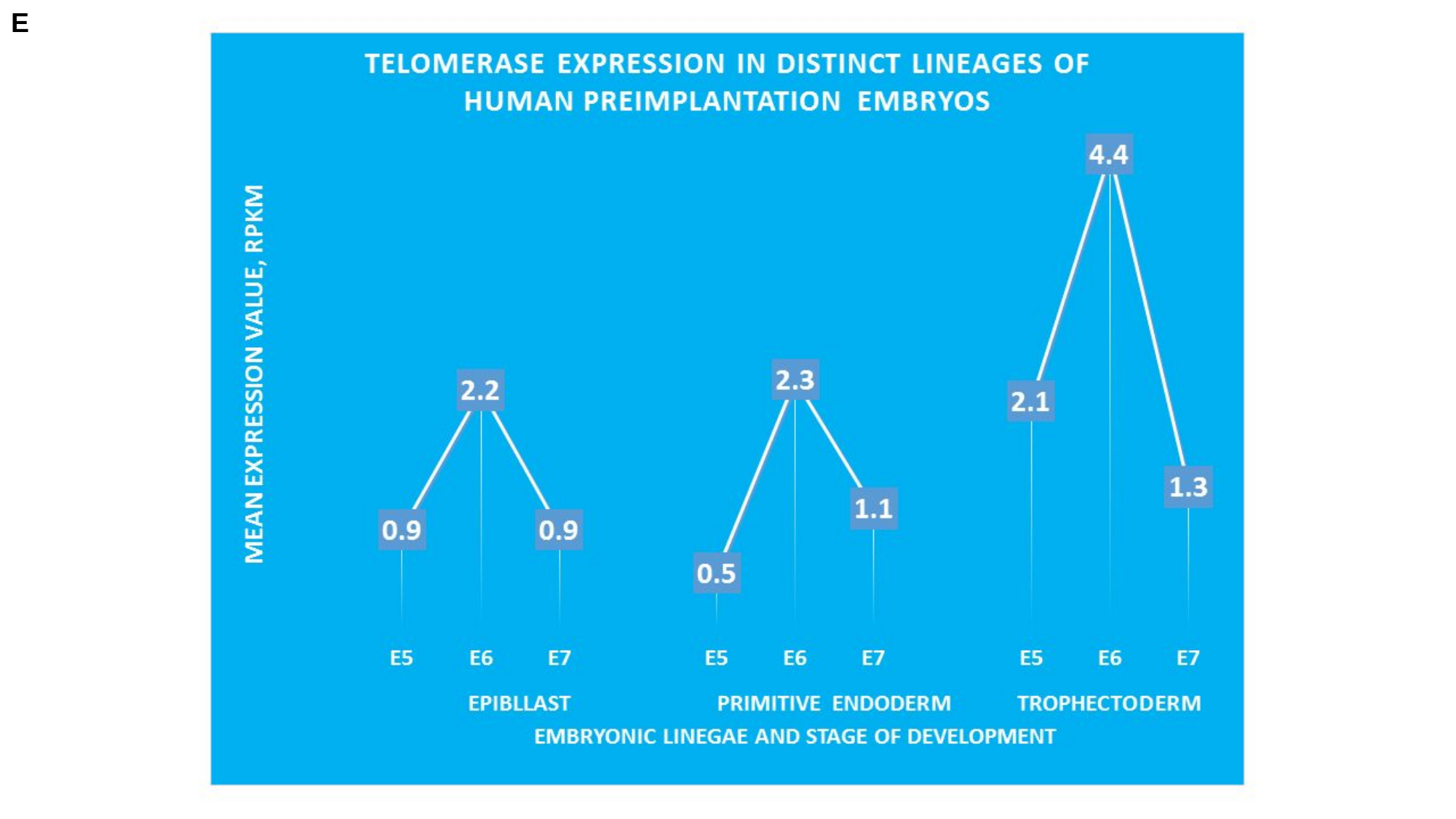

E

## Slide 7
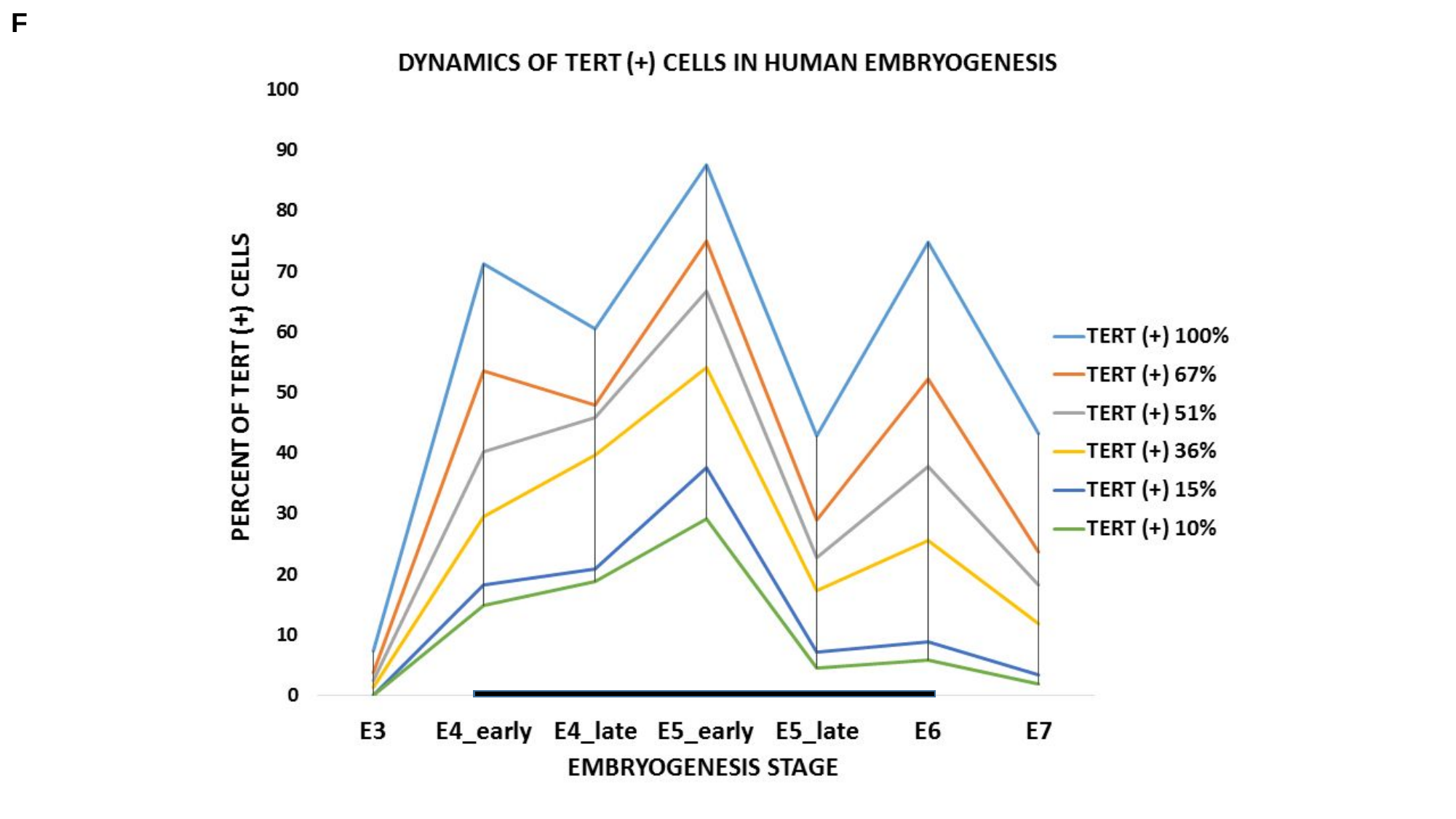

F
