# Supplementary material for: Single cell expression analysis of primate-specific retroviruses-derived HPAT lincRNAs in viable human blastocysts identifies embryonic cells co-expressing genetic markers of multiple lineages
Source: Heliyon. 2018 Jun 28;4(6):e00667. doi: 10.1016/j.heliyon.2018.e00667 (PMC6039856; doi:10.1016/j.heliyon.2018.e00667)
Supplement: Supplemental Figure S7 [file mmc10.pptx]

## Slide 1
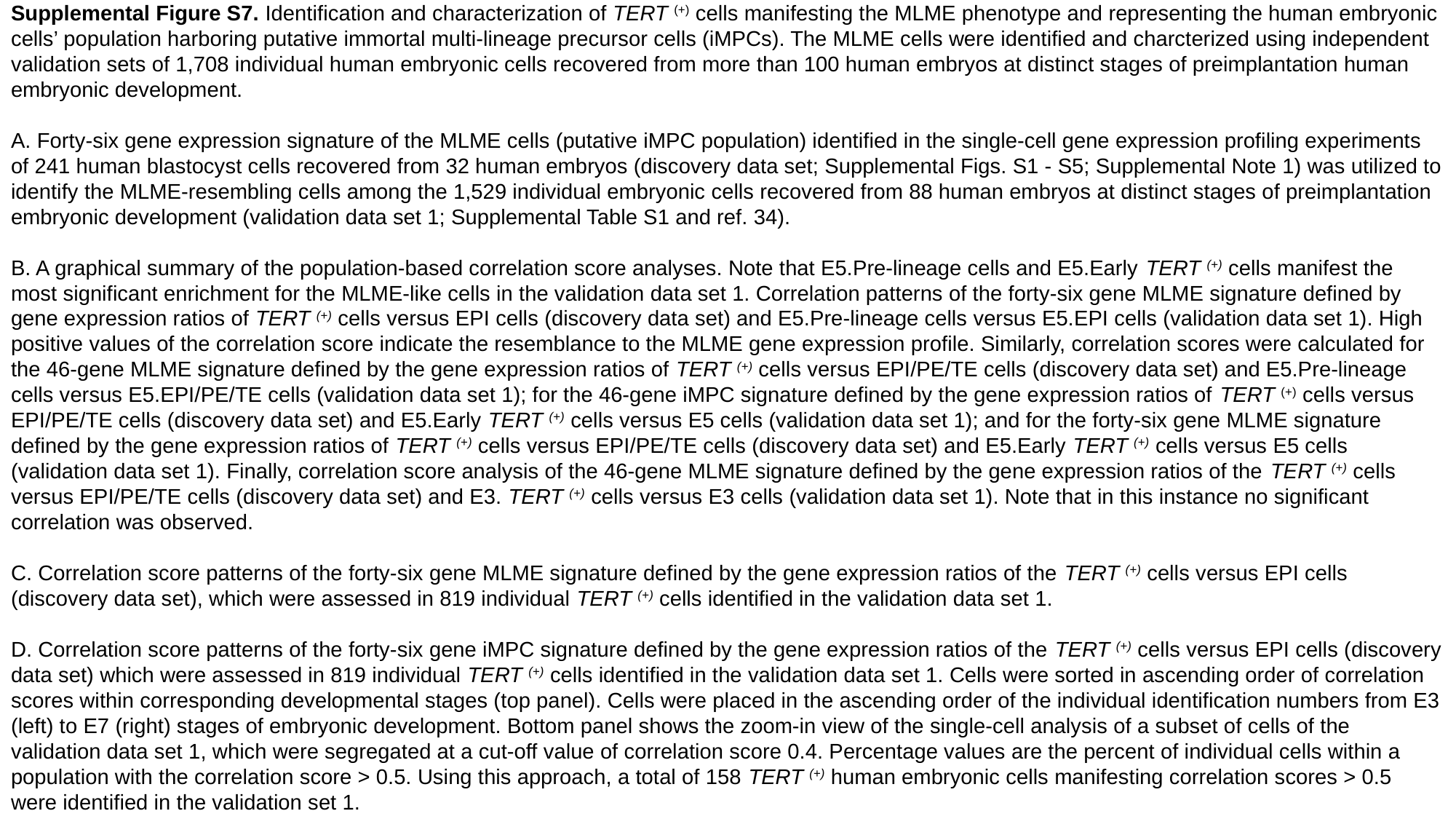

# Supplemental Figure S7. Identification and characterization of TERT (+) cells manifesting the MLME phenotype and representing the human embryonic cells’ population harboring putative immortal multi-lineage precursor cells (iMPCs). The MLME cells were identified and charcterized using independent validation sets of 1,708 individual human embryonic cells recovered from more than 100 human embryos at distinct stages of preimplantation human embryonic development. A. Forty-six gene expression signature of the MLME cells (putative iMPC population) identified in the single-cell gene expression profiling experiments of 241 human blastocyst cells recovered from 32 human embryos (discovery data set; Supplemental Figs. S1 - S5; Supplemental Note 1) was utilized to identify the MLME-resembling cells among the 1,529 individual embryonic cells recovered from 88 human embryos at distinct stages of preimplantation embryonic development (validation data set 1; Supplemental Table S1 and ref. 34). B. A graphical summary of the population-based correlation score analyses. Note that E5.Pre-lineage cells and E5.Early TERT (+) cells manifest the most significant enrichment for the MLME-like cells in the validation data set 1. Correlation patterns of the forty-six gene MLME signature defined by gene expression ratios of TERT (+) cells versus EPI cells (discovery data set) and E5.Pre-lineage cells versus E5.EPI cells (validation data set 1). High positive values of the correlation score indicate the resemblance to the MLME gene expression profile. Similarly, correlation scores were calculated for the 46-gene MLME signature defined by the gene expression ratios of TERT (+) cells versus EPI/PE/TE cells (discovery data set) and E5.Pre-lineage cells versus E5.EPI/PE/TE cells (validation data set 1); for the 46-gene iMPC signature defined by the gene expression ratios of TERT (+) cells versus EPI/PE/TE cells (discovery data set) and E5.Early TERT (+) cells versus E5 cells (validation data set 1); and for the forty-six gene MLME signature defined by the gene expression ratios of TERT (+) cells versus EPI/PE/TE cells (discovery data set) and E5.Early TERT (+) cells versus E5 cells (validation data set 1). Finally, correlation score analysis of the 46-gene MLME signature defined by the gene expression ratios of the TERT (+) cells versus EPI/PE/TE cells (discovery data set) and E3. TERT (+) cells versus E3 cells (validation data set 1). Note that in this instance no significant correlation was observed. C. Correlation score patterns of the forty-six gene MLME signature defined by the gene expression ratios of the TERT (+) cells versus EPI cells (discovery data set), which were assessed in 819 individual TERT (+) cells identified in the validation data set 1. D. Correlation score patterns of the forty-six gene iMPC signature defined by the gene expression ratios of the TERT (+) cells versus EPI cells (discovery data set) which were assessed in 819 individual TERT (+) cells identified in the validation data set 1. Cells were sorted in ascending order of correlation scores within corresponding developmental stages (top panel). Cells were placed in the ascending order of the individual identification numbers from E3 (left) to E7 (right) stages of embryonic development. Bottom panel shows the zoom-in view of the single-cell analysis of a subset of cells of the validation data set 1, which were segregated at a cut-off value of correlation score 0.4. Percentage values are the percent of individual cells within a population with the correlation score > 0.5. Using this approach, a total of 158 TERT (+) human embryonic cells manifesting correlation scores > 0.5 were identified in the validation set 1.

## Slide 2
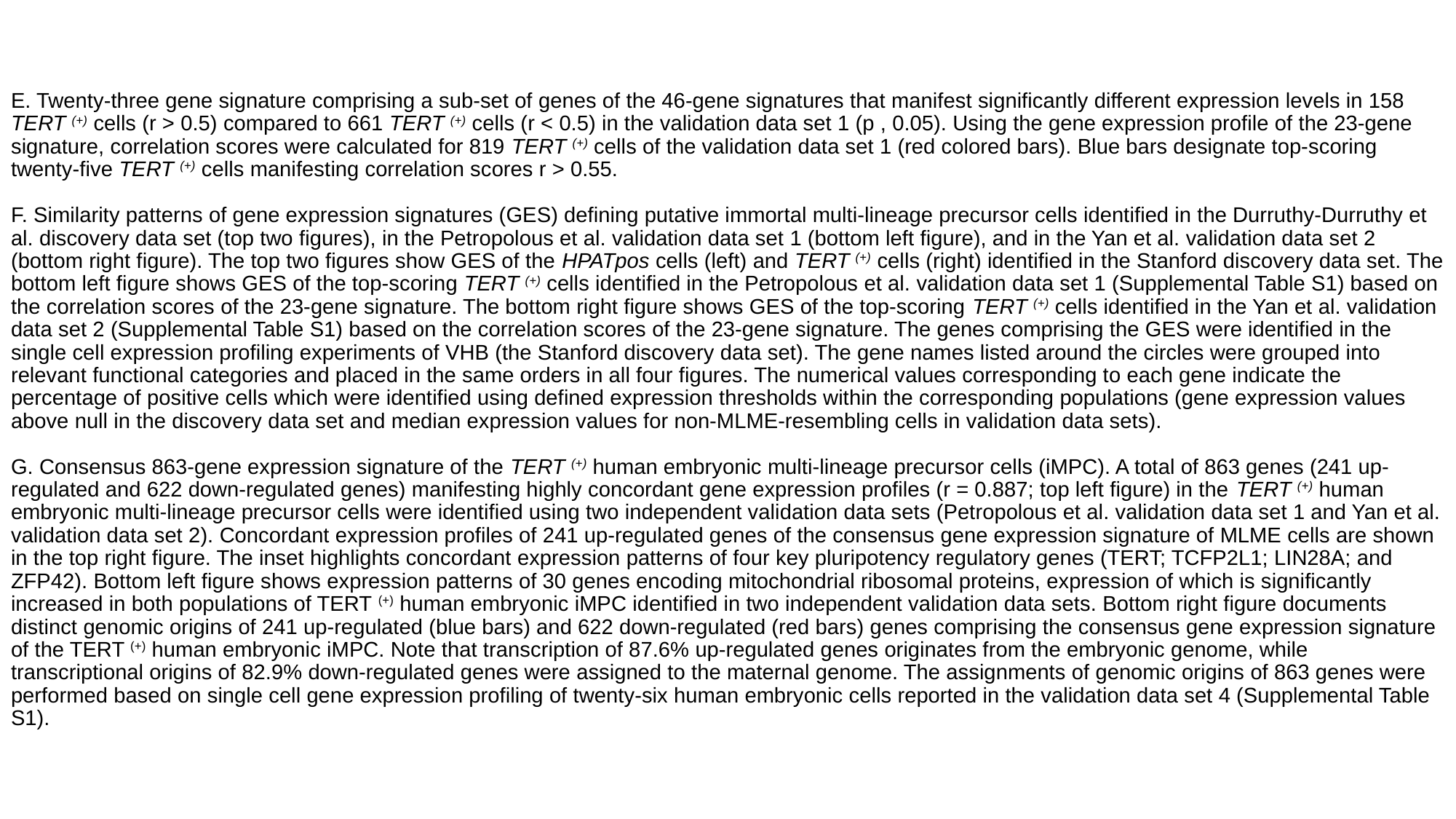

# E. Twenty-three gene signature comprising a sub-set of genes of the 46-gene signatures that manifest significantly different expression levels in 158 TERT (+) cells (r > 0.5) compared to 661 TERT (+) cells (r < 0.5) in the validation data set 1 (p , 0.05). Using the gene expression profile of the 23-gene signature, correlation scores were calculated for 819 TERT (+) cells of the validation data set 1 (red colored bars). Blue bars designate top-scoring twenty-five TERT (+) cells manifesting correlation scores r > 0.55. F. Similarity patterns of gene expression signatures (GES) defining putative immortal multi-lineage precursor cells identified in the Durruthy-Durruthy et al. discovery data set (top two figures), in the Petropolous et al. validation data set 1 (bottom left figure), and in the Yan et al. validation data set 2 (bottom right figure). The top two figures show GES of the HPATpos cells (left) and TERT (+) cells (right) identified in the Stanford discovery data set. The bottom left figure shows GES of the top-scoring TERT (+) cells identified in the Petropolous et al. validation data set 1 (Supplemental Table S1) based on the correlation scores of the 23-gene signature. The bottom right figure shows GES of the top-scoring TERT (+) cells identified in the Yan et al. validation data set 2 (Supplemental Table S1) based on the correlation scores of the 23-gene signature. The genes comprising the GES were identified in the single cell expression profiling experiments of VHB (the Stanford discovery data set). The gene names listed around the circles were grouped into relevant functional categories and placed in the same orders in all four figures. The numerical values corresponding to each gene indicate the percentage of positive cells which were identified using defined expression thresholds within the corresponding populations (gene expression values above null in the discovery data set and median expression values for non-MLME-resembling cells in validation data sets). G. Consensus 863-gene expression signature of the TERT (+) human embryonic multi-lineage precursor cells (iMPC). A total of 863 genes (241 up-regulated and 622 down-regulated genes) manifesting highly concordant gene expression profiles (r = 0.887; top left figure) in the TERT (+) human embryonic multi-lineage precursor cells were identified using two independent validation data sets (Petropolous et al. validation data set 1 and Yan et al. validation data set 2). Concordant expression profiles of 241 up-regulated genes of the consensus gene expression signature of MLME cells are shown in the top right figure. The inset highlights concordant expression patterns of four key pluripotency regulatory genes (TERT; TCFP2L1; LIN28A; and ZFP42). Bottom left figure shows expression patterns of 30 genes encoding mitochondrial ribosomal proteins, expression of which is significantly increased in both populations of TERT (+) human embryonic iMPC identified in two independent validation data sets. Bottom right figure documents distinct genomic origins of 241 up-regulated (blue bars) and 622 down-regulated (red bars) genes comprising the consensus gene expression signature of the TERT (+) human embryonic iMPC. Note that transcription of 87.6% up-regulated genes originates from the embryonic genome, while transcriptional origins of 82.9% down-regulated genes were assigned to the maternal genome. The assignments of genomic origins of 863 genes were performed based on single cell gene expression profiling of twenty-six human embryonic cells reported in the validation data set 4 (Supplemental Table S1).

## Slide 3
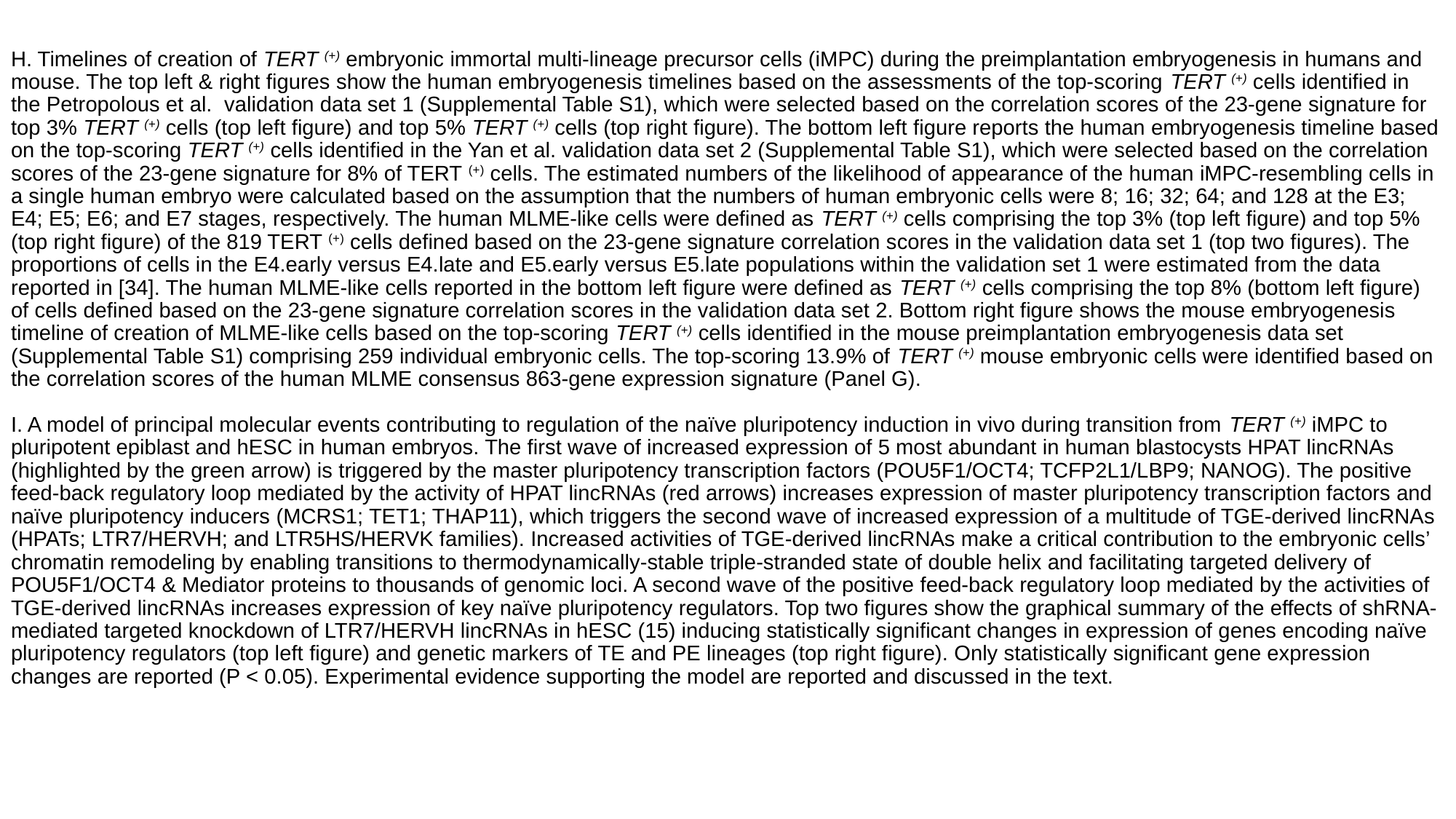

# H. Timelines of creation of TERT (+) embryonic immortal multi-lineage precursor cells (iMPC) during the preimplantation embryogenesis in humans and mouse. The top left & right figures show the human embryogenesis timelines based on the assessments of the top-scoring TERT (+) cells identified in the Petropolous et al. validation data set 1 (Supplemental Table S1), which were selected based on the correlation scores of the 23-gene signature for top 3% TERT (+) cells (top left figure) and top 5% TERT (+) cells (top right figure). The bottom left figure reports the human embryogenesis timeline based on the top-scoring TERT (+) cells identified in the Yan et al. validation data set 2 (Supplemental Table S1), which were selected based on the correlation scores of the 23-gene signature for 8% of TERT (+) cells. The estimated numbers of the likelihood of appearance of the human iMPC-resembling cells in a single human embryo were calculated based on the assumption that the numbers of human embryonic cells were 8; 16; 32; 64; and 128 at the E3; E4; E5; E6; and E7 stages, respectively. The human MLME-like cells were defined as TERT (+) cells comprising the top 3% (top left figure) and top 5% (top right figure) of the 819 TERT (+) cells defined based on the 23-gene signature correlation scores in the validation data set 1 (top two figures). The proportions of cells in the E4.early versus E4.late and E5.early versus E5.late populations within the validation set 1 were estimated from the data reported in [34]. The human MLME-like cells reported in the bottom left figure were defined as TERT (+) cells comprising the top 8% (bottom left figure) of cells defined based on the 23-gene signature correlation scores in the validation data set 2. Bottom right figure shows the mouse embryogenesis timeline of creation of MLME-like cells based on the top-scoring TERT (+) cells identified in the mouse preimplantation embryogenesis data set (Supplemental Table S1) comprising 259 individual embryonic cells. The top-scoring 13.9% of TERT (+) mouse embryonic cells were identified based on the correlation scores of the human MLME consensus 863-gene expression signature (Panel G). I. A model of principal molecular events contributing to regulation of the naïve pluripotency induction in vivo during transition from TERT (+) iMPC to pluripotent epiblast and hESC in human embryos. The first wave of increased expression of 5 most abundant in human blastocysts HPAT lincRNAs (highlighted by the green arrow) is triggered by the master pluripotency transcription factors (POU5F1/OCT4; TCFP2L1/LBP9; NANOG). The positive feed-back regulatory loop mediated by the activity of HPAT lincRNAs (red arrows) increases expression of master pluripotency transcription factors and naïve pluripotency inducers (MCRS1; TET1; THAP11), which triggers the second wave of increased expression of a multitude of TGE-derived lincRNAs (HPATs; LTR7/HERVH; and LTR5HS/HERVK families). Increased activities of TGE-derived lincRNAs make a critical contribution to the embryonic cells’ chromatin remodeling by enabling transitions to thermodynamically-stable triple-stranded state of double helix and facilitating targeted delivery of POU5F1/OCT4 & Mediator proteins to thousands of genomic loci. A second wave of the positive feed-back regulatory loop mediated by the activities of TGE-derived lincRNAs increases expression of key naïve pluripotency regulators. Top two figures show the graphical summary of the effects of shRNA-mediated targeted knockdown of LTR7/HERVH lincRNAs in hESC (15) inducing statistically significant changes in expression of genes encoding naïve pluripotency regulators (top left figure) and genetic markers of TE and PE lineages (top right figure). Only statistically significant gene expression changes are reported (P < 0.05). Experimental evidence supporting the model are reported and discussed in the text.

## Slide 4
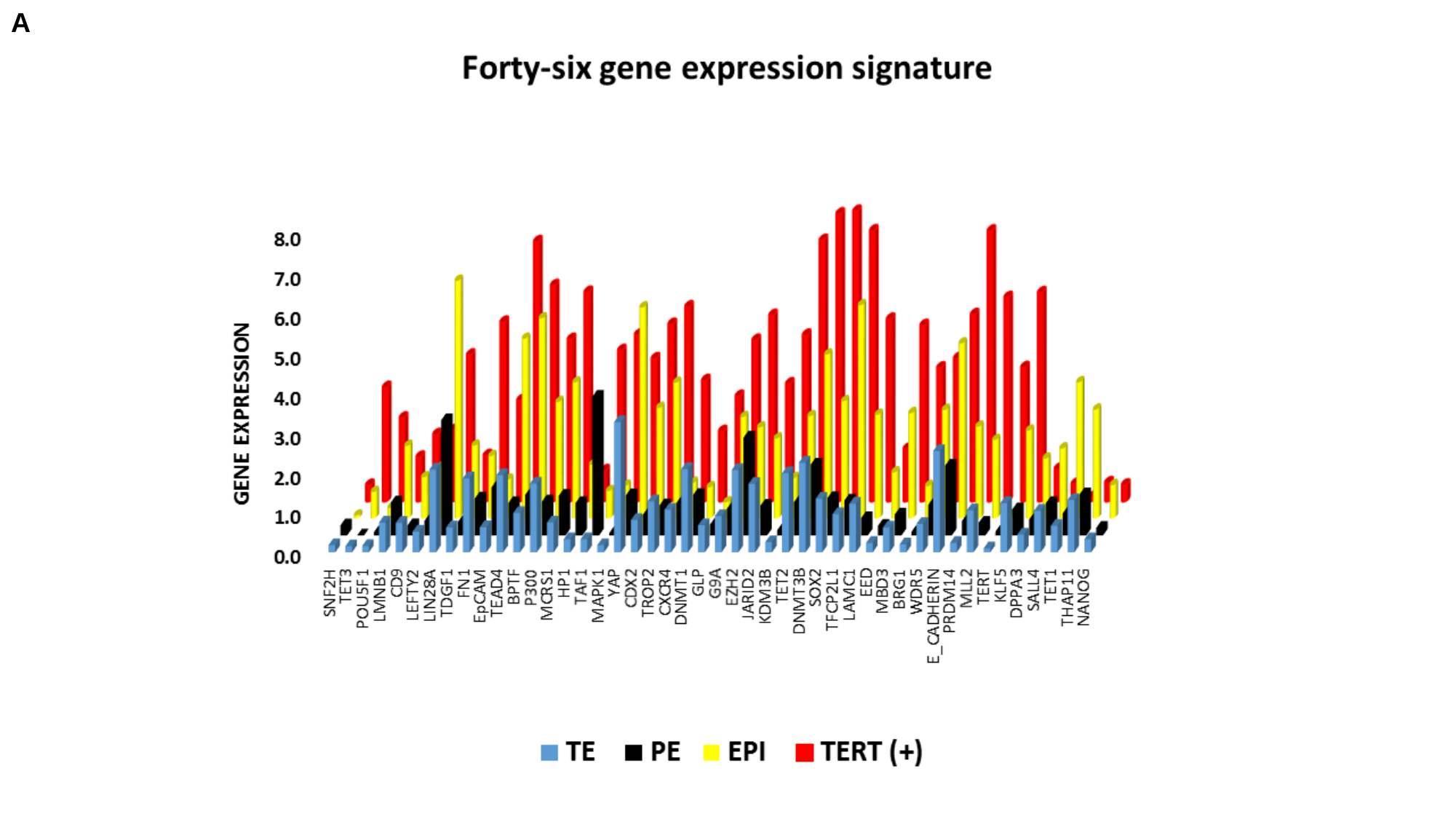

A

## Slide 5
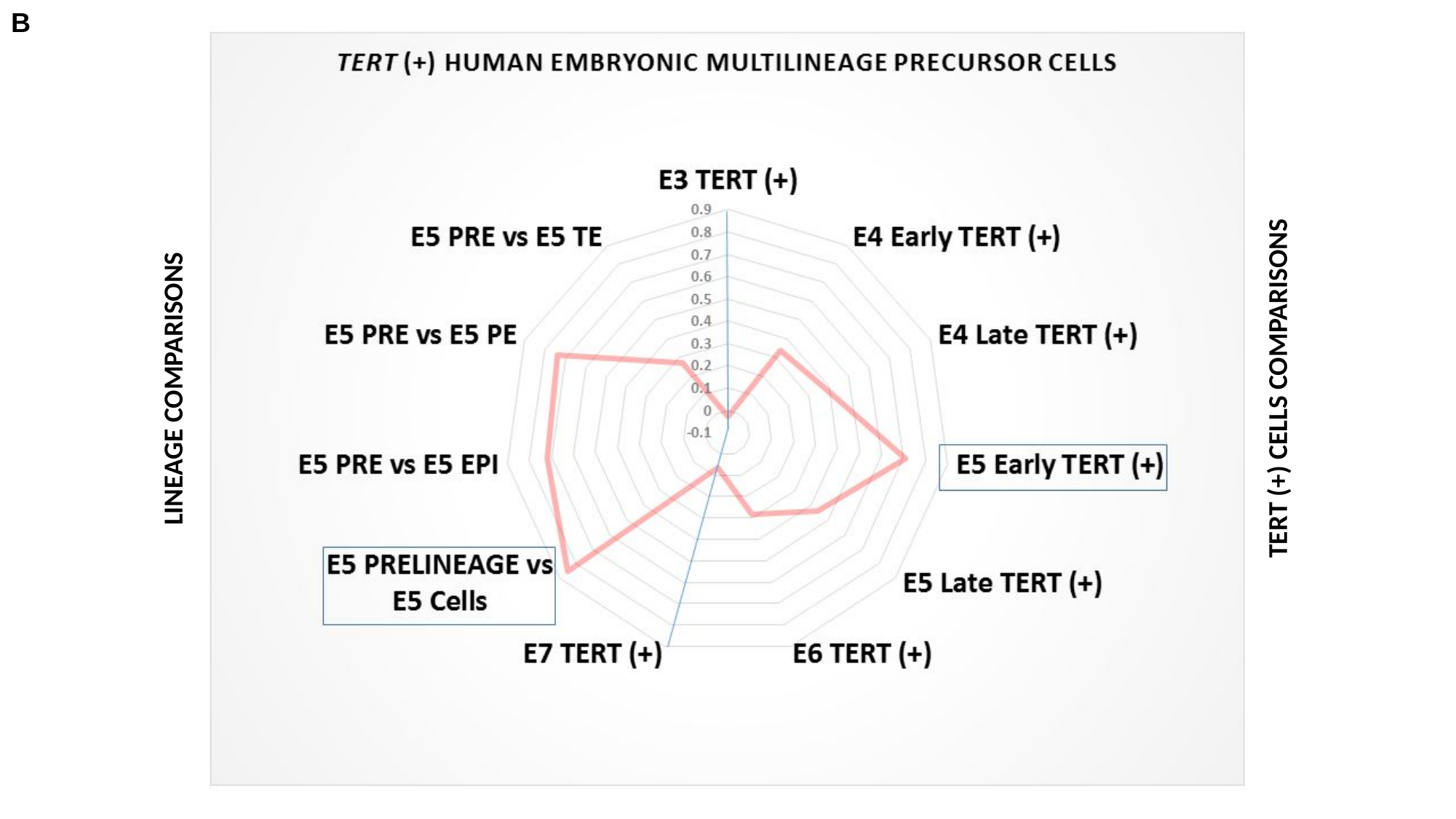

B
TERT (+) CELLS COMPARISONS
LINEAGE COMPARISONS

## Slide 6
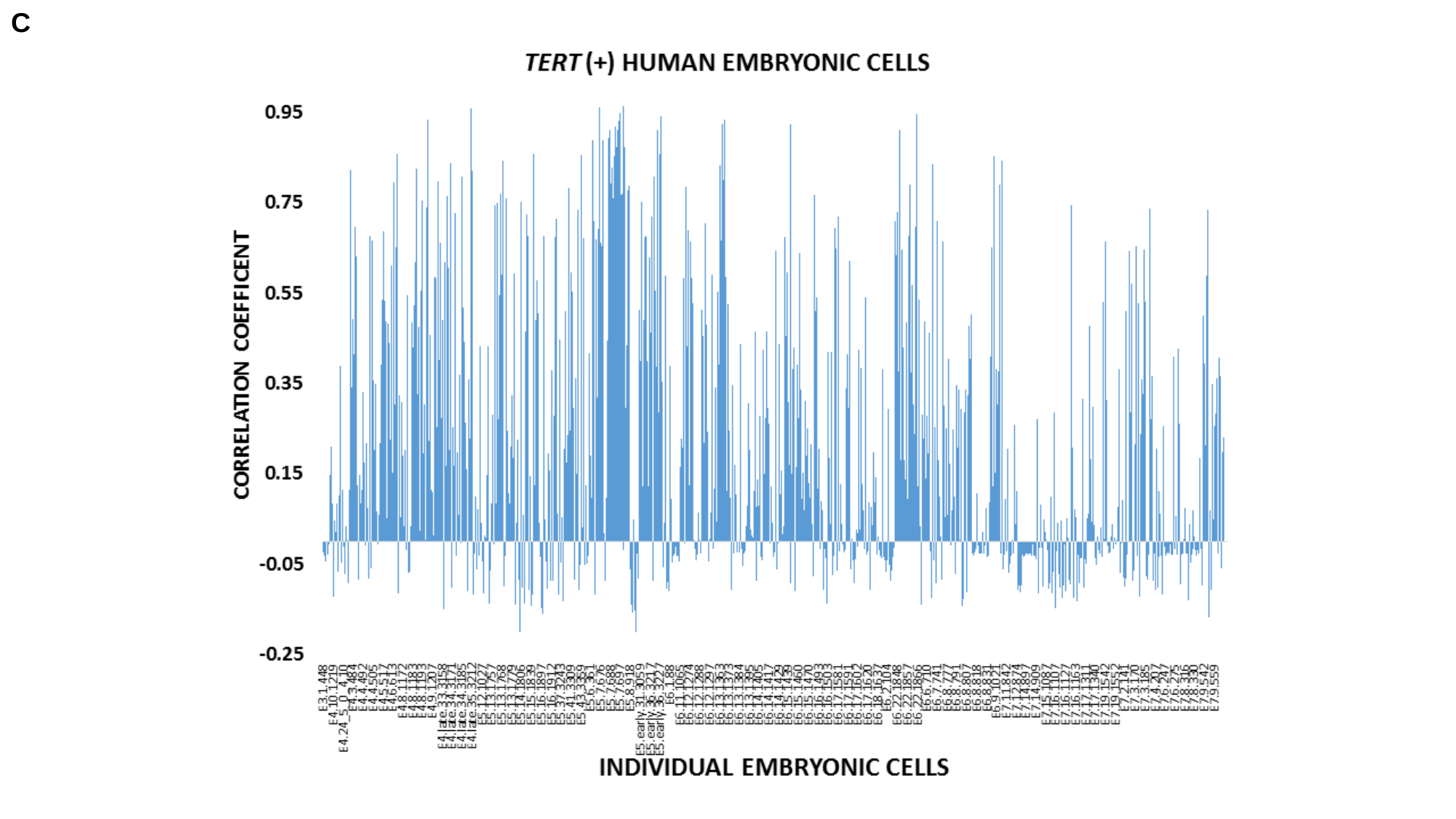

C

## Slide 7
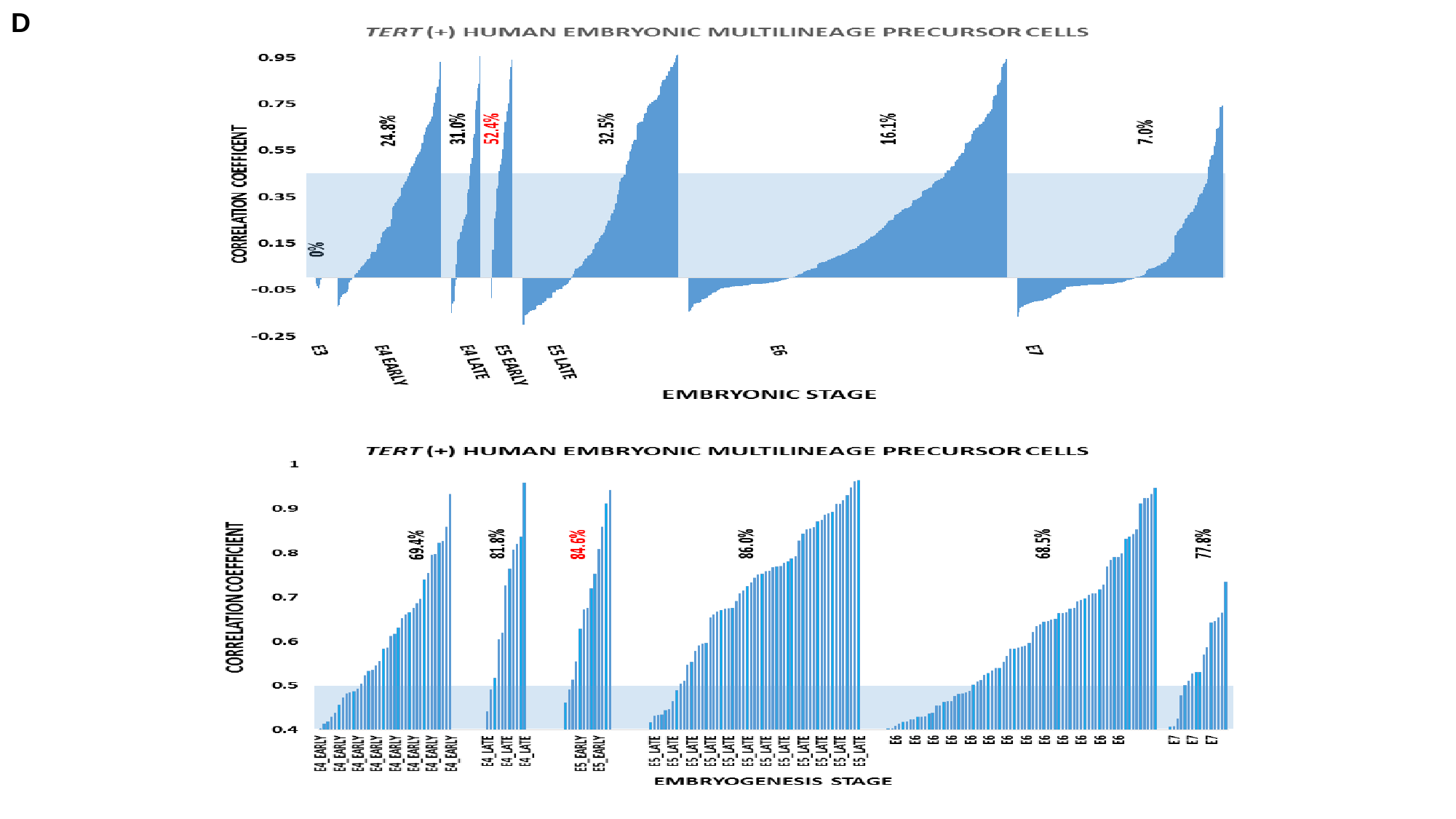

D

## Slide 8
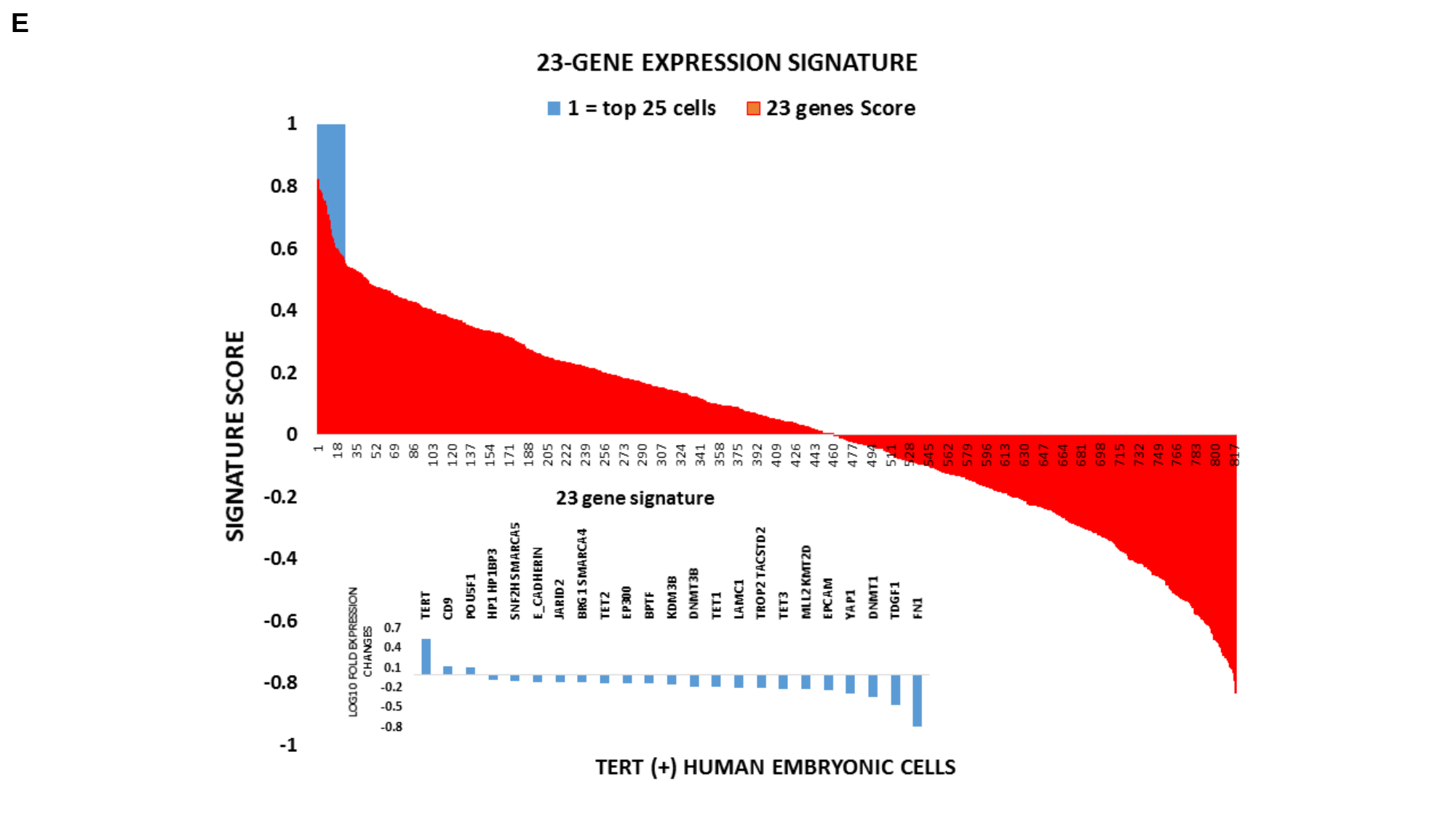

E

## Slide 9
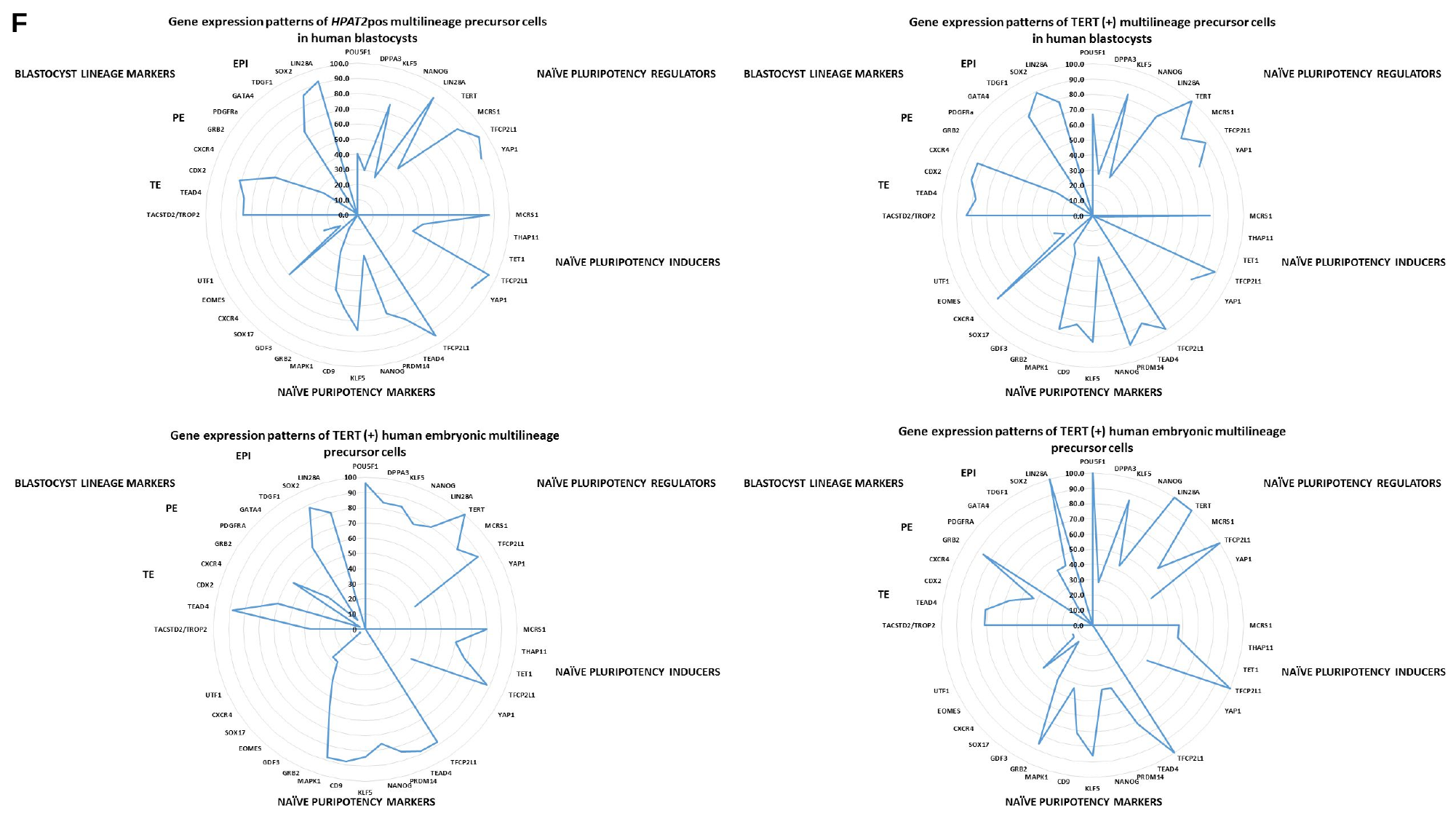

F

## Slide 10
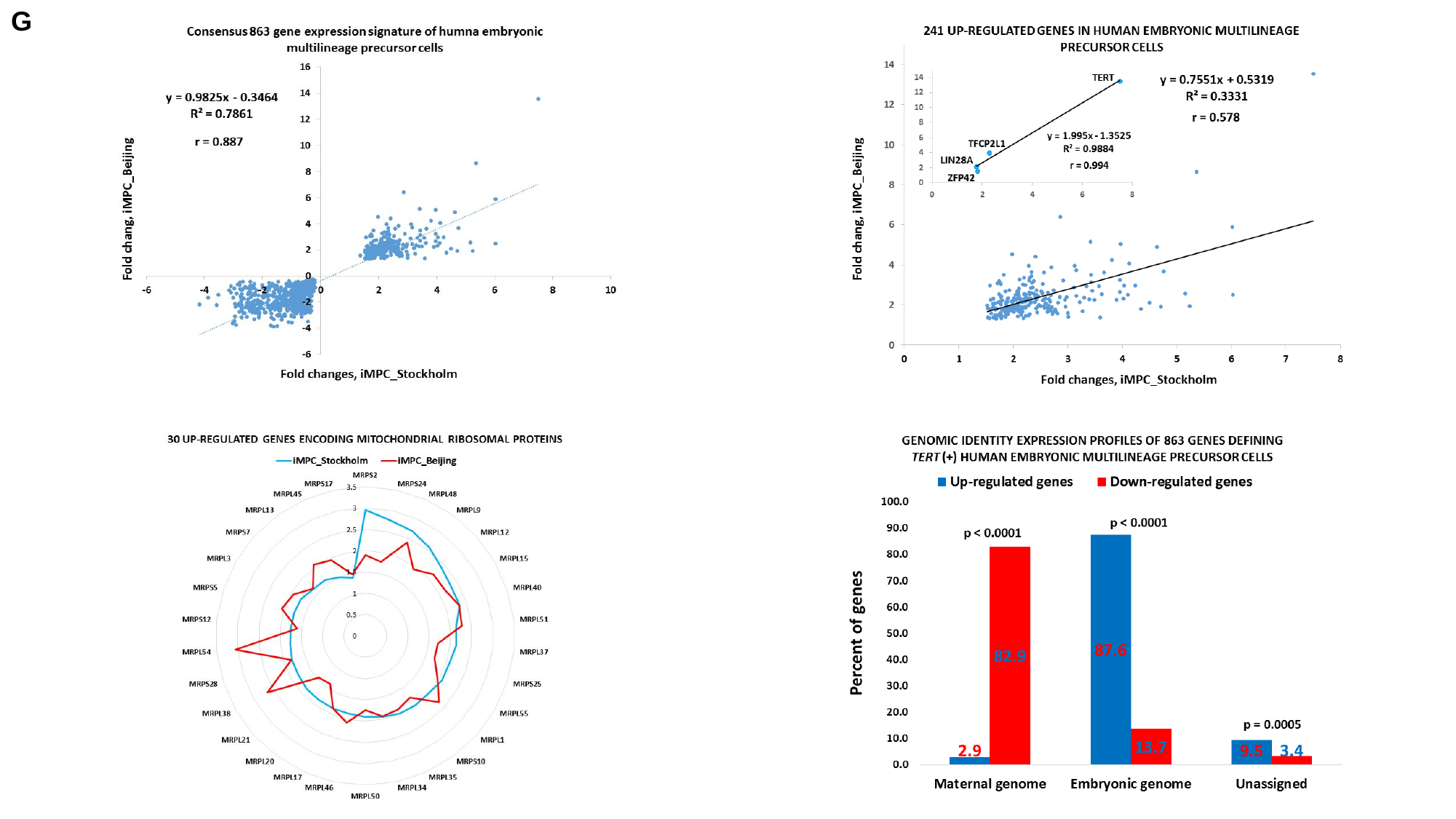

G

## Slide 11
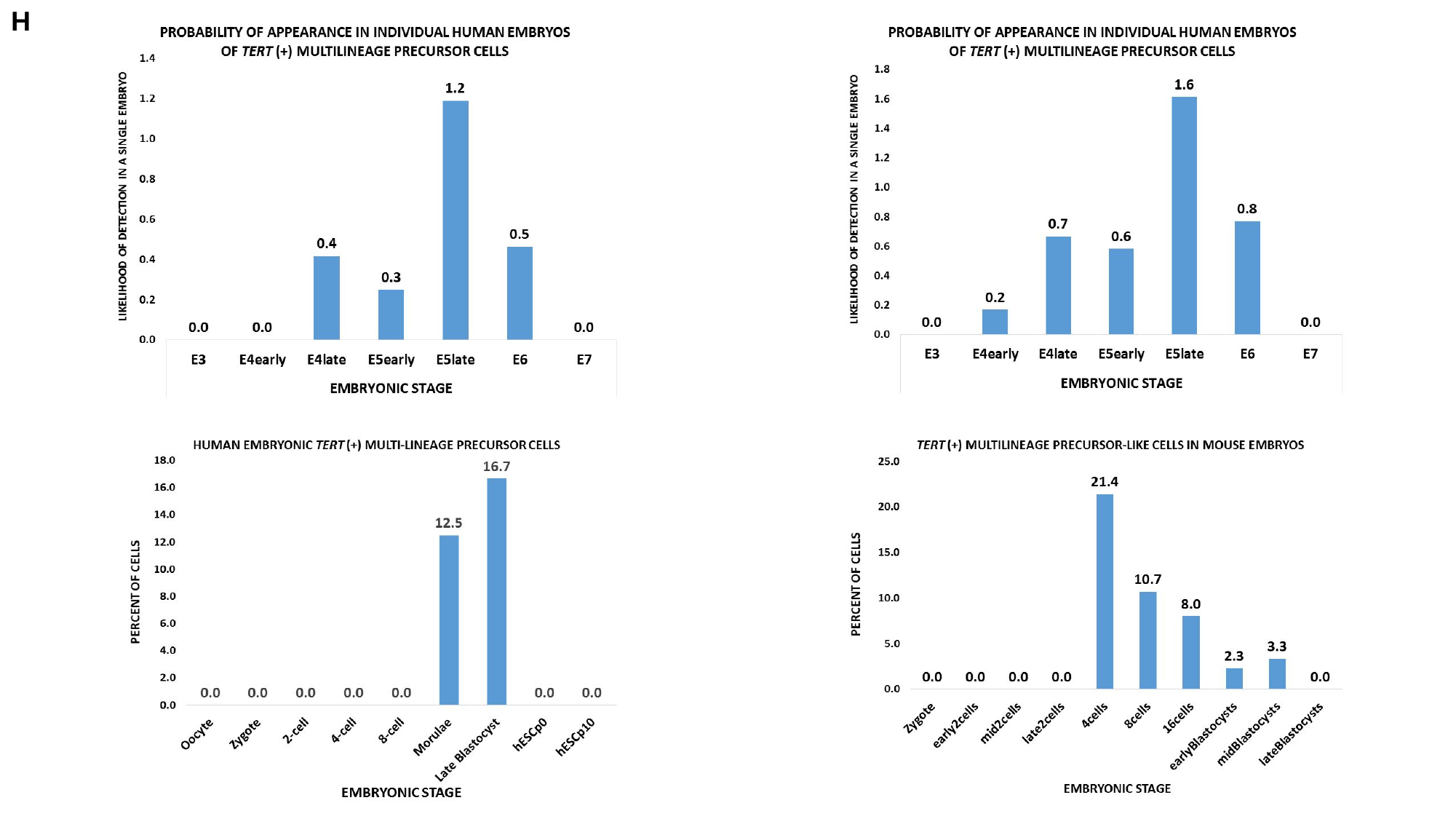

H

## Slide 12
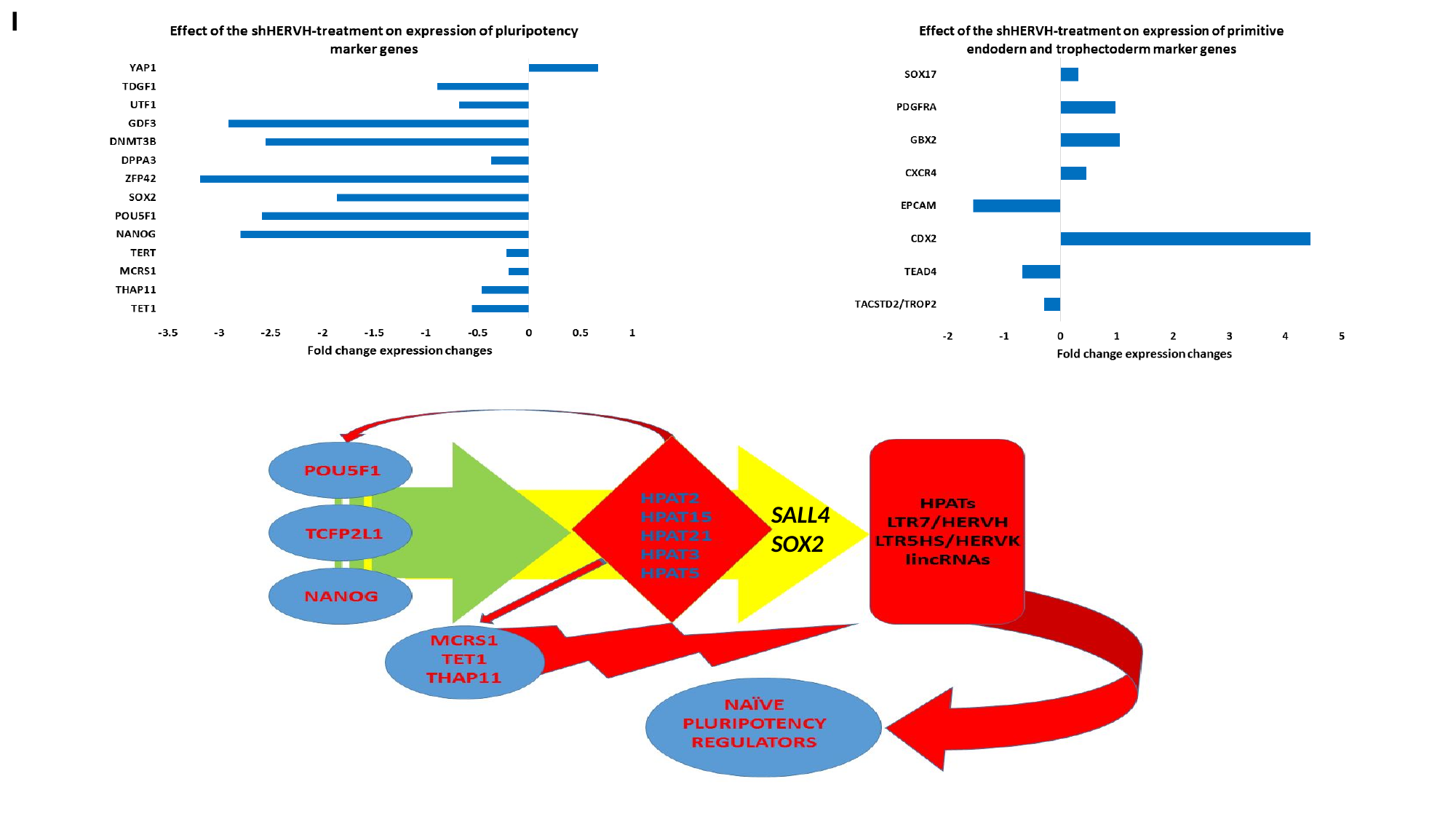

I
SALL4
SOX2
