# Supplementary figures and images for: Single cell expression analysis of primate-specific retroviruses-derived HPAT lincRNAs in viable human blastocysts identifies embryonic cells co-expressing genetic markers of multiple lineages
Source: Heliyon. 2018 Jun 28;4(6):e00667. doi: 10.1016/j.heliyon.2018.e00667 (PMC6039856; doi:10.1016/j.heliyon.2018.e00667)

## iMPC\_Stockholm - GO Biological Processes

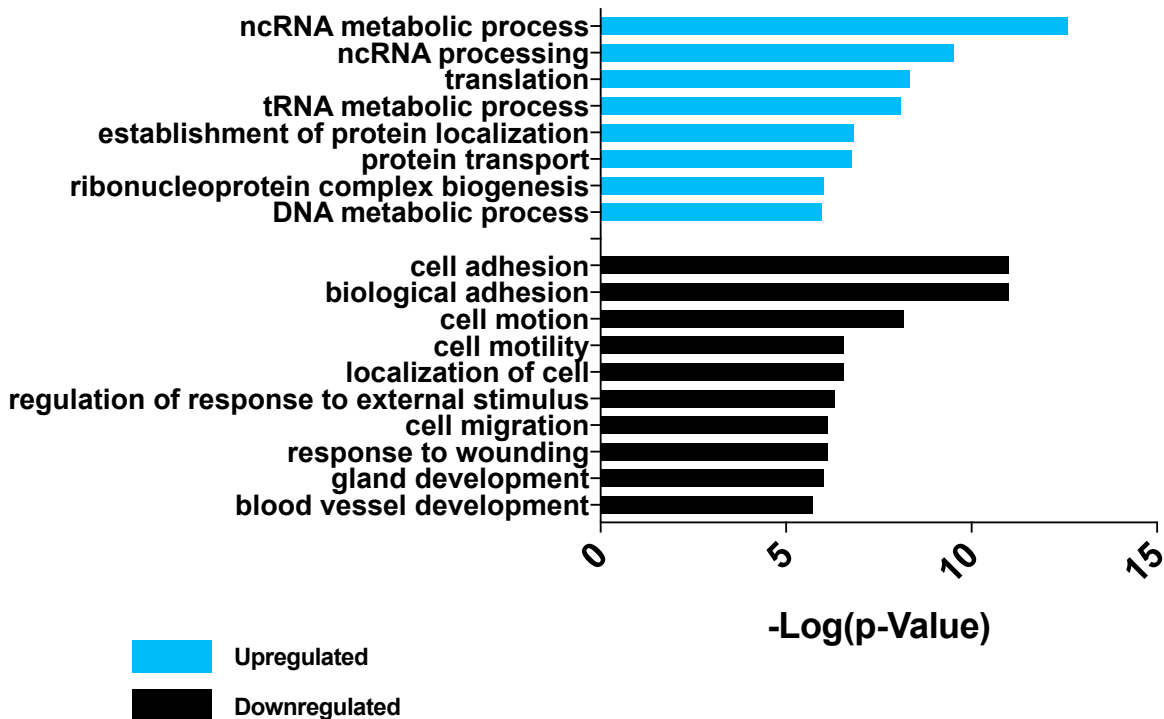

## iMPC\_Cosensus - GO Biological Processes

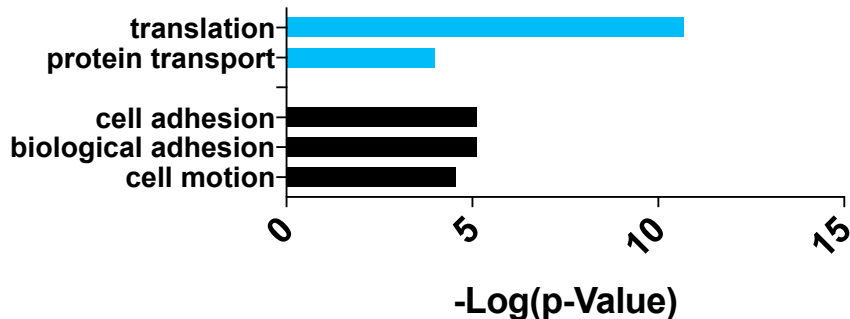

Supplement: Supplemental Figure S8 [file mmc11.pdf]
