# Supplementary material for: Single cell expression analysis of primate-specific retroviruses-derived HPAT lincRNAs in viable human blastocysts identifies embryonic cells co-expressing genetic markers of multiple lineages
Source: Heliyon. 2018 Jun 28;4(6):e00667. doi: 10.1016/j.heliyon.2018.e00667 (PMC6039856; doi:10.1016/j.heliyon.2018.e00667)
Supplement: Supplemental Figure S9 [file mmc12.pptx]

## Slide 1
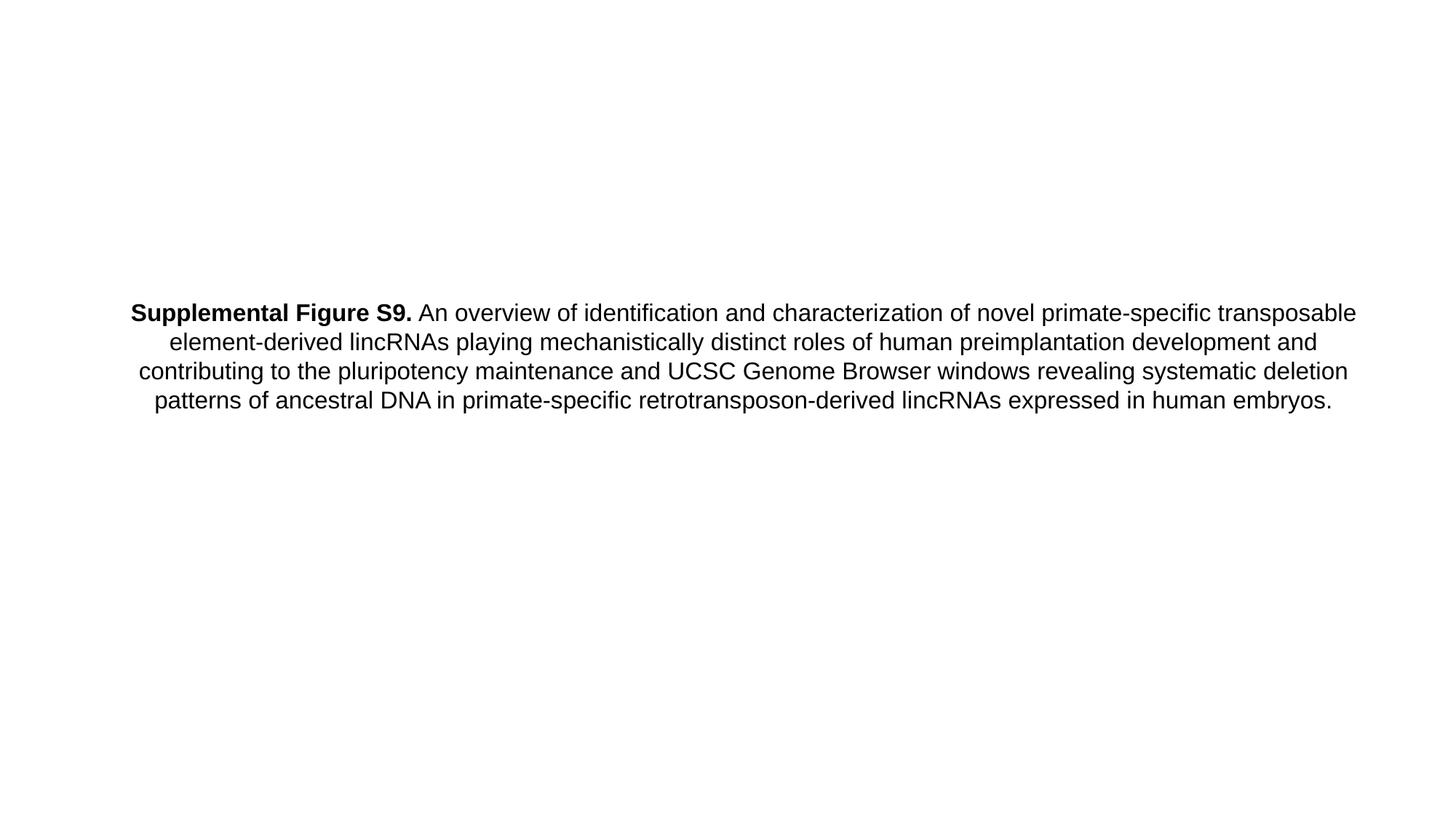

Supplemental Figure S9. An overview of identification and characterization of novel primate-specific transposable element-derived lincRNAs playing mechanistically distinct roles of human preimplantation development and contributing to the pluripotency maintenance and UCSC Genome Browser windows revealing systematic deletion patterns of ancestral DNA in primate-specific retrotransposon-derived lincRNAs expressed in human embryos.

## Slide 2
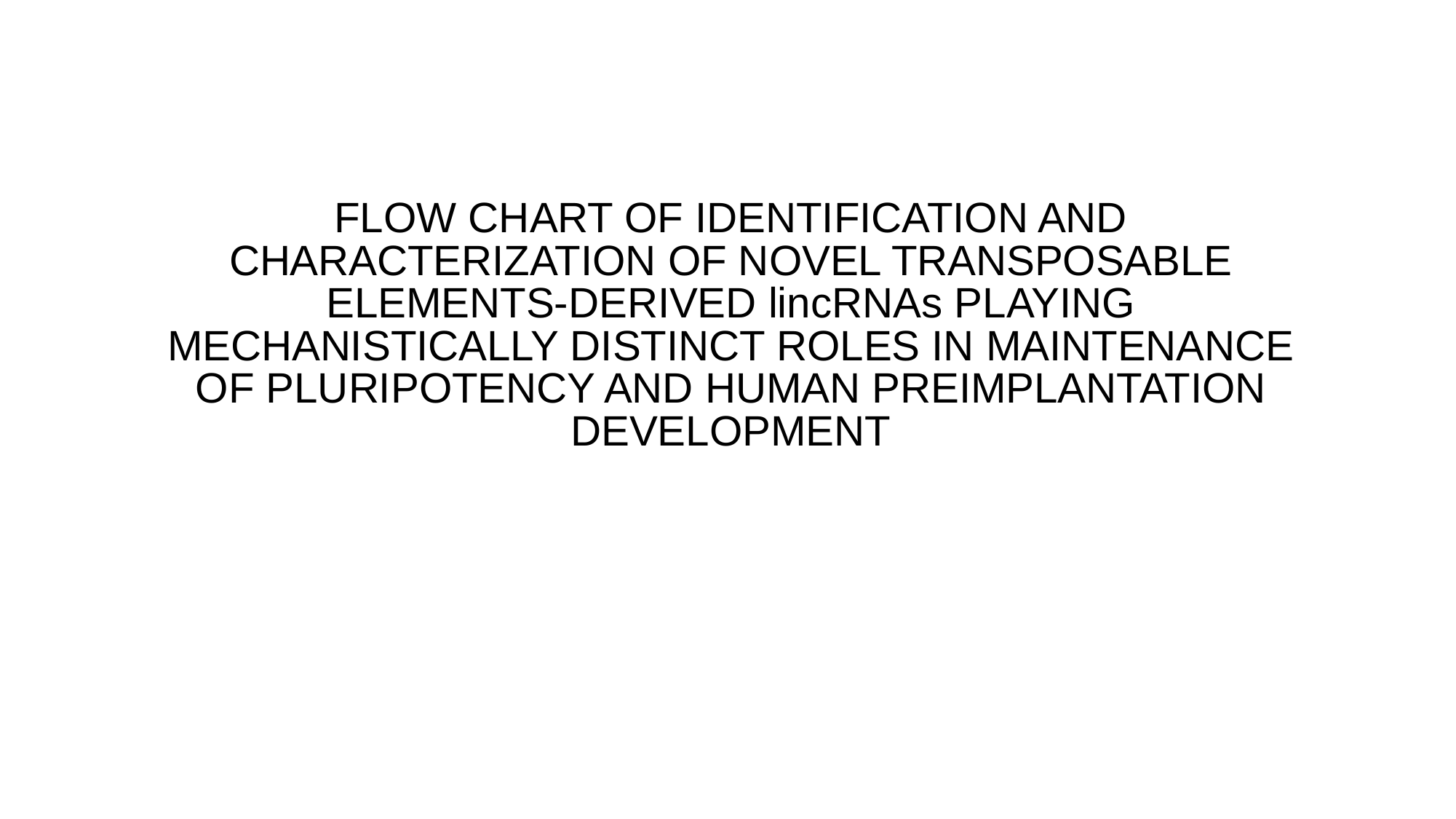

# FLOW CHART OF IDENTIFICATION AND CHARACTERIZATION OF NOVEL TRANSPOSABLE ELEMENTS-DERIVED lincRNAs PLAYING MECHANISTICALLY DISTINCT ROLES IN MAINTENANCE OF PLURIPOTENCY AND HUMAN PREIMPLANTATION DEVELOPMENT

## Slide 3
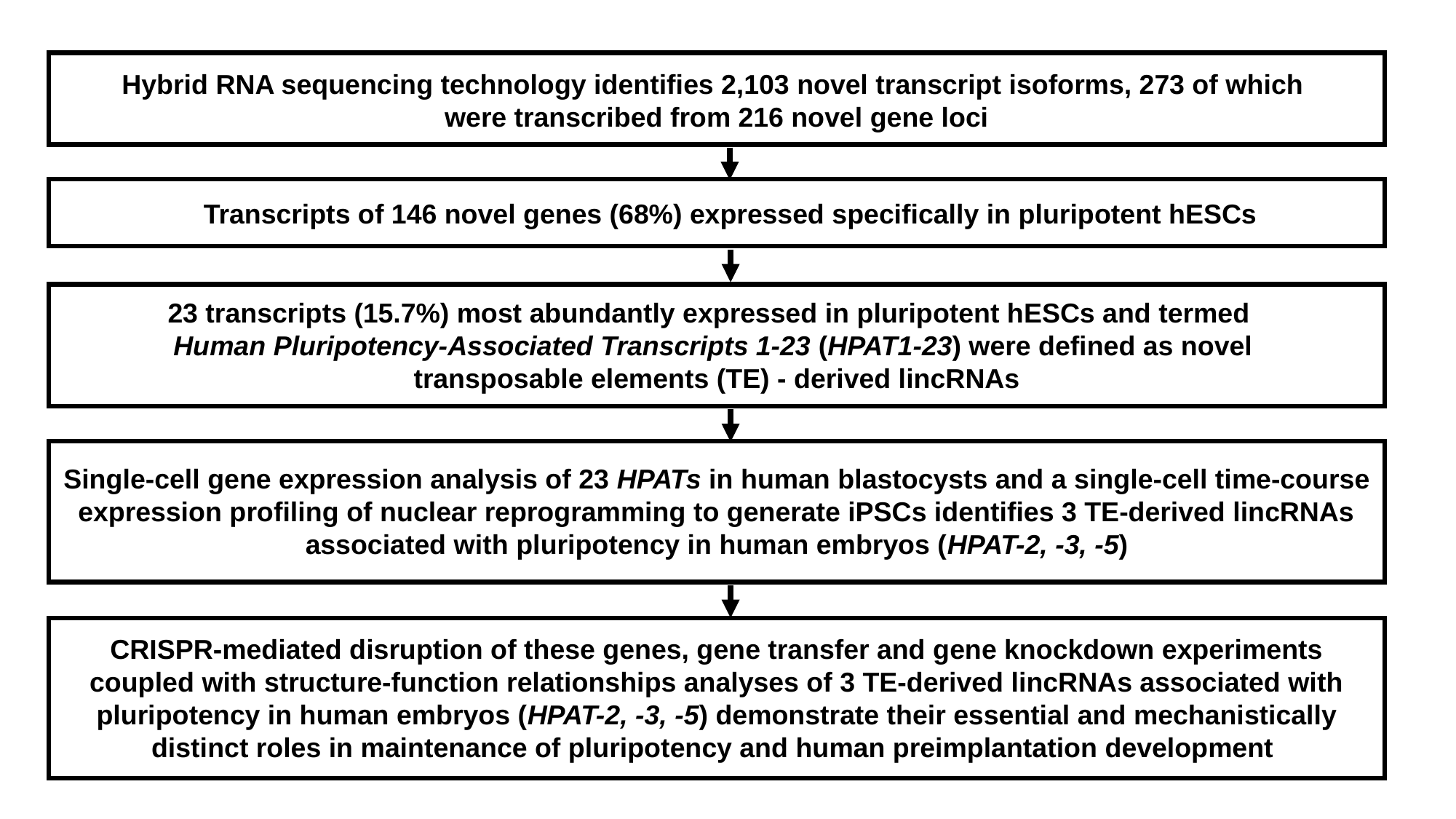

Hybrid RNA sequencing technology identifies 2,103 novel transcript isoforms, 273 of which
were transcribed from 216 novel gene loci
Transcripts of 146 novel genes (68%) expressed specifically in pluripotent hESCs
23 transcripts (15.7%) most abundantly expressed in pluripotent hESCs and termed
Human Pluripotency-Associated Transcripts 1-23 (HPAT1-23) were defined as novel
transposable elements (TE) - derived lincRNAs
Single-cell gene expression analysis of 23 HPATs in human blastocysts and a single-cell time-course expression profiling of nuclear reprogramming to generate iPSCs identifies 3 TE-derived lincRNAs associated with pluripotency in human embryos (HPAT-2, -3, -5)
CRISPR-mediated disruption of these genes, gene transfer and gene knockdown experiments coupled with structure-function relationships analyses of 3 TE-derived lincRNAs associated with pluripotency in human embryos (HPAT-2, -3, -5) demonstrate their essential and mechanistically distinct roles in maintenance of pluripotency and human preimplantation development

## Slide 4
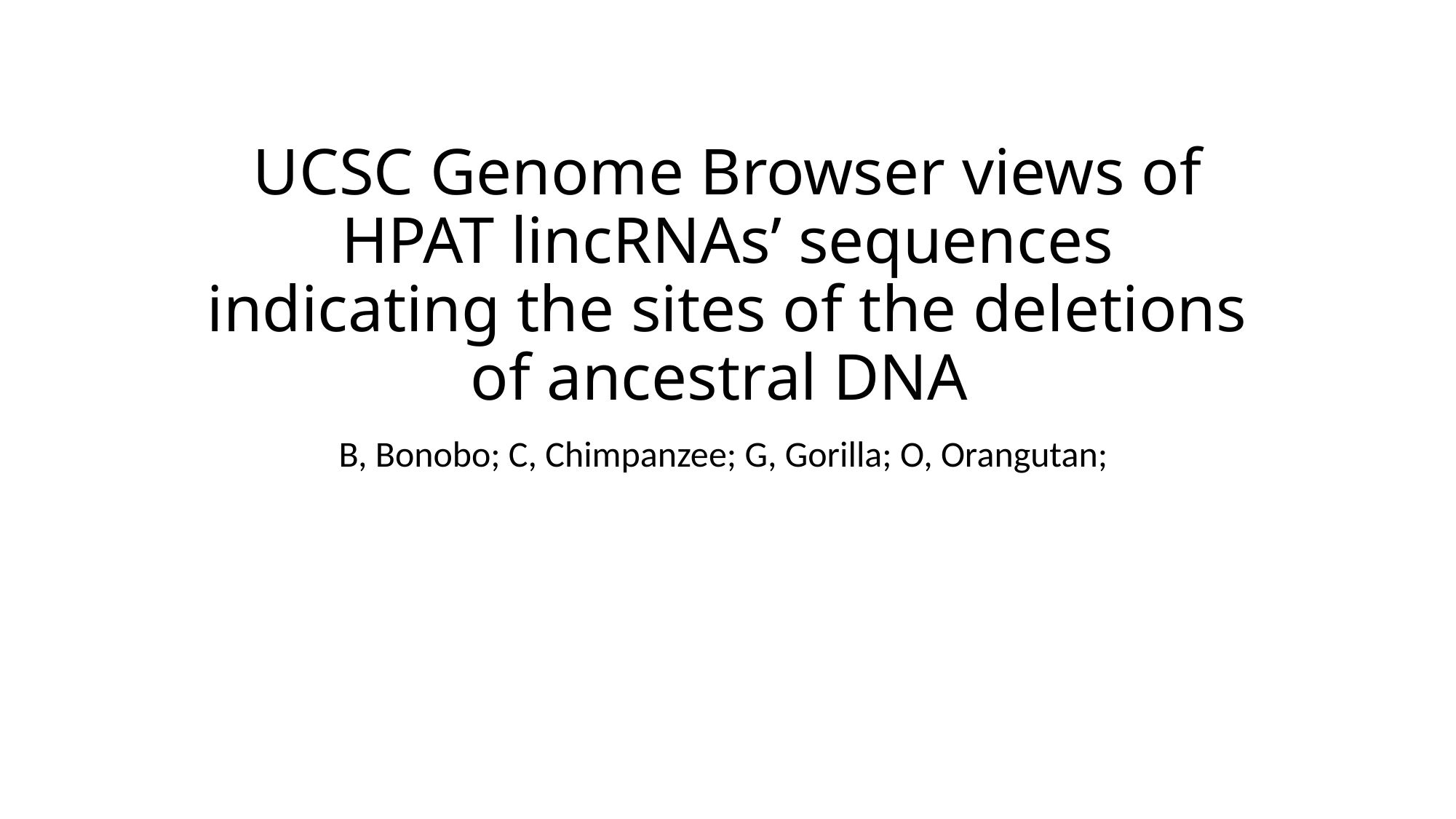

# UCSC Genome Browser views of HPAT lincRNAs’ sequences indicating the sites of the deletions of ancestral DNA
B, Bonobo; C, Chimpanzee; G, Gorilla; O, Orangutan;

## Slide 5
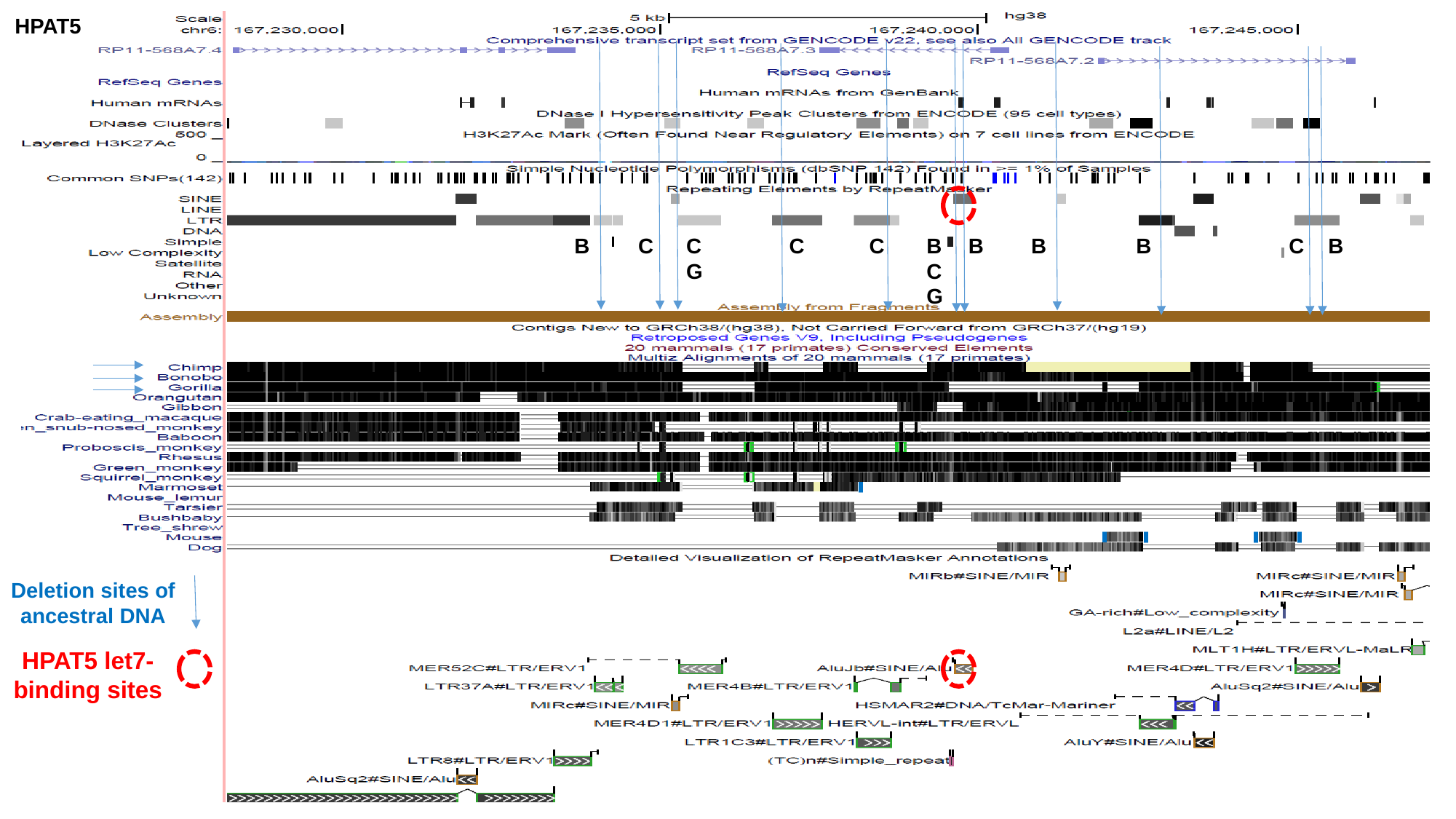

HPAT5
B
B
C
B
B
C
C
B
C
G
C
B
C
G
Deletion sites of ancestral DNA
HPAT5 let7-binding sites

## Slide 6
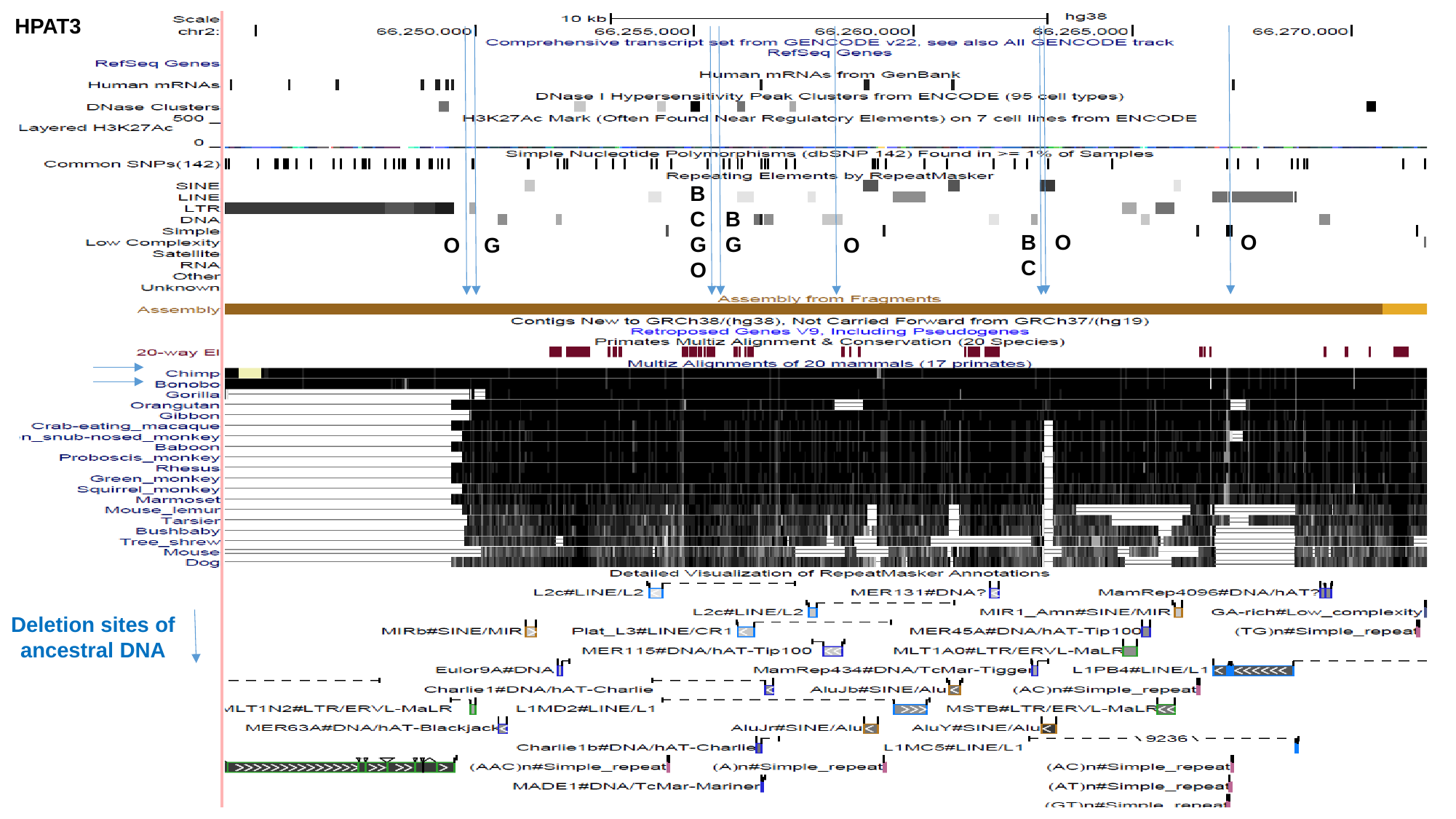

HPAT3
B
C
G
O
B
G
B
C
O
O
O
O
G
Deletion sites of ancestral DNA

## Slide 7
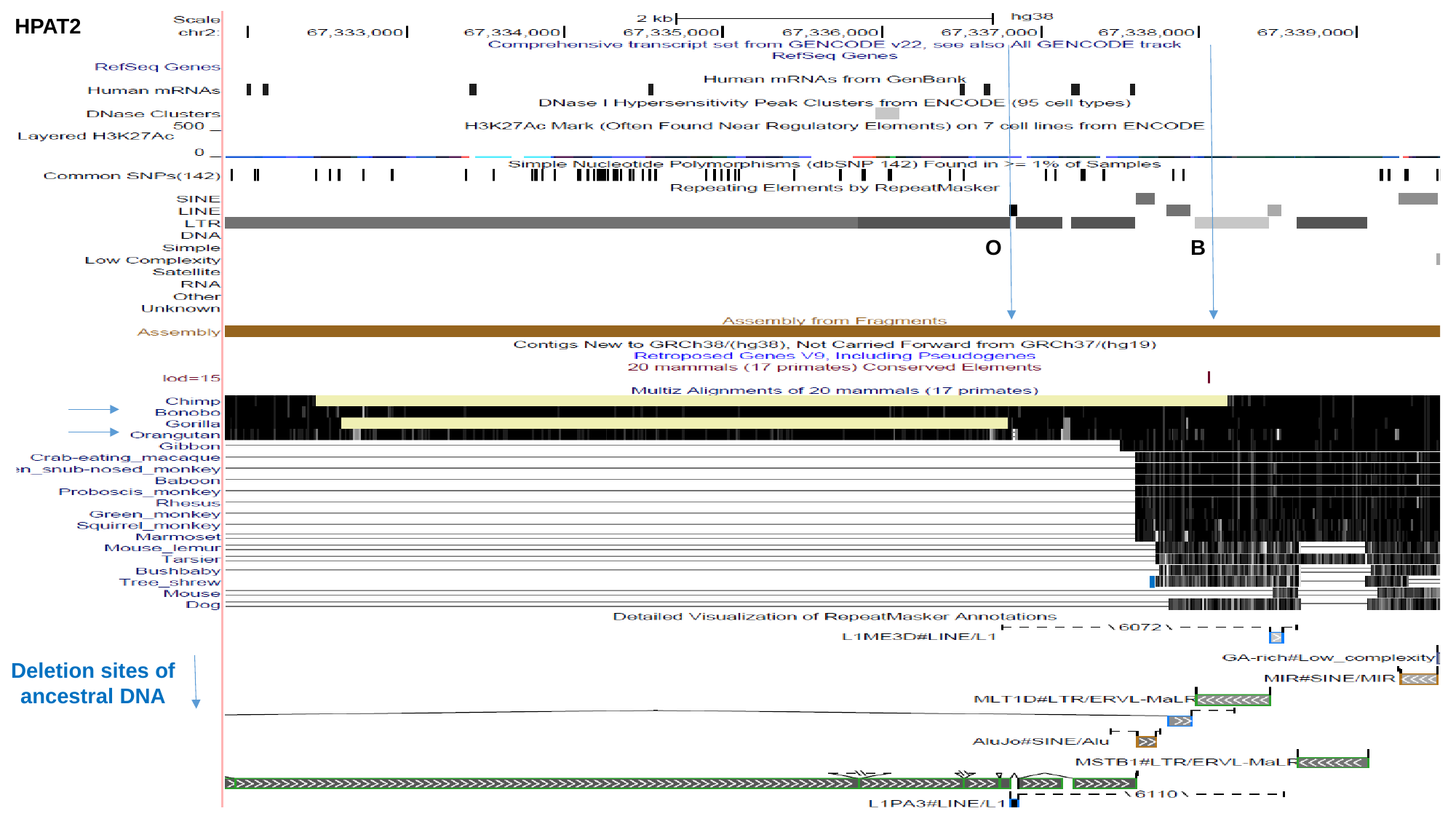

HPAT2
O
B
Deletion sites of ancestral DNA
