# Supplementary material for: Single cell expression analysis of primate-specific retroviruses-derived HPAT lincRNAs in viable human blastocysts identifies embryonic cells co-expressing genetic markers of multiple lineages
Source: Heliyon. 2018 Jun 28;4(6):e00667. doi: 10.1016/j.heliyon.2018.e00667 (PMC6039856; doi:10.1016/j.heliyon.2018.e00667)
Supplement: Supplemental Information Text [file mmc20.docx]

**Supplemental Information Text**

**Single cell expression analysis of primate-specific retroviruses-derived HPAT lincRNAs in viable human blastocysts identifies embryonic cells co-expressing genetic markers of multiple lineages**

**Authors**

Gennadi Glinsky^1†*^, Jens Durruthy-Durruthy^2,†^, Mark Wossidlo^3,†^, Edward J. Grow^4^, Jason L. Weirather^5^, Kin Fai Au^5^, Joanna Wysocka^4^, Vittorio Sebastiano^2,^*

^†^contributed equally

*correspondence

**Affiliations**

^1^Institute of Engineering in Medicine, University of California, San Diego

9500 Gilman Dr. MC 0435 La Jolla, CA 92093-0435, USA

^2^Department of Obstetrics and Gynecology, Institute for Stem Cell Biology & Regenerative Medicine, Stanford University, Stanford, CA 94305.

^3^Department of Cell- and Developmental Biology, Center of Anatomy and Cell Biology, Schwarzspanierstrasse 17, 1090 Vienna, Austria

^4^Department of Chemical and Systems Biology, Stanford University, Stanford, California, USA

^5^Department of Internal Medicine & Department of Biostatistics, University of Iowa, Iowa City, IA, USA

Correspondence:

Gennadi Glisnky, MD, Ph.D.

Institute of Engineering in Medicine

University of California, San Diego

9500 Gilman Dr. MC 0435

La Jolla, CA 92093-0435, USA

Email: [gglinskii@ucsd.edu](mailto:gglinskii@ucsd.edu)

Web: <http://iem.ucsd.edu/people/profiles/guennadi-v-glinskii.html>

**Supplemental Information Inventory**

1. Supplemental Note 1: HPAT expression-guided classification of 241 individual human embryonic cells recovered during differentiation of viable blastocysts.
2. Supplemental Figure S1 & Legend.
3. Supplemental Figure S1.1 & Legend.
4. Supplemental Figure S2 & Legend.
5. Supplemental Figure S2.2. & Legend.
6. Supplemental Figure S3 & Legend.
7. Supplemental Figure S4 Legend.
8. Supplemental Figure S5 & Legend.
9. Supplemental Figure S6 & Legend.
10. Supplemental Figure S7 & Legend.
11. Supplemental Figure S8 & Legend
12. Supplemental Figure S9 & Legend
13. Supplemental Table S1 (Excel): Description of data sets analyzed in this study.
14. Supplemental Table Set S2 (Excel): Estimated p values.
15. Supplemental Table S3 (Excel): Gene expression signatures.
16. Supplemental Table Set S4.1-S4.4: Genetic markers of major embryonic lineages in the MLME cells.
17. Supplemental Table S5: HPAT lincRNAs conservation analyses.
18. Supplemental Data Set S1 (Excel): Single-cell analysis of lincRNAs in 241 cells recovered from viable human blastocysts.
19. Supplemental Data Set S2 (Excel): Single-cell analysis of mRNAs in 241 cells recovered from viable human blastocysts.
20. Supplemental Text, Supplemental Figures’ Legends, Extended Experimental Protocols of the single cell expression profiling analyses of human blastocyst differentiation.
21. Supplemental Movie (related to the Figs. 1 - 6).

**Supplemental Figure Legends**

**Supplemental Figure S1.** Single-cell gene expression analysis of HPAT lincRNAs captures the entire spectrum of a cellular diversity during human blastocyst differentiation.

1. A high complexity expression matrix of 89 genes expression of which were measured in 241 individual human blastocyst cells.
2. Expression profiles of the *HPAT21; HPAT15*; and *HAPT2* lincRNAs define major sub-populations of human blastocyst cells. A color-coded stacked area plot depicts relative expression values of designated HPAT lincRNAs in each individual blastocyst cell. Note that there is only one cell manifesting *HPAT21/HPAT2/HPAT15*-null phenotype.
3. Expression patterns of HPAT lincRNAs in distinct sub-populations of human blastocyst cells. Stars designate sub-populations harboring the significantly enriched numbers of cells expressing defined genetic markers. P values were estimated using two-tailed Fisher’s exact test and reported in the Supplemental Table S2.
4. Expression patterns of pluripotency regulatory genes in distinct sub-populations of human blastocyst cells. Stars designate sub-populations harboring the significantly enriched numbers of cells expressing defined genetic markers.
5. Cell identity gene expression signature (GES) of the *HPAT21 ^(+)^ HPAT15 ^(+)^ HPAT2 ^(+)^* cells recovered from human blastocysts. Stars designate transcripts encoded by the genomic loci that were implicated in the regulation of the human pluripotency state. Positions of colored lines’ heights inside the circle reflect the percentage of cells within a population that express transcripts encoded by the corresponding genes names of which are listed on the circle.
6. Cell identity gene expression signature (GES) of the *HPAT21 ^(+)^ HPAT15 ^(-)^ HPAT2 ^(+)^* cells recovered from human blastocysts. Stars designate transcripts encoded by the genomic loci that were implicated in the regulation of the human pluripotency state. Positions of colored lines’ heights inside the circle depict the percentage of cells within a population that express transcripts encoded by the corresponding genes listed on the circle.

**Supplemental Figure S1.1.** Single cell analysis of expression patterns of HPAT lincRNAs during human blastocyst differentiation.

Expression patterns of HPAT lincRNAs in distinct sub-populations of human blastocyst cells. Stars designate sub-populations harboring the significantly enriched numbers of cells expressing defined genetic markers. P values were estimated using two-tailed Fisher’s exact test and reported in the Supplemental Table S1.

**Supplemental Figure S2.** Single-cell analysis of expression patterns of the lineage-specific genetic markers in HPAT lincRNA expression-defined sub-populations during human blastocyst differentiation.

Expression changes of designated lineage-specific genetic markers (see text for details) were evaluated in individual cells comprising the corresponding HPAT expression-defined sub-populations that were recovered from the early-stage and late-stage human blastocysts. Differentiation patterns in human blastocyst cells of genetic markers of PE-like cells are shown in (A) and (C). Differentiation patterns of genetic markers of TE-like cells are shown in (B). Note that HPAT21 ^(+)^ HPAT15 (+ HPAT2 ^(-)^ cells manifest gene expression differentiation patterns typical of PE-like cells (A), while HPAT21 ^(+)^ HPAT15 ^(-)^ HPAT2 ^(-)^ cells exhibit gene expression differentiation patterns typical of TE-like cells (B). In contrast, no gene expression differentiation patterns resembling either PE-like or TE-like cells were observed in HPAT2 ^(+)^ cell sub-populations (C).

(D) HPAT2 ^(+)^ cell sub-populations manifest significant enrichment of cells expressing genetic markers of all three major lineages created during human blastocyst differentiation. .

(E) HPAT lincRNA expression patterns in 241 individual human blastocyst cells. Each number on the circle designates one randomly placed blastocyst cell.

(F) HPAT lincRNA expression patterns in 88 individual cells recovered from the early-stage human blastocysts. Each number on the circle designates one randomly placed early-stage blastocyst cell.

(G) HPAT lincRNA expression patterns in 153 individual cells recovered from the late-stage human blastocysts. Each number on the circle designates one randomly placed late-stage blastocyst cell.

In the Figs. 2E-G each number on the circle corresponds to a single blastocyst cell. The colored lines inside the circle depict the corresponding *HPAT* lincRNAs and positions of the lines’ heights reflect the *HPAT* lincRNAs’ expression values in corresponding cells. All human blastocyst cells are segregated into sub-groups based on common *HPAT*s’ expression patterns (Fig. 1). The numbers of cells comprising the corresponding *HPAT*’s expression-defined sub-populations of the early (Fig. 2F) and late (Fig. 2G) blastocysts are shown. The statistical significance of the observed expression changes between sub-populations or within a sub-population during differentiation were estimated based on comparisons of the numbers of positive and negative cells using two-tailed Fisher’s exact test. Stars designate sub-populations harboring the significantly different numbers of cells expressing defined genetic markers. P values reported in the Supplemental Table S2.

**Supplemental Figure S2.2.** Dynamics of expression changes in human preimplantation embryos of HERV regulatory loci associated with HPAT lincRNAs.

Panels (A-F) show the patterns of expression changes defined by the mean expression values of transcripts encoded by the designated loci at corresponding stages of human embryogenesis. Panels (G-J) report the expression values of designated transcripts in 156 individual cells recovered from the human embryos at the designated developmental stages. Results of the additional analyses are reported in the Supplemental Fig. S3.

**Supplemental Figure S3.** Single-cell next generation sequencing-defined dynamics of expression changes of LTR7/HERVH loci of the HPAT lincRNA genes during human preimplantation embryonic development.

Panels (A-E) show the patterns of expression changes defined by the mean expression values of transcripts encoded by the designated loci at corresponding stages of human embryogenesis. Panels (F-I) report the expression values of designated transcripts in 156 individual cells recovered from the human embryos at the designated developmental stages. Results of the additional analyses are reported in the Supplemental Fig. S2.2.

**Supplemental Figure S4.** Identification and characterization of telomerase-positive MLME cells created during human blastocyst differentiation.

1. Expression profiles of the human telomerase reverse transcriptase *(TERT)* gene transcript and *HPAT21; HPAT15*; and *HAPT2* lincRNAs in human blastocyst cells. A color-coded stacked area plot depicts relative expression values of the *TERT* gene transcript and designated HPAT lincRNAs in individual blastocyst cells. Note that a vast majority (89%) of *TERT* ^(+)^ cells emerged within the HPAT21 ^(+)^ HPAT15 ^(+)^ HPAT2 ^(+)^ & HPAT21 ^(+)^ HPAT15 ^(-)^ HPAT2 ^(+)^ sub-populations of human blastocyst cells.
2. Marked expansion of *TERT* ^(+)^ cells during human blastocyst differentiation. Note that there is only a single *TERT ^(+)^* cell was detected in the population of the early-stage blastocyst cells (~1%) whereas the *TERT* gene expression was detected in a relatively large sub-population (11%) of the late-stage blastocyst cells. The inset lists genes that are expressed in a vast majority (89% to 100%) of *TERT ^(+)^* cells recovered from human blastocysts.
3. Cell identity gene expression signature (GES) of *TERT ^(+)^* cells recovered from human blastocysts. The list includes genes that are not expressed in *TERT ^(+)^* cells and genes that are expressed in at least two-third of *TERT ^(+)^* cells (from 67% to 100% of telomerase-positive cells). Stars in (C) and (D) designate genes that were identified as pluripotency regulators, inducers, or markers of the pluripotent state, including eight HPAT lincRNAs that are expressed in *TERT ^(+)^* cells. Right brace in (C) designates eight lincRNAs expression of which appears repressed *TERT ^(+)^* cells. Positions of the blue line inside the circle reflect the percentage of cells within the TERT ^(+)^ population that express transcripts encoded by the corresponding genes.
4. List of genes that are expressed in 6%-56% of *TERT ^(+)^* cells recovered from human blastocysts. Bar heights correspond to the percentage of *TERT ^(+)^* cells expressing designated genes.
5. Visualization of *TERT ^(+)^* putative immortal ground-state pluripotency precursor cells using the 3D reconstructed blastocyst model that was utilized to design the first web-based online tool to study early cell fate decisions in the human blastocyst, which is available online at <http://web.stanford.edu/~sunilpai/HumanBlastocystViewer.html> (Firefox/Chrome compatible). A single frame of the 3D blastocyst sphere is shown that captured ten TERT ^(+)^ human late blastocyst cells. Expression patterns of other genetic markers in individual human blastocyst cells located within the same field of the 3D reconstructed blastocyst sphere are shown. The extended report of this analysis is available in the Supplemental Movie.

**Supplemental Figure S5.** Dynamics of telomerase expression in human preimplantation embryos.

The figure reports fold changes of increased telomerase expression and associated P values in E5.Pre-lineage cells compared with E5.EPI; E5.PE; and E5.TE cells.

**Supplemental Figure S6.** Spatiotemporal dynamics of *TERT* expression and individual *TERT* (+) cells prevalence in human preimplantation embryos.

*TERT* mRNA expression patterns were evaluated in 1,529 individual human embryonic cells recovered from different stages of preimplantation embryogenesis and assigned to different developmental lineages based on their gene expression profiles [Petropoulos et al., 2016]. These analyses demonstrate that *TERT* (+) cells could be observed among all lineages and are readily detectable as early as at the E3-E4 stages followed by the expansion during the E5 stage coincidently with blastocyst formation (A; B; E; F). E5 pre-lineage cells manifest significantly increased levels of *TERT* mRNA expression compared to EPI, PE, and TE lineages (C; D). The second peak of *TERT* activity was observed at the E6 (B; E) in all three major lineages created during human preimplantation embryonic development. E5.early cell population has the highest level of *TERT* mRNA expression and contains the largest percentage of *TERT* (+) cells compared to other stages of human preimplantation embryonic development (B; F).

**Supplemental Figure S7.** Identification and characterization of TERT (+) cells manifesting the MLME phenotype and representing the human embryonic cells’ population harboring putative immortal multi-lineage precursor cells (iMPCs). The MLME cells were identified and characterized using independent validation sets of 1,708 individual human embryonic cells recovered from more than 100 human embryos at distinct stages of preimplantation human embryonic development.

A. Forty-six gene expression signature of the MLME cells (putative iMPC population) identified in the single-cell gene expression profiling experiments of 241 human blastocyst cells recovered from 32 human embryos (discovery data set; Supplemental Figs. S1 - S5; Supplemental Note 1) was utilized to identify the MLME-resembling cells among the 1,529 individual embryonic cells recovered from 88 human embryos at distinct stages of preimplantation embryonic development (validation data set 1; Supplemental Table S1 and ref. 34).

B. A graphical summary of the population-based correlation score analyses. Note that E5.Pre-lineage cells and E5.Early TERT (+) cells manifest the most significant enrichment for the MLME-like cells in the validation data set 1. Correlation patterns of the forty-six gene MLME signature defined by gene expression ratios of TERT (+) cells versus EPI cells (discovery data set) and E5.Pre-lineage cells versus E5.EPI cells (validation data set 1). High positive values of the correlation score indicate the resemblance to the MLME gene expression profile. Similarly, correlation scores were calculated for the 46-gene MLME signature defined by the gene expression ratios of TERT (+) cells versus EPI/PE/TE cells (discovery data set) and E5.Pre-lineage cells versus E5.EPI/PE/TE cells (validation data set 1); for the 46-gene iMPC signature defined by the gene expression ratios of TERT (+) cells versus EPI/PE/TE cells (discovery data set) and E5.Early TERT (+) cells versus E5 cells (validation data set 1); and for the forty-six gene MLME signature defined by the gene expression ratios of TERT (+) cells versus EPI/PE/TE cells (discovery data set) and E5.Early TERT (+) cells versus E5 cells (validation data set 1). Finally, correlation score analysis of the 46-gene MLME signature defined by the gene expression ratios of the TERT (+) cells versus EPI/PE/TE cells (discovery data set) and E3. TERT (+) cells versus E3 cells (validation data set 1). Note that in this instance no significant correlation was observed.

C. Correlation score patterns of the forty-six gene MLME signature defined by the gene expression ratios of the TERT (+) cells versus EPI cells (discovery data set), which were assessed in 819 individual TERT (+) cells identified in the validation data set 1.

D. Correlation score patterns of the forty-six gene iMPC signature defined by the gene expression ratios of the TERT (+) cells versus EPI cells (discovery data set) which were assessed in 819 individual TERT (+) cells identified in the validation data set 1. Cells were sorted in ascending order of correlation scores within corresponding developmental stages (top panel). Cells were placed in the ascending order of the individual identification numbers from E3 (left) to E7 (right) stages of embryonic development. Bottom panel shows the zoom-in view of the single-cell analysis of a subset of cells of the validation data set 1, which were segregated at a cut-off value of correlation score 0.4. Percentage values are the percent of individual cells within a population with the correlation score > 0.5. Using this approach, a total of 158 TERT (+) human embryonic cells manifesting correlation scores > 0.5 were identified in the validation set 1.

E. Twenty-three gene signature comprising a sub-set of genes of the 46-gene signatures that manifest significantly different expression levels in 158 TERT (+) cells (r > 0.5) compared to 661 TERT (+) cells (r < 0.5) in the validation data set 1 (p , 0.05). Using the gene expression profile of the 23-gene signature, correlation scores were calculated for 819 TERT (+) cells of the validation data set 1 (red colored bars). Blue bars designate top-scoring twenty-five TERT (+) cells manifesting correlation scores r > 0.55.

F. Similarity patterns of gene expression signatures (GES) defining putative immortal multi-lineage precursor cells identified in the Durruthy-Durruthy et al. discovery data set (top two figures), in the Petropolous et al. validation data set 1 (bottom left figure), and in the Yan et al. validation data set 2 (bottom right figure). The top two figures show GES of the HPATpos cells (left) and TERT (+) cells (right) identified in the Stanford discovery data set. The bottom left figure shows GES of the top-scoring TERT (+) cells identified in the Petropolous et al. validation data set 1 (Supplemental Table S1) based on the correlation scores of the 23-gene signature. The bottom right figure shows GES of the top-scoring TERT (+) cells identified in the Yan et al. validation data set 2 (Supplemental Table S1) based on the correlation scores of the 23-gene signature. The genes comprising the GES were identified in the single cell expression profiling experiments of VHB (the Stanford discovery data set). The gene names listed around the circles were grouped into relevant functional categories and placed in the same orders in all four figures. The numerical values corresponding to each gene indicate the percentage of positive cells which were identified using defined expression thresholds within the corresponding populations (gene expression values above null in the discovery data set and median expression values for non-MLME-resembling cells in validation data sets).

G. Consensus 863-gene expression signature of the TERT (+) human embryonic multi-lineage precursor cells (iMPC). A total of 863 genes (241 up-regulated and 622 down-regulated genes) manifesting highly concordant gene expression profiles (r = 0.887; top left figure) in the TERT (+) human embryonic multi-lineage precursor cells were identified using two independent validation data sets (Petropolous et al. validation data set 1 and Yan et al. validation data set 2). Concordant expression profiles of 241 up-regulated genes of the consensus gene expression signature of MLME cells are shown in the top right figure. The inset highlights concordant expression patterns of four key pluripotency regulatory genes (TERT; TCFP2L1; LIN28A; and ZFP42). Bottom left figure shows expression patterns of 30 genes encoding mitochondrial ribosomal proteins, expression of which is significantly increased in both populations of TERT (+) human embryonic iMPC identified in two independent validation data sets. Bottom right figure documents distinct genomic origins of 241 up-regulated (blue bars) and 622 down-regulated (red bars) genes comprising the consensus gene expression signature of the TERT (+) human embryonic iMPC. Note that transcription of 87.6% up-regulated genes originates from the embryonic genome, while transcriptional origins of 82.9% down-regulated genes were assigned to the maternal genome. The assignments of genomic origins of 863 genes were performed based on single cell gene expression profiling of twenty-six human embryonic cells reported in the validation data set 4 (Supplemental Table S1).

H. Timelines of creation of TERT (+) embryonic immortal multi-lineage precursor cells (iMPC) during the preimplantation embryogenesis in humans and mouse. The top left & right figures show the human embryogenesis timelines based on the assessments of the top-scoring TERT (+) cells identified in the Petropolous et al. validation data set 1 (Supplemental Table S1), which were selected based on the correlation scores of the 23-gene signature for top 3% TERT (+) cells (top left figure) and top 5% TERT (+) cells (top right figure). The bottom left figure reports the human embryogenesis timeline based on the top-scoring TERT (+) cells identified in the Yan et al. validation data set 2 (Supplemental Table S1), which were selected based on the correlation scores of the 23-gene signature for 8% of TERT (+) cells. The estimated numbers of the likelihood of appearance of the human iMPC-resembling cells in a single human embryo were calculated based on the assumption that the numbers of human embryonic cells were 8; 16; 32; 64; and 128 at the E3; E4; E5; E6; and E7 stages, respectively. The human MLME-like cells were defined as TERT (+) cells comprising the top 3% (top left figure) and top 5% (top right figure) of the 819 TERT (+) cells defined based on the 23-gene signature correlation scores in the validation data set 1 (top two figures). The proportions of cells in the E4.early versus E4.late and E5.early versus E5.late populations within the validation set 1 were estimated from the data reported in [34]. The human MLME-like cells reported in the bottom left figure were defined as TERT (+) cells comprising the top 8% (bottom left figure) of cells defined based on the 23-gene signature correlation scores in the validation data set 2. Bottom right figure shows the mouse embryogenesis timeline of creation of MLME-like cells based on the top-scoring TERT (+) cells identified in the mouse preimplantation embryogenesis data set (Supplemental Table S1) comprising 259 individual embryonic cells. The top-scoring 13.9% of TERT (+) mouse embryonic cells were identified based on the correlation scores of the human MLME consensus 863-gene expression signature (Panel G).

I. A model of principal molecular events contributing to regulation of the naïve pluripotency induction in vivo during transition from TERT (+) iMPC to pluripotent epiblast and hESC in human embryos. The first wave of increased expression of 5 most abundant in human blastocysts HPAT lincRNAs (highlighted by the green arrow) is triggered by the master pluripotency transcription factors (POU5F1/OCT4; TCFP2L1/LBP9; NANOG). The positive feed-back regulatory loop mediated by the activity of HPAT lincRNAs (red arrows) increases expression of master pluripotency transcription factors and naïve pluripotency inducers (MCRS1; TET1; THAP11), which triggers the second wave of increased expression of a multitude of TGE-derived lincRNAs (HPATs; LTR7/HERVH; and LTR5HS/HERVK families). Increased activities of TGE-derived lincRNAs make a critical contribution to the embryonic cells’ chromatin remodeling by enabling transitions to thermodynamically-stable triple-stranded state of double helix and facilitating targeted delivery of POU5F1/OCT4 & Mediator proteins to thousands of genomic loci. A second wave of the positive feed-back regulatory loop mediated by the activities of TGE-derived lincRNAs increases expression of key naïve pluripotency regulators. Top two figures show the graphical summary of the effects of shRNA-mediated targeted knockdown of LTR7/HERVH lincRNAs in hESC (15) inducing statistically significant changes in expression of genes encoding naïve pluripotency regulators (top left figure) and genetic markers of TE and PE lineages (top right figure). Only statistically significant gene expression changes are reported (P < 0.05). Experimental evidence supporting the model are reported and discussed in the text.

**Supplemental Figure S8.** Statistically significant categories (GO biological processes) of up-regulated (blue bars) and down-regulated (black bars) genes differentially regulated in human embryonic MLME cells containing putative immortal multi-lineage precursor cells (iMPCs).

GO, gene ontology. Identities of genes comprising corresponding gene expression signatures of the MLME cells are listed in the Supplemental Table S3.

**Supplemental Figure S9.** An overview of identification and characterization of novel primate-specific transposable element-derived lincRNAs playing mechanistically distinct roles of human preimplantation development and contributing to the pluripotency maintenance and UCSC Genome Browser windows revealing systematic deletion patterns of ancestral DNA in primate-specific retrotransposon-derived lincRNAs expressed in human embryos.

**Extended Experimental Protocols**

**Assay performance validation**

Primers were designed intron-spanned to avoid amplification of possible contaminating genomic DNA. Each primer pair was tested prior use for single-cell gene expression analysis for efficiency, sensitivity and specificity as well as to determine the expected melting temperature (T_m_) for the specific amplicon for each assay as previously described [17].

**Single-cell qPCR**

We used the C1 Single-Cell Auto Prep System (Fluidigm, Inc.) for single-cell capture and pre-amplification according to the manufacturer’s instructions (protocol # PN 100-4904). Briefly, we prepared a pool of all primers (500 nM). We then prepared lysis final mix, Reverse-Transcriptase (RT) final mix and PreAmp final mix on stored them on ice. Next, the C1 IFC chip for medium single cells (10 – 17 µm, barcode 1782x) was primed: 200 µl of C1 harvest reagent, preloading reagent, blocking reagent and wash buffer was loaded onto the chip, placed into the C1 Single-Cell Auto Prep System and the script “Prime (1782x)” was run. Priming lasted 20 min and cells were prepared in the meantime as described. Then, 12 µl of single-cell suspension was mixed with 8 µl of C1 Cell Suspension Reagent (Fluidigm, Inc.). After priming, blocking and priming solutions were removed and 10 µl of cell mix was loaded onto the C1 chip. The C1 chip was placed back into the instrument and the script “Cell Load (1782x)” was run. After cell capturing the C1 chip was removed and single-cell capturing was evaluated on a microscope. Empty capture sites were noted and the C1 chip was loaded with 4x 150 µl harvest reagent, 7 µl lysis final mix, 7 µl RT final mix and 24 µl PreAmp final mix. The chip was placed back into the instrument and the script “PreAmp (1782x)” was run with the following settings - reverse transcription: 25ºC (600 sec), 42ºC (3600 sec); pre-amplification: 95ºC (600 sec), 18 cycles of (95ºC (15 sec), 60ºC (240 sec)), 4ºC (hold). After pre-amplification, the C1 chip was removed from the instrument and 3 µl of cDNA (for each single cell) was harvested and diluted in 25 µl DNA Suspension buffer (Fluidigm, Inc.). Pre-amplified samples were then subsequently used on the Biomark HD using the protocol # PN 100-3488 and starting with the preparation of sample and assay mix (see “*RNA isolation and gene expression analysis of bulk samples with qPCR*” below).

**Determine limit of detection (LOD) value**

Because of the lognormal distribution described by Bengtsson *et al.* ([Bengtsson et al., 2005](#_ENREF_1)) and others, single-cell data are best viewed as expression level above detection limit on a log scale. For qPCR data we determine the log base 2 and defined Log_2_Ex with Log_2_Ex = LOD Ct – Ct raw [of gene]. We used bulk RNA and the dilution series of generated cDNA samples to calculate LOD Ct as follows: mean Ct and standard deviations for each assay (6 replicates) were calculated for all serial dilutions. Average Ct values with SD > 1 determined the threshold that was assigned to the limit of detection for each assay. We finally calculated the median of all LOD Ct values across all assays to determine a universal LOD Ct score of 27, which was used throughout this study.

**Quality assessment and normalization of single-cell expression values**

Melting curves were analyzed and false positive signals excluded. Chip to chip variation was assessed with 2 IFCs (2 rounds of late blastocysts) to identify assays that significantly change across different IFC chips. We excluded 7 assays for subsequent analysis since they did not correlate within an acceptable range between the three 2 IFCs and did not pass quality assessment. Then, raw Ct values were converted to expression levels using Log_2_Ex = LOD Ct – Ct raw [of gene] with LOD Ct = 27. Values with Log_2_Ex < 0 were excluded. Genes expressed in fewer than 5 % of single cells were eliminated as well. Single cells with Log_2_Ex lower than 3x SD of an assay across all cells were labeled apoptotic and were excluded. 134 cells were removed from further analysis due to failed quality assessment, resulting in 241 cells. We normalized such that each cell has the same median Log_2_Ex value across all genes detected in that cell. This ensured that the normalization factor included data from all genes in the study. For this study we generated a high quality data matrix of 241 genes across 89 assays resulting in 21,449 single cell expression values that was used for data analysis (Table S2).

**Data analysis**

We used R (version 3.1.2, Matlab (version 8.4.0) and GraphPad Prism 6 for all multivariate single-cell data analysis, statistical computing and graphic visualizations.

**Source and procurement of human embryos**

Supernumerary human blastocysts from successful (*in vitro* fertilized) IVF cycles, donated for basic research, were obtained with written informed consent from the Stanford University RENEW Biobank. De-identification was performed according to the Stanford University Institutional Review Board approved protocol #10466 entitled ‘The RENEW Biobank’ and the molecular analysis of the embryos was in compliance with institutional regulations.

**Immunofluorescence on human preimplantation embryos and embryonic stem cells**

For immunostaining of early and late stage human blastocysts, the *zona pellucida* was removed by Acidic Tyrode’s solution (Millipore) and embryos were fixed in 4% PFA in PBS for 20 min at 4⁰ C. After permeabilization in 0.2% Triton-X, 0.1% BSA in PBS for 10 min at RT blastocysts were blocked overnight in 0.1% BSA in PBS at 4⁰ C. Embryos were then incubated with primary antibodies in blocking solution for 3-4 h at RT at following conditions: 1:200 OCT4 (goat, Santa Cruz), 1:100 NANOG (rabbit, ReproCell), 1:200 CDX2 (mouse, Abcam), 1:200 GATA4 (goat, abcam), 1:250 MCRS1 (rabbit, Santa Cruz) and 1:500 THAP11 (mouse, Abcam). After several washes in blocking solution at RT blastocysts were incubated with secondary antibodies using 488-, 568- or 647 Alexa Fluor conjugates (Invitrogen) at 1:500 dilution for 1-2 h at RT. Following several washes in blocking solution embryos were stained with DAPI for 10 min.

For immunostaining of human primed and MTTH-overexpressed embryonic stem cells, cells were fixed in 4% PFA in PBS for 20 min at RT on a silanized slide. After permeabilization in 0.2% Triton-X, 0.1% BSA in PBS for 15 min at RT, cells were blocked overnight in 0.1% BSA in PBS at 4⁰ C. Primary antibody staining in blocking solution was done using 1:500 H3K9me3 (rabbit, Actif Motif) for 2h at RT. After several washes in blocking solution at RT secondary antibody staining with 1:500 Alexa Fluor 568 (Invitrogen) was done 1h at RT. Following several washes in blocking solution cells were stained with DAPI for 5 min.

Images were acquired using a Zeiss LSM510 Meta inverted laser scanning confocal microscope and computations of z-stack images were processed as described previously ([Wossidlo et al., 2011](#_ENREF_7)).

**hESC culture and induction of naive pluripotency *in vitro***

Conventional (primed) human iPSC lines C1 (Whitehead Institute Center for Human Stem Cell Research, Cambridge, MA) ([Hockemeyer et al., 2008](#_ENREF_4)) were maintained on mitomycin C inactivated MEF feeder layers and passaged mechanically using a drawn Pasteur pipette or enzymatically by treatment for 20 min with 1 mg/ml Collagenase type IV (GIBCO) followed by sequential sedimentation steps in human ESC medium (hESM) to remove single cells. C1 hESCs were cultured in hESM—DMEM/F12 (Invitrogen) supplemented with 15% FBS (Hyclone), 5% KSR (Invitrogen), 1 mM glutamine (Invitrogen), 1% nonessential amino acids (Invitrogen), penicillin-streptomycin (Invitrogen), 0.1 mM β-mercaptoethanol (Sigma), and 4 ng/ml FGF2 (R&D systems). Human ESC line H1 and derived iPSC lines were maintained in feeder-free conditions and cultured in basal mTeSR1 medium (STEMCELL Technologies) supplemented with 5x mTeSR1 supplement (STEMCELL Technologies). Cells were maintained in culture by daily media change and enzymatically passaged at 1:2 to 1:5 dilutions with pre-warmed Accutase (Innovative Cell Technologies). Differentiated cells were removed and/or cleaned under a laminar flow dissection hood. All cultures were maintained at 37°C, 5 % CO_2_ and 4 % O_2_.

For conversion of preexisting primed human ESCs, se seeded 2 x 10^5^ trypsinized single cells on a MEF feeder layer in hESC medium supplemented with ROCK inhibitor Y-27632 (Stemgent, 10 μM). Two days later, medium was switched to 5i/L/A naïve hESC medium. Dome-shaped naive colonies appeared within 10 days and could be picked or expanded polyclonally using 3–5 min treatment with Accutase (GIBCO) on an MEF feeder layer. Naive human pluripotent cells were derived and maintained in serum-free N2B27-based media supplemented with 5i/L/A. Medium was generated as described ([Theunissen et al., 2014](#_ENREF_6)).

For transient expression experiments we transfected primed hESCs with 3 μg of circular *MCRS1*, *TET1* and *THAP11* constitutive expression plasmids. Two days later, medium was switched to 2i/L conditions ([Takashima et al., 2014](#_ENREF_5)). At day 4, cells were retransfected (nucleofection), and on day 7 cells were assayed for gene expression and reporter activity. Naive human ESCs were cultured on mitomycin C-inactivated MEF feeder cells and were passaged every 5–7 days by a brief PBS wash followed by single-cell dissociation using 3–5 min treatment with Accutase (GIBCO) and centrifugation in fibroblast medium (DMEM [Invitrogen] supplemented with 10% FBS [Hyclone], 1 mM glutamine [Invitrogen], 1% nonessential amino acids [Invitrogen], penicillin-streptomycin [Invitrogen], and 0.1 mM β-mercaptoethanol). For continues passaging (up to passage 4), cells were retransfected prior replating. For cells cultured in 5i/L/A conditions ([Theunissen et al., 2014](#_ENREF_6)) no transfection was necessary.

**Flow cytometry**

To assess the proportion of OCT4-∆PE-GFP+ human ESCs, a single cell suspension was filtered, washed once in PBS and re-suspended in PBS + 5 % FBS prior fluorescence-activated cell sorting (FACS) analysis.

**Transcriptome analysis**

Global transcriptome datasets from Takashima et al. and Theunissen et al. (including datasets from Chan et al., Yan et al., and Gafni et al.) were used for comparative gene expression analysis ([Chan et al., 2013](#_ENREF_2); [Gafni et al., 2013](#_ENREF_3); [Takashima et al., 2014](#_ENREF_5); [Theunissen et al., 2014](#_ENREF_6); [Yan et al., 2013](#_ENREF_8)).

Gene expression values of genes in this study were extracted from all reported datasets and normalized against expression values of identified NANOG+ epiblast cells (n = 22) in this study. Data were processed using Bioconductor packages in R.
